# A genome-wide CRISPR/Cas9 screen identifies calreticulin as a selective repressor of ATF6α

**DOI:** 10.7554/eLife.96979

**Published:** 2024-07-29

**Authors:** Joanne Tung, Lei Huang, Ginto George, Heather P Harding, David Ron, Adriana Ordonez

**Affiliations:** 1 Cambridge Institute for Medical Research (CIMR), University of Cambridge, Cambridge Biomedical Campus Cambridge United Kingdom; Centre de Recherche en Cancérologie de Marseille France; University of Oxford United Kingdom

**Keywords:** unfolded protein response, UPR, genome-wide CRISPR/Cas9 screen, ATF6⍺, calreticulin, CRT, endoplasmic reticulum, CHO-K1 cells, Other

## Abstract

Activating transcription factor 6 (ATF6) is one of three endoplasmic reticulum (ER) transmembrane stress sensors that mediate the unfolded protein response (UPR). Despite its crucial role in long-term ER stress adaptation, regulation of ATF6 alpha (α) signalling remains poorly understood, possibly because its activation involves ER-to-Golgi and nuclear trafficking. Here, we generated an ATF6α/Inositol-requiring kinase 1 (IRE1) dual UPR reporter CHO-K1 cell line and performed an unbiased genome-wide CRISPR/Cas9 mutagenesis screen to systematically profile genetic factors that specifically contribute to ATF6α signalling in the presence and absence of ER stress. The screen identified both anticipated and new candidate genes that regulate ATF6α activation. Among these, calreticulin (CRT), a key ER luminal chaperone, selectively repressed ATF6α signalling: Cells lacking CRT constitutively activated a BiP::sfGFP ATF6α-dependent reporter, had higher BiP levels and an increased rate of trafficking and processing of ATF6α. Purified CRT interacted with the luminal domain of ATF6α *in vitro* and the two proteins co-immunoprecipitated from cell lysates. CRT depletion exposed a negative feedback loop implicating ATF6α in repressing IRE1 activity basally and overexpression of CRT reversed this repression. Our findings indicate that CRT, beyond its known role as a chaperone, also serves as an ER repressor of ATF6α to selectively regulate one arm of the UPR.

## Introduction

The endoplasmic reticulum (ER), and its associated chaperones, constitutes the major cellular compartment for the synthesis, folding, and quality control of secretory proteins (Sun and Brodsky, 2019). An imbalance between synthesis and folding can lead to ER stress, potentially resulting in ER dysfunction and pathological conditions (Wang and Kaufman, 2016). To restore cellular homeostasis, adaptive pathways, collectively known as the unfolded protein response (UPR), are activated. The UPR is coordinated by three known ER stress transducers: inositol-requiring kinase 1 (IRE1), protein kinase R-like endoplasmic reticulum kinase (PERK), and activating transcription factor 6 (ATF6) (Walter and Ron, 2011). Of the three arms of the UPR, the mechanisms that govern ER stress-dependent ATF6 activation remain the least well understood, even though ATF6 mediates much of the ER-stress-induced changes in gene expression observed in vertebrates. Thus, ATF6 is specialised in the regulation of quality control proteins in the ER (Adachi et al., 2008) and complete loss of ATF6 impairs survival upon ER stress in cellular and animal models (Wu et al., 2007).

ATF6 is a type II transmembrane ER glycoprotein containing an N-terminal portion comprising its basic leucine zipper (b-ZIP), DNA-binding and transactivation domains and a C-terminal ER luminal domain (LD) that senses stress and regulates ATF6α activity . Vertebrates have two isoforms: ATF6α and ATF6β. The ATF6α isoform dominates the regulation of UPR target genes in mammalian cells and is the focus of this study. Unlike IRE1 and PERK, which remain in the ER after UPR activation, ATF6 translocates from the ER to the Golgi, where it is sequentially cleaved by two serine protease, site-1 (S1P) and site-2 (S2P) proteases (Haze et al., 1999) to release its soluble active N-cytosolic domain (N-ATF6α) that is further translocated into the nucleus. There, N-ATF6α acts as a potent but short-live transcription factor (George et al., 2020) that binds ER stress response elements (ERSE-I and -II) in the promoter regions of UPR target genes in complex with the general transcription factor NF-Y and YY1 to upregulate the expression of a large number of ER chaperones (Li et al., 2000; Yoshida et al., 2000). Among those, the gene encoding the ER Hsp70 chaperone BiP (*GRP78*) has been well documented as an important ATF6α target (Adachi et al., 2008).

At ER level, it has been proposed that ATF6α is retained by its association with BiP (Shen et al., 2002), yet the existence of additional factors influencing its ER localisation remains uncertain. Redox regulation has also been proposed to keep ATF6α in the ER as a mixture of monomeric and multimeric disulphide-linked forms (Nadanaka et al., 2007) that upon ER stress are reduced by protein disulphide isomerase (PDI) family members (such as ERp18 or PDIA5), promoting the transit of a reduced monomeric ATF6α population (Higa et al., 2014; Oka et al., 2019). The Coat Protein Complex II (COPII)-coated vesicles have been implicated in the trafficking of ATF6α (Schindler and Schekman, 2009; Lynch et al., 2012) and the ATF6α_LD’s *N*-glycosylation state has also been proposed to influence its ER trafficking (Hong et al., 2004), yet the details of these processed remain incompletely understood. Furthermore, it is widely recognised that a key step of ATF6α activation occurs in the Golgi, the site of proteolytic processing. This is reflected in the inability of cells lacking S2P to trigger BiP transcription in response to ER stress (Ye et al., 2000). Hence, although there is a relatively clear understanding of the events triggering ATF6α cleavage and activation upon reaching the Golgi, much remains unknown about the factors influencing ATF6α regulation in the ER.

ATF6α signalling also involves crosstalk with the IRE1 pathway. This is partly mediated by direct heterodimerisation of N-ATF6α with XBP1s, the active, spliced form (Yamamoto et al., 2007; Yamamoto et al., 2004). However, the relationship between the ATF6α and IRE1 pathway is complex: (1) XBP1 and IRE1 genes are transcriptional targets of activated ATF6α (Yoshida et al., 2001), (2) XBP1s and IRE1 have some different target genes (Shoulders et al., 2013), and (3) ATF6α activity can also repress IRE1 signalling (Walter et al., 2018).

Here, we established an ATF6α/IRE1 dual UPR reporter cellular model to conduct an unbiased genome-wide CRISPR/Cas9 screen focussed on ATF6α. Complemented by bioinformatic analysis, post-screen hit validation using targeted gene editing and *in vitro* biophysical assays we report on a comprehended analysis of a complex signalling pathway.

## Results

### A dual UPR reporter cell line to monitor ATF6α and IRE1 activity

**Figure 1.**
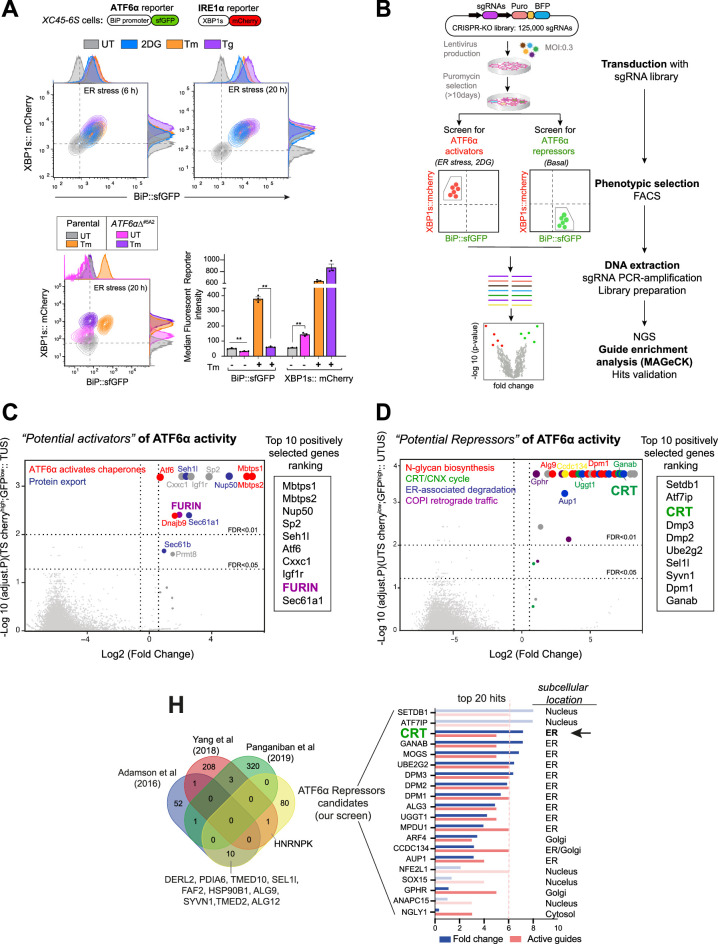
High-throughput CRISPR/Cas9 screens to identify new modulators of ATF6α signalling. (**A**) Characterisation of the *XC45-6S* cells, a dual unfolded protein response (UPR) reporter CHO-K1 cell line stably expressing XBP1s::mCherry to report on IRE1 activity and BiP::sfGFP to report on ATF6α activity was generated to perform the CRISPR/Cas9 screens. *Top panel*: Two-dimensional contour plots of BiP::sfGFP and XBP1s::mCherry signals in untreated cells (UT, grey) and cells treated with the endoplasmic reticulum (ER) stressors 2-deoxy-D-glucose (2DG, 4 mM, blue), tunicamycin (Tm, 2.5 µg/ml, orange), or thapsigargin (Tg, 0.5 µM, purple) over a short (6 hr) and long period of time (20 hr). Histograms of the signal in each channel are displayed on the oppsite axes. *Lower panel*: Contour plots as in ‘A, top panel’ from *XC45-6S* cells (parental) and a knockout *ATF6*αΔ clone (*ATF6*αΔ^#5A2^) generated by CRISPR/Cas9 gene editing. Cells were analysed under basal (UT) and ER stress conditions (Tm, 2.5 µg/ml, 20 hr). The bar graph shows the mean ± SEM of the median fluorescent reporter intensity normalised to untreated cells from three independent experiments. Statistical analysis was performed by a two-sided unpaired Welch’s *t*-test and significance is indicated by asterisks (**p < 0.01). (**B**) Flowchart illustrating the two parallel CRISPR/Cas9 screenings aimed to identify genetic ATF6α regulators. ATF6α/IRE1 dual reporter cells were transduced with a CHO sgRNA CRISPR knockout (KO) library. After puromycin selection, the transduced population was split into two subpopulations, subjected to either ER stress with 2DG treatment (4 mM) for 18 hr or left untreated (basal) to identify the activators and repressors of ATF6α, respectively. A BiP::sfGFP^low^;XBP1s::mCherry^high^ population under ER stress and a BiP::sfGFP^high^; XBP1s::mCherry^low^ population devoid of ER stress were collected through fluorescent-activated cell sorting (FACS) and processed to determine sgRNAs frequencies after two rounds of selection, expansion, and enrichment. (**C**) Volcano plot depicting the Log2 (fold-change) and the negative Log10 (adjusted p-value) of the genes targeted by sgRNAs in 2DG-treated-sorted (TS) cells. This analysis identified genes whose loss confers repression of the ATF6α reporter during ER stress without impacting ER-stress-induced IRE1, compared to treated-unsorted (TUS) cells. The table lists the top 10 genes positively selected in the ATF6α *activator screen*, with FURIN highlighted as a hit for further analysis. (**D**) Volcano plot, as in ‘C’, depicting the genes targeted by sgRNAs in untreated-sorted (UTS) cells. This analysis identified genes whose loss confers activation of the ATF6α reporter under basal condition, compared to untreated-unsorted (UTUS) cells. The table lists the top 10 genes positively selected in the ATF6α *repressor screen*, with calreticulin (CRT) highlighted as the hit for further focussed analysis. (**H**) Venn diagram depicting unique and common upregulated top genes examined in ‘D’, between our screen (yellow) and previous high-throughput UPR screenings (Adamson et al., 2016; Panganiban et al., 2019; Yang et al., 2018) to discard hits that were confirmed to be IRE1- or PERK-dependent UPR target genes. The Log2 (fold-change) and the number of active sgRNA are provided for the 20 genes that remained in our screen, to be put forth as possible ATF6α repressor candidates.

In vertebrates, the transcriptional activation of BiP is predominantly governed by ATF6α (Adachi et al., 2008; Adamson et al., 2016). To dynamically monitor ATF6α activity in live cells, a ‘landing pad’ cassette, containing recombination sites and expressing the *Cricetulus griseus* (*cg*) BiP promoter region fused with a super folder green fluorescent protein (BiP::sfGFP), was targeted to the *ROSA26* safe-harbour locus of Chinese hamster ovary cells (CHO-K1) using CRISPR/Cas9 (Gaidukov et al., 2018; Figure 1—figure supplement 1A) in a previously characterised cell line (*CHO-XC45*) in the lab carrying an integrated XBP1s::mCherry reporter transgene monitoring the IRE1 pathway (Harding et al., 2019). Flow cytometry analysis of these ATF6α/IRE1 dual UPR reporter cells (referred as *XC45-6S*) treated with ER stress-inducing agents such as tunicamycin (which inhibits *N*-glycosylation), 2-deoxy-D-glucose (which inhibits glycolysis), or thapsigargin (which depletes ER calcium), revealed time-dependent activation of both UPR arms with a broad dynamic range (Figure 1A, top panel, and Figure 1—figure supplement 1B). Additionally, the introduction of the reporter systems did not affect basal BiP protein levels when compared with plain CHO-K1 cells (Figure 1—figure supplement 1C).

**Figure 1—figure supplement 1.**
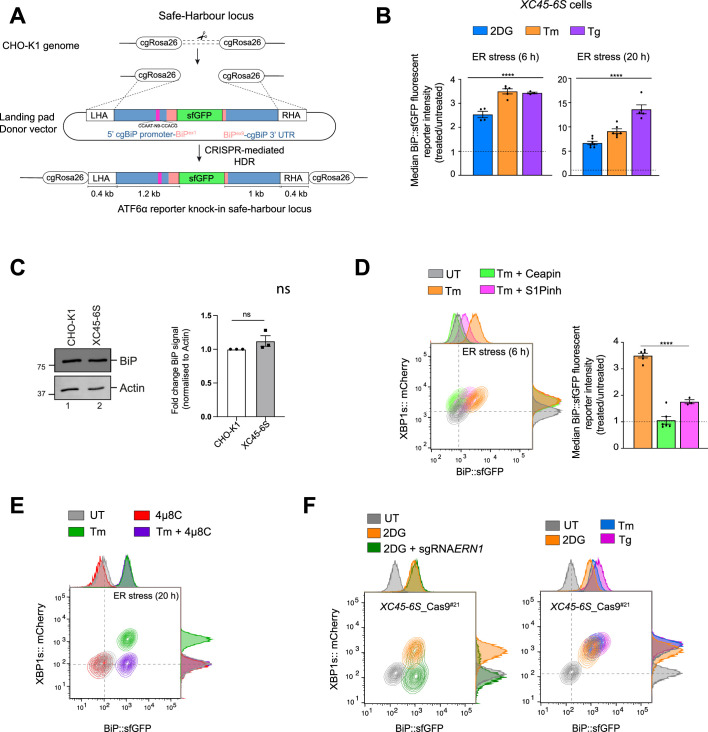
Characterisation of the ATF6α/IRE1 dual unfolded protein response (UPR) reporter cell (*XC45-6S*). (**A**) Schematic representation of the strategy for targeted integration at the *cgROSA26* safe-harbour locus in the CHO genome, mediated by CRISPR/Cas9-mediated homology-directed repair (HDR). The approach involves a unique sgRNA/Cas9 sequence targeting the *cgROSA26* locus (Gaidukov et al., 2018) and a landing pad donor plasmid containing homology arms (LHA and RHA) flanking a minigene encoding the promoter region of the *cgBiP* fused with the superfolder green fluorescent protein (sfGFP) to create a BiP::sfGFP reporter to monitor ATF6α activity in cells. (**B**) Related to Figure 1A, top panel. Quantification of fold-change BiP::sfGFP signals in *XC45-6S* dual UPR reporter cells under basal conditions (untreated) and treated with the endoplasmic reticulum (ER) stressors 2-deoxy-D-glucose (2DG), Tm, and Tg (6 or 20 hr). Shown is the normalised (treated/untreated) median fluorescence intensity (MFI) as mean ± standard error of the mean (SEM) of independent repetitions (*n* > 3). (**C**) Representative immunoblots of endogenous BiP protein levels in cell lysates from parental CHO-K1 cells and ATF6α/IRE1 dual UPR reporter cells (*XC45-6S*). The samples were also blotted for actin (loading control). The right graph bar shows the fold-change of BiP signal normalised to the signal of the loading control in three independent experiments indicated by mean ± SEM. (**D**) Two-dimensional contour plots of BiP::sfGFP and XBP1s::mCherry levels in *XC45-6S* cells under basal conditions (UT, grey) or after treatment with tunicamycin alone (2.5 µg/ml, Tm, orange) or in combination with Ceapin A7 (5 µM, green) or S1P inhibitor (15 µM, pink) for 6 hr. Histograms of the signal in each channel are displayed on the axes. The bar graph shows the mean ± SEM of the normalised median BiP::sfGFP reporter intensity from more than four independent experiments. (**E**) Contour plot, as in ‘D’, from parental cells treated with tunicamycin alone (Tm, 2.5 µg/ml, green) or in combination with the IRE1 inhibitor 4µ8C (10 µM, purple) for 18 hr. A representative dataset from two independent experiments is shown. (**F**) Characterisation of the ATF6α/IRE1 dual UPR reporter cell line used in the CRISPR/Cas9 screen after stable integration of Cas9. *Left panel*: Contour plots to confirm Cas9 stable integration. Cells were analysed under basal conditions (grey) or after treatment with 2DG alone (orange) or transfection with an individual lentiGuide-Puro plasmid (without expression of Cas9) targeting exon 18 of IRE1 (encoded by *cgERN1*, green). ER stress treatments with 4 mM 2DG lasted 24 hr. *Right panel*: As in ‘left panel’, but cells were treated with three different ER stressors for 20 hr: 2DG (4 mM), Tm (2.5 µg/ml), or Tg (0.5 µM). A representative dataset from two independent experiments is shown. All statistical analysis was performed by a two-sided unpaired Welch’s *t*-test and significance is indicated by asterisks (****p < 0.0001; ns: non-significant). Figure 1—figure supplement 1—source data 1.Raw images for gels shown in Figure 1—figure supplement 1.

**Figure 1—figure supplement 2.**
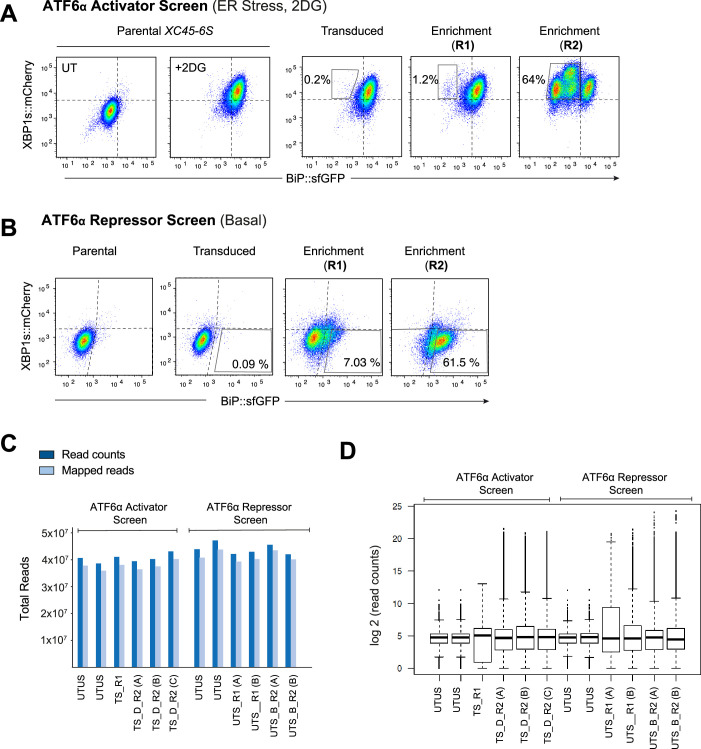
Enrichment method and quality control data analysis by MAGeCK of the CRISPR/Cas9 screens. (**A**) Two-dimensional contour plots of BiP::sfGFP and XBP1s::mCherry signals in *XC45-6S* obtained after each sort of enrichment (R1: round 1; R2: round 2) in the screen designed to identify ATF6α *activators*. The plots reveal a progressive enrichment of cells displaying CRISPR/Cas9-induced genetic perturbations that specifically impairing BiP::sfGFP activation upon unfolded protein response (UPR) induction with 2-deoxy-D-glucose (2DG) (4 mM). (**B**) Contour plots, as in ‘A’, representing the screen designed to identify ATF6α *repressors* by successive rounds of enrichments. The focus is on cells exhibiting CRISPR/Cas9-induced genetic perturbations that lead to the constitutive activation of the BiP::sfGFP reporter under basal conditions, while leaving the XBP1s::mCherry reporter unaffected. (**C**) Analysis of total read counts and mapped reads to the CHO library analysed by MAGeCK [UTUS: untreated and unsorted; TS: treated (plus 2DG) and sorted; R1: round 1 of enrichment; R2: round 2 of enrichment; D: dull green fluorescent protein (GFP) population; B: bright GFP population; A–C: pools of cells]. (**D**) Frequency distribution of sgRNA in each sample, showing the median-normalised read counts.

**Figure 1—figure supplement 3.**
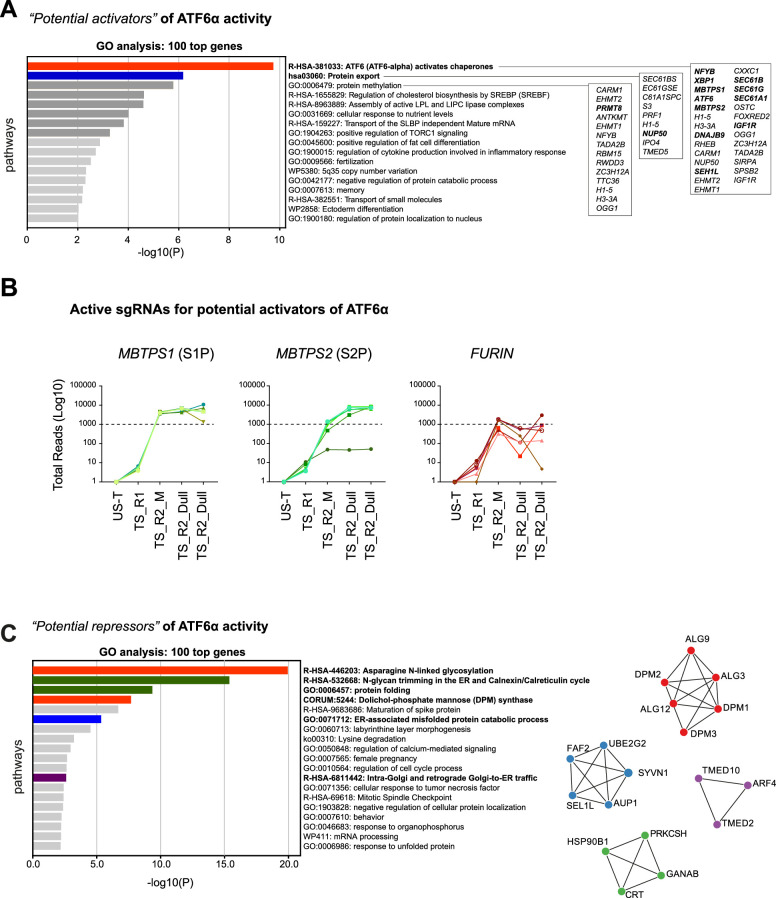
Integration of CRISPR screen hits. (**A**) Pathway enrichment analysis for biological processes using Gene Ontology (GO) terms for the top 100 hits identified in the CRISPR/Cas9 screen aimed to identify potential activators of ATF6α signalling. Selected pathways are ranked by p-value. (**B**) Total reads count enrichment through the selection process for each active sgRNA targeting the most significant genes identified in the *activator ATF6*α *screen*, including known regulators of ATF6α (*MBTPS1-*S1P and *MBPTPS2-*S2P) and those with possible roles (*FURIN*) in the activation of ATF6α signalling. (**C**) Pathway enrichment analysis, as in ‘A’, for the top 100 hits identified in the CRISPR/Cas9 screen aimed to identify potential *repressors* of ATF6α signalling. Selected pathways are ranked by p-value. On the left, a protein–protein interaction network (Metascape) for the 100 top hits identified in the CRISPR/Cas9 screen for potential repressors of ATF6α signalling is shown.

**Figure 1—figure supplement 4.**
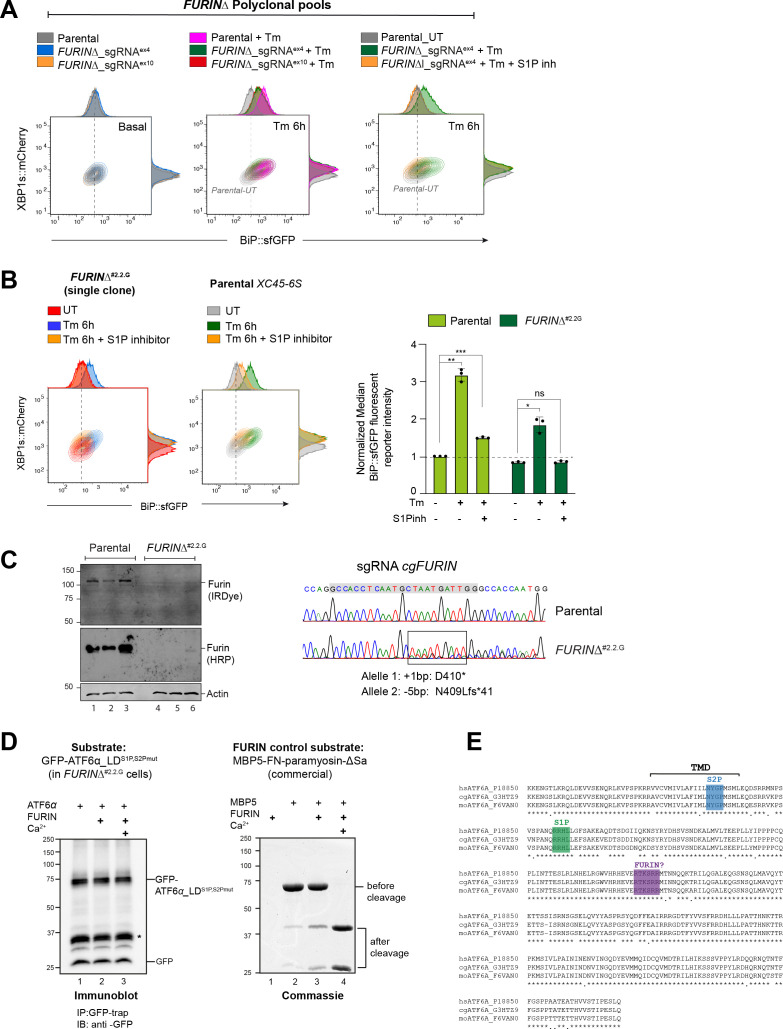
FURIN depletion decreases responsiveness to ATF6α activation upon endoplasmic reticulum (ER) stress. (**A**) Two-dimensional contour plots of BiP::sfGFP and XBP1s::mCherry signals in a sorted pooled population with FURIN depletion through CRISPR/Cas9 sgRNAs targeting exons 4 and 10. The analysis was conducted under basal conditions (left panel), after tunicamycin (Tm)-ER stress induction alone for 6 hr (middle panel) or in combination with S1P inhibitor treatment (15 µM) (right panel). Parental cells were used as a reference. A representative dataset from more than three experiments is shown. (**B**) Contour plots as in ‘A’ using parental cells and a single *FURIN∆* clone (#2.2.G). The cells were assessed under basal conditions (UT) or after treatment with tunicamycin alone (Tm, 2.5 µg/ml) or in combination with the S1P inhibitor (15 µM) for 6 hr. The bar graph shows the mean ± standard error of the mean (SEM) of the normalised median BiP::sfGFP reporter intensity from three independent experiments. All statistical analysis was performed by a two-sided unpaired Welch’s *t*-test and significance is indicated by asterisks (*p < 0.05; **p < 0.01; ***p < 0.001; ns: non-significant). (**C**) Immunoblot of whole-cell lysates from the indicated genotyped probed with antibodies against FURIN and the loading control actin. FURIN protein levels were determined using horseradish peroxidase (HRP) or IRDye fluorescently labelled secondary antibodies. On the right, DNA sequence chromatograms of FURIN-targeted region are represented, showcasing parental cells and an *FURIN∆* clone. The rectangle indicates where the FURIN indel mutation occurs, with the sgRNA/Cas9 sequence indicated in a grey rectangle. (**D**) FURIN cleavage assay. *Left panel*: *FURIN∆* cells were transfected with a plasmid encoding GFP-ATF6α_TM^S1P,S2P mut^-LD^WT^ to be used as substrate for *in vitro* FURIN cleavage reactions. Forty-eight hours after transfection, cells were lysed, ATF6α was immunoprecipitated (IP) using GFP-Trap Agarose and then subjected to FURIN cleavage by incubating with 1 U of commercial FURIN (#P8077S, NEB) for 6 hr at 25°C in the presence or absence of calcium (Ca^2+^). Beads were eluted in 2xLB-DTT, boiled for 5 min at 90°C and loaded into 10% sodium dodecyl sulfate–polyacrylamide gel electrophoresis (SDS–PAGE) gels. Membranes were immunoblotted (IB) with an anti-GFP antibody. The expected molecular weight of GFP-ATF6α_TM-^S1P,S2P mut^-LD^WT^ before cleavage is 67 and 45 kDa post-FURIN cleavage. *Right panel*: As in left panel but using an FURIN control substrate (MBP5-FN-paramyosin-ΔSa). MBP5FN-paramyosin-ΔSa (2.5 µg) was also incubated for 6 hr at 25°C in the presence or absence of calcium with 1 U of FURIN. The molecular weight of the MBP5-FN-paramyosin-ΔSal substrate before cleavage is 70.6 kDa. FURIN cleavage resulted in an MBP5 mass of 42.8 kDa and a mass of 27.8 kDa. All samples were subjected to SDS–PAGE, and bands were detected by Coomassie blue staining. (**E**) Amino acid sequence homology between residues 349 and 670 of human (hs), hamster (cg), and mouse (mo) ATF6α indicating the transmembrane domain (TMD) and the cleavage sites for S1P and S2P proteases. The FURIN putative cleavage site is highlighted in purple. The alignment was performed using the MacVector programme. Figure 1—figure supplement 4—source data 1.Raw images for gels shown in Figure 1—figure supplement 4.

Disruption of endogenous *cgATF6*α in *XC45-6S* cells by CRISPR/Cas9 genome editing abolished the responsiveness of the BiP::sfGFP reporter to ER stress and derepressed IRE1 signalling, both basally and in response to ER stress. These observations are consistent with ATF6α obligatory role in BiP expression and with findings that IRE1 signalling is repressed by ATF6α activity (Walter et al., 2018).

To further validate these UPR reporters, cells were subjected to tunicamycin treatment in the presence of Ceapin-A7 (a small molecule that blocks ATF6α activation by tethering it to the lysosome) (Gallagher et al., 2016) or an S1P inhibitor (PF429242) (Hawkins et al., 2008). Ceapin-A7 effectively blocked the activation of the ATF6α fluorescent reporter, whereas the S1P inhibitor partially attenuated the BiP::sfGFP signal in stressed cells (Figure 1—figure supplement 1D).

The selective IRE1 inhibitor 4μ8C (Cross et al., 2012) did not affect the ATF6α-dependent BiP::sfGFP reporter under ER stress conditions, but blocked the IRE1-dependent XBP1s::mCherry reporter (Figure 1—figure supplement 1E). Collectively, these observations confirm that the dual UPR reporter *XC45-6S* cell line enables independent monitoring of ATF6α and IRE1 activities, making it well suited for identifying additional modulators of ATF6α signalling by high-throughput techniques.

### Genome-wide CRISPR/Cas9 knock-out screens to profile ATF6α signalling

To identify regulators of ATF6α a library of pooled lentiviral single-guide RNAs (sgRNAs) targeting 20,680 predicted protein-coding genes in CHO cells, with ~6 sgRNAs per gene, was used (Ordóñez et al., 2021). Transduction of the *XC45-6S* ATF6α/IRE1 dual UPR reporter cells was performed at a coverage of ~640×, with a multiplicity of infection (MOI) of 0.3, intentionally set low to disfavour acquisition of more than one sgRNA by any one cell (Shalem et al., 2014). Non-transduced cells were purged by puromycin selection, and the pool of transduced cells was expanded for 10 days to favour gene editing and the establishment of loss-of-function phenotypes.

To systematically identify genes regulating the ATF6α transcriptional programme, two positive selection screens were conducted in parallel: a ‘loss-of-ATF6α function screen’ under ER stress conditions to identify activators/enhancers of ATF6α, and a ‘gain-of-ATF6α function screen’ under basal conditions to identify ATF6α repressors (Figure 1B). In the ATF6α *activator screen*, cells were stressed by exposure to 2-deoxy-D-glucose for 18 hr before fluorescent-activated cell sorting (FACS). Cells at the lowest 2% of BiP::sfGFP signal, with preserved IRE1 signalling (BiP::sfGFP^low^; XBP1s::mCherry^high^), were selected for further analysis. The search for genes whose inactivation derepresses ATF6α (ATF6α *repressor screen*) was conducted in the absence of ER stress. Here, cells exhibiting a constitutively active ATF6α reporter but only basal levels of the IRE1 reporter (BiP::sfGFP^high^; XBP1s::mCherry^low^) were selected for further analysis. Sorted cells from both screens underwent two rounds of progressive enrichment through cell culture expansion and phenotypic selection by sorting. As a result, a progressive increase in the proportion of BiP:sfGFP^low^ or BiP:sfGFP^high^ cells occurred in the respective screen (Figure 1—figure supplement 2A and Figure 1—figure supplement 2B).

The integrated sgRNAs were amplified from genomic DNA, and their abundance in the enriched populations after one or two rounds of sorting was estimated by deep sequencing and compared to a reference sample of transduced and unsorted population. MAGeCK bioinformatics analysis (Li et al., 2014) was used to determine sgRNA sequence enrichment, establishing the corresponding ranking of targeted genes (Supplementary file 1; see GEO accession number: GSE254745) and quality controls measures (Figure 1—figure supplement 2C and Figure 1—figure supplement 2D). Both screens were conducted at least in duplicate for all conditions.

### Expected and new candidate genes that contribute to ATF6α activation

MAGeCK-based analysis of sgRNAs enriched in cells with attenuated ATF6α signalling and intact activity of the IRE1 reporter, revealed that those targeting S1P and S2P proteases (encoding by the *MBTPS1* and *MBTPS2* genes) were the two most enriched genes (Figure 1C), validating the experimental methodology. Gene Ontology (GO) cluster analysis of the 100 most significantly enriched targeted genes revealed up to 19 enriched pathways (Figure 1—figure supplement 3A), with the top 2 pathways highlighted in Figure 1C being closely related to ATF6α signalling.

We focussed our attention on targeted genes that could act upstream to find early regulatory events of ATF6α. Therefore, targeted genes that were likely to act downstream and impact on the cleaved ATF6α form (N-ATF6α) were dismissed, including the *SP2* transcription factor, which shares the binding site with NF-Y (Völkel et al., 2015), and also genes involved in nuclear import, such as *NUP50* and *SEH1L* (Figure 1C and Figure 1—figure supplement 3A). Similar consideration was applied to *PRMT8*, as the family member *PRMT1* has been shown to enhance ATF6α transcriptional activity (Baumeister et al., 2005). Additionally, guides targeting *IGFR1* and *CXXC1* were also dismissed as likely affecting ER proteostasis indirectly via cellular growth (Pfaffenbach et al., 2012). Guides targeting the genes encoding the ER-resident proteins *SEC61A1*, *SEC61B* (both components of the translocon machinery), and the *DNAJB9*/ERDj4 co-chaperone were highly enriched. However, given their roles in selectively regulating IRE1 signalling (Adamson et al., 2016; Shoulders et al., 2013), we deemed their identification to reflect a bias towards IRE1 activators arising from the selection scheme for ATF6α^low^ and IRE1^high^ cells.

One of the genes that could plausibly play a proximal role and act upstream on ATF6α activation was the ubiquitous Golgi-localised protease FURIN (Figure 1C). Like S1P, FURIN belongs to the subtilisin-like proprotein convertase family (Nakayama, 1997; Van de Ven et al., 1991) and is thus poised to act at the level of ATF6α proteolytic processing. All six sgRNAs targeting *FURIN* were enriched in the BiP:sfGFP^low^ cells, a level of enrichment similar to that observed to the established ATF6α regulators *MBTPS1* (S1P) and *MBTPS2* (S2P) (Figure 1—figure supplement 3B). The genotype–phenotype relationship suggested by the screen was also observed upon targeting *FURIN* in ATF6α/IRE1 dual reporter cells, as both pools of targeted cells and an individual clone had diminished responsiveness of BiP:sfGFP to ER stress (Figure 1—figure supplement 4A, left and middle panels). Furthermore, the S1P inhibitor more effectively attenuated the BiP::sfGFP reporter in *FURIN*∆ cells compared to wild-type (WT) cells (Figure 1—figure supplement 4A, right panel, Figure 1—figure supplement 4B and Figure 1—figure supplement 4C).

These observations raised the possibility of redundant roles of FURIN and S1P in activating ATF6α by cleaving its LD. However, ATF6α_LD expressed and purified from mammalian cells, failed to serve as a substrate for FURIN *in vitro* (Figure 1—figure supplement 4D), suggesting instead that FURIN’s role in regulating ATF6α signalling is indirect.

### Calreticulin and an interconnected gene network repress ATF6α

To identify genes that basally repress ATF6α, a similar MAGeCK-based data analysis was applied to the targeted genes enriched in cells that selectively activated the ATF6α reporter under basal conditions. GO analysis of the 100 most significantly enriched genes established an interconnected network highlighting signalling processes including the calreticulin (CRT)/calnexin (CNX) cycle, *N*-glycan biosynthesis, ERAD and COPI-dependent Golgi-to-ER retrograde traffic (Figure 1D and Figure 1—figure supplement 3C). Among these, the top 10 genes included CRT, components of the dolichol-phosphate mannose (DPM) synthase complex, such as *DPM3*, *DPM2*, and *DPM1* involved in the *N*-glycosylation process, and ERAD components, such as *UBE2G2* and *SEL1L* (Figure 1D).

**Figure 2.**
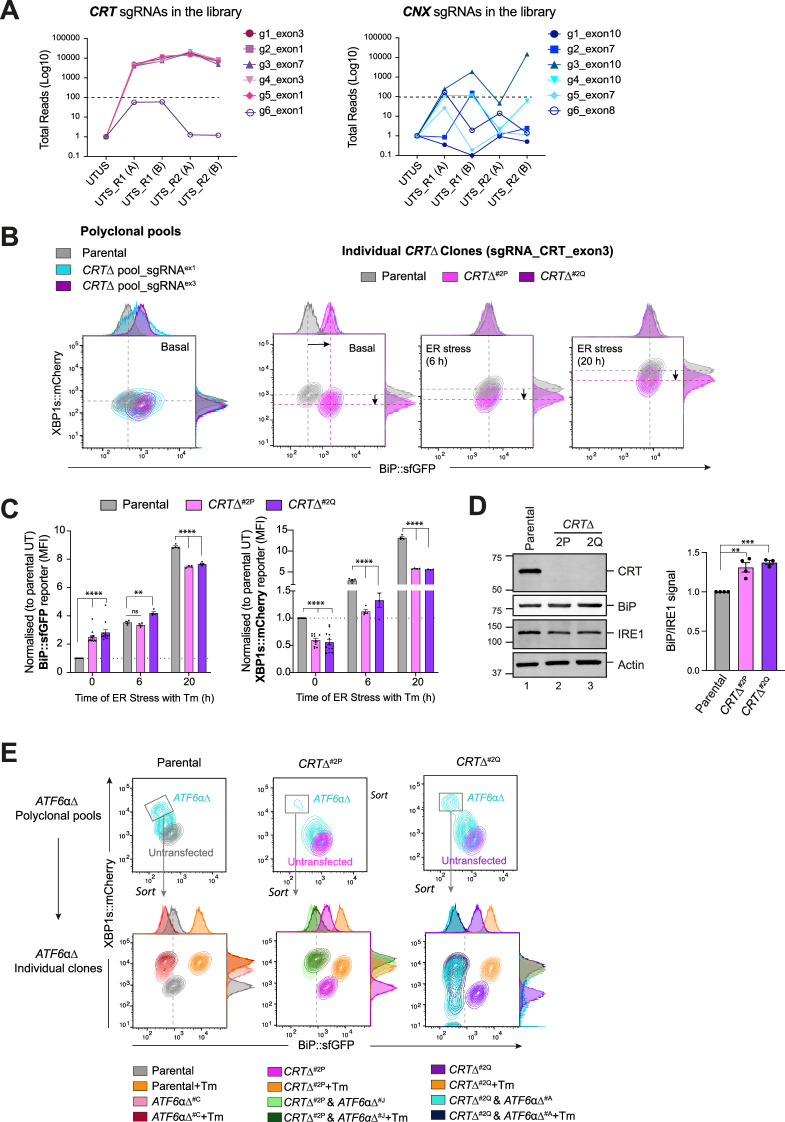
Calreticulin (CRT)-depleted cells exhibit constitutive activation of ATF6α signalling. (**A**) Total read count enrichment through the selection process for each active sgRNA targeting CRT and calnexin (CNX) [UTUS: untreated and unsorted; UTS: untreated and sorted; R1: round 1 of enrichment; R2: round 2 of enrichment; A–C: pools of cells; g1–g6: sgRNAs]. (**B**) Two-dimensional contour plots of BiP::sfGFP and XBP1s::mCherry signals examined in two derivative *CRT*Δ polyclonal pools (left) and two independent *CRT*Δ clones (right, named *CRT*Δ^#2P^ and *CRT*Δ^#2Q^) under basal conditions or endoplasmic reticulum (ER) stress induced with 2.5 µg/ml Tm for a short (6 hr) and extended period of time (20 hr). A representative dataset from more than four independent experiments is shown. (**C**) Normalised quantification expressed as fold-change of the median fluorescence intensity (MFI) of BiP::sfGFP (left) and XBP1s::mCherry signals (right) in the two independent *CRT*Δ clones from more than four independent experiments, as described in ‘B’, indicated by mean ± standard error of the mean (SEM). (**D**) Representative immunoblots of endogenous CRT and BiP protein levels in cell lysates from *XC45-6S* parental cells and two *CRT*Δ derivatives clones selected for functional experiments. The samples were also blotted for IRE1 and actin (loading controls). The right graph bar shows the ratio of BiP to IRE1 signal in four independent experiments indicated by mean ± SEM. (**E**) *Top panel*: Two-dimensional contour plots of BiP::sfGFP and XBP1s::mCherry signals in polyclonal pools of parental cells and two *CRT*Δ clones after depleting the endogenous *ATF6*α locus by CRISPR/Cas9 gene editing. *Lower panel*: Grey rectangles in top panels indicate the subcellular polyclonal population re-sorted to analyse single *ATF6*αΔ clones in parental and *CRT*Δ clones. ER stress treatments with 2.5 µg/ml Tm lasted 20 hr. All statistical analysis was performed by two-sided unpaired Welch’s *t*-test and significance is indicated by asterisks (**p < 0.01; ***p < 0.001; ****p < 0.0001). Figure 2—source data 1.Raw images for gels shown in Figure 2.

Because these four pathways potentially activate all three branches of the UPR, genes previously identified in genome-wide screens to modulate IRE1 or PERK functionality were excluded from further analysis (e.g. *TMED10*, *SEL1L*, *FAB2*, *SYVN1*, and *PDIA6*) (Figure 1H; Adamson et al., 2016; Panganiban et al., 2019; Yang et al., 2018). Of the remaining genes, we focussed on those encoding proteins localised in the ER lumen or Golgi, which could act as specific proximal regulators of ATF6α. Among these, CRT, an abundant luminal ER lectin chaperone, caught our attention (Figure 1H). Notably, although CRT promotes the folding of ER synthesised glycoproteins *via* the CRT/CNX cycle (Lamriben et al., 2016), its ER transmembrane homologue CNX was not identified in the screen. This observation was further supported by the fact that sgRNAs targeting *CNX* were not enriched, whereas five out of six sgRNAs targeting *CRT* showed significant enrichment during the selection process (Figure 2A).

**Figure 2—figure supplement 1.**
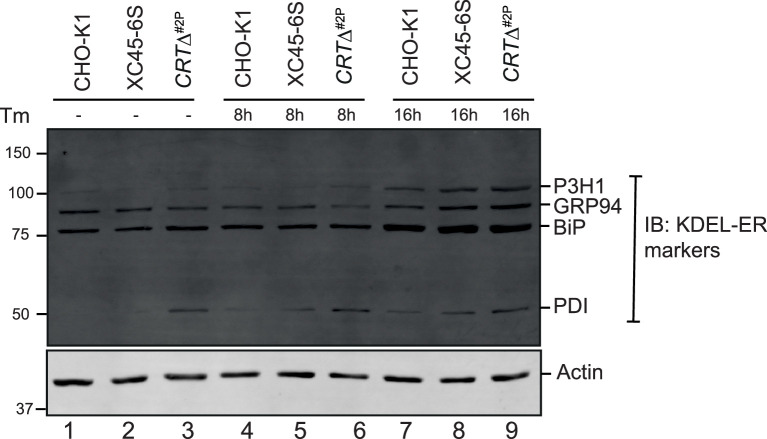
ER-resident proteins (with a KDEL-COOH tag) in calreticulin (CRT)-depleted cells. Representative immunoblots from two independent experiments show the expression levels of endogenous KDEL (Lys-Asp-Glu-Leu) receptors in cell lysates from plain CHO-K1 cells, *XC45-6S* dual unfolded protein response (UPR) reporter cells and a derivative *CRT*Δ clone (2P). The samples were also blotted for actin as a loading control. Figure 2—figure supplement 1—source data 1.Raw images for gels shown in Figure 2—figure supplement 1.

**Figure 2—figure supplement 2.**
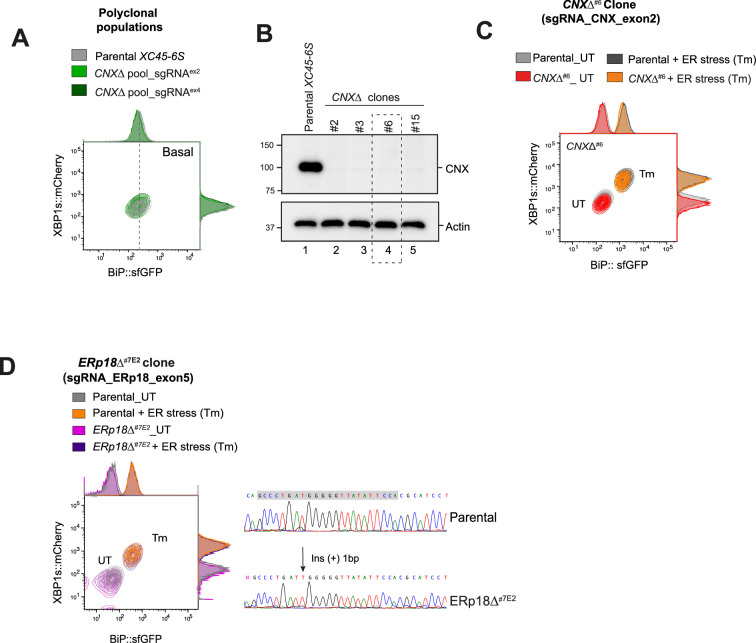
Calnexin and ERp18 depletion do not deregulate ATF6α pathway. (**A**) Two-dimensional contour plots of BiP::sfGFP and XBP1s::mCherry signals in basal conditions in sorted pooled populations where calnexin (CNX) was depleted by CRISPR/Cas9 sgRNAs targeting exons 2 and 4 (in green) and in parental *XC45-6S* cells. (**B**) Immunoblot depicting CNX protein levels in whole-cell lysates from parental cells and four *CNX*∆ derivatives clones. The *CNX∆*^#6^ clone (highlighted into a rectangle dashed box) was selected for subsequent functional experiments in ‘C’. Actin was used as a loading control marker. (**C**) Contour plot, as in ‘A’, from parental cells and the *CNX∆*^#6^ single clone treated with Tm for 20 hr (black and orange) or left untreated (grey and red). (**D**) *Left panel*: Contour plot, as in ‘C’, from parental cells and the *ERp18∆*^#7E2^ single clone treated with Tm for 20 hr (orange and purple) or left untreated (grey and pink). *Right panel*: DNA sequence chromatograms of ERp18-targeted region, in parental cells and an *ERp18*∆ clone with the arrow indicating the peak where the ERp18 indel mutation occurs. sgRNA Cas9 sequence is indicated with a grey rectangle. Figure 2—figure supplement 2—source data 1.Raw images for gels shown in Figure 2—figure supplement 2.

### Constitutive activation of ATF6α in cells lacking CRT

To explore *CRT*’s role in ATF6α regulation, *CRT* was targeted by CRISPR/Cas9 in WT *XC45-6S* dual UPR reporter cells. *CRT*-targeted cells exhibited large populations of BiP::sfGFP^high^; XBP1s::mCherry^low^ cells, mirroring the results of the initial screen. This phenotype was stable in single clones from the targeted pool (Figure 2B, C). Targeting CRT repressed the IRE1 reporter basally and left little room for further induction of the ATF6α reporter upon ER stress (Figure 2B, C). Derepression of the ATF6α reporter in *CRT*∆ clones correlated with a slight increase in BiP protein levels (Figure 2D). Immunoblots of ER-resident proteins (reactive with antibody directed to their endogenous KDEL C-terminal tag) confirmed the increase in BiP protein levels in *CRT*∆ cells and revealed no marked changes in GRP94 protein levels, a slight increase in P3H1 levels, and a notably increase in a band likely corresponding to PDIA in a *CRT*∆ clone (Figure 2—figure supplement 1). In contrast, depletion of CNX had no impact on the BiP::sfGFP reporter (Figure 2—figure supplement 2A and Figure 2—figure supplement 2C), consistent with the findings of the screen.

To test whether derepression of the BiP::sfGFP reporter in *CRT∆* cells was ATF6α dependent, endogenous *ATF6*α was also inactivated in *CRT*∆ cells. ATF6α inactivation restored BiP::sfGFP reporter to its low baseline levels and derepressed the IRE1 reporter to levels observed in *XC45-6S* parental cells (Figure 2E). Overall, these findings confirmed that BiP::sfGFP reporter activity correlates with the transcriptional activity of ATF6α in *CRT*∆ cells.

### CRT represses Golgi-trafficking and processing of ATF6α

**Figure 3.**
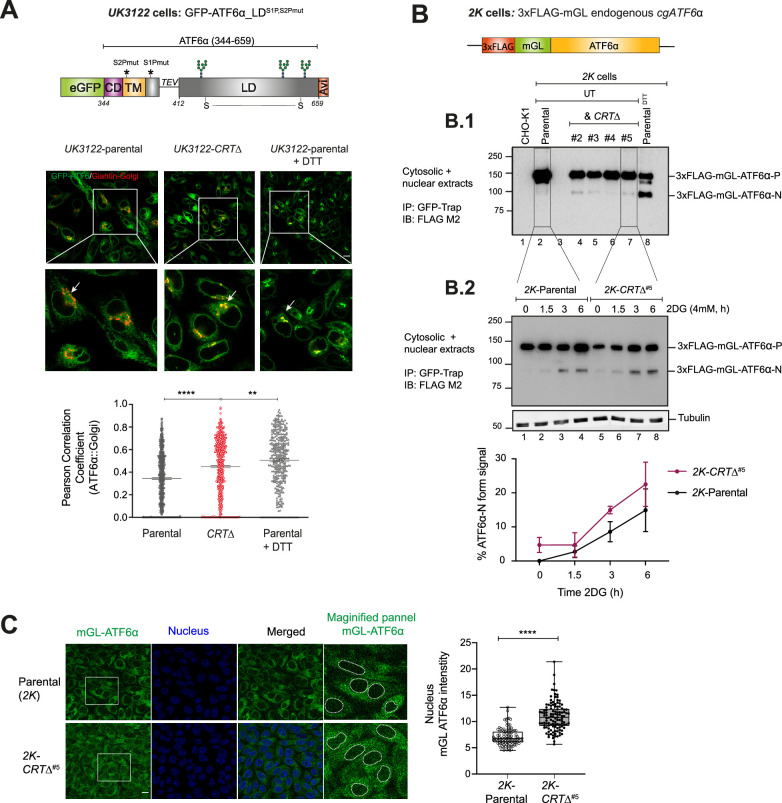
Loss of calreticulin promotes higher proportion of ATF6α trafficking to Golgi and nucleus. (**A**) *Top panel*: Schematic representation of the genetic construct (UK3122) designed for generating a CHO-K1 stable cell line expressing a N-terminus-tagged ATF6α, where eGFP replaces most of the cytosolic domain of *cg*ATF6α, and the S2P and S1P cleavage sites are mutated to increase the intact fraction in the Golgi. Colour code: eGFP tag in green; cytosolic domain (CD) in purple; transmembrane domain (TM) in yellow; Tobacco Etch Virus (TEV) protease cleavage site (ENLYFQ↓G) is represented with a linker line; luminal domain (LD) in grey; and a C-terminus Avi-tag in red. *Lower panels*: Representative live cell confocal microscopy images of *UK3122* CHO-K1 cells stably expressing GFP-ATF6α_LD^S1P,S2Pmut^ and transiently expressing a pmScarlet _Giantin-C1 plasmid as a red fluorescent Golgi marker. Both parental cells and a CRISPR/Cas9 gene edited *CRT∆* derivative pool were imaged. Dithiothreitol (DTT) treatment (4 mM, 1 hr) was applied to parental cells to positively influence ATF6α’s Golgi localisation. Each panel’s magnified sections are presented in the insets. Arrows indicate GFP-ATF6α_LD^S1P,S2Pmut^::Golgi co-localisation. Scale bar: 10μm. Pearson coefficients for the co-localisation of GFP-ATF6α_LD^S1P,S2Pmut^ with the Golgi apparatus marker Giantin in parental cells (*n* > 150), parental cells treated with 4 mM DTT for 1 hr (*n* > 100) and *CRT*∆ cells (*n* > 150) are presented in the bar graph, including cells from three independent experiments. Volocity software was used for co-localisation quantification. Statistical analysis was performed by a two-sided unpaired Welch’s *t*-test and significance is indicated by asterisks (**p < 0.01; ****p < 0.0001). (**B**) Schematic representation of engineered stable *2K* cells with the endogenous *cgATF6*α locus (in orange) tagged with 3xFLAG (in red) and mGreenLantern (mGL) (in green) at the N-terminus. (**B.1**) Cell lysates from *2K* cells and four independent *2K-CRT∆* derivative clones were harvested, immunoprecipitated (IP) using GFP-Trap Agarose (ChromoTek) and analysed by immunoblot (IB) using an anti-FLAG M2 antibody to detect processing of ATF6α. Full-length ATF6α-Precursor (ATF6α-P) and processed ATF6α-N forms were identified. Treatment with DTT for 1 hr (4 mM) was used to induce ATF6α processing. Parental untagged cells were used as control for background. Data shown are representative of one experiment. (**B.2**) Parental *2K* cells and a derivative single *CRT∆* clone (#5) were treated with 4 mM 2-deoxy-D-glucose (2DG) at the indicated time points (1.5, 3, and 6 hr). Cells were harvested and analysed by immunoblot as in ‘B.1’. Lower panel shows the percentage of ATF6α-N form signal/total ATF6α signal from three independent experiments indicated by mean ± standard error of the mean (SEM). (**C**) ATF6α nuclear translocation live cell microscopy assay showing unstressed *2K* parental cells and a *2K-CRT∆* clone. mGL-ATF6α is in green, and nucleus in blue. mGL-ATF6α signal intensity in the nucleus (shown by dashed lines) was measured using Volocity software. Scale bar: 10μm. Data from parental cells (*n* > 100) and *CRT*∆ cells (*n* > 100) are displayed in a box and whiskers graph, displaying all points (with min. and max. intensities). Figure 3—source data 1.Raw images for gels shown in Figure 3.

Considering the crucial role of trafficking in ATF6α activation, we next investigated whether depletion of CRT affected the stress-induced localisation of ATF6α by comparing the subcellular localisation of ATF6α in parental and *CRT*∆ cells. As trafficking is closely linked to proteolytic processing, WT ATF6α does not accumulate in the Golgi of stressed cells (Shen et al., 2002). To stabilise ATF6α molecules reaching the Golgi, a CHO-K1 cell line stably expressing a GFP-tagged version of *cg*ATF6α_LD lacking S1P and S2P cleavage sites (GFP-ATF6α_LD^S1P,S2Pmut^) was engineered (Figure 3A). In non-stressed cells, GFPATF6α_LD^S1P,S2Pmut^ was distributed in a prominent reticular ER pattern with only a minor fraction co-localising with the Scarlet-tagged Giantin Golgi localisation marker. Exposure to the rapidly acting ER stress-inducing agent dithiothreitol (DTT), increased the Golgi pool at the expense of the ER pool (Figure 3A); an expected outcome validating GFP-ATF6α_LD^S1P,S2Pmut^ as a sentinel for the ATF6α protein. Compared to parental cells, GFP-ATF6α_LD^S1P,S2Pmut^ showed significantly more Golgi co-localisation in *CRT∆* cells under basal conditions (Figure 3A). These results were in line with our previous flow cytometry observations and suggested that CRT contributes to ER retention of ATF6α, either directly or indirectly.

**Figure 3—figure supplement 1.**
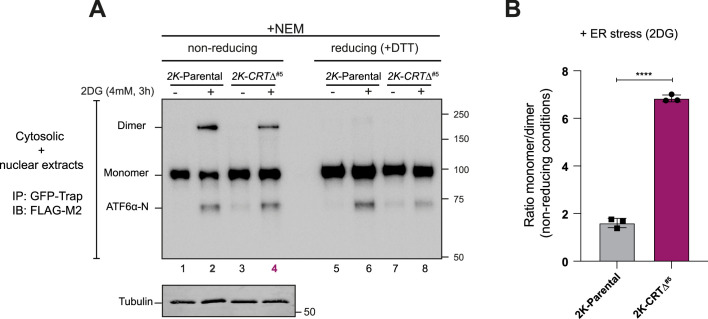
Analyses of ATF6α redox status in calreticulin (CRT)-depleted cells. (**A**) Whole-cell lysates from stable *2K* cells with the endogenous *cgATF6*α locus tagged with 3xFLAG and mGreenLantern (mGL) and a derivative *2K-CRT∆* clone (#5) were separated under non-reducing (-DTT) and reducing (+50 mM DTT) sodium dodecyl sulfate/polyacrylamide gel electrophoresis (SDS/PAGE) conditions. ATF6α redox status was analysed under basal conditions and following 2-deoxy-D-glucose (2DG) treatment (4 mM, 3 hr) to induce endoplasmic reticulum (ER) stress and ATF6α trafficking. Samples were harvested and lysed in the presence of 20 mM N-ethylmaleimide (NEM), immunoprecipitated (IP) using GFP-Trap Agarose (ChromoTek) and analysed by immunoblot (IB) using an anti-FLAG M2 antibody to detect ATF6α. Shown is a representative immunoblot from three independent repetitions. (**B**) The graph bar shows the ratio of ATF6αmonomer to ATF6αdimer signals in *2K* cells (line 2 from ‘A’) and *2K-CRT∆* clone (line 4 from ‘A’) under stress conditions in non-reducing conditions from three independent experiments indicated as mean ± standard error of the mean (SEM). Statistical analysis was performed by a two-sided unpaired Welch’s *t*-test and significance is indicated by asterisks (****p < 0.0001). Figure 3—figure supplement 1—source code 1.Raw images for gels shown in Figure 3—figure supplement 1.

GFP-ATF6α_LD^S1P,S2Pmut^ reporter cannot be proteolytically processed. Consequently, to gauge the effect of CRT on ATF6α processing we sought to create a CHO-K1 stable cell line to track endogenous ATF6α. For that, the *cgATF6*α locus was tagged with a 3xFLAG and a monomeric GreenLantern (mGL) fluorescent protein, generating a cell line referred to as *2K*: 3xFLAG-mGL-ATF6α (Figure 3B). The functionality of this probe was confirmed by treatment with DTT that resulted in the appearance of a prominent faster migration band in sodium dodecyl sulfate–polyacrylamide gel electrophoresis (SDS–PAGE), compatible with the processed S2P-cleaved ATF6α (N-ATF6α) form, and depletion of the precursor form (ATF6α-P) (Figure 3B.1, lane 8). Subsequently, *2K* cells were targeted to generate *2K-CRT*∆ derivatives cells, enabling a comparison of ATF6α processing. *2K-CRT*∆ clonal cells exhibited elevated levels of the processed N-ATF6α form under both basal and stress-induced conditions (Figure 3B.1,B.2) that correlated with a higher nuclear mGL-ATF6α signal observed by live cell microscopy (Figure 3C). These findings indicated that CRT represses both ER-to-Golgi trafficking and processing of ATF6α into its active form.

It has been reported that ATF6α can exist in three redox forms: a monomer and two inter-chain disulfide-stabilised dimers (Nadanaka et al., 2007). To investigate whether CRT depletion alters the redox status of ATF6α, cell lysates from *2K*-parental cells and *2K-CRT*∆ clonal cells were analysed under both basal and ER-stress conditions using non-reducing SDS–PAGE (Figure 3—figure supplement 1A). We detected two redox forms of ATF6α, consistent with an inter-chain disulfide-stabilised dimer and a monomer. Under basal conditions, ATF6α predominantly existed as a high mobility monomer in both *2K*-parental and *2K-CRT*∆ cells, with the appearance of an even faster-migrating processed N-ATF6α form in *2K-CRT*∆ cells, consistent with the findings presented in Figure 3B. ER stress (induced by 2DG) was associated with appearance of a low-mobility band consistent with a disulfide-stabilised dimer, and a concomitant decrease in the ATF6α monomer intensity in *2K*-parental cells, as previously reported (Oka et al., 2022). Interestingly, under stress conditions, the monomer levels in *2K-CRT*∆ cells remained largely unchanged, indicating a higher monomer-to-dimer ratio compared to parental cells (Figure 3—figure supplement 1B). These data suggest that the loss of CRT may stabilise the monomeric form of ATF6α, which is proposed to be more efficiently trafficked. This observation aligns with our results showing that CRT depletion is linked to activation of ATF6α.

### CRT interacts with the ATF6α LD *in vitro*

**Figure 4.**
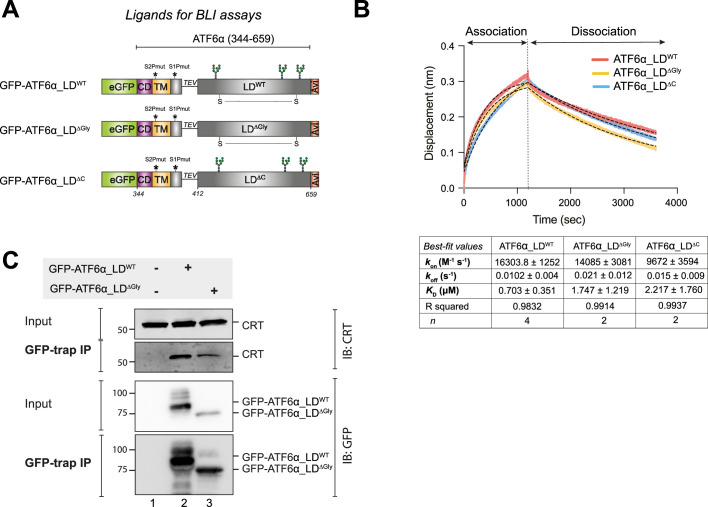
Calreticulin (CRT) interacts directly with the ATF6α luminal domain (LD) *in vitro* and in cells. (**A**) Schematic representation of the constructs designed to produce three LDs of ATF6α to be used as ligands in biolayer interferometry (BLI) assays: wild-type (WT) ATF6α_LD, non-glycosylated (∆Gly) ATF6α_LD, and ATF6α_LD lacking its cysteines (∆C). The probes underwent *in vivo* biotinylation, purification using GFP-Trap Agarose (ChromoTek), and were eluted via TEV cleavage. Colour code: eGFP tag in green; cytosolic domain (CD) in purple; transmembrane domain (TM) in yellow; TEV cleavage site with a linker line; LD in grey; C-terminus Avi-tag in red. S–S indicates for the disulphide bond in the LD between two cysteines. (**B**) Representative BLI signals depicting the interaction of rat (r) CRT (analyte) with different forms of *cg*ATF6α_LD (from HEK293T cells) immobilised on streptavidin biosensors: ATF6α_LD^WT^ in red, ATF6α_LD^∆Gly^ in yellow, and ATF6α_LD^∆C^ in blue. The interaction kinetics were calculated over the entire 1200s of the association phase and 2400s of the dissociation phase. Dotted lines represent the best-fit association then dissociation curves using a nonlinear regression to calculate kinetic constants by Prism10. The table represents the BLI measurements of the kinetics of the interaction between rCRT and *cg*ATF6α_LD from two to four independent experiments (means ± standard error of the mean [SEM]). Interaction kinetics were measured at an rCRT concentration of 10 µM. (**C**) Representative immunoblots (IB) of endogenous CRT recovered in complex with ATF6α in HEK293T cells overexpressing GFP-*cg*ATF6α_LD^WT^, GFP-*cg*ATF6α_LD^∆Gly^, or neither. Immunoprecipitations (IP) were performed using GFP-Trap Agarose, and then membranes were blotted with anti-human CRT and anti-GFP antibodies. The immunoblots are representative for three independent experiments. Figure 4—source data 1.Raw images for gels shown in Figure 4.

To investigate the underlying mechanism of CRT’s repressive effect on ATF6α, we explored the physical interaction between the two purified proteins *in vitro* using BioLayer Interferometry (BLI). The biotinylated ATF6α_LD, expressed in mammalian cells, was immobilised on the BLI probe and used as the ligand, and bacterially expressed CRT was used as the analyte. Considering possible regulatory roles of *N*-glycosylation and disulphide bonds in ATF6α activation (Hong et al., 2004; Oka et al., 2019), three different versions of ATF6α_LD were used: (1) fully glycosylated ATF6α_LD (WT), (2) non-glycosylated ATF6α_LD (∆Gly), and (3) ATF6α_LD lacking its cysteines (∆C) (Figure 4A). BLI showed reversible binding of CRT to ATF6α_LD^WT^ that was characterised by slow association (*k*_on_) and dissociation (*k*_off_) rates (Figure 4B). Both the association and dissociation phases were fitted to a single exponential association-dissociation model, yielding a *K*_D_ within the range of 1 µM, a concentration that was consisted with the estimated CRT concentration in the ER of CHO-K1 cells obtained through quantitative immunoblotting (~7 µM) (Figure 4—figure supplement 1). Similar interactions were also observed with ATF6α_LD^∆Gly^ and ATF6α_LD^∆C^.

**Figure 4—figure supplement 1.**
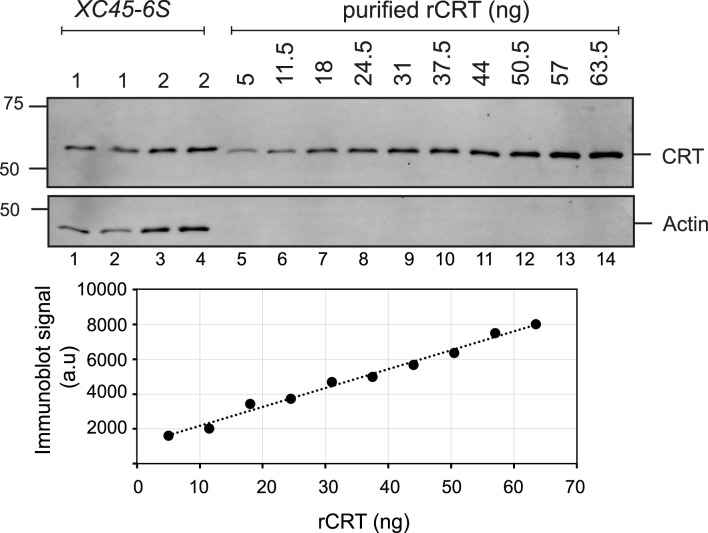
Estimation of calreticulin’s (CRT) physiological abundance by quantitative immunoblotting. *Top panel*: Immunoblot showing CRT and actin levels for parental XC45-6S cells and known amounts of purified bacterially expressed rCRT. *Lower panel*: Calibration curve derived from known quantities of purified rCRT, fitted to a linear function. Assuming the endoplasmic reticulum (ER) constitutes 10% of cell total volume (Alberts et al., 2002), the concentration of CRT in the ER of CHO-K1 cells was estimated to be around 7 µM. Figure 4—figure supplement 1—source data 1.Raw images for gels shown in Figure 4—figure supplement 1.

To examine a possible interaction between CRT and ATF6α in cells, HEK293T cells were transfected with either GFP-ATF6α_LD^WT^ or GFP-ATF6α_LD^∆Gly^, followed by selective recovery using GFP-Trap Agarose and subsequent immunoblotting with an anti-CRT antibody. The co-immunoprecipitation results showed that both ATF6α_LD^WT^ and, to a lesser extent, ATF6α_LD^∆Gly^ immunoprecipitated endogenous CRT (Figure 4C). Re-probing the blot with an anti-GFP antibody revealed that GFP-ATF6_LD^∆Gly^ was expressed at lower levels than ATF6α_LD^WT^ (Figure 4C), potentially accounting for the reduced recovery of CRT in complex with ATF6α_LD^∆Gly^. Overall, these observations suggested that CRT binds to ATF6α_LD both in cells and in isolation *in vitro*, supporting the notion of a direct interaction between the two proteins.

### A genetic platform to study endogenous ATF6α signalling

**Figure 5.**
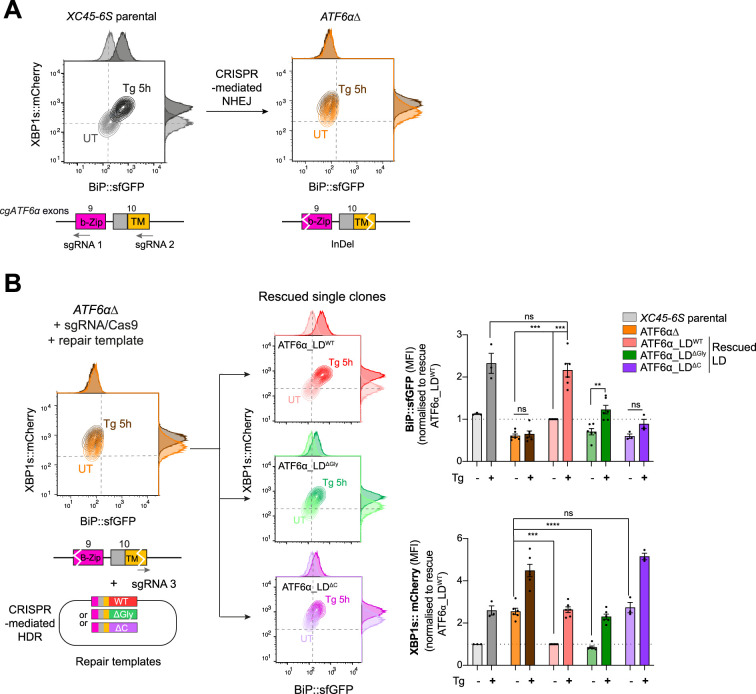
A homologous CRISPR/Cas9 gene editing recombination platform to study ATF6α variants at endogenous levels in CHO-K1 cells. (**A**) Two-dimensional contour plots of BiP::sfGFP and XBP1s::mCherry signals in *XC45-6S* parental cells (in grey; left) and in a null functional ATF6α clonal cell (*ATF6α*∆, in orange; right) generated by CRISPR/Cas9-mediated NHEJ (non-homologous end joining) to introduce frameshifting mutations into the DNA-binding (b-Zip, pink) and transmembrane (TM, yellow) encoding regions by targeting exons 9 and 10 of the endogenous locus of *cgATF6*α. Cells were analysed in basal conditions (UT) or upon endoplasmic reticulum (ER) induction with Tg (0.5 µM, 5 hr). A schema for the sgRNAs/Cas9 that target cgATF6 is shown below the plots. (**B**) *ATF6α*Δ cells were re-targeted with a sgRNA/Cas9 directed to the mutated exon 10 and offering three repair templates: ATF6α_LD^WT^ in red, ATF6α_LD^∆Gly^ in green, and ATF6α_LD^∆C^ in purple. Successfully rescued single clones by CRISPR/Cas9-mediated homology-directed repair (HDR) were evaluated in the absence (pale colour) and presence of ER stress (0.5 µM Tg, 5 hr, dark colour). Contour plots are representative from three to six independent experiments. Bar graphs display the quantification of the fold-change of BiP::sfGFP (top) and XBP1s::mCherry signals (bottom) in more than three independent experiments indicated by mean ± standard error of the mean (SEM). Statistical analysis was performed by a two-sided unpaired Welch’s *t*-test and significance is indicated by asterisks (***p < 0.001; ****p < 0.0001; ns: non-significant).

To circumvent the potentially corrupting effect of protein overexpression, we sought to express ATF6α variants from the endogenous locus, by replacing the ATF6αLD-coding sequence with mutants of our design. To enable CRISPR–Cas9-mediated homology-directed repair (HDR) that replaces the WT sequence with the mutants, we first created a non-functional ATF6α allele that could be subsequently restored to function by homologous recombination, offering WT or mutant repair templates (Figure 5A). As expected, the deletion of ATF6α resulted in the loss of the BiP::sfGFP signal in stressed cells (and enhanced XBP1s::mCherry signal in basal conditions, Figure 5A). This ATF6α∆ clone was re-targeted with a unique sgRNA/Cas9, alongside ATF6α_LD^WT^, ATF6α_LD^∆Gly^, or ATF6α_LD^∆C^ repair templates (Figure 5B).

**Figure 5—figure supplement 1.**
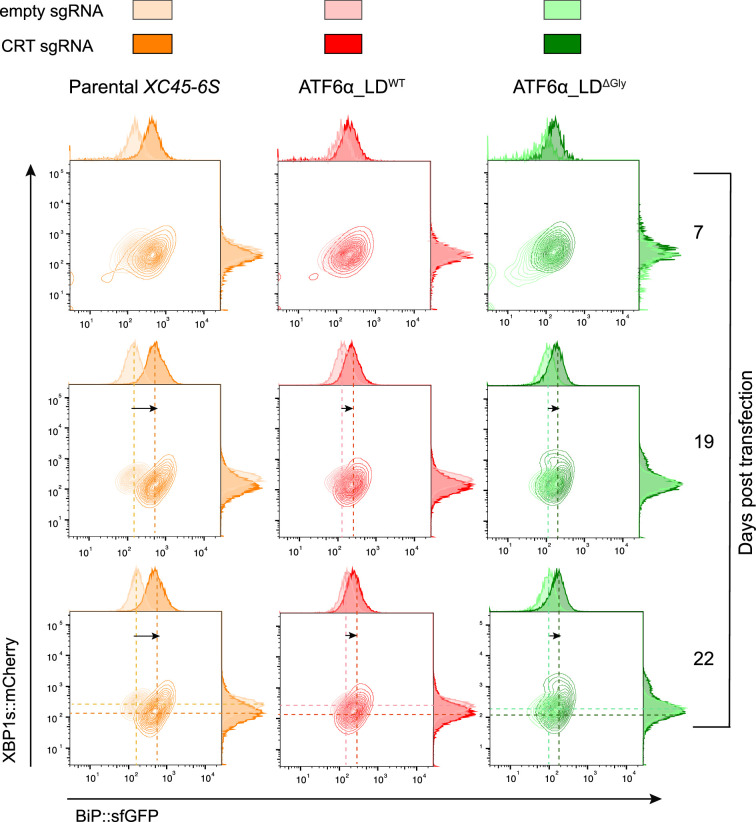
Calreticulin (CRT) depletion in ATF6α_LD^∆Gly^ knock-in cells does not derepress basally ATF6α reporter activity. Contour plots of BiP::sfGFP and XBP1s::mCherry signals from parental cells (orange) and ATF6α_LD^WT^ (in red) or ATF6α_LD^∆Gly^ (in green) rescued cells (generated in Figure 5B) where endogenous CRT was depleted by transient transfection with a sgRNA/Cas9 (Puromycin selection) targeting *cgCRT* locus. Transfected cells were selected in the presence of puromycin (8 μg/ml) and analysed under basal conditions at 7-, 19-, or 22-day post-transfection. Cells were also transfected with an empty sgRNA/Cas9 plasmid (pale colour) as control for the transfection effect. The presented dataset is representative of a single experiment.

Unlike the ATF6α_LD^WT^, neither the ATF6α_LD^∆Gly^ nor the ATF6α_LD^∆C^ repair templates fully restored BiP::sfGFP responsiveness to stress (Figure 5B, top panel), consistent with the importance of *N*-glycosylation and disulphide bond formation to ATF6α functionality. Notably, whereas ATF6α_LD^∆C^ knock-in cells exhibited basal activation of the IRE1 reporter comparable to levels observed in the ATF6α∆ parent (Figure 5B, lower panel), the IRE1 reporter signal in the ATF6α_LD^∆Gly^ knock-in cells shifted to that observed in WT cells, suggesting that ∆Gly allele retained some functionality. The partial ATF6α functionality of the ATF6α_LD^∆Gly^ knock-in allele aligned with the lower expression levels of ATF6α_LD^∆Gly^ compared to ATF6αLD^WT^ in transfected cells, as previously observed in Figure 4C. However, this feature complicated interpretation of experiments designed to establishing the role of ATF6α_LD glycans on CRT-mediated repression of ATF6α: failure of CRT deletion to derepress the BiP::sfGFP signal in ATF6α_LD^∆Gly^ knock-in cells (Figure 5—figure supplement 1) could be interpreted as either an obligatory role for glycans in the repression or as lack of dynamic range of the signal needed to detect a residual effect of CRT deletion in ATF6α_LD^∆Gly^ knock-in cells.

### Impact of CRT variants on ATF6α reporter activity

**Figure 6.**
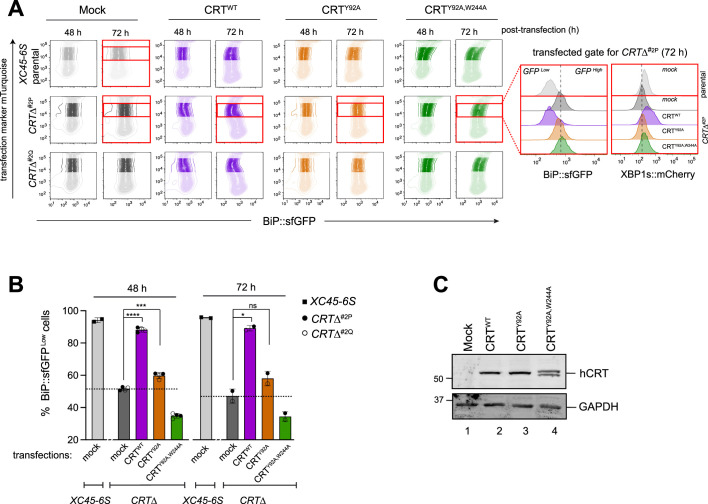
Impact of calreticulin (CRT) overexpression on cells depleted of CRT. (**A**) Two-dimensional contour plots of BiP::sfGFP and mTurquoise signals (transfection marker) in parental cells and two derivative *CRT*Δ clones transiently transfected for 48 and 72 hr with plasmids encoding wild-type human CRT (CRT^WT^) or two lectin CRT mutants, CRT^Y92A^ and CRT^Y92A,W244A^. As a control for transfection, cells were transfected with a mock plasmid. Shadowed rectangles mark cells that were not selected for analysis because they had low levels of mTurquoise-tagged plasmid (indicating low transfection) or have very high transfection levels that could be toxic. Cells within unshadowed rectangles and marked with red delineate those cells expressing moderate/high levels of mTurquoise-tagged plasmid selected for analysis of BiP::sfGFP and XBP1s::mCherry signals distribution. Right histograms indicate the transfected, gated cells 72 hr post-transfection (red rectangles). (**B**) Bar graph displaying the percentage of BiP::sfGFP^low^ cells (rescue phenotype) in mTurquoise-positive cells gated by the unshadowed boxes in ‘A’ as mean ± standard deviation (SD) of data obtained from one (72 hr) to two (48 hr) independent experiments. Data from both *CRT*Δ clones have been combined in each time point of analysis. Statistical analysis was performed by a two-sided unpaired Welch’s *t*-test and significance is indicated by asterisks (*p < 0.05; ***p < 0.001; ****p < 0.0001; ns: non-significant). (**C**) CHO-K1 cells were transiently transfected with the hCRT plasmids used in ‘A’ for 72 hr. Following transfection, cell lysates were recovered, and equal protein amounts were loaded into 12.5% sodium dodecyl sulfate–polyacrylamide gel electrophoresis (SDS–PAGE) gels and immunoblotted for hCRT to analyse protein stability in CRT mutants. GAPDH served as a loading control. The immunoblots are representative for two independent experiments. Figure 6—source data 1.Raw images for gels shown in Figure 6.

CRT is a lectin chaperone that interacts with the PDI ERp57 to stabilise CRT–substrate binding (Oliver et al., 1999). Therefore, we tested the ability of human CRT^WT^ and two mutants that compromised glycan binding (Y92A) or Erp57 co-chaperone binding (W244A) (Del Cid et al., 2010) to reverse the *CRT*∆ phenotype. Parental and *CRT∆* clones were transfected with CRT^WT^, CRT^Y92A^, and the double mutant CRT^Y92A,W244A^ expressing plasmids, and cells were evaluated by flow cytometry 48 and 72 hr post-transfection (Figure 6A). Expression of CRT^WT^ restored basal levels of both BiP::sfGFP and XBP1s:mCherry reporters in *CRT*∆ cells (Figure 6A, purple panels and Figure 6B), confirming the repressive role of CRT on ATF6α. In contrast, expression of the double CRT^Y92A,W244A^ mutant failed to rescue the *CRT*∆ phenotype (Figure 6A, green panels and Figure 6B), whereas CRT*^Y92A^*, selectively incapable of glycan binding, retained some ability to repress BiP::sfGFP and modestly rescuing the *CRT∆* phenotype (Figure 6A, orange panels and Figure 6B). Immunoblots targeting CRT raised concerns about the integrity of theCRT^Y92A,W244A^ protein, precluding a separate analysis of Erp57’s role in ATF6α repression (Figure 6C). However, CRT^WT^ and CRT^Y92A^ proteins were expressed similarly (Figure 6C). Together, our observations pointed to an important role for interactions between CRT and ATF6α_LD glycans but also left room for some lectin independent repression of ATF6α by CRT.

### CRT depletion exposes a negative feedback loop by which ATF6α represses IRE1

**Figure 7.**
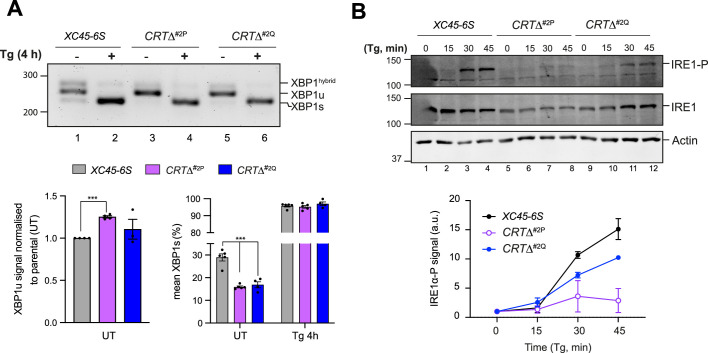
Calreticulin depletion exposes a negative feedback loop of ATF6α activity on IRE1. (**A**) *Top panel*: Agarose gel of XBP1 cDNA from thapsigargin (Tg)-treated parental cells and two *CRT∆* clones. Migration of the unspliced (XBP1u), spliced XBP1 (XBP1s), and hybrid (XBP1^hybrid^) stained DNA fragments is indicated. The XBP1^hybrid^ band represents DNA species containing one strand of XBP1u and one strand of XBPs (Shang and Lehrman, 2004). Splicing was assessed by reverse transcription (RT)-PCR. *Lower panel*: Plots displaying the total XBP1u signal in the left panel, and the fraction of XBP1s in the right panel. For quantification, 50% of the hybrid band signal was assumed to be XBP1s and the other 50% XBP1u. The bars and error bars represent the mean ± standard error of the mean (SEM) of data obtained from more than three independent experiments. Statistical analysis was performed by a two-sided unpaired Welch’s *t*-test and significance is indicated by asterisks (***p < 0.001). (**B**) Representative immunoblots of endogenous levels of cgIRE1 and phosphorylated IRE1 (IRE1-P) in parental cells and two *CRT*Δ clones basally and upon endoplasmic reticulum (ER) stress induction with 0.5 μM Tg for the indicated times. The levels of IRE1-P signal over the time in each genotype are represented in a graph as mean ± standard deviation (SD) of data obtained from two independent experiments. Figure 7—source data 1.Raw images for gels shown in Figure 7.

Given the well-established interplay between the ATF6α and IRE1 branches of the UPR, we sought to assess whether the constitutive activation of ATF6α observed in *CRT∆* clones could be a contributing factor to the conspicuous downregulation of IRE1 activity. Through reverse transcription (RT)-Polymerase Chain Reaction (PCR) and gel electrophoresis, IRE1-mediated XBP1 mRNA splicing in parental cells and *CRT∆* clones were compared. Under basal conditions, unspliced mRNA levels (XBP1u) were significantly higher in *CRT∆* clones (Figure 7A, left bar graph), consistent with the role of NATF6α in the transcriptional activation of XBP1 (Yoshida et al., 2001). In addition, compared to parental cells, *CRT∆* cells exhibited a smaller fraction of spliced XBP1 mRNA (XBP1s) (Figure 7A, right bar graph), consistent with the lower IRE1 activity measured by a XBP1s::mCherry reporter.

IRE1 activity is closely tied to its phosphorylation state (Shamu and Walter, 1996), therefore IRE1α-phosphorylation (IRE1α-P) levels were compared in parental and *CRT*∆ clones. Accordingly, *CRT*∆ cells exhibited lower levels of stress-induced phosphorylation of their endogenous IRE1 protein and slightly reduced total IRE1 levels (Figure 7B). These results supported earlier findings that IRE1 signalling is repressed by ATF6α activity (Walter et al., 2018). However, the defect in IRE1 activity in CRT-depleted cells was partial, as in the presence of ER stress, all genotypes exhibited a comparable increase in XBP1s (Figure 7A).

## Discussion

This study reports on a comprehensive and unbiased genome-wide CRISPR/Cas9-based knockout screen dedicated to discovering regulators of ATF6α. Whereas previous high-throughput studies have broadly studied modulators of the UPR (Adamson et al., 2016; Jonikas et al., 2009; Panganiban et al., 2019), we designed an ATF6α/IRE1 dual UPR reporter cell line to selectively detect regulators of ATF6α. Although a connection between CRT and ATF6α was proposed previously based on an interaction between overexpressed proteins (Hong et al., 2004), our study utilises unbiased genetic tools and manipulation of endogenous proteins to establish CRT’s role as a selective repressor of endogenous ATF6α signalling, mapping its function in retaining ATF6α in the ER.

It is notable that, though the screen identified well-known regulators of ATF6α processing (including S1P, S2P, and NF-Y, and thus validating the experimental approach), only a limited number of new genes that contribute to ATF6α signalling emerged from the screen. Despite the biochemical evidence for the role of COPII-coated vesicles and the oligomeric state of ATF6α in its activation, no cluster of genes involved in COPII vesicular transport or PDIs were found among the most enriched pathways. Nor were any counterparts to SCAP or INSIG, regulators of ER-to-Golgi trafficking of the related SREBP proteins (Yang et al., 2002), identified. This might reflect essentiality of the genes involved or redundancy amongst them. Cell-type specificity may also be implicated in failure to identify components that are unimportant to ATF6α activation in CHO-K1 cells. For example, deletion of ERp18, a small PDI-like protein implicated in ATF6α activation in HEK cells (Oka et al., 2019), affected neither the ATF6α nor IRE1α UPR branches in CHO-K1 cells studied here (Figure 2—figure supplement 2D).

The screen implicated two components of the nuclear pore complex (*NUP50* and *SEH1L*) in ATF6α pathway activity. While it might be tempting to consider a role for these components in the nuclear translocation of the processed soluble ATF6α N-terminal domain, there is no reason to think it would be regulatory, as it operates downstream of ER stress-regulated trafficking and processing events.

In addition to the identified S1P and S2P proteases, the *screen for ATF6*α *activators* identified FURIN, a Golgi-localised protease belonging to the subtilisin-like proprotein convertase family (like S1P protease) responsible for proteolytic activation of a wide array of precursor proteins within the secretory pathway (Braun and Sauter, 2019). FURIN deletion synergised with S1P inhibitors in attenuating ATF6α signalling. Additionally, ATF6α_LD has a conserved region (RTKSRR) related to the FURIN cleavage motif generally described as RX-R-X-[K/R]-R↓ (Nakayama, 1997; Figure 1—figure supplement 4E). It has been suggested that S1P’s role in activation is to reduce the size of ATF6α’s_LD, thereby promoting S2P cleavage (Shen et al., 2002; Ye et al., 2000). FURIN cleavage could contribute to such a process and account for partial redundancy between S1P inhibition and FURIN depletion observed here. Nonetheless, *in vitro* FURIN failed to cleave the ATF6α_LD. These findings collectively suggest that FURIN may function as an indirect enhancer of ATF6α signalling, perhaps by setting up conditions in a post-ER compartment that favours ATF6α cleavage and activation.

The *screen for ATF6*α *repressors* revealed a substantial enrichment of genes involved in glycoprotein metabolism. Among that cluster, the most notable hit, not previously identified in other high-throughput UPR screens, was CRT, a well-characterised soluble ER chaperone with a key role in protein quality control. Intriguingly, its ER-membrane counterpart, CNX, did not emerge in our screen, despite the inclusion of six sgRNA targeting CNX in the CRISPR library. This suggests a special role of CRT in the regulation of ATF6α, at least in CHO-K1 cells. CRT may not be the only repressor of ATF6α; this role may be shared by BiP, which has been widely suggested to suppress all the three branches of the UPR under basal conditions. Nevertheless, because of BiP’s pervasive effects on ER proteostasis and its role in direct repression of the IRE1 branch, its role in regulating ATF6 might have been missed by this screen.

Disruption of CRT in the *XC45-6S* dual ATF6α/IRE1 UPR reporter cell line selectively activated the ATF6α pathway reporter under basal conditions. This activation was accompanied by an increased baseline trafficking of ATF6α from the ER to the Golgi, enhanced processing of ATF6α to its active form and increased endogenous BiP protein levels. Loss of CRT also resulted in a conspicuous basal downregulation of the XBP1s::mCherry reporter that was associated with a reduced activity of IRE1 in *CRT∆* cells. These findings align with previous evidence that N-ATF6α suppresses IRE1 signalling (Walter et al., 2018), and highlights the importance of ATF6α/IRE1 crosstalk.

CRT engages monoglycosylated glycoproteins in the ER via its glycan-binding lectin domain, retaining them in the CRT/CNX cycle (Hebert et al., 1996; Ou et al., 1993). A role for CRT’s interaction with ATF6α_LD glycans in repressing ATF6α is suggested by the markedly diminished ability of a lectin mutant CRT^Y92A^ to repress the ATF6α pathway compared to the WT. However, it is worth noting that CRT has been observed to bind proteins also in a glycan-independent manner (Wijeyesakere et al., 2013). Both BLI data and immunoprecipitation from cell extracts revealed that CRT can associate with both WT and unglycosylated ATF6α (∆Gly). In the former, direct interaction is characterised by slow kinetics, consistent with previous measurements of glycosylation-independent binding of CRT to its clients (Table 1 from Wijeyesakere et al., 2013). These biophysical features, coupled with the residual ability of the lectin mutant CRT^Y92A^ to repress endogenous ATF6α, lead us to suggest that CRT repression of ATF6α may involve both glycosylation-dependent and -independent interactions.

**Figure 8.**
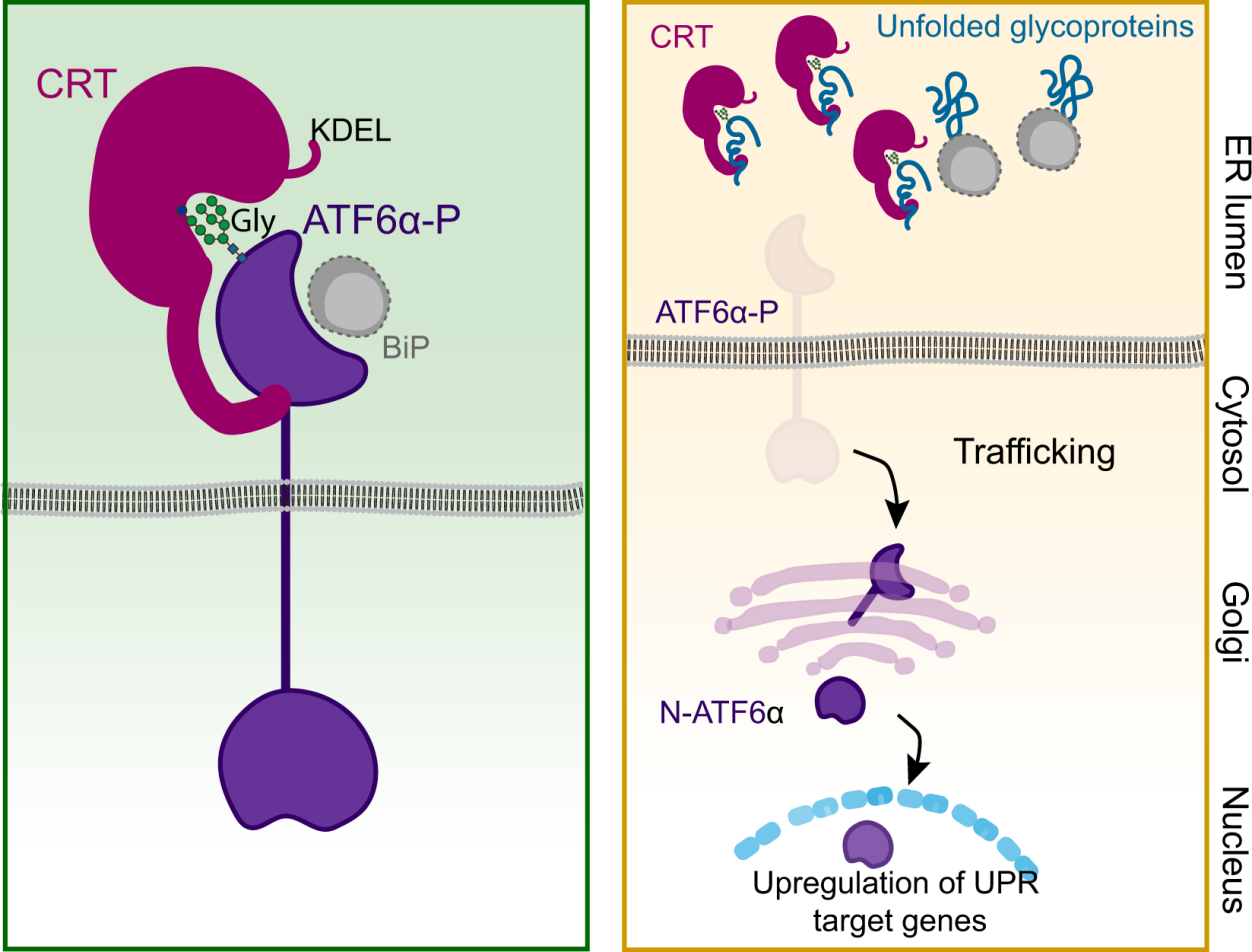
Proposed regulatory model of ATF6α by calreticulin (CRT) proteostasis. *Left panel*: When concentrations of competing ligands (unfolded glycoproteins) are low, endoplasmic reticulum (ER) retention of the ATF6α–CRT complex is favoured (via CRT’s KDEL signal). The ATF6α–CRT interaction is cartooned here as having both glycan (Gly)-dependent and -independent components. *Right panel*: Unfolded glycoproteins compete with ATF6α for CRT and their increasing concentration favours ATF6α trafficking to the Golgi in stressed cells. A similar mechanism, involving BiP and shared by other unfolded protein response (UPR) transducers, has been previously proposed (Bertolotti et al., 2000) and is included here as a complement to ATF6α-specific regulation by CRT. ATF6α-P refers to the precursor form of ATF6α, while N-ATF6α denotes the active form of ATF6α.

Currently, the structural basis for ATF6α_LD interactions with CRT remains unknown, and its role in the direct or indirect retention of ATF6α in the ER is a subject for speculation. Nonetheless, the observations presented here support previous studies indicating that the stable interaction of CRT with cellular substrates combines both lectin-dependent and -independent interactions into a hybrid mode. Moreover, our observations raise the possibility that the lectin activity of CRT is dispensable for its interaction with ATF6α_LD but implicated in recruitment of a third component required for repression. Either way titration of CRT by competing ligands is a plausible regulatory mechanism coupling ATF6α activity to the burden of unfolded (glycol) proteins in the ER (Figure 8).

## Materials and methods

**Key resources table:** 

Reagent type (species) or resource	Designation	Source or reference	Identifiers	Additional information
Recombinant DNA reagent	pRK793 His6_TEV(S219V)_Arg	Gift from Wang Lab	UK759	His-TEV(S219V)-Arg from pRK793
Recombinant DNA reagent	pSpCas9(BB)-2A-Puro	Gift from Richard Timms	UK1367	Puro-tagged CRISPR introduces double strand breaks (Addgene 48139) (Zhang PX459)
Recombinant DNA reagent	Lenti-Cas9-Blast	Harding et al., 2019	UK1674	Lenti-Cas9-blasticidin resistance to make stable Cas9 expressing cells for CRISPR KO screening
Recombinant DNA reagent	pMD2.G	Gift from Didier Trono	UK1700; RRID:Addgene_12259	Addgene plasmid #12259, lentiviral packaging helper (VSVG)
Recombinant DNA reagent	psPAX2	Gift from Didier Trono	UK1701; RRID:Addbebe_12260	Addgene plasmid #12260, lentiviral packaging helper
Recombinant DNA reagent	pKLV-U6gRNA(BbsI)PGKpuro2ABFP	Koike-Yusa et al., 2014	UK1789; RRID:Addgene_73542	Addgene #50946, BFP-2A-Puro-tagged gRNA vector
Recombinant DNA reagent	pKLV-CHO_libA-PGKpuro2ABFP	Ordóñez et al., 2021	UK2561	CRISPR-KO library of 125,030 selected guides for whole CHO genome screening
Recombinant DNA reagent	cgROSA26_BiP::sfGFP_HDR	This study	UK2846	DNA repair plasmid to integrate an ATF6 reporter (BiP::sfGFP) at ROSA26 locus in CHO genome
Recombinant DNA reagent	cgROSA26_sgRNA_pSp Cas9(BB)-2A-Puro	This study	UK2847	Puro-tagged sgRNA/Cas9 introduce double strand breaks in cgROSA26
Recombinant DNA reagent	pSpCas9 (BB)-2A-mTurquoise	This study	UK2915	pSpCas9(BB)-2A vector to express mTurquoise together with guide RNA and Cas9
Recombinant DNA reagent	cgATF6α_sgRNA_Ex8_pSp Cas9(BB)-2A-mTurquoise	This study	UK2919	mTurquoise-tagged sgRNA/Cas9 introduces double strand breaks in exon 8 of cgATF6α
Recombinant DNA reagent	cgERp18_sgRNA_g1_pSp Cas9(BB)-2A-mTurquoise	This study	UK2920	mTurquoise-tagged sgRNA/Cas9 introduces double strand breaks in exon 2 of TXNDC12
Recombinant DNA reagent	cgERp18_sgRNA_g2_pSp Cas9(BB)-2A-mTurquoise	This study	UK2921	mTurquoise-tagged sgRNA/Cas9 introduces double strand breaks in exon 5 of TXNDC12
Recombinant DNA reagent	cgIRE1sgRNA_lentiGuide-Puro	This study	UK 2929	Lentiviral vector expressing cgIRE1 CRISPR guide targeting exon 18 of IRE1 without expression of Cas9
Recombinant DNA reagent	cgATF6α_sgRNA_Ex2_pSpCas9 (BB)-2A-mCherry (MP1)	This study	UK2942	mCherry-tagged sgRNA/Cas9 introduces double strand breaks in exon 2 of cgATF6α
Recombinant DNA reagent	cgATF6_3xFLAG-mGL- TEV_HDR_pBS_V3 (MP1)	This study	UK2955	HDR template for mGreenLantern knock-in at N-term of Hamster ATF6
Recombinant DNA reagent	SP_FLAGM1_9E10_BirA_WT_K DEL_pCDNA5_FRT_TO_MP3	This study	UK2969	WT BirA, targeted to ER (with a signal peptide and a KDEL)
Recombinant DNA reagent	cgFURIN_sgRNA_Ex4_pSp Cas9(BB)-2A-mTurquoise	This study	UK2988	mTurquoise-tagged sgRNA/Cas9 introduces double strand breaks in exon 4 of cgFURIN
Recombinant DNA reagent	cgFURIN_sgRNA_Ex10_pSp Cas9(BB)-2A-mTurquoise	This study	UK2989	mTurquoise-tagged sgRNA/Cas9 introduces double strand breaks in exon 10 of cgFURIN
Recombinant DNA reagent	cgCRT_sgRNA_Ex1_pSp Cas9(BB)-2A-mTurquoise	This study	UK2990	mTurquoise-tagged sgRNA/Cas9 introduces double strand breaks in exon 1 of cgCRT
Recombinant DNA reagent	cgCRT_sgRNA_Ex3_pSp Cas9(BB)-2A-mTurquoise	This study	UK2991	mTurquoise-tagged sgRNA/Cas9 introduces double strand breaks in exon 3 of cgCRT
Recombinant DNA reagent	cgATF6_g1A_ex9_pSp Cas9(BB)2A-mTurquoise_MP2	This study	UK3021	mTurquoise-tagged sgRNA/Cas9 introduces double strand breaks in exon 9 of cgATF6α (PR3125 and 3126 based)
Recombinant DNA reagent	cgATF6_g2A_ex10_pSp Cas9(BB)2A-mTurquoise_MP2	This study	UK3024	mTurquoise-tagged sgRNA/Cas9 introduces double strand breaks in exon 10 of cgATF6α (PR3133 and 3134 based)
Recombinant DNA reagent	cgATF6_g2B_ex10_pSp Cas9(BB)2A-mTurquoise_MP2	This study	UK3025	mTurquoise-tagged sgRNA/Cas9 introduces double strand breaks in exon 10 of cgATF6α (PR3135 and 3136 based)
Recombinant DNA reagent	cgATF6_TV_EX8-9_Strep-Tag- Twin_V.1.3_pUC57	This study	UK3054	Genescript plasmid encodes cgATF6 LD^WT^ for rescue experiments
Recombinant DNA reagent	cgATF6_410-659_noGly_V1_pUC57	This study	UK3086	pUC57 with ATF6 CDS gene fragment (all three glycans removed) for rescue experiments
Recombinant DNA reagent	EGFP_cgATF6_TEV_410-659_ S12MUT_AviTag_pCEFL_pu (MP4)	This study	UK3122	EGFP replacing the cytosolic domain of cgATF6 LD with a -AviTag and with S1P/S2P mutations in
Recombinant DNA reagent	EGFP_cgATF6_TEV_410-659_ S12MUT_noGly_AviTag_pCEFL_pu (MP1)	This study	UK3141	Glycan-free counterpart of UK3122 (TEV cleavage after TM)
Recombinant DNA reagent	rCRT_18-416_pSUMO3 (MP3)	This study	UK3142	Bacterial expression of luminal fragment of rat Calreticulin
Recombinant DNA reagent	cgATF6_TV_EX8-9_Strep- tagtwin_CtoS_pUC57 mp7	This study	UK3147	Recombination template, UK3054 based, two cysteines changed to serine for rescue experiments
Recombinant DNA reagent	EGFP_cgATF6_TEV_410-659_S1& 2MUT_CtoS_AviTag_pCEFL_pu_MP2	This study	UK3157	Cysteine free counterpart of UK3122 (TEV cleavage after TM)
Recombinant DNA reagent	cgCNX_sgRNA_Ex2_pSp Cas9(BB)-2A-mTurquoise_MP1	This study	UK3206	mTurquoise-tagged sgRNA/Cas9 introduces double strand breaks in exon 2 of cgCNX (PR3364 and 3365 based)
Recombinant DNA reagent	cgCNX_sgRNA_Ex4_pSp Cas9(BB)-2A-mTurquoise_MP2	This study	UK3207	mTurquoise-tagged sgRNA/Cas9 introduces double strand breaks in exon 4 of cgATF6α (PR3366 and 3367 based)
Recombinant DNA reagent	hCRT_WT_pCEFL_BFP	This study	UK3231	BFP-marked mammalian expression of untagged, WT human Calreticulin
Recombinant DNA reagent	hCRT_Y92A_pCEFL_BFP	This study	UK3232	BFP-marked mammalian expression of untagged, human Calreticulin lectin mutant
Recombinant DNA reagent	hCRT_Y92A&W244A_pCEFL_BFP	This study	UK3233	BFP-marked mammalian expression of untagged, human Calreticulin functional doble mutant
Sequence-based reagent	cgATF6a_ex2_sgRNA_1S	This study	3010	CACCGGCTGGTGCGGCCGGGACCA
Sequence-based reagent	cgATF6a_ex2_sgRNA_2AS	This study	3011	AAACTGGTCCCGGCCGCACCAGCC
Sequence-based reagent	cgATF6a_sgRNA_ex8_1S	This study	2921	CACCGAAGAAGAAGGAGTATATGC
Sequence-based reagent	cgATF6a_sgRNA_ex8_1S	This study	2922	AAACGCATATACTCCTTCTTCTTC
Sequence-based reagent	cgATF6a_sgRNA_ex9_1S	This study	3125	CACCGTTTCCTAGATTGCTGTGCTA
Sequence-based reagent	cgATF6a_sgRNA_ex9_2AS	This study	3126	AAACTAGCACAGCAATCTAGGAAAC
Sequence-based reagent	cgATF6a_sgRNA_ex10_1S	This study	3133	CACCGCATTTATAATGCTGAACTA
Sequence-based reagent	cgATF6a_sgRNA_ex10_2AS	This study	3134	AAACTAGTTCAGCATTATAAATGC
Sequence-based reagent	cgATF6a_sgRNA_ex10_3S	This study	3135	CACCGAGGTGAGTATTCAAGTGTT
Sequence-based reagent	cgATF6a_sgRNA_ex10_4AS	This study	3136	AAACAACACTTGAATACTCACCTC
Sequence-based reagent	cgCRT_sgRNA_ex1_1S	This study	3068	CACCGTTCCGCGGCGGCCAGGCCG
Sequence-based reagent	cgCRT_sgRNA_ex1_2AS	This study	3069	AAACCGGCCTGGCCGCCGCGGAAC
Sequence-based reagent	cgCRT_sgRNA_ex3_1S	This study	3070	CACCGCGCGTAAAATCGGGCATCC
Sequence-based reagent	cgCRT_sgRNA_ex3_2AS	This study	3071	AAACGGATGCCCGATTTTACGCGC
Sequence-based reagent	cgFURIN_sgRNA_ex4_1S	This study	3064	CACCGTGGTCTCCATCCTGGACGA
Sequence-based reagent	cgFURIN_sgRNA_ex4_2AS	This study	3065	AAACTCGTCCAGGATGGAGACCAC
Sequence-based reagent	cgFURIN_sgRNA_ex10_1S	This study	3066	CACCGCCACCTCAATGCTAATGAT
Sequence-based reagent	cgFURIN_sgRNA_ex10_2AS	This study	3067	AAACATCATTAGCATTGAGGTGGC
Sequence-based reagent	cgCNX_sgRNA_ex2_1S	This study	3364	CACCGCAAAGCTCCAGTTCCAACAG
Sequence-based reagent	cgCNX_sgRNA_ex2_2AS	This study	3365	AAACCTGTTGGAACTGGAGCTTTGC
Sequence-based reagent	cgCNX_sgRNA_ex4_3S	This study	3366	CACCGGCTTGGTATCAAACAGGAA
Sequence-based reagent	cgCNX_sgRNA_ex4_4AS	This study	3367	AAACTTCCTGTTTGATACCAAGCC
Sequence-based reagent	cgERp18_sgRNAg1_ex2_1S	This study	2962	CACCGTTTTGGAGATCATATTCAC
Sequence-based reagent	cgERp18_sgRNAg1_ex2_2AS	This study	2963	AAACGTGAATATGATCTCCAAAAC
Sequence-based reagent	cgERp18_sgRNAg2_ex5_1S	This study	2964	CACCGTGGAATATAACCCCCATCA
Sequence-based reagent	cgERp18_sgRNAg2_ex5_2AS	This study	2965	AAACTGATGGGGGTTATATTCCAC
Sequence-based reagent	cgXBP1.19S5	This study	1470	GGCCTTGTAATTGAGAACCAGGAG
Sequence-based reagent	cgXBP1.14AS	This study	5	GAATGCCCAAAAGGATATCAGACTC
Antibody	Polyclonal Rabbit anti-calreticulin	Abcam	Cat# ab19261, RRID:AB_955722	Used at 1:1000
Antibody	Polyclonal Chicken anti-calreticulin	Invitrogen	Cat# PA1-902A, RRID:AB_2069607	Used at 1:1000
Antibody	Polyclonal Rabbit anti-calnexin	Enzo Life Sciences	Cat# ADI-SPA-860-D, RRID:AB_2038898	Used at 1:1000
antibody	Polyclonal Rabbit anti-FURIN	Abcam	Cat# ab3467, RRID:AB_303828	Used at 1:1000
Antibody	Monoclonal Mouse anti IRE1a_LD serum	Bertolotti et al., 2000	NY200	Used at 1:1000
Antibody	Monoclonal Rabbit anti p-IRE1	Genetech		Used at 1:1000
Antibody	Polyclonal Chicken anti-hamster BiP	Avezov et al., 2013	anti-BiP	Used at 1:1000
Antibody	Monoclonal Mouse anti FLAG-M2	Sigma	Cat# F1804, RRID:AB_262044	Used at 1:1000
Antibody	Polyclonal Rabbit anti GFP	Marciniak et al., 2004	NY	Used at 1:1000
Antibody	Monoclonal Mouse anti KDEL (10C3)	Enzo Life Sciences	ENZ-ABS679	Used at 1:1000
Antibody	Monoclonal Mouse anti-actin	Fisher Scientific	clone C4-691002	Used at 1:1000
Antibody	Monoclonal Rabbit anti-GAPDH	Cell Signalling	14C10; mab# 2118	Used at 1:2000
Antibody	Polyclonal rabbit polyclones anti-α/β-Tubulin	Cell Signalling	CST2148S	Used at 1:1000
Chemical compound, drug	DMEM	Sigma	D6429	
Chemical compound, drug	HyClone II Serum	Thermo Fisher Scientific	SH30066.03	
Chemical compound, drug	Penicillin/Streptomycin	Sigma	P0781	
Chemical compound, drug	L-Glutamine	Sigma	G7513	
Chemical compound, drug	Non-essential amino acids solution	Sigma	M7145	
Chemical compound, drug	Nutrient Mixture F12	Sigma	N4888	
Chemical compound, drug	Lipofectamine LTX	Thermo Fisher Scientific	A12621	
Chemical compound, drug	Puromycin	MERCK-milipore	540222	
Chemical compound, drug	EDTA-free Protease inhibitor Cocktail	Roche	11873580001	
Chemical compound, drug	Anti-FLAG M2 Affinity Gel	Sigma	F3165	
Chemical compound, drug	ChromoTek GFP-Trap Agarose	Thermo Fisher Scientific	Tag-20	
Chemical compound, drug	Tunicamycin	Melford	T2250	
Chemical compound, drug	2-Deoxyglucose (2DG)	ACROS Organics	D6134	
Chemical compound, drug	Ceapin A7	Sigma	SML2330	
Chemical compound, drug	Thapsigargin	Calbiochem	CAS 67526-95-8	
Chemical compound, drug	PF-429242 dihydrochloride (S1P inhibitor)	Tocris	SML0667	
Chemical compound, drug	4μ8C	Tocris Bioscience	4479	
Chemical compound, drug	TRIzolä Reagent	Ambion/Invitrogen	15596026	
Chemical compound, drug	PureLinkä RNA mini kit	Invitrogen	12183018A	
Chemical compound, drug	RevertAid Reverse Transcriptase	Thermo Scientific	EP0441	
Chemical compound, drug	FURIN	New England Biolabs	P8077S	
Chemical compound, drug	MBP5-FN-paramyosin-ΔSal	New England Biolabs	E8052S	
Chemical compound, drug	MycoAlert (TM) Mycoplasma Detection Kit	Lonza	LT07-118	
Cell line (*Cricetulus griseus*)	CHO XC45-6S	This study	XC45-6S	Parental ATF6α/IRE dual UPR reporter CHO cell line expressing XBP1s::mCherry and BiP:sfGFP reporters
Cell line (*Cricetulus griseus*)	XC45-6S cells-Cas9 stable*#21*	This study	XC45-6S-Cas9 stable*#21*	Parental ATF6α/IRE dual UPR reporter CHO cell line expressing XBP1s::mCherry and BiP:sfGFP reporters stably expressing Cas9
Cell line (*Cricetulus griseus*)	CHO-K1 *XC45-6S*, XBP1s::mCherry & BiP:sfGFP *ATF6*α∆ (KO)	This study	XC45-6S, *ATF6*∆*, #B6*	ATF6α/IRE dual reporter cell line with depletion of ATF6_clone B6
Cell line (*Cricetulus griseus*)	CHO-K1 *XC45-6S*, XBP1s::mCherry & BiP:sfGFP *ATF6*α∆ (KO)	This study	XC45-6S, *ATF6*∆*, #5*A2	ATF6α/IRE dual reporter cell line with depletion of ATF6_clone 5A2
Cell line (*Cricetulus griseus*)	CHO-K1 *XC45-6S*, *ATF6*αLD^WT^ rescue	This study	XC45-6S, *ATF6*αLD^WT^ *#R2_9*	ATF6α/IRE dual reporter cell line with depletion of ATF6_LD and rescue with *ATF6*αLD^WT^ _clone R2.9
Cell line (*Cricetulus griseus*)	CHO-K1 *XC45-6S*, *ATF6*αLD^∆Gly^ rescue	This study	XC45-6S, *ATF6*αLD^∆Gly^*#GF6*	ATF6α/IRE dual reporter cell line with depletion of ATF6_LD and rescue with *ATF6*αLD^∆Gly^_clone 6
Cell line (*Cricetulus griseus*)	CHO-K1 *XC45-6S*, *ATF6*αLD^∆C^ rescue	This study	XC45-6S, *ATF6*αLD^∆C^*#CS10*	ATF6α/IRE dual reporter cell line with depletion of ATF6_LD and rescue with *ATF6*αLD^∆C^_clone 10
Cell line (*Cricetulus griseus*)	CHO-K1 3xFLAG-mGL_ATF6α knock-in cells	This study	2K cells	CHO-K1 cells with endogenous ATF6 tagged with a 3xFLAG-mGL tag
Cell line (*Cricetulus griseus*)	CHO-K1 GFP-cgATF6αLD_S1P&S2P^mut^	This study	UK3122 cells	CHO-K1 stable expressing a GFP-cgATF6αLD_S1P&S2P^mut^
Cell line (*Cricetulus griseus*)	CHO-K1 *XC45-6S*, *CRT*∆*#2*P	This study	XC45-6S, CRTα∆#2P	ATF6α/IRE dual reporter cell line with depletion of CRT_clone 2P
Cell line (*Cricetulus griseus*)	CHO-K1 *XC45-6S*, *CRT*∆*#2Q*	This study	XC45-6S, CRTα∆#2Q	ATF6α/IRE dual reporter cell line with depletion of ATF6-clone 2Q
Cell line (*Cricetulus griseus*)	CHO-K1 *XC45-6S*, *FURIN*∆*#2.2*.G	This study	XC45-6S, FURIN∆#2.2.G	ATF6α/IRE dual reporter cell line with depletion of Furin_ clone *2.2*.G
Cell line (*Cricetulus griseus*)	CHO-K1 *XC45-6S*, *CNX*∆*#6*	This study	XC45-6S, CNX∆#6	ATF6α/IRE dual reporter cell line with depletion of CNX_clone *6*
Cell line (*Cricetulus griseus*)	CHO-K1 *XC45-6S*, *ERp18*∆*#7E2*	This study	XC45-6S, ERp18∆#7E2	ATF6α/IRE dual reporter cell line with depletion of ERp18_clone *7E2*
Software, algorithm	MAGeCK	Li et al., 2014		
Software, algorithm	Metascape	Zhou et al., 2019		
Software, algorithm	MacVector			
Software, algorithm	Photoshop and Illustrator			
Software, algorithm	FlowJo			
Software, algorithm	ImageJ			
Software, algorithm	GraphPad-Prism V8			
Software, algorithm	Volocity V6.3	PerkinElmer		

### Mammalian cell culture, transfections, and treatments

Adherent Chinese Hamster Ovary (CHO-K1) cells (ATCC CCL-61) were maintained in Ham’s nutrient mixture F12 (Sigma) supplemented with 10% (vol/vol) serum (FetalClone-2, HyClone), 2 mM l-glutamine (Sigma), and 1% penicillin/streptomycin (Sigma). HEK293T cells (ATCC CRL-3216) were cultured in Dulbecco's Modified Eagle Medium (DMEM) cell media (Sigma) supplemented as above. All cells were grown in a humidified 37°C incubator with 5% CO_2_ and passed every 2–3 days. When indicated, cells were treated with 5 μM Ceapin A7 (SML2330; Sigma), 15 μM S1P inhibitor (PF429242 dihydrochloride; Tocris), 2.5 μg/ml Tunicamycin (Tm, Melford), 4 mM 2DG (ACROS Organics), 0.5 μM Thapsigargin (Tg; Calbiochem), and 16 μM 4μ8C (Sigma) for the indicated times. All compounds were diluted in pre-warmed culture medium and immediately added to the cells by medium exchange. Untreated cells were treated with dimethylsulfoxide (DMSO) solvent vehicle control. Transfections in CHO-K1 cells were performed using Lipofectamine LTX (Thermo Fisher Scientific, USA) at 1:3 DNA (µg) to LTX (µl) ratio, as for HEK293T using TransIT-293 Transfection Reagent (MIR2704, Mirus), according to the manufacturer’s instructions. Typically, cells were seeded at a density of 2.5 × 10^5^ cells/well in 6-well plates a day before transfection and analysed 48–72 hr after transfection.

The cell lines used in this study have tested negative for mycoplasma contamination using the MycoAlert Mycoplasma Detection Kit (Lonza). None of the cell lines appear on the list of commonly misidentified cell lines maintained by the International Cell Line Authentication Committee. The identity of the CHO-K1 cell lines has been authenticated by successfully targeting essential genes using a species-specific CRISPR whole-genome library and sequencing the WT or mutant alleles of the genes studied, which confirmed the sequences reported for the corresponding genome.

### Mammalian cell lysates and immunoblotting

CHO-K1 or HEK293T cells were cultured in 6-, 12-well plates, or 10-cm dishes until reaching 95% confluence. Cells were washed twice with prechilled phosphate-buffered saline (PBS) on ice, and whole-cell extracts were scrapped out in 1 mM EDTA (Ethylenediaminetetraacetic Acid)–PBS, pelleted at 370 × *g* for 10 min at 4°C and incubated in Nonidet P40 (NP-40) lysis buffer (150 mM NaCl, 50 mM Tris–HCl pH 7.5, 1% (vol/vol) NP-40) supplemented with 2× protease inhibitor cocktail (Roche Applied Science) for 30 min. Next, the samples were clarified at 21,130 × *g* for 20 min at 4°C and the supernatants were transferred to fresh tubes. Protein concentration was determined using Bio-Rad protein assay. To assess the interaction between CRT and ATF6α through either BLI or co-immunoprecipitation, the lysis buffer was supplemented with 5 mM CaCl_2_. The protein samples were separated on 8–12.5% SDS–PAGE under reducing conditions and transferred onto PVDF (Polyvinylidene Fluoride) membranes as described previously (Ordóñez et al., 2013). Membranes were probed with the following primary antibodies: rabbit polyclonal anti-calreticulin (1:1000) (ab19261, abcam; to detect human CRT); chicken polyclonal anti-calreticulin (1:1000) (PA1-902A, Invitrogen; to detect Chinese hamster CRT); rabbit polyclonal anti-calnexin (1:1000) (SPA-860, Enzo Life Sciences); rabbit polyclonal anti-FURIN (1:1000) (ab3467, abcam); mouse monoclonal anti IRE1_LD serum (NY200) (1:1000) Bertolotti et al., 2000; rabbit monoclonal anti p-IRE1 (1:1000) (Genetech); rabbit polyclonal anti GFP (NY1066) (1:1000) (Marciniak et al., 2004) made against purified bacterially expressed GFP following removal of the GST affinity tag via thrombin cleavage site; chicken anti-BiP IgY (1:1000) Avezov et al., 2013; mouse monoclonal anti-FLAG M2 (1:1000) (F1804, Sigma); mouse monoclonal anti-KDEL (10C3) (1:1000) (ENZ-ABS679; Enzo Life Sciences); mouse monoclonal anti-actin (1:1000) (clone C4-691002, Fisher Scientific); rabbit monoclonal anti-GAPDH (1:2000) (14C10; Cell Signalling Technology); rabbit polyclonal anti-α/β-Tubulin (1:1000) (CST2148S, Cell Signalling Technology). For secondary antibody, IRDye fluorescently labelled antibodies or horseradish peroxidase antibodies were used. Membranes were scanned using an Odyssey near infrared imager (LI-COR) and signals were quantified using ImageJ software.

Signal quantification and analysis were performed using Prism 10 (GraphPad).

### Flow cytometry and FACS

To analyse UPR reporter activities, cells were grown on 6- or 12-well plates until reaching 80-90% confluence and then treated as indicated. For flow cytometry analysis, cells were washed twice in PBS, collected in PBS supplemented with 4 mM EDTA, and 20,000 cells/sample were analysed by multi-channel flow cytometry on a LSRFortessa cell analyser (BD Biosciences). For FACS, cells were collected in PBS containing 4 mM EDTA and 0.5% bovine serum albumin (BSA) and then sorted on a Beckman Coulter MoFlo cell sorter. Sorted cells were either collected in fresh media as a bulk of cells or individually sorted into 96-well plates and then expanded. Gating for live cells was based on FSC-A/SSC-A and for singlets was based on FSC-W/SSC-A. BiP::sfGFP and mGreenLantern (mGL) fluorescent signals were detected with an excitation laser at 488 nm and a 530/30 nm emission filter; XBP1s::mCherry fluorescence with an excitation laser 561 nm and a 610/20 nm emission filter, while mTurquoise and BFP fluorescence with an excitation laser 405 nm and a 450/50 nm filter emission. Data analysis was performed using FlowJo V10, and median reporter analysis was conducted using Prism 10 (GraphPad).

### Lentiviral production

Lentiviral particles were produced by co-transfecting HEK293T cells with the library plasmid (UK2561), the packaging plasmids psPAX2 (UK1701) and pMD2.G (UK1700) at 10:7.5:5 ratio using TransIT-293 Transfection Reagent (MIR2704, Mirus) according to the manufacturer’s instructions. Eighteen hours after transfection, medium was changed to medium supplemented with 1% BSA (Sigma). The supernatant containing the viral particles was collected 48 hr after transfection, filtering through a 0.45-μm filter, and directly used to infect CHO-K1 cells seeded in 6-well plates for viral titration to calculate the amount of virus to use aiming a low MOI around 0.3 to ensure a single integration even per cell.

### Generation of the *XC45-6S* double UPR reporter cell by genome editing in *ROSA26* locus of CHO-K1 cells

The putative *ROSA26* locus in CHO cells has been recently identified and described as a ‘safeharbour’ site for heterologous gene expression and stable long-term expression of a transgene (Gaidukov et al., 2018). To generate a double UPR reporter cell line, we used a previously described *XC45* CHO-K1 cell line bearing a XBP1s::mCherry reporter expressing a pCAX-F-XBP1∆DBD-mCherry transgene randomly integrated in the genome (Harding et al., 2019), for targeting the *ROSA26* locus for knock-in with a landing pad cassette, comprised the *cg*BiP promoter region containing ERSE-I elements, fused to the superfolded (sf) GFP into the CHO genome by CRISPR/Cas9 integration to make this reporter cell line also sensitive to ATF6α activity. The landing pad donor vector was constructed by appending homology arm sequences of *ROSA26* locus (440 bp predefined sequence from Gaidukov’s paper) to the 5′ promoter region of *cg*BiP (1000 bp), along with the first eight amino acids of the BiP CDS fused to the sfGFP. Homology arms were PCR amplified from CHO genomic DNA using predefined primers from Gaidukov’s paper. Subsequently, the *cg*BiP promoter, a geneblock containing sfGFP, and the flanking homology arms were fused by Gibson assembly cloning method, which allows multiple DNA fragments joining in a single, isothermal reaction (1 hr at 50°C). Targeted integration was performed by co-transfection with the circular landing pad donor vector (UK2846) and a single sgRNA/Cas9 targeting *cgROSA26* locus (UK2847). Transfected cells were selected with puromycin (8 μg/ml) for 3 days and then treated with 2DG (4 mM) for 20 hr to induce ER stress prior FACS. Cells were phenotypically sorted into 96-well plates by their activation of both UPR reporters (mCherry^high^; GFP ^high^). Phenotypically selected clones were subsequently confirmed by correct genetic integration. For stability integration, clones were maintained and expanded every 2–3 days for 1 month and weekly analysed with flow cytometry for mCherry and GFP expression under treatment with different ER stressors agents. For the genome-wide CRISPR/Cas9 screen, Cas9 was ultimately introduced by transduction with lentiviral particles prepared from packaging Lenti-Cas9 plasmid (UK1674). Cas9 activity was confirmed by targeting the exon 18 of IRE1 (*ERN1* locus) (UK2929) followed by induction of ER stress with 2DG (4 mM) for 20 hr (Figure 1—figure supplement 1F).

### CRISPR/Cas9 screen

The screen was performed following established protocols using a pooled CHO-K1 knockout CRISPR library containing 125,030 sgRNAs targeting 21,896 genes, with six guides per gene, as well as 1000 non-targeting sgRNAs as a negative control cloned into the lentiviral sgRNA expression vector pKLV-U6gRNA(BbsI)-PGKpuro2ABFP (Shalem et al., 2014; Ordóñez et al., 2021). Approximately 2.1 × 10^8^ CHO-K1 Cas9 stable cells carrying the dual UPR reporter ATF6α/IRE1 (*XC45-6S* cells) were infected at an MOI of 0.3 to favour infection with a single viral particle per cell. Two days after infection, transduced cells were selected with 8 µg/ml puromycin for 14 days. Afterwards, transduced cells were split into two subpopulations: one was treated with 2DG to induce ER stress, while the other one remained untreated. For the *ATF6*α *activator screen*, conducted under ER stress conditions, less than 2% of cells that showed a XBP1s::mCherry^high^; BiP::sfGFP^low^ phenotype were selected for analysis. Similarly, for the *ATF6*α *repressor screen*, conducted under basal (untreated) conditions, less than 2% of total sorted cells that showed a XBP1s::mCherry^low^; BiP::sfGFP^high^ phenotype were selected for analysis. Rounds of enrichment were carried out through cellular recovery, expansion, and sorting. Equal number of transduced cells, both untreated or 2DG treated, was passed without sorting as a control group in each round of sorting. To prepare samples for deep sequencing, genomic DNA from enriched and sorted populations as well as unsorted cells (to represent the entire library) was extracted from ~3.6 × 10^7^ and~1–3 × 10^6^ cells, respectively, by incubation in proteinase K solution [100 mM Tris–HCl pH 8.5, 5 mM EDTA, 200 mM NaCl, 0.25% (wt/vol) SDS, 0.2 mg/ml Proteinase K] overnight at 50°C. Integrated sgRNA sequences were amplified by nested PCR and the necessary adaptors for Illumina sequencing were introduced at the final round of amplification. The purified products were quantified and sequenced on an Illumina NovaSeq 6000 by 50 bp single-end sequencing. Downstream analyses to obtain sgRNA read counts, gene rankings, and statistics were performed with MAGeCK software (Li et al., 2014). GO analyses were performed using Metascape with default parameters.

### Knockout cells using CRISPR/Cas9 technology

sgRNAs targeting exon regions of *C. griseus ATF6*α, *CRT*, *CNX*, *FURIN*, and *ERp18* were designed using CRISPR-Design tools from *CCTop* (https://cctop.cos.uniheidelberg.de:8043/index.html) and *CRISPy-Cas9 target finder for CHO-K1* (http://staff.biosustain.dtu.dk/laeb/crispy/) databases. sgRNAs were then cloned into the pSpCas9(BB)-2A-mTurquoise (UK2915) or pSpCas9(BB)-2A-Puro plasmids (UK1367) as previously reported (Ran et al., 2013). The integrity of the constructs was confirmed by DNA sequencing. To generate the knockout cells, 0.75–1 µg of sgRNA/Cas9 plasmids was transfected into *XC45-6S* cells using Lipofectamine LTX (Thermo Fisher) in a 6-well plate format. After 48 hr post-transfection, mTurquoise^high^ cells were sorted into 96-well plates at 1 cell/well using a MoFlo Cell Sorter (Beckman Coulter). For cells transfected with sgRNA/Puro plasmids, selection was performed in the presence of 8 μg/ml puromycin, and serial dilution was used to isolate single clones. The knockouts cells were confirmed by Sanger sequencing and immunoblotting.

### ATF6α-tagged cells by CRISPR/Cas9 genome editing

To investigate potential modulators of ATF6α activity following the CRISPR/Cas9 high-throughput screen using biochemistry and microscopy-compatible tools, two mammalian cell lines were engineered. These cell lines either harboured a knock-in of the endogenous *cgATF6*α locus or carried a stable transgene encoding ATF6α_LD tagged with a bright GFP. The tags were positioned at the N-cytosolic terminus, known to be well tolerated.

#### 3xFLAG-mGreenLantern_ATF6α knock-in cells (*2K* cells)

Protein alignment of *hs*ATF6α and *cg*ATF6α (UniProt database) indicated that translation initiation predominantly occurs at the second Met at the beginning of exon 2 (MESP). Instead of tagging at the first Met in the CHO genome, tags were introduced before the third Met. CHO-K1 plain cells were offered with a unique pSpCas9(BB)-2A-mCherry targeting exon 2 of *cg*ATF6α locus (UK2942) and a donor plasmid containing homology arms flanking a minigene encoding a 3xFLAGmGreenLantern(mGL)-TEV sequence geneblock (UK2955) to create an endogenous tagged ATF6α. After 48 hr post-transfection, mGL^high^ cells were sorted as single cells into 96-well plates. Clones that preserved stable mGL fluorescent signal by flow cytometry and showed expected ATF6α processing upon ER stress induction (DTT) were subsequently confirmed by correct genetic integration. Microscopy characterisation was performed under basal conditions or after treatments with ER stressors (DTT), Ceapin A7 and S1P inhibitor. A clone referred to as *2K* was expanded and selected for functional analysis as a 3xFLAG-mGL-tagged endogenous ATF6α.

#### GFP-cgATF6α_LD^S1P,S2Pmut^ (*UK3122* cells)

ATF6 has a half-life of about 3 hr (George et al., 2020). To increase the proportion of intact, active N-ATF6α molecules reaching the Golgi, a genetic construct featured the TM and LD of ATF6α deliberately lacking S1P and S2P cleavage sites, and further strategically appended with an Avi-tag at the C-terminal, was engineered. This construct was then used for a dual purpose: as a tool for both microscopy studies as well as to be biotinylated and purified to be used as a ligand in BLI experiments. Shen and Ye’s research provided the basis for the design of S2P and S1P mutations (N391F and P394L for S2P mutant, and LL408/9VV for S1P mutant) (Shen et al., 2002; Ye et al., 2000). A DNA-plasmid encoding eGFP_cgATF6α_TEV_LD410-659_S12PMUT_AviTag_pCEFL_pu (UK3122) was introduced into CHO-K1 plain cells via Lipofectamine LTX. Transfected cells were selected for resistance to puromycin (8 µg/ml), and the isogenic clones were selected for confocal microscopy assays and *in vitro* BLI interaction assays.

### A genetic platform to generate cgATF6α LD variants knock-in cells

#### ATF6α∆

Two sgRNA sequences targeting exons 9 and 10 of cg*ATF6*α were incorporated into pSpCas9(BB)-2A-mTurquoise plasmid (UK2915) to generate two sgRNA/Cas9 expression plasmids (UK3021 and UK3024) according to the published procedure (Ran et al., 2013). These plasmids were then transfected into CHO-K1 *XC45-6S* double UPR reporter cells. After 72 hr, mTurquoise^high^ cells were sorted as single cells into 96-well plates. Genomic DNA from clones showing no response to ATF6α activation (BiP::sfGFP^low^) upon tunicamycin treatment (0.5 µM, 20 hr) was extracted for Sanger sequencing. Clones with frameshifts causing deletions in both alleles of the DNA region flanking by both sgRNAs were selected for further functional assays.

#### cgATF6αLD ‘minigenes’ knock-in

A unique sgRNA sequence targeting the exon 10 of α*ATF6*α**∆** cells was inserted into the pSpCas9(BB)-2A-mTurquoise plasmid to create the sgRNA/Cas9 plasmid UK3025. Three repair ‘minigenes’ templates were constructed: A WT, glycan-free (∆Gly), or a cysteine-free (∆C) LD to be reconstituted in ATF6α∆ cells by CRISPR/Cas9-mediated HDR.

For the construction of the WT LD repair template (WT; UK3054), a minigene block that contains exons 10–17 of *cgATF6*α (785 bp; GenScript) was digested with *Age*I and *Afl*II and ligated into *Age*I/ *Afl*II-digested pUC57 plasmid. To create a Glycan free LD (∆Gly; UK3086) template, a minigene block containing exons 10–17 (GenScrip) with the three *N*-glycosylation sites were mutated by replacing Thr 464, 575, and 634 with Ala, was cloned into pUC57 plasmid as previously described. Similarly, to generate a cysteine-free LD (∆C; UK3147) template, a minigene block containing exons 10–17 (GenScrip) with Cys457 and Cys607 replaced with Ser, was cloned into pUC57 plasmid as previously performed. The integrity of the constructs was confirmed by DNA sequencing.

### Immunoprecipitation using GFP-Trap Agarose

Equal volumes of the cleared and normalised lysates were incubated with 15–20 μl GFP-Trap Agarose (ChromoTek) pre-equilibrated in lysis buffer. The mixture was incubated overnight at 4°C with rotation. The beads were then recovered by centrifugation (845 × *g*, 3 min) and washed four times with washing buffer (50 mM Tris–HCl, 1 mM EDTA, 0.1% Triton X-100) and one more with PBS. Proteins were eluted in 35 μl of 2× SDS–DTT sample for 5 min at 95°C. Equal sample volumes were analysed by SDS–PAGE and immunoblotting, as described above. Normalised cell lysates (15–30 μg) were loaded as an ‘input’ control.

### BLI, protein expression, and *in vivo* biotinylation

BLI studies were undertaken using on the Octet RED96 System (Pall FortéBio) using ATF6α_LD variants as biotinylated ligands that were immobilised on streptavidin (SA)-coated biosensors (Pall FortéBio) and rat calreticulin (*r*CRT) as the analyte. Prior to use, SA‐coated biosensors were hydrated in a BLI buffer (150 mM NaCl, 20 mM Tris, 1 mM CaCl_2_) for 10 min. BLI reactions were prepared in 200 μl volumes in 96‐well microplates (Greiner Bio‐One), at 30°C. Ligand loading was performed for 600 s until a binding signal of 1 nm displacement was reached. Following ligand immobilisation, the sensor was baselined in the reaction buffer for at least 200 s, after which it was immersed in wells containing the analyte for association for 1200s. After each association, the sensor was dipped into a buffer containing well to allow dissociation for 2400s.

Biotinylated ATF6αLD constructs [ATF6αLD^WT^ (UK3122); ATF6αLD^∆Gly^ (UK3141); and ATF6LD^∆C^ (UK3157)] were expressed in HEK293T cells, each one encoding a N-terminal GFP tag followed by a TEV protease cleavage site just after the TM and an Avi-tag at C-terminal. S1P and S2P cleavage sites were also mutated to increase expression yield. Biotinylation was conducted *in vivo*, where cells were co-transfected with a BirA-encoding plasmid (biotin ligase, UK2969) in a 1:0.06 ratio. After 24 hr, media was replaced with fresh media containing 50 μM biotin dissolved in DMSO and cells were harvested and lysed the following day. The lysis buffer was supplemented with 5 mM CaCl_2_. Post immunoprecipitation using GFP-Trap Agarose, proteins were eluted by incubation with an excess of TEV protease (UK759, 16 μg) in 400 μl TEV cleavage buffer [(25 mM Tris–HCl pH 7.4, 150 mM NaCl, 0.05% NP-40, 0.05 mM CaCl_2_, 0.05 mM tris(2-carboxyethyl)phosphine (TCEP))] for 16 hr at 4°C with orbital rotation. Cleavage products were snap-frozen in liquid nitrogen and stored at −80°C for BLI experiments.

WT full-length *r*CRT (UK3142) was expressed in *E. coli* cells (BL21 C3013). Bacterial cultures were grown in LB medium with 100 µg/ml ampicillin at 37°C to an OD_600 nm_ of 0.6 to 0.8. Expression was induced with 1 mM isopropyl β- d-1-thiogalactopyranoside (IPTG), and the cells were further grown for 16 hr at 18°C. After cell sedimentation by centrifugation, the pellets were lysed with a high‐pressure homogeniser (EmulsiFlex‐C3; Avestin) in bacterial lysis buffer (50 mM Tris–HCl pH 7.4, 500 mM NaCl, 2 mM CaCl_2_, 20 mM imidzole) containing protease inhibitors (1 mM phenylmethylsulfonyl fluoride (PMSF), 2 µg/ml pepstatin, 8 µg/ml aprotinin, 2 µM leupeptin) and 0.1 mg/ml DNaseI. Typical yields from 2 l of bacterial culture were obtained.

### Immunoprecipitation using GFP-Trap Agarose

To investigate the interaction between CRT and ATF6α in cells, HEK293T cells were cultured in 10 cm dishes (3–4 plates per sample) and transfected with constructs expressing either GFPATF6α_LD^WT^ (UK3122) or GFP-ATF6α_LD^∆Gly^ (UK3141). Equal volumes of the cleared and normalised lysates were incubated with 15–20 μl GFP-Trap Agarose (ChromoTek) preequilibrated in lysis buffer. The mixture was incubated overnight at 4°C with rotation. The beads were then recovered by centrifugation (845 × *g*, 3 min) and washed four times with washing buffer (50 mM Tris–HCl, 1 mM EDTA, 0.1% Triton X-100). Proteins were eluted in 35 μl of 2× SDS–DTT sample for 5 min at 95°C. Equal sample volumes were analysed by SDSPAGE and immunoblotting, as described above. Normalised cell lysates (15–30 μg) were loaded as an ‘input’ control.

### Analysis of endogenous ATF6α processing in CRT-depleted cells

To investigate the impact of CRT depletion on ATF6α cleavage, CRT was depleted in *2K* cells 3xFLAG-mGL-ATF6α by CRISPR/Cas9 gene editing (UK2991). *2K-CRT*∆ derivative clones were confirmed by Sanger sequencing and selected for further experiments. Both *2K* parental cells and *2K-CRT*∆ derivative cells were cultured in 10-cm dishes for 2 days and then treated with 2DG (4 mM) for a time course up to 6 hr before harvested. Following treatment, the cells were lysed in Nonidet-lysis buffer for extracting the cytosolic fraction, as previously described. For nuclear extraction, high-salt lysis buffer (500 mM NaCl, 50 mM Tris–HCl pH 7.5, 1% (vol/vol) NP-40) supplemented with 2× protease inhibitor cocktail was added to the nuclear pellets, followed by rotation at 4°C for 2 hr. Subsequently, samples were centrifuged at 15,000 rpm at 4°C for 15 min, and the soluble portion containing the nuclear extract was combined with the cytosolic fraction. Equal volumes of clarified and normalised cytosolic and nuclear extracts were incubated with 15–20 μl GFP-Trap Agarose (ChromoTek) for overnight at 4°C with rotation. Afterwards, beads were recovered by centrifugation, washed three times in washing buffer and eluted in 35 μl of 2× SDS–DTT sample for 5 min at 95°C.

### Live cell confocal microscopy

Cells were grown on 35 mm live cells imaging dishes (MatTek), transfected as required, and maintained in culture for 48 hr after transfection. Live cell microscopy was conducted using an inverted confocal laser scanning microscope (Zeiss LSM880) in a 37°C/5% vol/vol chamber with a ×64 oil immersion objective lens with 1 Airy unit pinhole using the 488 nm (GFP; mGL), 405 nm [4′,6-diamidino-2-phenylindole (DAPI)], and/or 594 nm (mCherry) lasers as appropriate. Co-localisation analysis between fluorescence channels (Pearson correlation coefficient) within individual cells was performed using Volocity software, version 6.3 (PerkinElmer).

### RNA isolation and RT

To extract total RNA, cells were treated with TRIzol Reagent (Invitrogen) for 10 min and then transferred to a new tube. Chloroform (200 µl) was added into each sample, vortexed for 1 min, and then centrifuged at 13,500 × *g*, at 4°C for 15 min. The upper clear phase was collected and mixed with equal volume of 70% ethanol. Afterward, PureLink RNA mini kit (Invitrogen) was used for washing and elution. RNA yield was assessed using NanoDrop, and 2 µg of RNA from each sample was evaluated for integrity via electrophoresis on a 1.2% agarose gel.

For RT-PCR, 2 µg of RNA was initially heated up at 70°C with RT buffer (Thermo Scientific; Cat #EP0441), 0.5 mM deoxynucleotide triphosphate (dNTPs) and 0.05 mM Oligo dT18 for 10 min. Subsequently, the reaction was supplemented with 0.5 µl RevertAid Reverse Transcriptase (Thermo Scientific) and 100 mM DTT, followed by incubation at 42°C for 90 min. The resulting cDNA product was diluted up to 1:4.

### PCR analysis of XBP1 mRNA splicing

XBP1s and XBP1u fragments were amplified from cDNA by PCR using primers flanking the splicing site identified by IRE1 with NEB Q5 High-Fidelity 2X Master Mix, as previously reported (Neidhardt et al., 2024). Briefly, XBP1u (255 bp) and XBP1s (229 bp) were separated in a 3% agarose gel by electrophoresis and stained with SYBR Safe nucleic acid gel stain (Invitrogen). Additionally, a XBP1^hybrid^ band (280 bp) was observed. The percentage of XBP1s was quantified by determining the band intensity with Fiji, v1.53c. For quantification, it was assumed that 50% of the XBP1^hybrid^ signal represented XBP1s, while the remaining 50% represented XBP1u.

### Immunoprecipitation and FURIN cleavage assay

To investigate whether ATF6α is directly cleaved by FURIN, GFP-ATF6α_LD^S1P,S2Pmut^ (UK3122) construct was expressed and purified from an *FURIN∆* clone (#2.2.G). Equal volumes of clarified and normalised cell lysates were incubated with 15–20 μl GFP-Trap Agarose (ChromoTek) for overnight at 4°C with rotation. Afterwards, beads were recovered by centrifugation, washed three times in washing buffer and then incubated with 75 μl of FURIN cleavage buffer (20 mM 4-(2-hydroxyethyl)-1-piperazineethanesulfonic acid (HEPES), 1 mM CaCl_2_, 0.2 mM β-mercaptoethanol, 0.1% Triton X-100; pH 7.5) supplemented with 1 U FURIN (#P8077S, NEB) for 6 hr at 25°C. As a negative control, calcium-depleted FURIN buffer was used. Additionally, an FURIN control substrate, MBP5-FN-paramyosin-ΔSal (#E8052S, NEB) containing an FURIN site, was included in parallel reactions and incubated for 6 hr at 25°C. The eluted protein was then analysed either via immunoblot or Coomassie blue staining.

### Analysis of ATF6α redox status

Both *2K-*parental cells and *2K-CRT*∆ derivative cells were cultured in 10-cm dishes for 2 days and then treated with 2DG (4 mM) for 3 hr before harvested. Following treatment, cells were harvested in PBS containing 1 mM EDTA and 20 mM NEM and lysed in Nonidet-lysis buffer supplemented with 20 mM NEM. All subsequent buffers were also supplemented with 20 mM NEM. Equal volumes of clarified and normalised cell lysates were incubated with 15–20 μl GFP-Trap Agarose (ChromoTek) for overnight at 4°C with rotation. Afterwards, beads were recovered by centrifugation, washed three times in washing buffer/NEM and eluted in SDS/NEM loading buffer. After heating at 95°C, eluted proteins were split into two equal samples and separated under reducing (+50 mM DTT) or non-reducing SDS/PAGE, followed by immunoblot.

### Statistics

Data groups were analysed as described in the figure legends using GraphPad Prism10 software. Differences between groups were considered statistically significant if *p < 0.05; **p < 0.01; ***p < 0.001; and ****p < 0.0001. All error bars represent mean ± standard error of the mean.

## Data Availability

All data generated or analysed during this study are included in the manuscript, supporting files, or are submitted to public databases. The raw and processed high-throughput sequencing data reported in this paper have been uploaded in NCBI's Gene Expression Omnibus (GEO, accession number: GSE254745). The following dataset was generated: OrdoñezA HardingPH RonD 2024A genome wide CRISPR/Cas9 screen identifies calreticulin as a selective repressor of ATF6⍺NCBI Gene Expression OmnibusGSE25474510.7554/eLife.96979PMC1128626639073063

## Additional files

Supplementary file 1. Ranking of genes enriched in the *activator and repressor ATF6*α *screens*. Genes are ranked by ‘pos | rank’ value. Notably, *MBTPS1*, encoding for S1P protease, is on the top of positively selected genes in the *activator screen*, while *SETDB1* (SET Domain Bifurcated Histone Lysine Methyltransferase 1) is on the top position among the positively selected genes in the *repressor screen*. The top 100 positively selected genes correspond to Figure 1C, D.

Transparent reporting form
