# Supplementary figures and images for: A genome-wide CRISPR/Cas9 screen identifies calreticulin as a selective repressor of ATF6α (part 1 of 2)
Source: eLife. 2024 Jul 29;13:RP96979. doi: 10.7554/eLife.96979 (PMC11286266; doi:10.7554/eLife.96979)

03.05.2024 Uncropped and labelled blots for figure 1, supplement 1

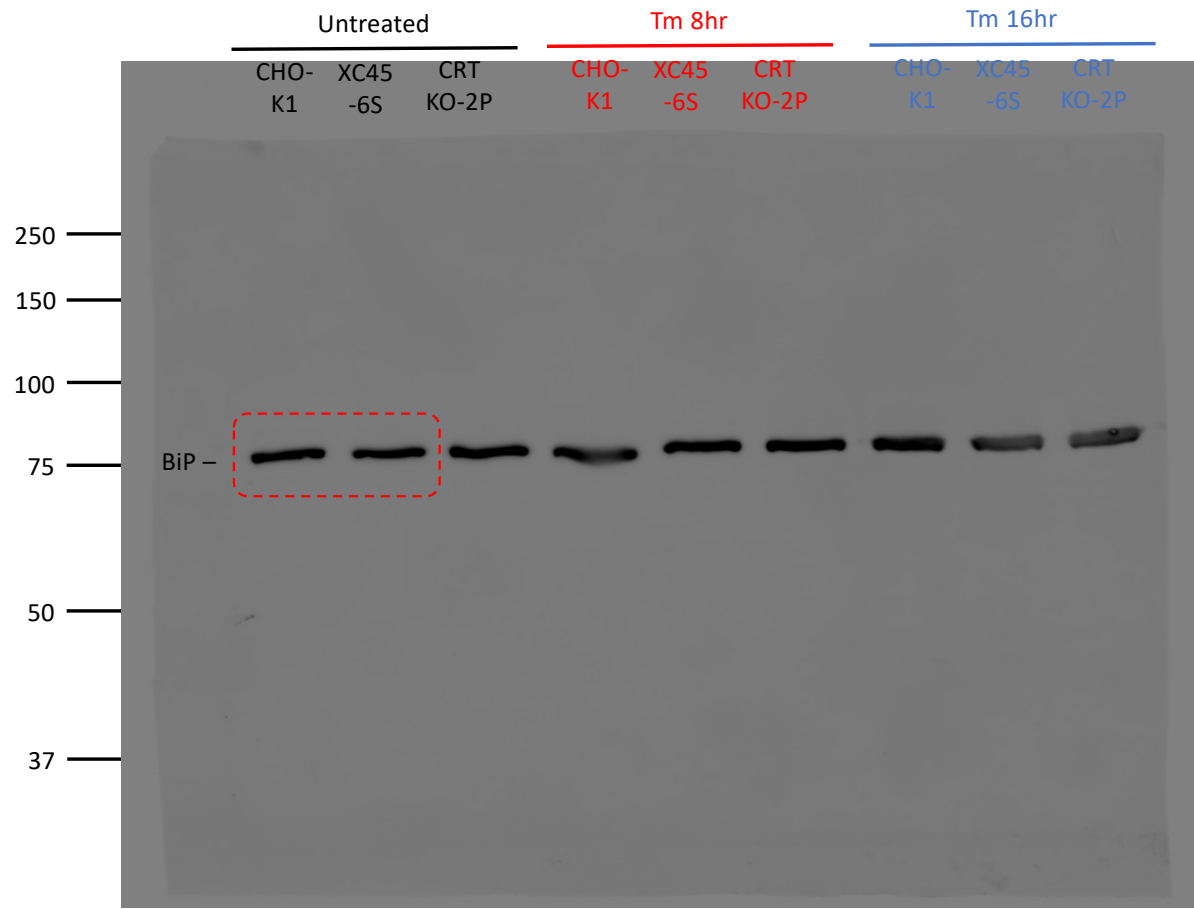

03.05.24 BiP Chicken, IR800

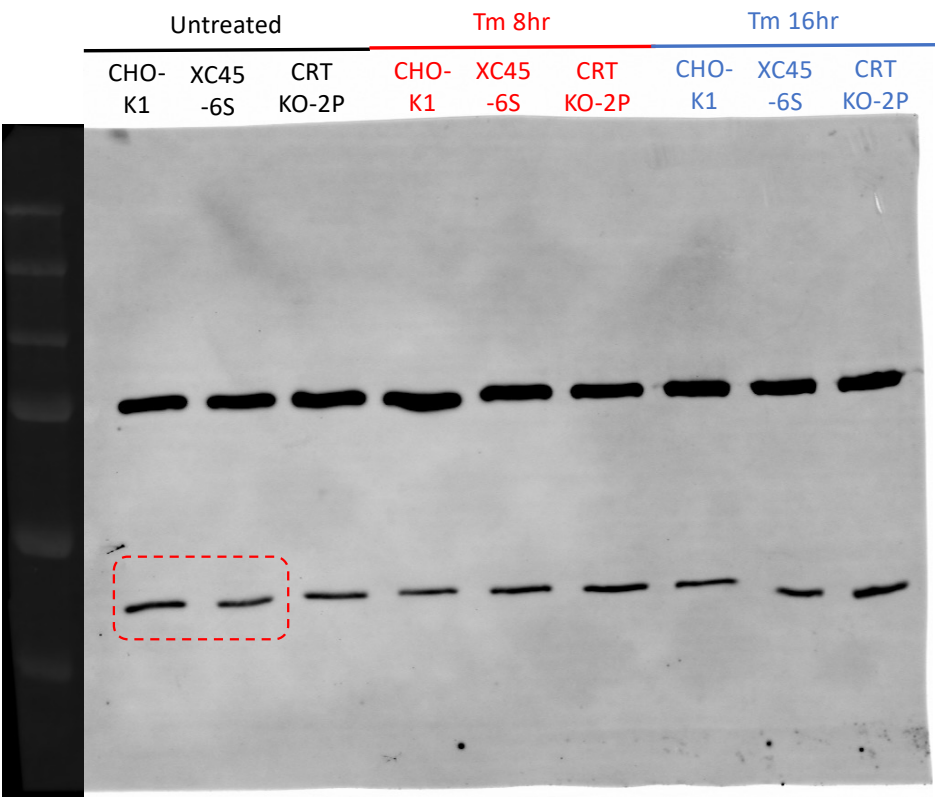

07.05.24 Actin Mouse, IR800

Supplement: Figure 1—figure supplement 1—source data 1. [file elife-96979-fig1-figsupp1-data1.zip › Figure 1_ figure supplement 1_source data/Uncrooped and labeled blots for Figure 1_figure supplement 1.pdf]

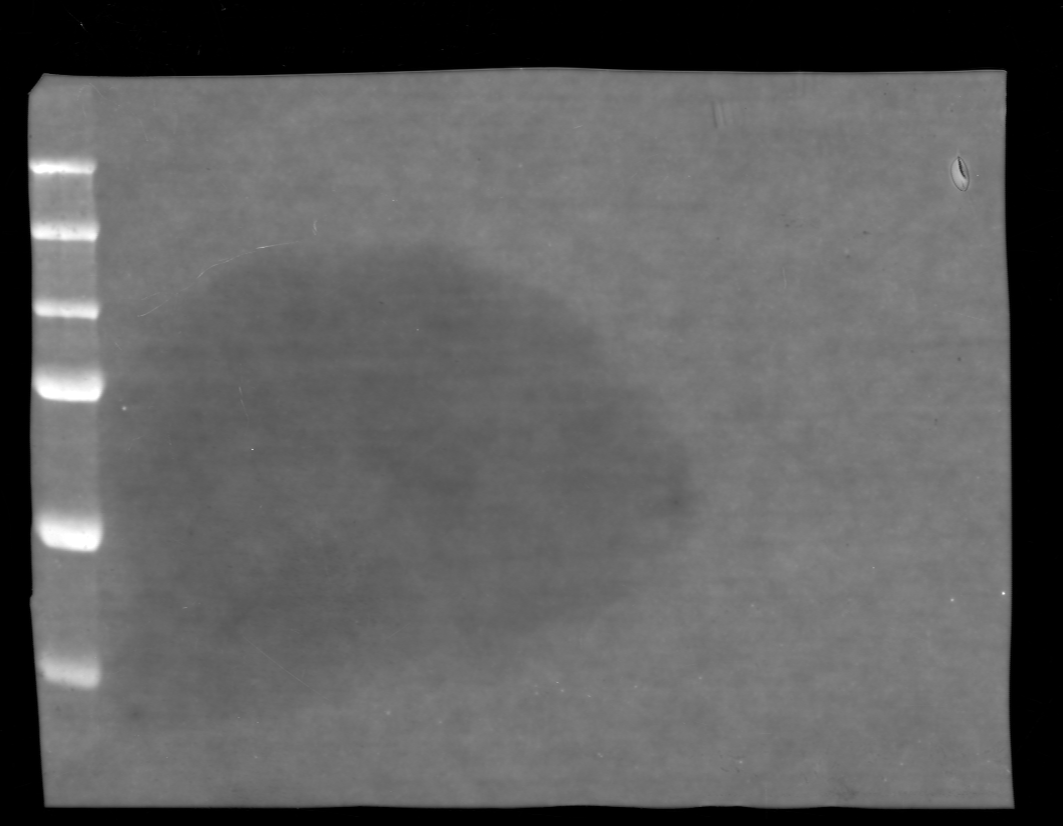

Supplement: Figure 1—figure supplement 1—source data 1. [file elife-96979-fig1-figsupp1-data1.zip › Figure 1_ figure supplement 1_source data/Raw unedited gels for (Figure 1_figure supplement 1)/Anti-BiP/700.TIF]

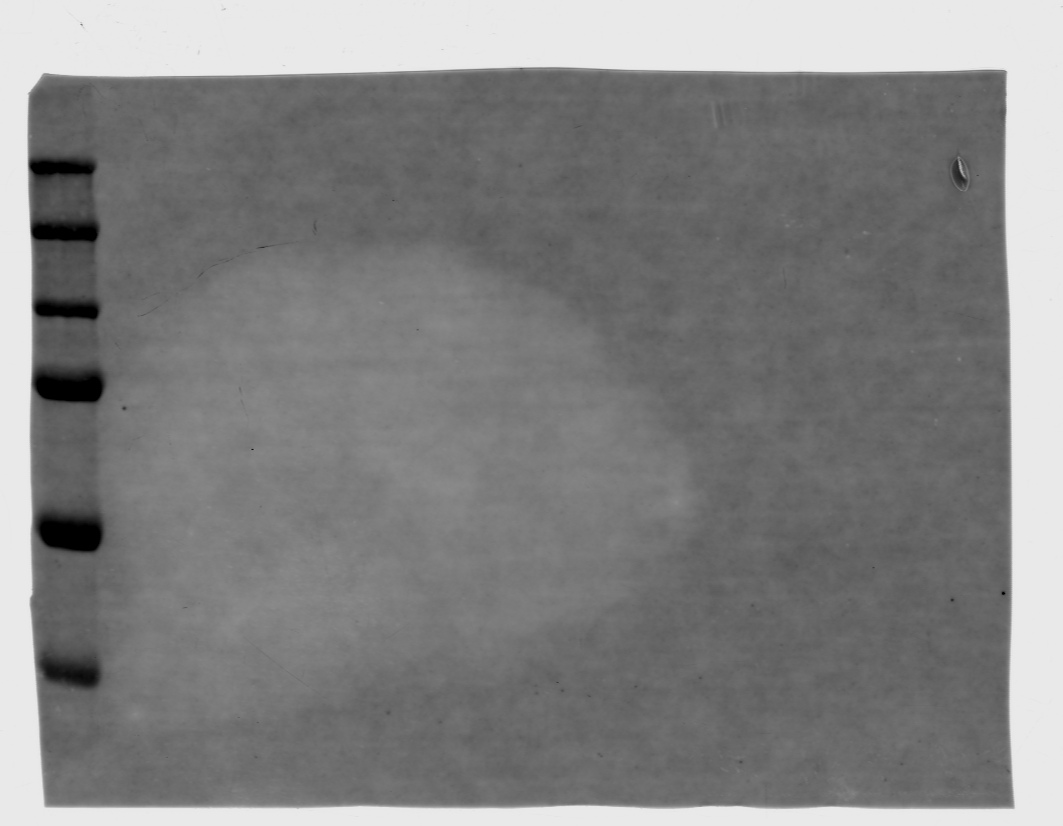

Supplement: Figure 1—figure supplement 1—source data 1. [file elife-96979-fig1-figsupp1-data1.zip › Figure 1_ figure supplement 1_source data/Raw unedited gels for (Figure 1_figure supplement 1)/Anti-BiP/700MODIIFED.tif]

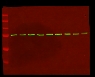

Supplement: Figure 1—figure supplement 1—source data 1. [file elife-96979-fig1-figsupp1-data1.zip › Figure 1_ figure supplement 1_source data/Raw unedited gels for (Figure 1_figure supplement 1)/Anti-BiP/2024-05-03-134440_1_TH.jpg]

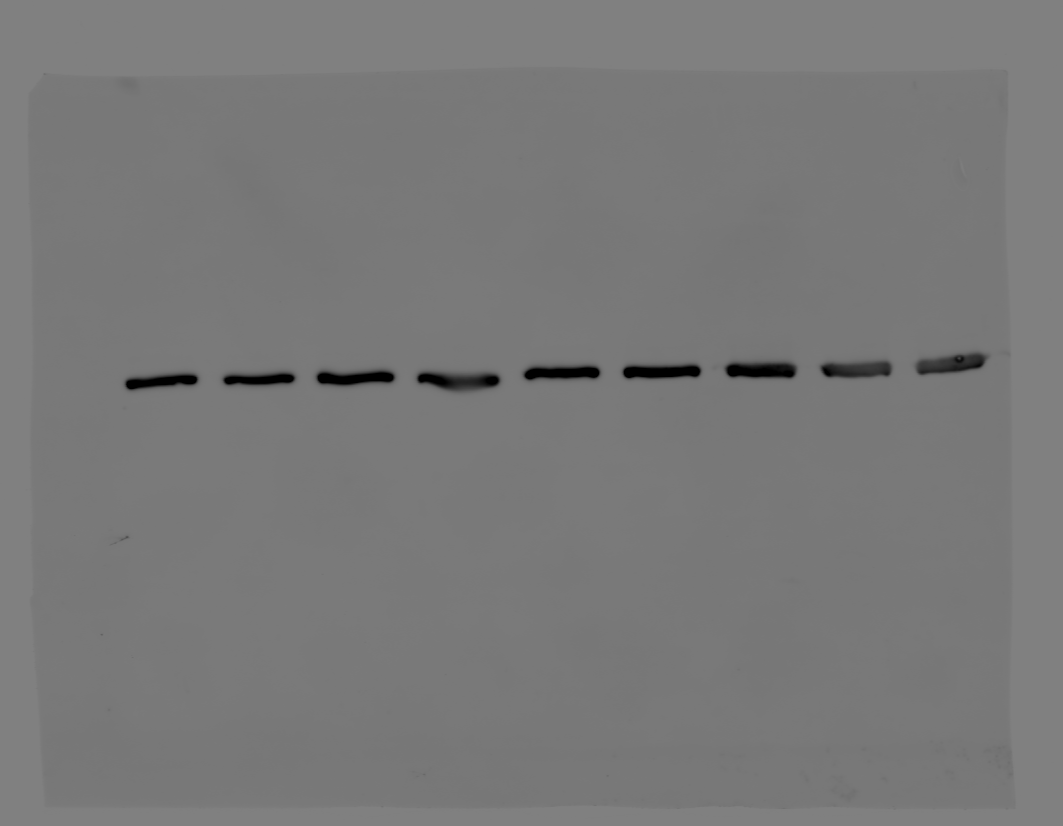

Supplement: Figure 1—figure supplement 1—source data 1. [file elife-96979-fig1-figsupp1-data1.zip › Figure 1_ figure supplement 1_source data/Raw unedited gels for (Figure 1_figure supplement 1)/Anti-BiP/800MODIFIED.tif]

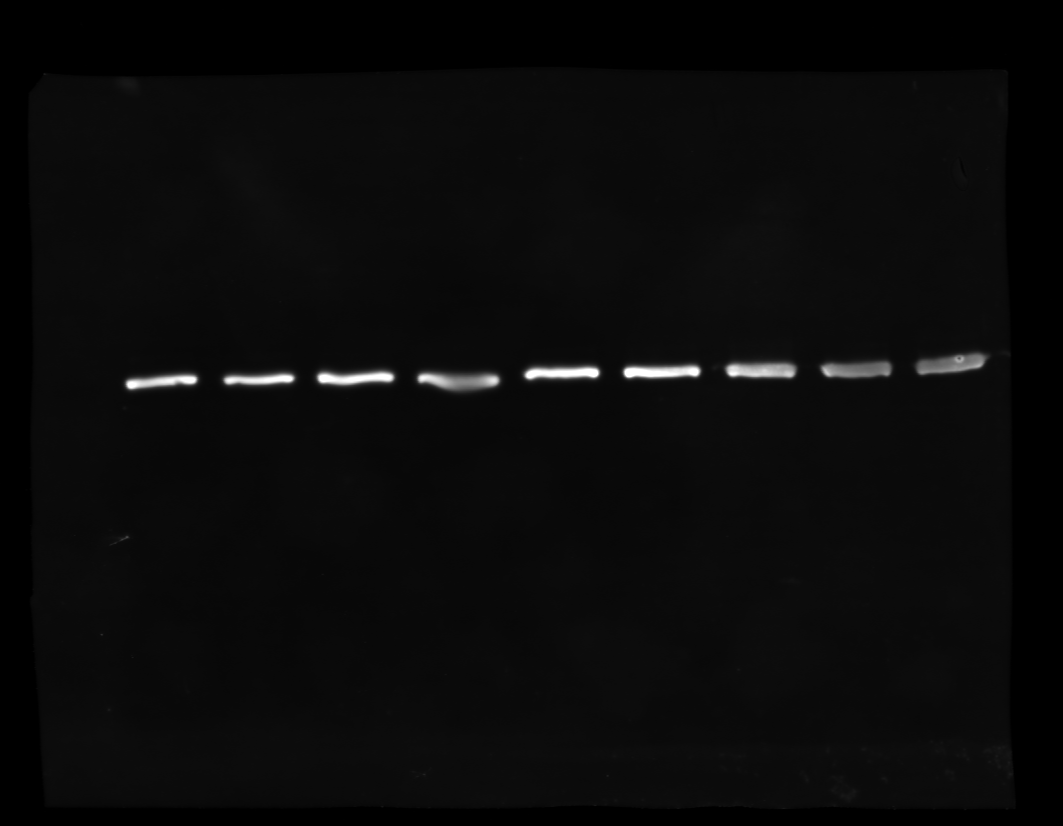

Supplement: Figure 1—figure supplement 1—source data 1. [file elife-96979-fig1-figsupp1-data1.zip › Figure 1_ figure supplement 1_source data/Raw unedited gels for (Figure 1_figure supplement 1)/Anti-BiP/800.TIF]

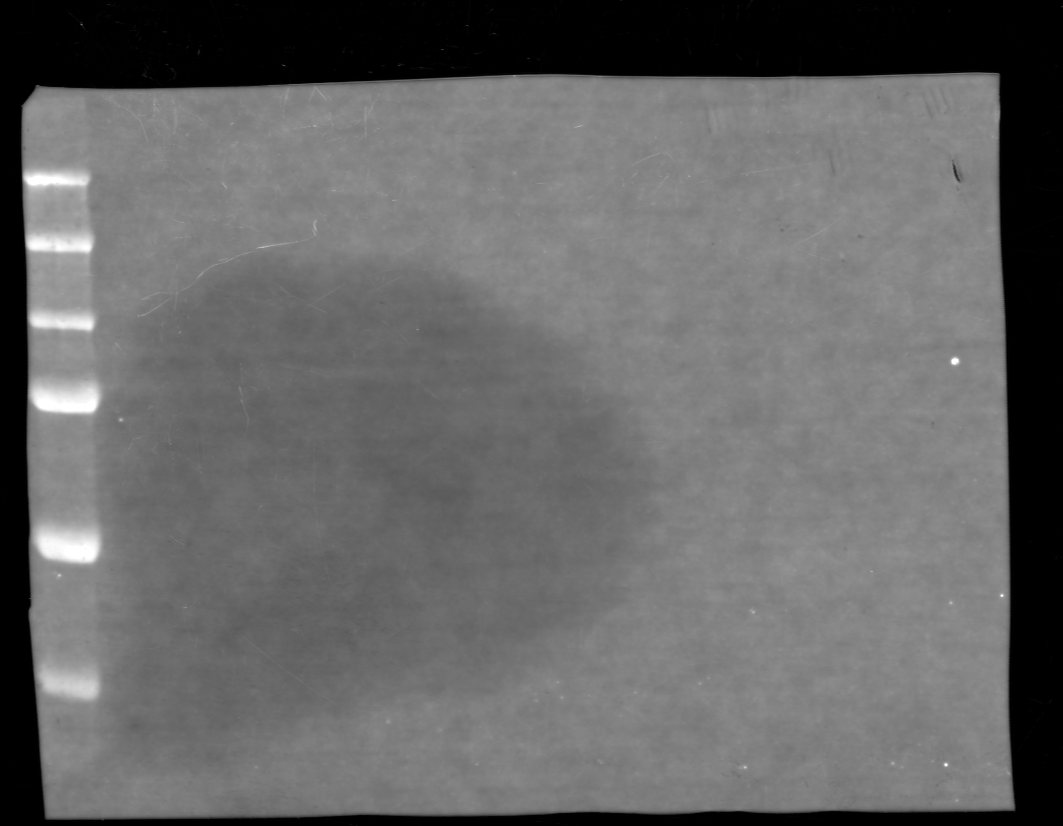

Supplement: Figure 1—figure supplement 1—source data 1. [file elife-96979-fig1-figsupp1-data1.zip › Figure 1_ figure supplement 1_source data/Raw unedited gels for (Figure 1_figure supplement 1)/Anti-Actin/2024-05-07-150822/700.TIF]

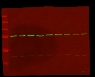

Supplement: Figure 1—figure supplement 1—source data 1. [file elife-96979-fig1-figsupp1-data1.zip › Figure 1_ figure supplement 1_source data/Raw unedited gels for (Figure 1_figure supplement 1)/Anti-Actin/2024-05-07-150822/2024-05-07-150822_1_TH.jpg]

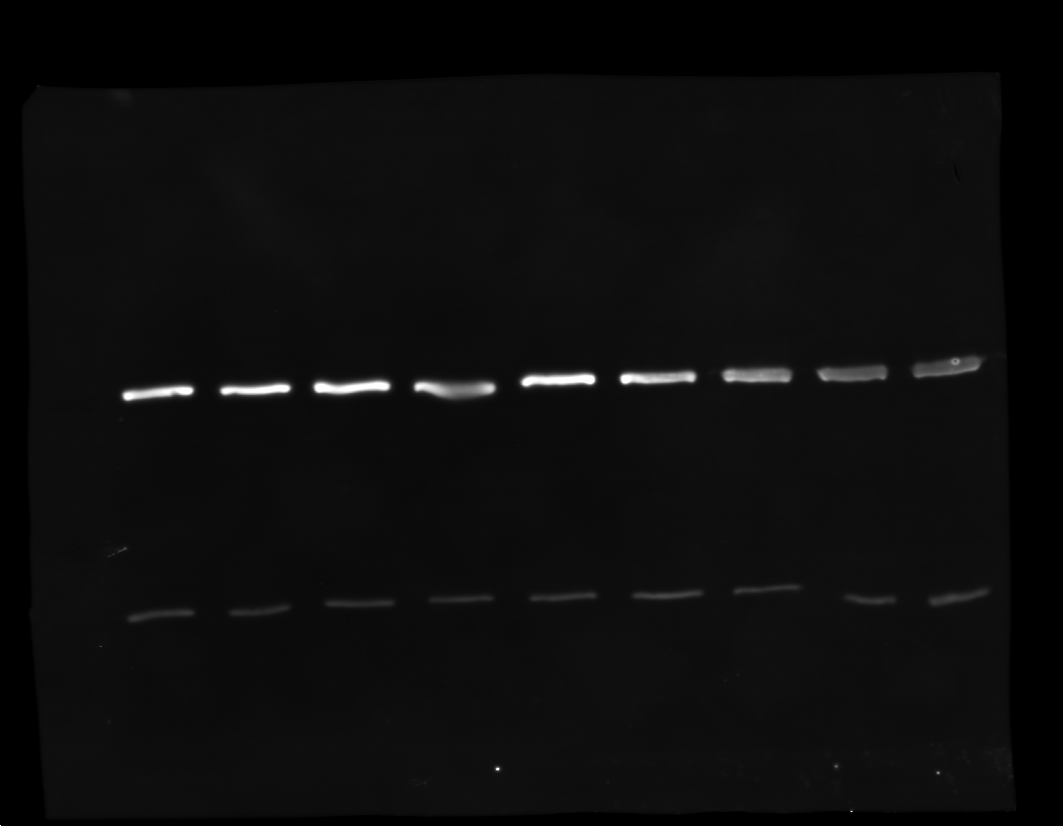

Supplement: Figure 1—figure supplement 1—source data 1. [file elife-96979-fig1-figsupp1-data1.zip › Figure 1_ figure supplement 1_source data/Raw unedited gels for (Figure 1_figure supplement 1)/Anti-Actin/2024-05-07-150822/800.TIF]

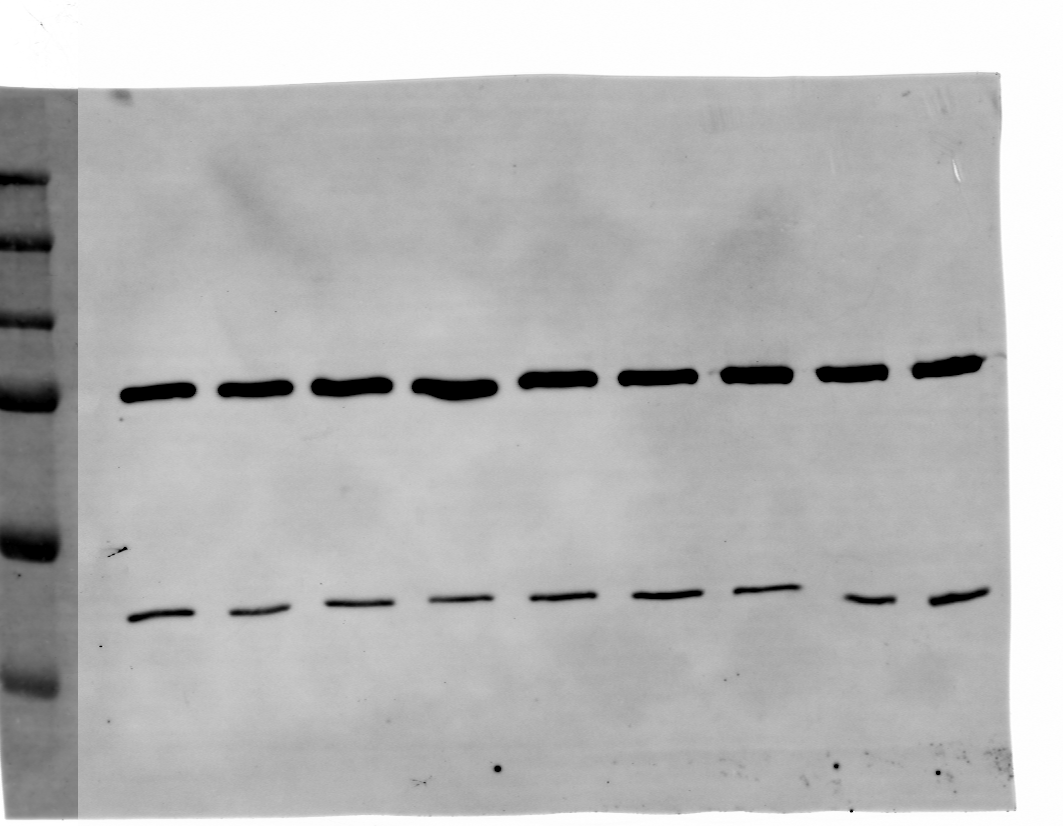

Supplement: Figure 1—figure supplement 1—source data 1. [file elife-96979-fig1-figsupp1-data1.zip › Figure 1_ figure supplement 1_source data/Raw unedited gels for (Figure 1_figure supplement 1)/Anti-Actin/2024-05-07-150822/800_modified.tif]

Uncropped and labelled gels for (Figure 1-figure supplement 4)

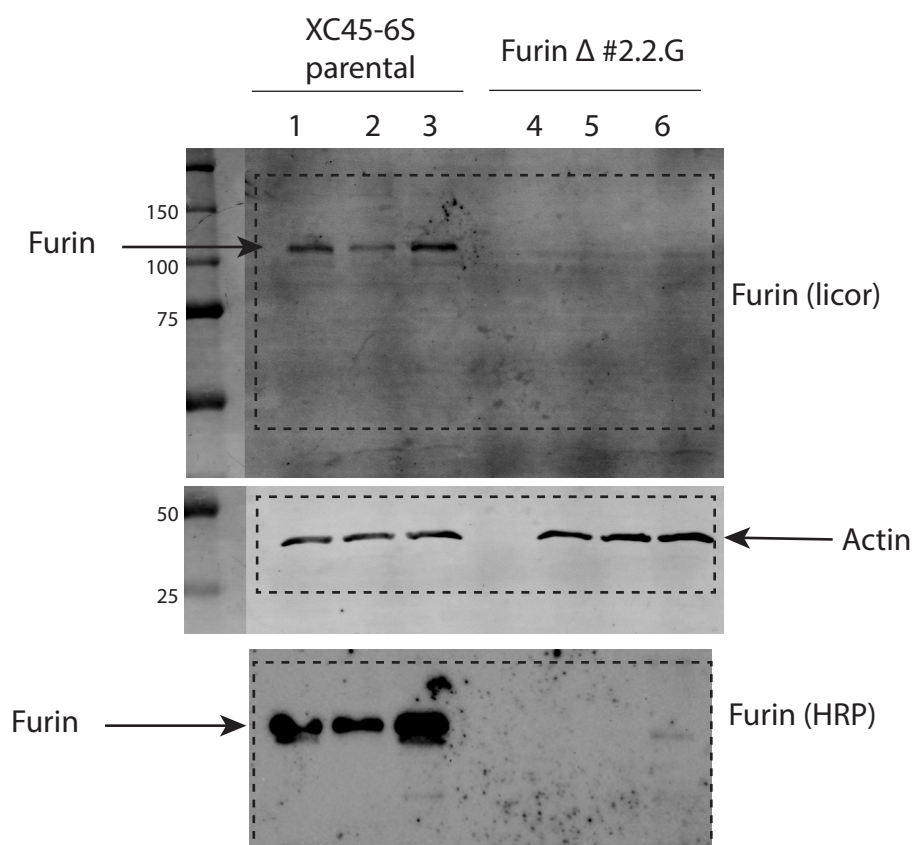

Supplement: Figure 1—figure supplement 4—source data 1. [file elife-96979-fig1-figsupp4-data1.zip › Figure 1_figure supplement 4_source data/Uncropped and labelled gels for (Figure 1-figure supplement 4).pdf]

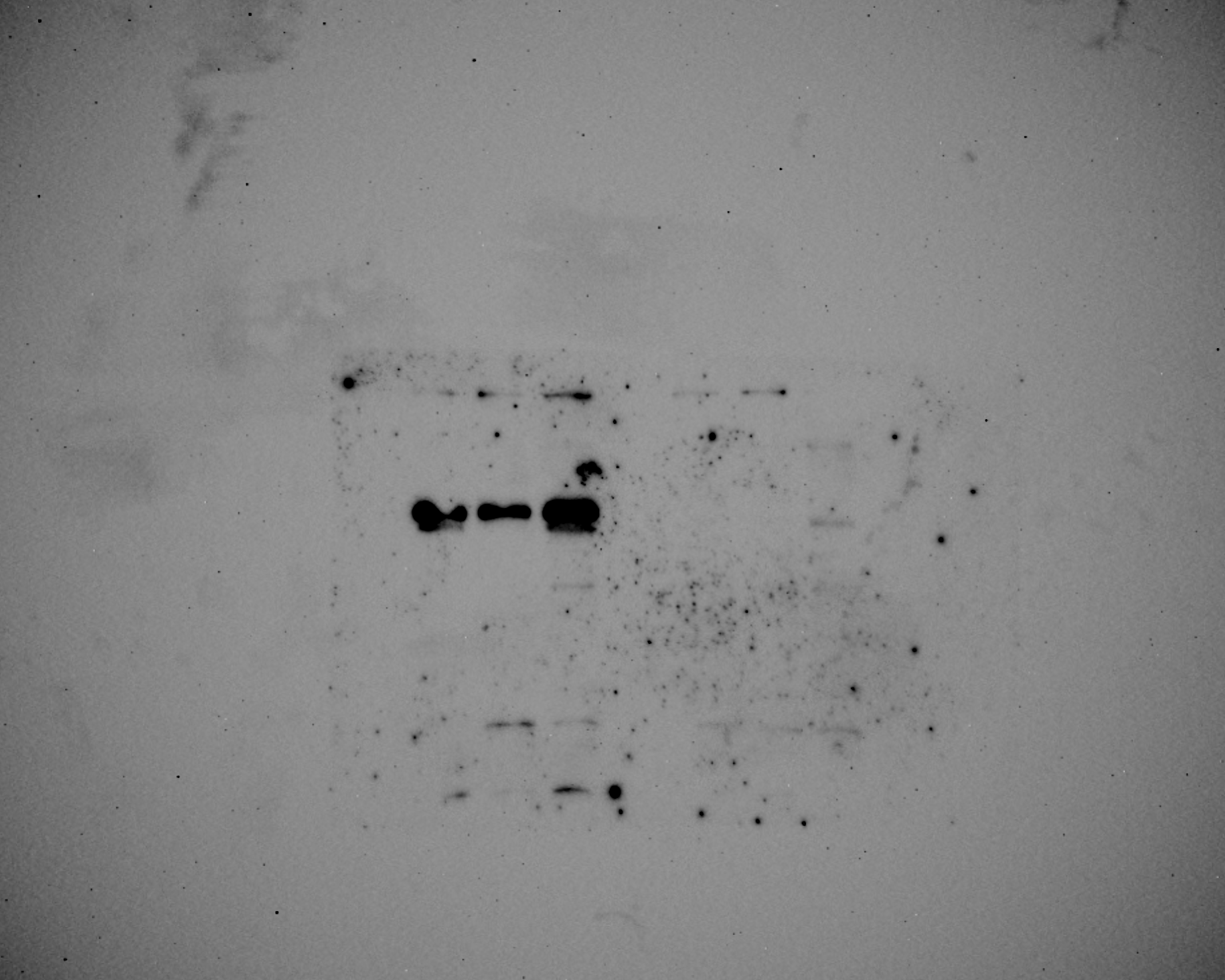

Supplement: Figure 1—figure supplement 4—source data 1. [file elife-96979-fig1-figsupp4-data1.zip › Figure 1_figure supplement 4_source data/Raw unedited gels for (Figure 1-figure supplement 4)/Furin HRP/aog 2022-06-28 13h52m07s(Chemiluminescence).tif]

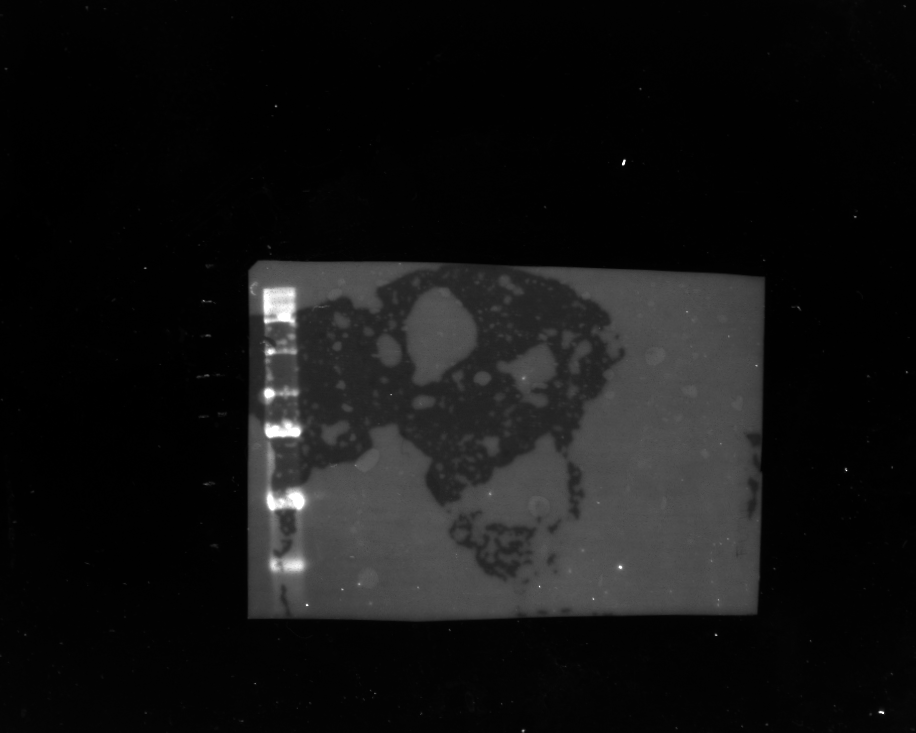

Supplement: Figure 1—figure supplement 4—source data 1. [file elife-96979-fig1-figsupp4-data1.zip › Figure 1_figure supplement 4_source data/Raw unedited gels for (Figure 1-figure supplement 4)/Furin HRP/aog 2022-06-28 13h53m50s(Coomassie Blue).raw16.tif]

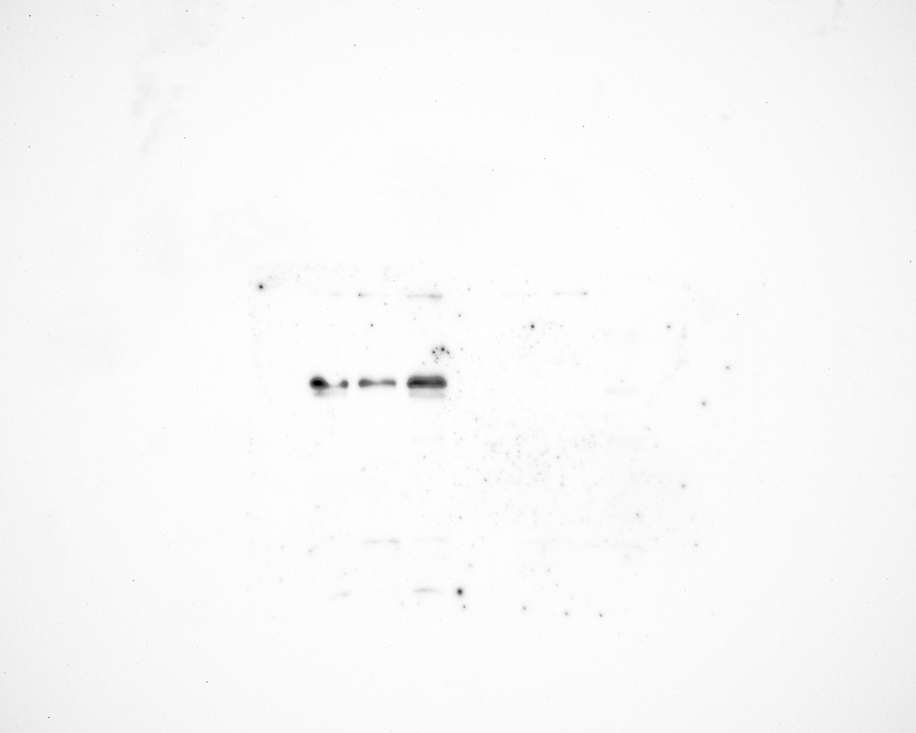

Supplement: Figure 1—figure supplement 4—source data 1. [file elife-96979-fig1-figsupp4-data1.zip › Figure 1_figure supplement 4_source data/Raw unedited gels for (Figure 1-figure supplement 4)/Furin HRP/aog 2022-06-28 13h52m07s(Chemiluminescence).raw16.tif]

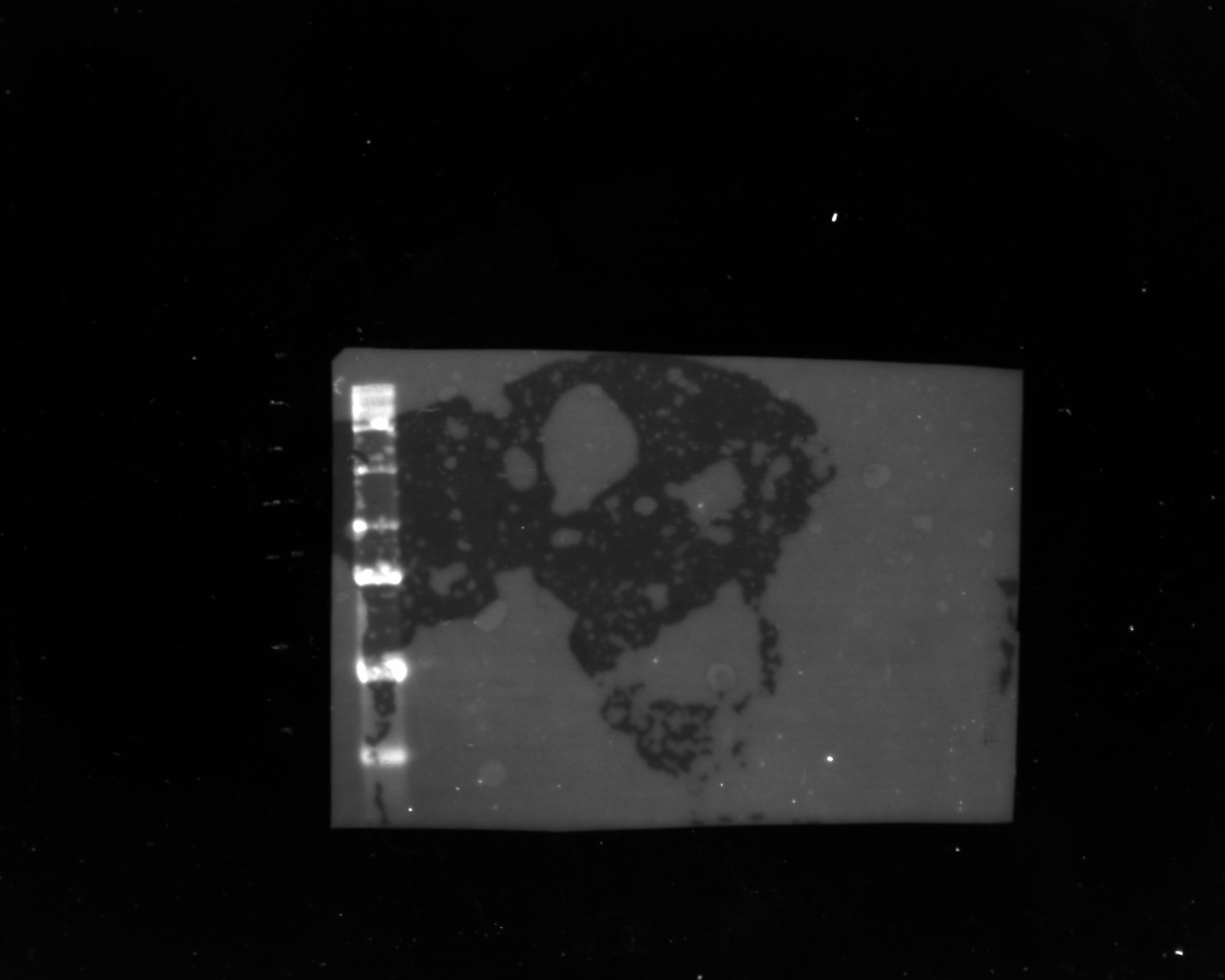

Supplement: Figure 1—figure supplement 4—source data 1. [file elife-96979-fig1-figsupp4-data1.zip › Figure 1_figure supplement 4_source data/Raw unedited gels for (Figure 1-figure supplement 4)/Furin HRP/aog 2022-06-28 13h53m50s(Coomassie Blue).tif]

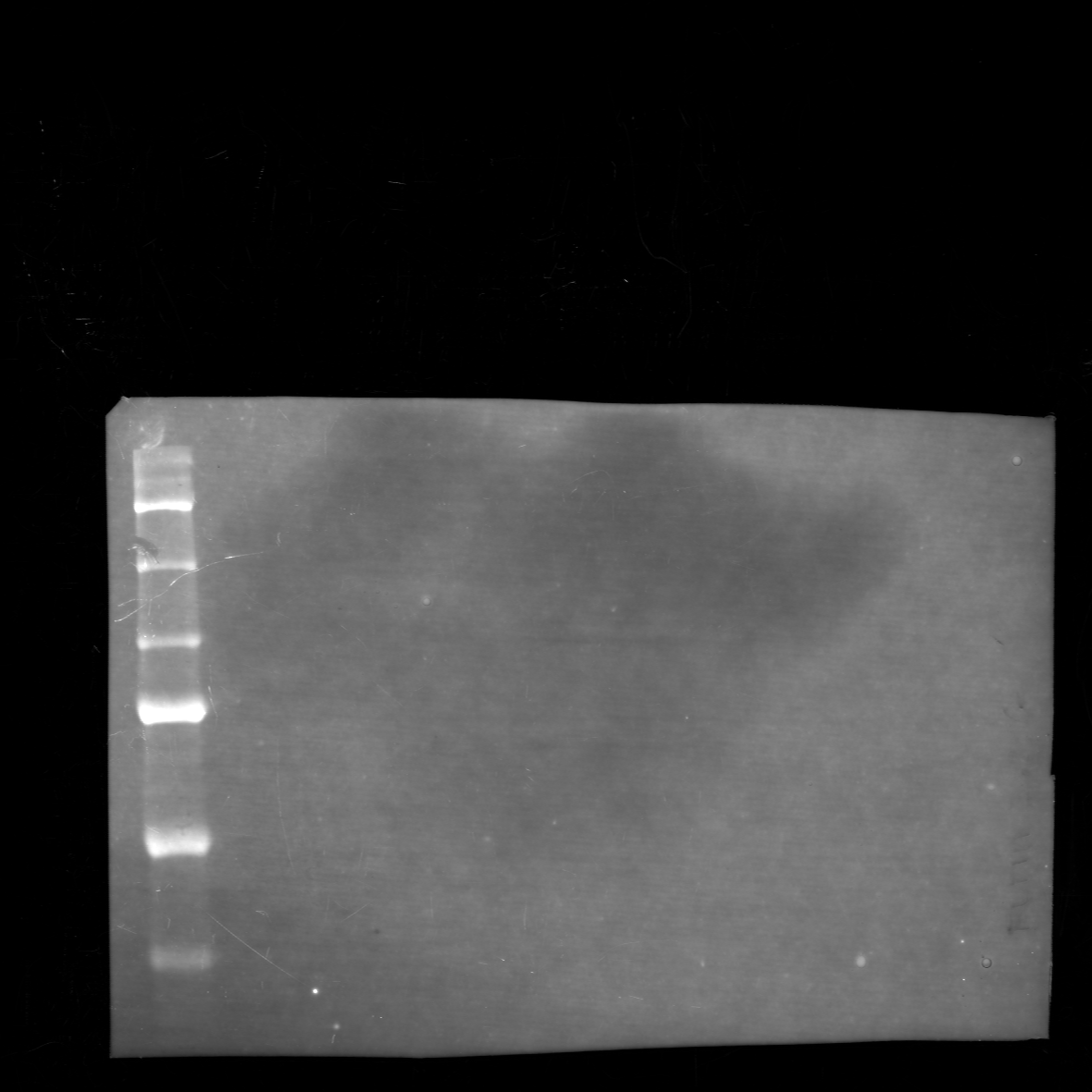

Supplement: Figure 1—figure supplement 4—source data 1. [file elife-96979-fig1-figsupp4-data1.zip › Figure 1_figure supplement 4_source data/Raw unedited gels for (Figure 1-figure supplement 4)/Furin Licor/2022-06-29-143422/700.TIF]

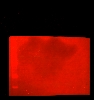

Supplement: Figure 1—figure supplement 4—source data 1. [file elife-96979-fig1-figsupp4-data1.zip › Figure 1_figure supplement 4_source data/Raw unedited gels for (Figure 1-figure supplement 4)/Furin Licor/2022-06-29-143422/2022-06-29-143422_1_TH.jpg]

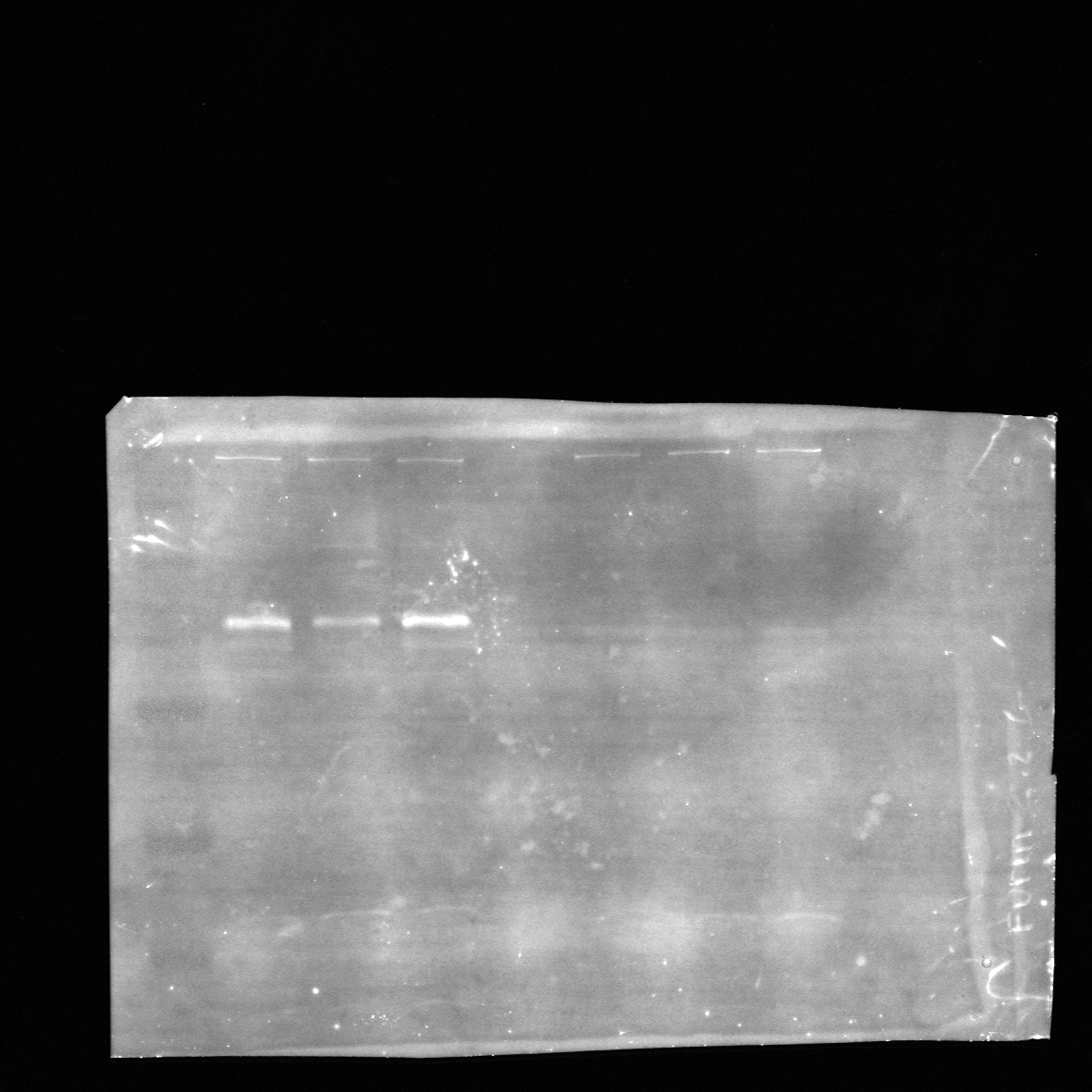

Supplement: Figure 1—figure supplement 4—source data 1. [file elife-96979-fig1-figsupp4-data1.zip › Figure 1_figure supplement 4_source data/Raw unedited gels for (Figure 1-figure supplement 4)/Furin Licor/2022-06-29-143422/800.TIF]

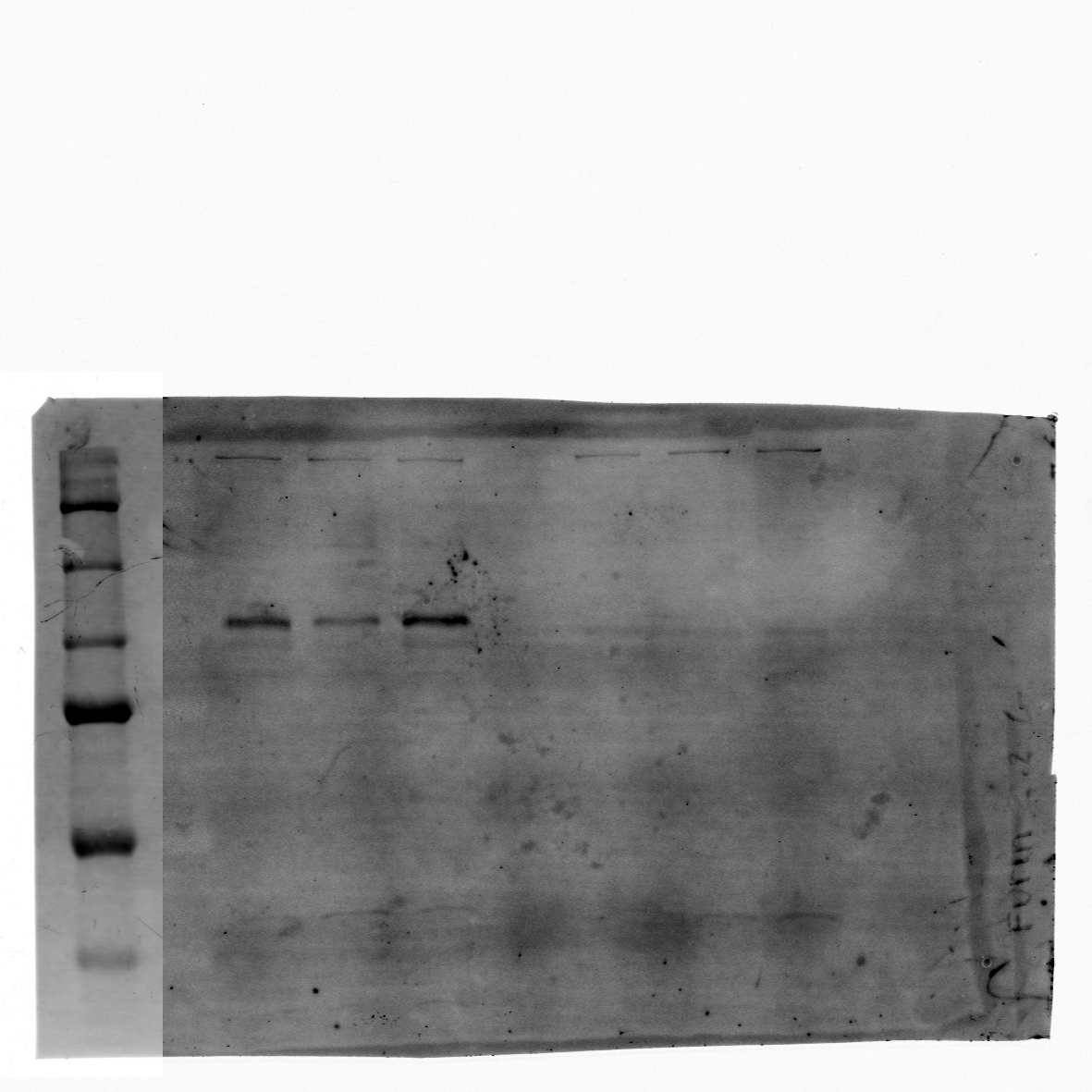

Supplement: Figure 1—figure supplement 4—source data 1. [file elife-96979-fig1-figsupp4-data1.zip › Figure 1_figure supplement 4_source data/Raw unedited gels for (Figure 1-figure supplement 4)/Furin Licor/2022-06-29-143422/800_modified.tif]

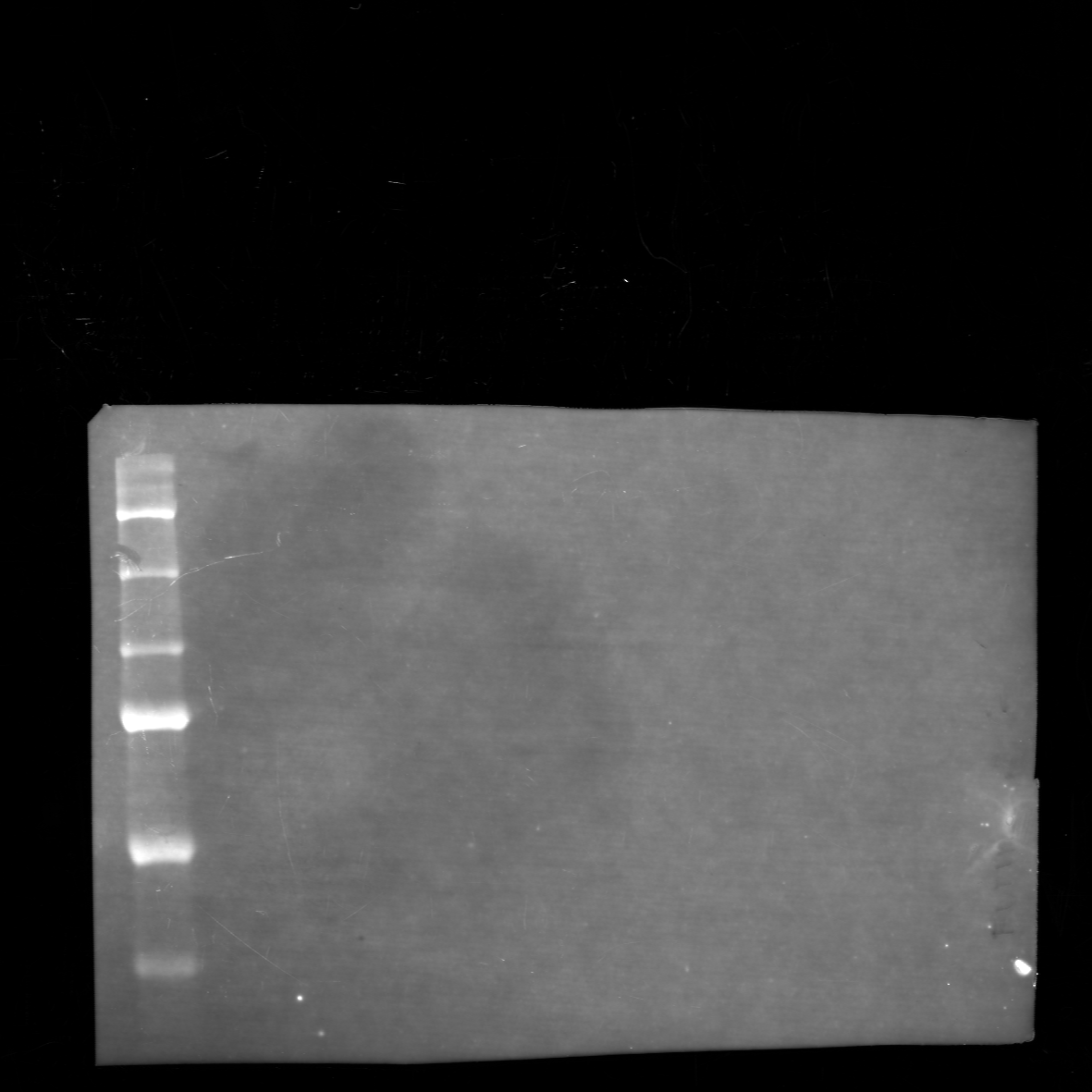

Supplement: Figure 1—figure supplement 4—source data 1. [file elife-96979-fig1-figsupp4-data1.zip › Figure 1_figure supplement 4_source data/Raw unedited gels for (Figure 1-figure supplement 4)/Actin/2022-06-30-135857/700.TIF]

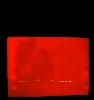

Supplement: Figure 1—figure supplement 4—source data 1. [file elife-96979-fig1-figsupp4-data1.zip › Figure 1_figure supplement 4_source data/Raw unedited gels for (Figure 1-figure supplement 4)/Actin/2022-06-30-135857/2022-06-30-135857_1_TH.jpg]

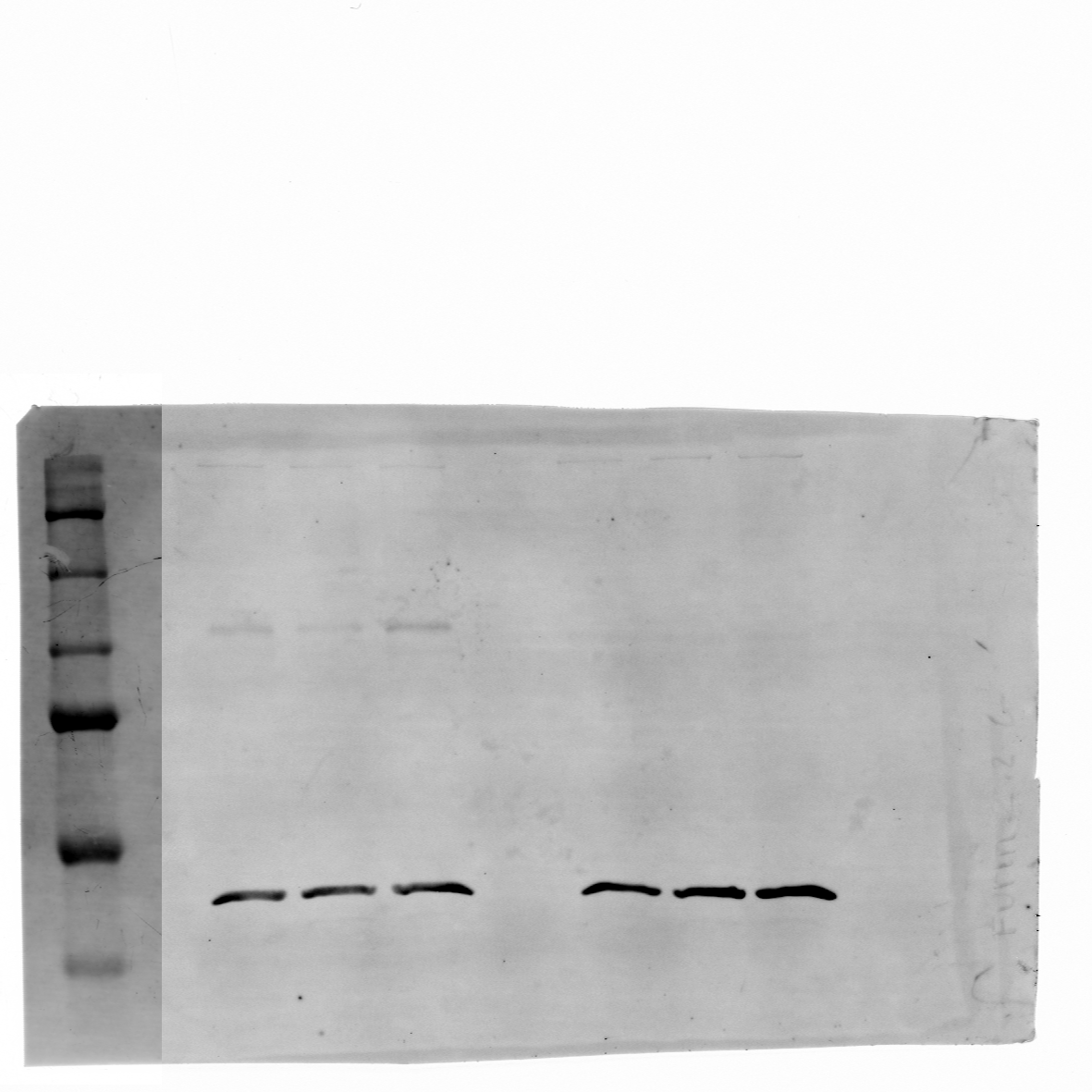

Supplement: Figure 1—figure supplement 4—source data 1. [file elife-96979-fig1-figsupp4-data1.zip › Figure 1_figure supplement 4_source data/Raw unedited gels for (Figure 1-figure supplement 4)/Actin/2022-06-30-135857/800_modified2.tif]

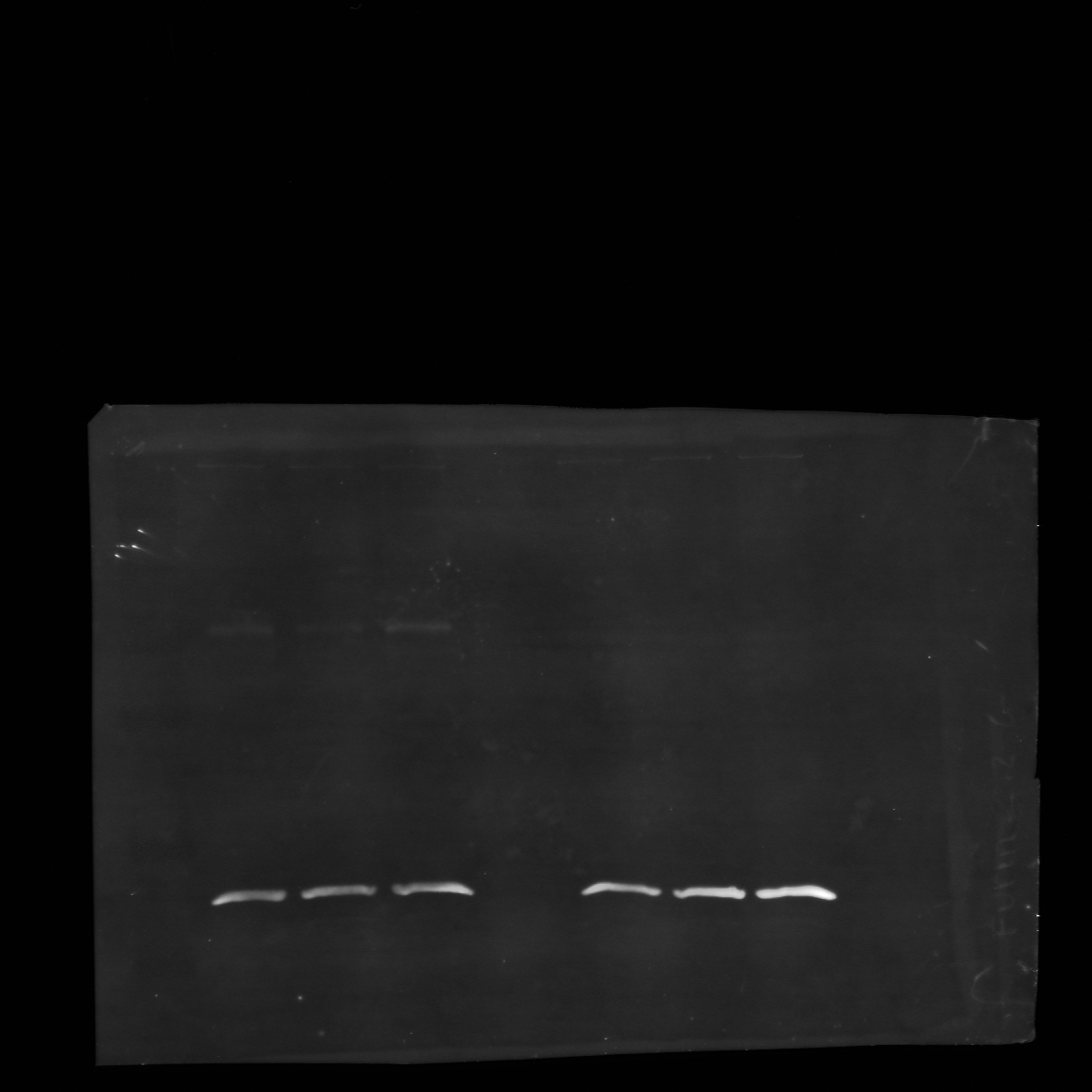

Supplement: Figure 1—figure supplement 4—source data 1. [file elife-96979-fig1-figsupp4-data1.zip › Figure 1_figure supplement 4_source data/Raw unedited gels for (Figure 1-figure supplement 4)/Actin/2022-06-30-135857/800.TIF]

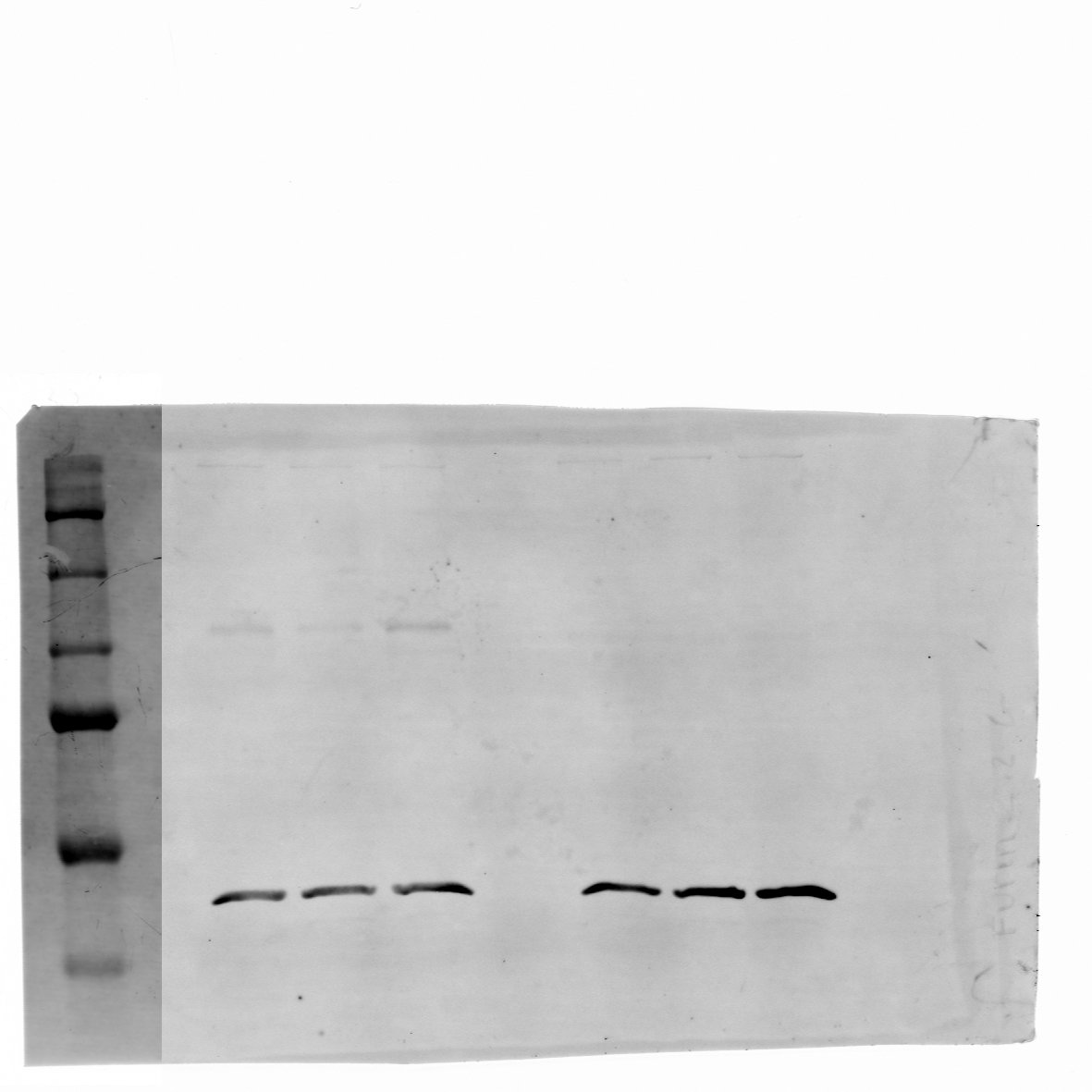

Supplement: Figure 1—figure supplement 4—source data 1. [file elife-96979-fig1-figsupp4-data1.zip › Figure 1_figure supplement 4_source data/Raw unedited gels for (Figure 1-figure supplement 4)/Actin/2022-06-30-135857/800_modified.tif]

Uncropped and labelled gels for (Figure 2)

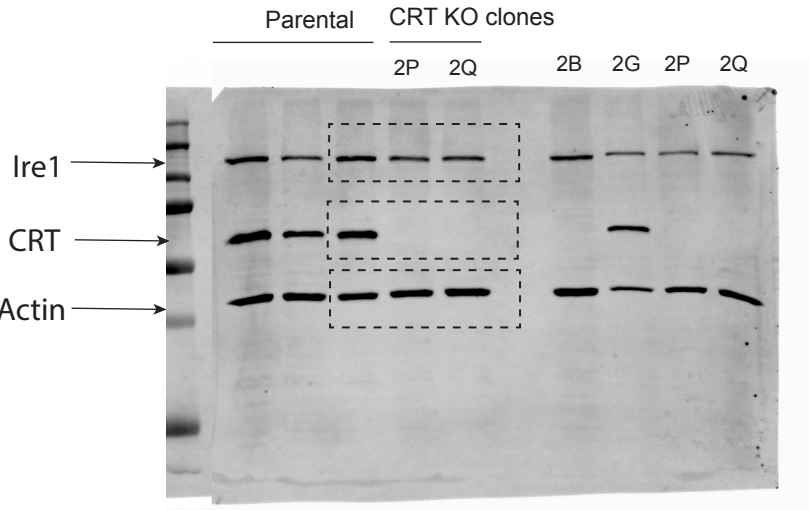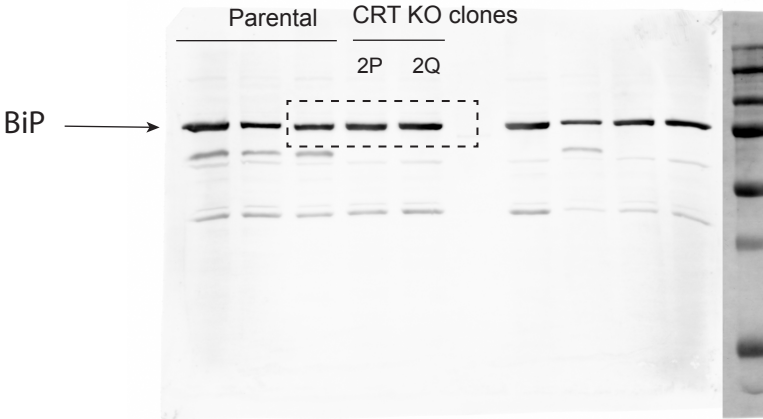

Supplement: Figure 2—source data 1. [file elife-96979-fig2-data1.zip › Figure 2_ source data/Uncropped and labelled gels for (Figure 2).pdf]

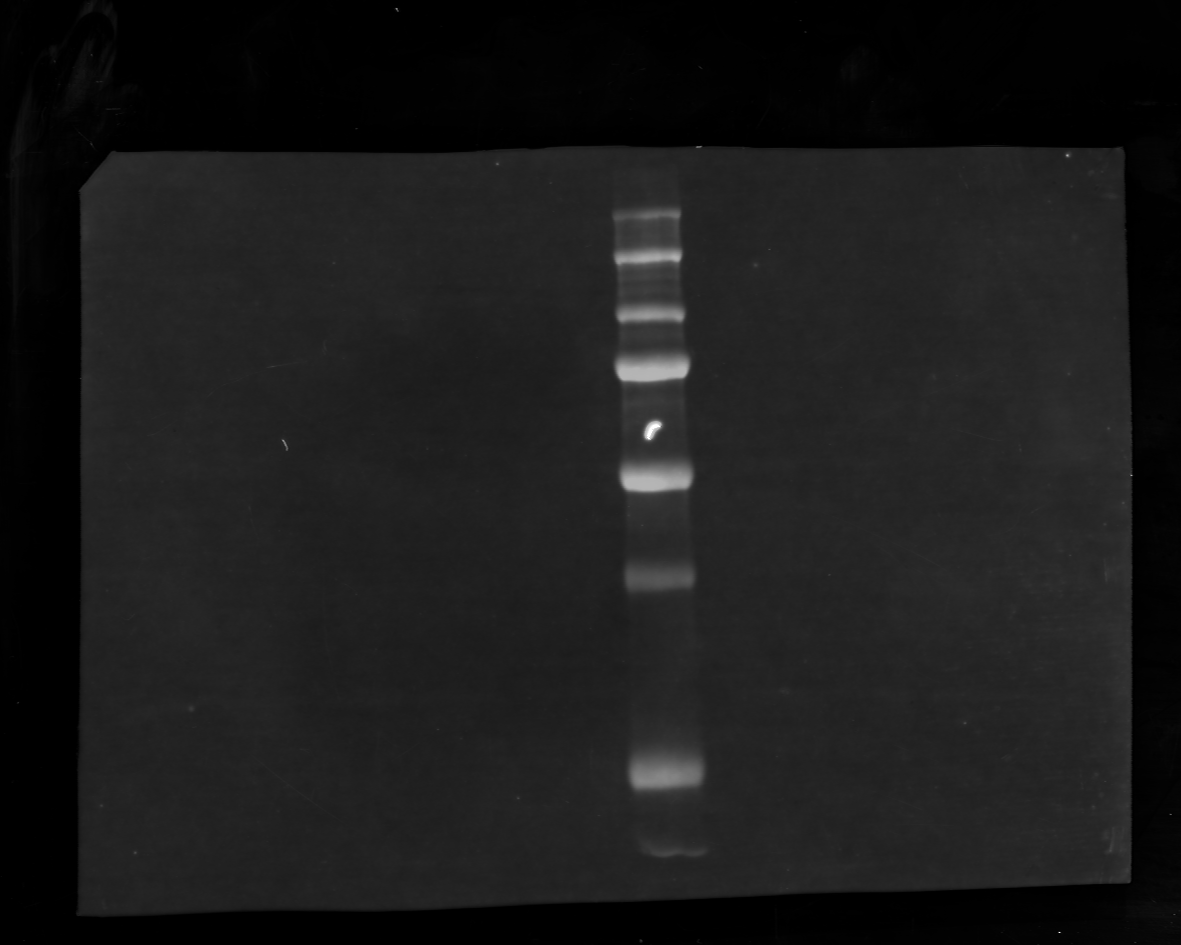

Supplement: Figure 2—source data 1. [file elife-96979-fig2-data1.zip › Figure 2_ source data/Raw unedited gels for (Figure 2)/Anti-Calreticulin/2023-04-28-155909/700.TIF]

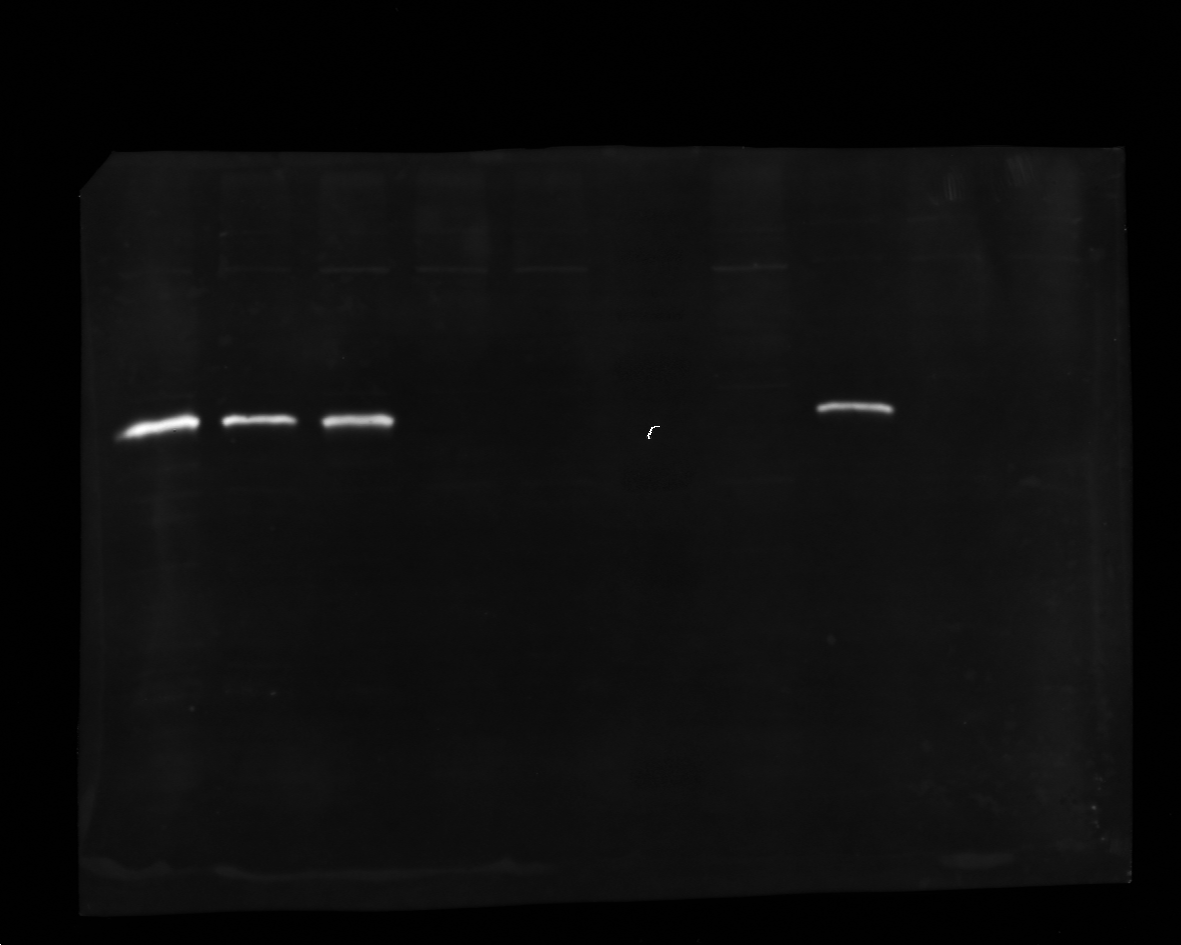

Supplement: Figure 2—source data 1. [file elife-96979-fig2-data1.zip › Figure 2_ source data/Raw unedited gels for (Figure 2)/Anti-Calreticulin/2023-04-28-155909/800.TIF]

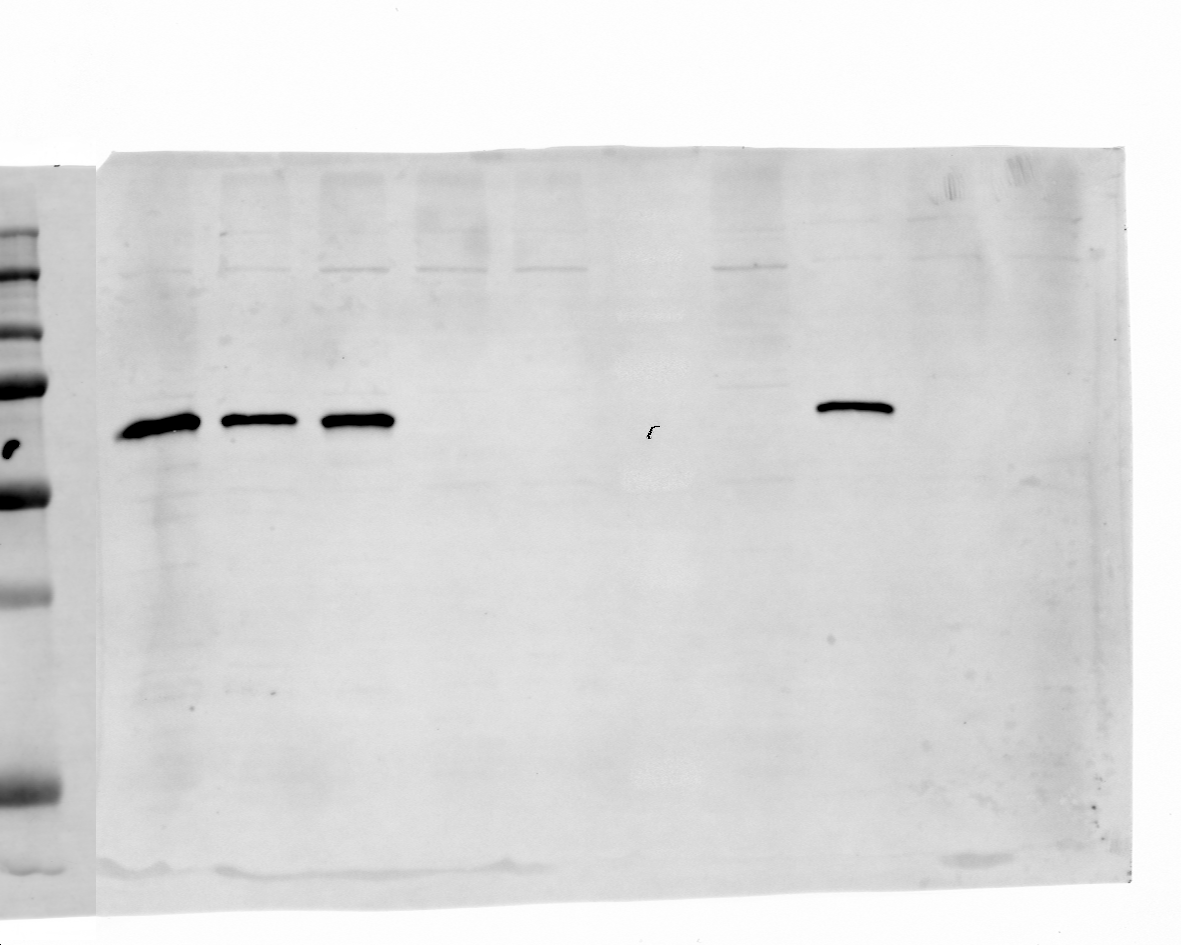

Supplement: Figure 2—source data 1. [file elife-96979-fig2-data1.zip › Figure 2_ source data/Raw unedited gels for (Figure 2)/Anti-Calreticulin/2023-04-28-155909/800_modified.tif]

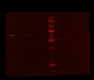

Supplement: Figure 2—source data 1. [file elife-96979-fig2-data1.zip › Figure 2_ source data/Raw unedited gels for (Figure 2)/Anti-Calreticulin/2023-04-28-155909/2023-04-28-155909_1_TH.jpg]

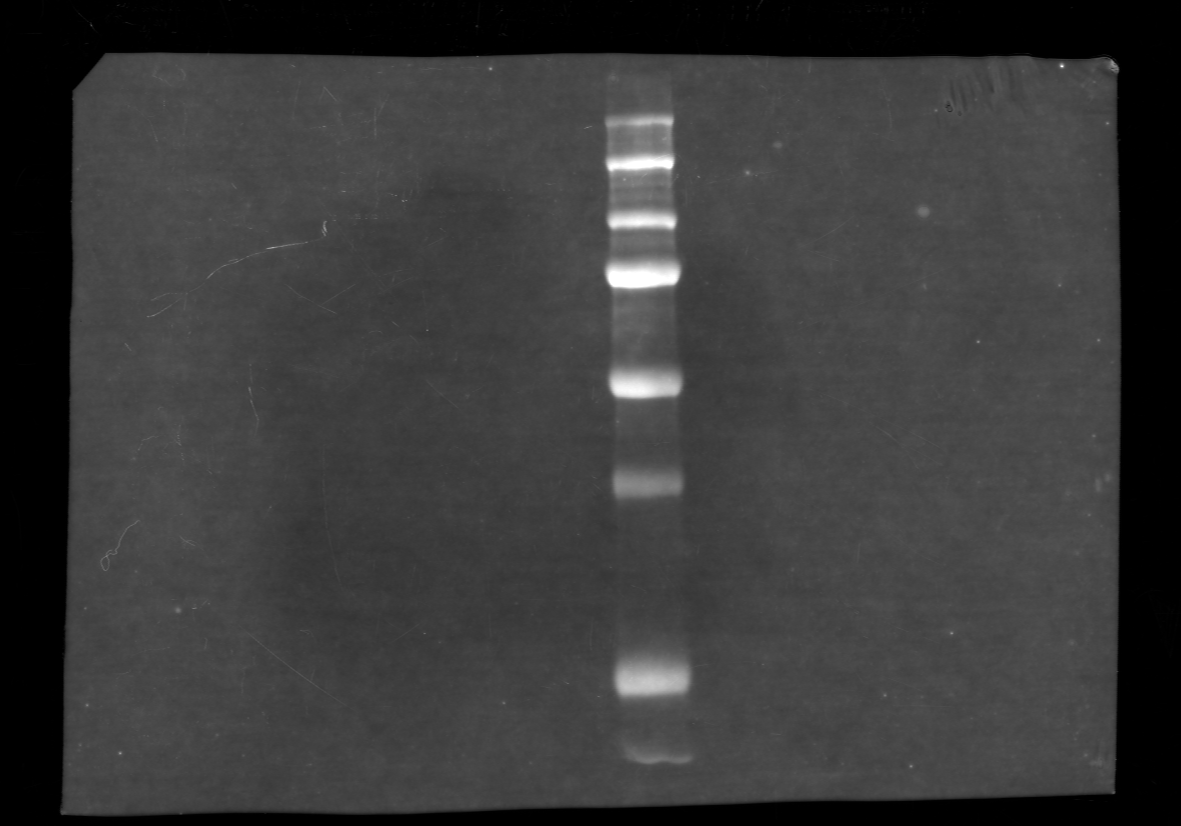

Supplement: Figure 2—source data 1. [file elife-96979-fig2-data1.zip › Figure 2_ source data/Raw unedited gels for (Figure 2)/Anti-BiP/2023-05-03-143040/700.TIF]

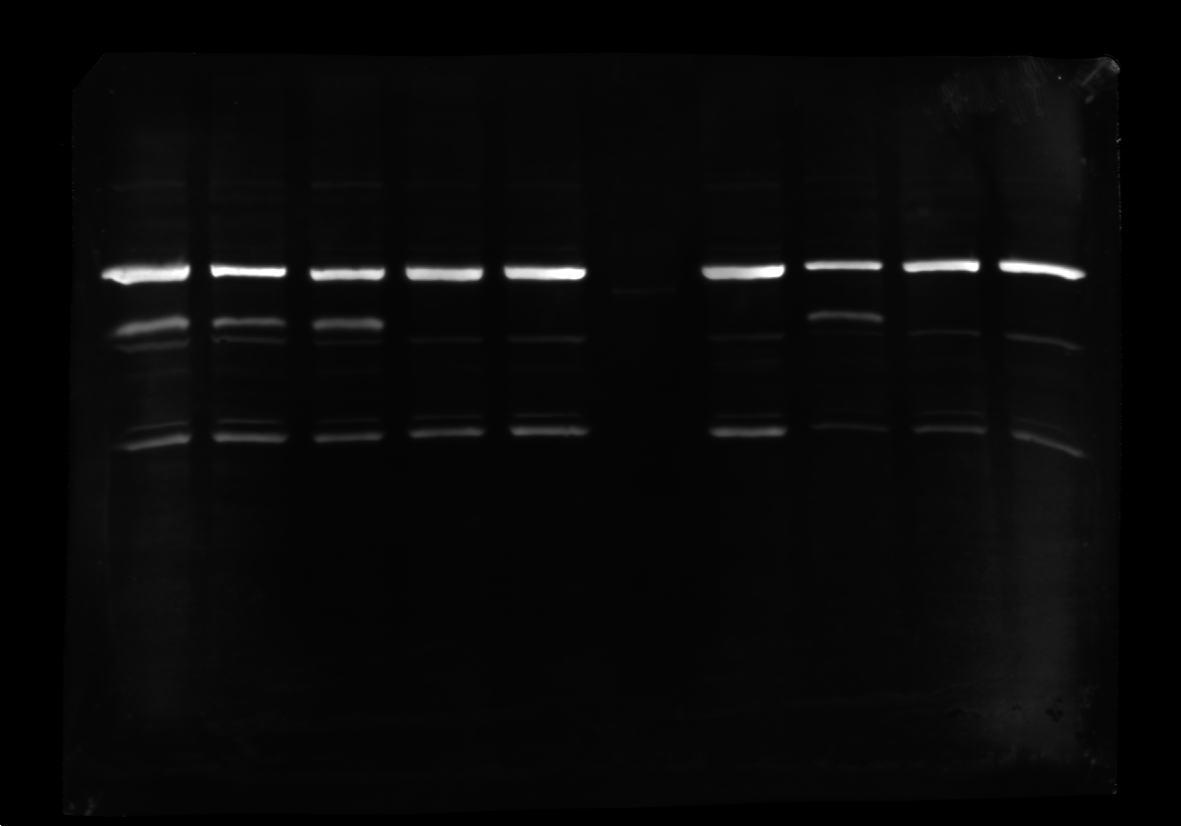

Supplement: Figure 2—source data 1. [file elife-96979-fig2-data1.zip › Figure 2_ source data/Raw unedited gels for (Figure 2)/Anti-BiP/2023-05-03-143040/800.TIF]

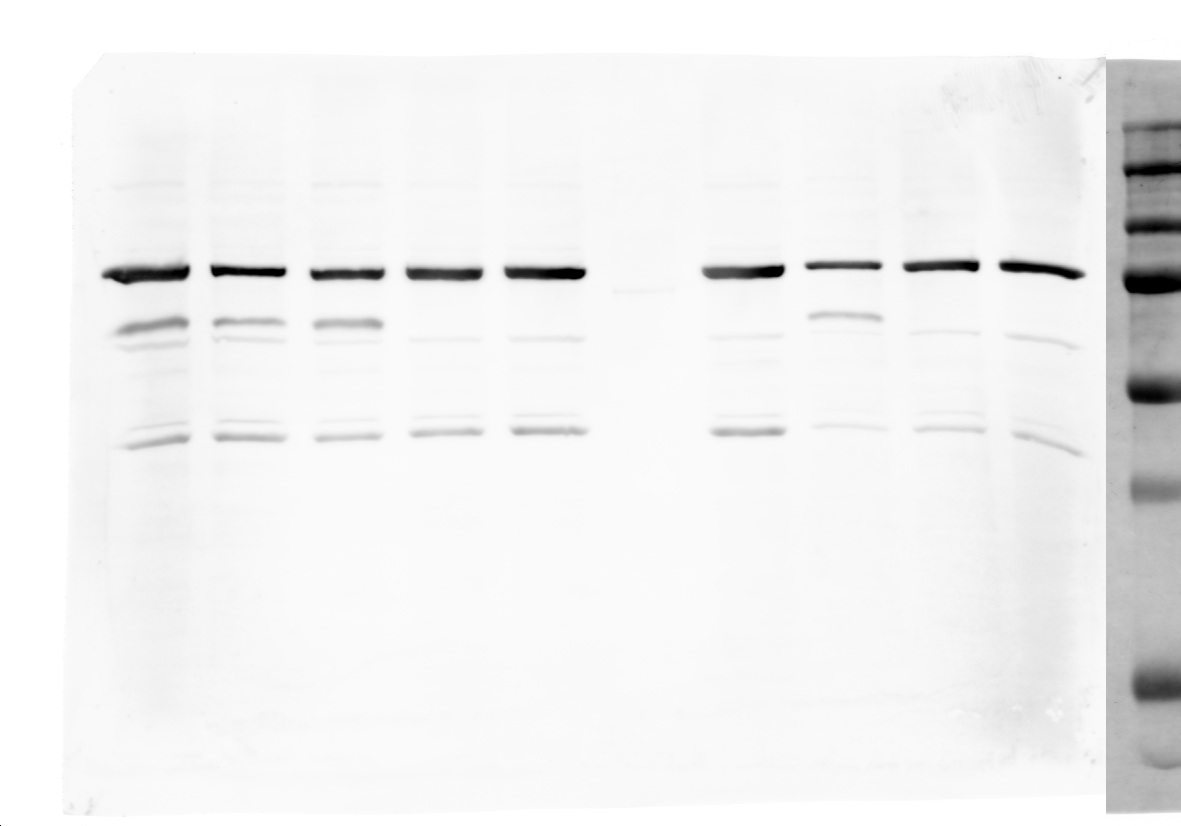

Supplement: Figure 2—source data 1. [file elife-96979-fig2-data1.zip › Figure 2_ source data/Raw unedited gels for (Figure 2)/Anti-BiP/2023-05-03-143040/800_modified.tif]

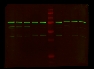

Supplement: Figure 2—source data 1. [file elife-96979-fig2-data1.zip › Figure 2_ source data/Raw unedited gels for (Figure 2)/Anti-BiP/2023-05-03-143040/2023-05-03-143040_1_TH.jpg]

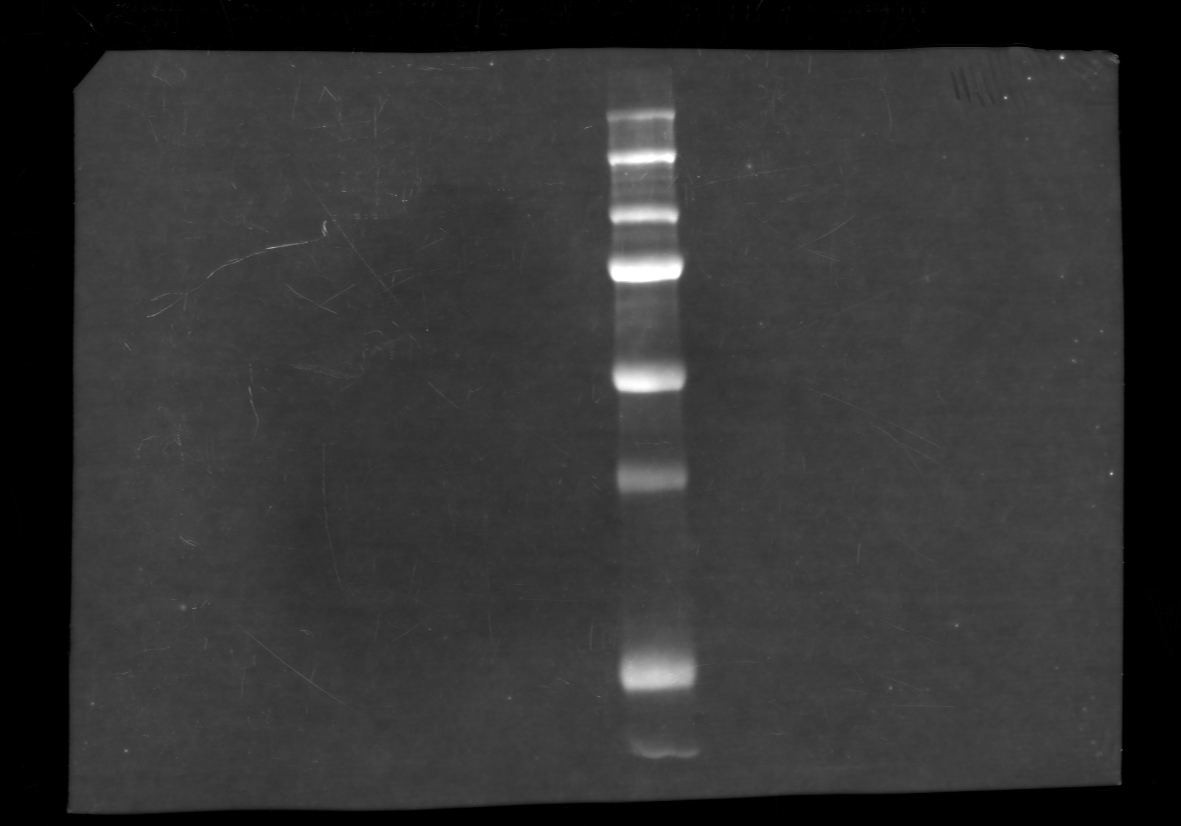

Supplement: Figure 2—source data 1. [file elife-96979-fig2-data1.zip › Figure 2_ source data/Raw unedited gels for (Figure 2)/Anti-IRE1/2023-05-02-152742/700.TIF]

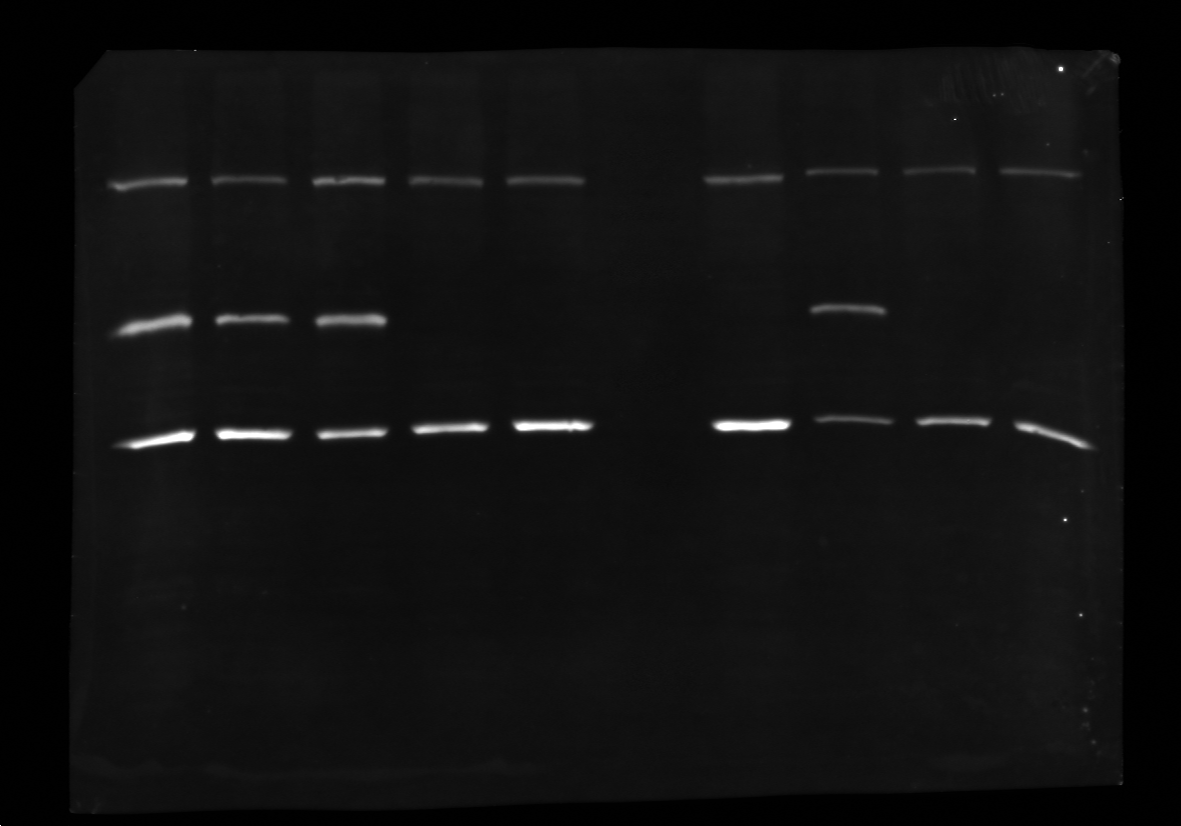

Supplement: Figure 2—source data 1. [file elife-96979-fig2-data1.zip › Figure 2_ source data/Raw unedited gels for (Figure 2)/Anti-IRE1/2023-05-02-152742/800.TIF]

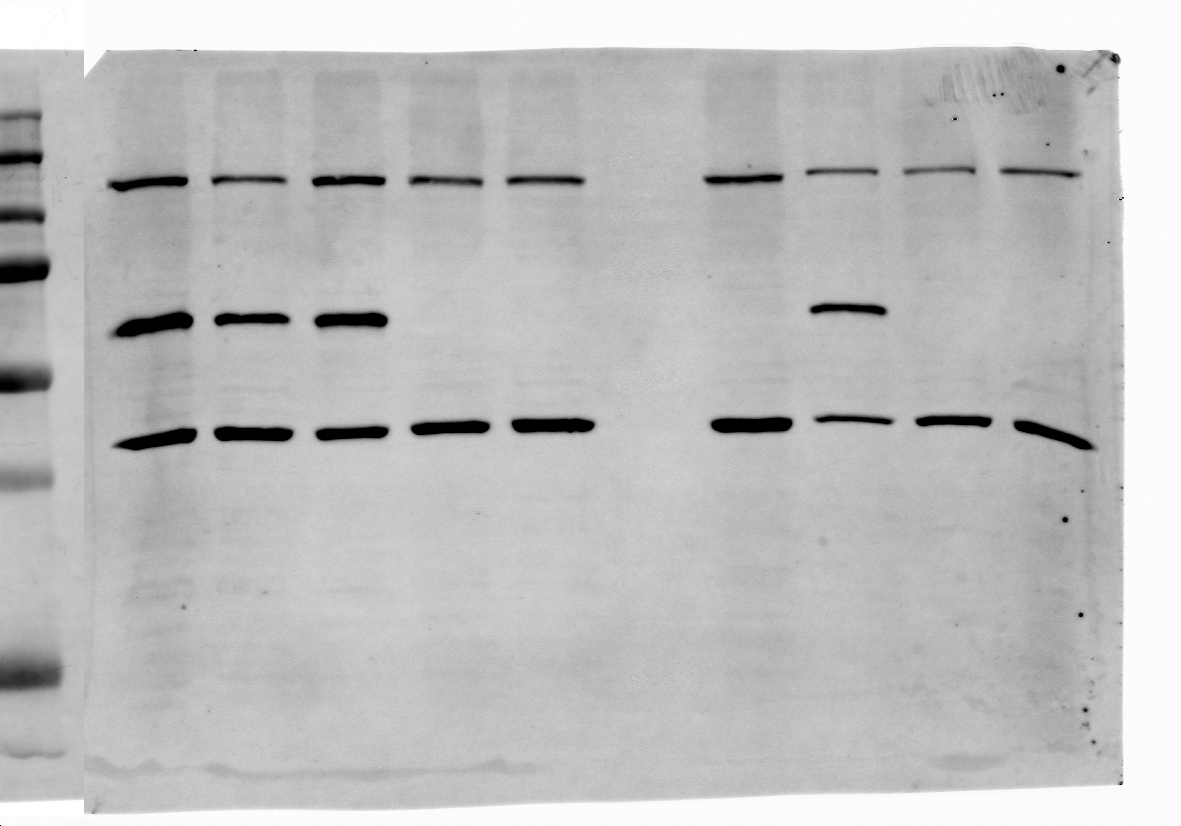

Supplement: Figure 2—source data 1. [file elife-96979-fig2-data1.zip › Figure 2_ source data/Raw unedited gels for (Figure 2)/Anti-IRE1/2023-05-02-152742/800_modified.tif]

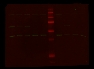

Supplement: Figure 2—source data 1. [file elife-96979-fig2-data1.zip › Figure 2_ source data/Raw unedited gels for (Figure 2)/Anti-IRE1/2023-05-02-152742/2023-05-02-152742_1_TH.jpg]

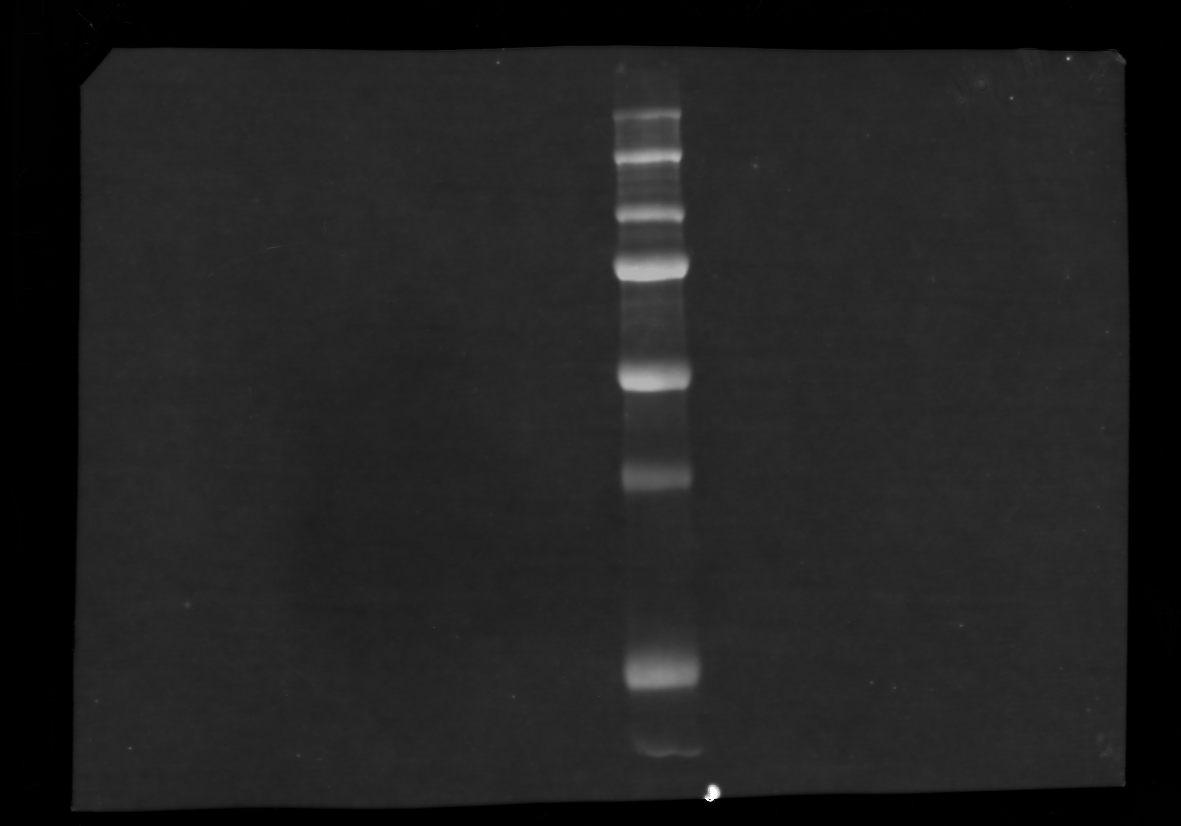

Supplement: Figure 2—source data 1. [file elife-96979-fig2-data1.zip › Figure 2_ source data/Raw unedited gels for (Figure 2)/Anti-Actin/2023-05-01-005331/700.TIF]

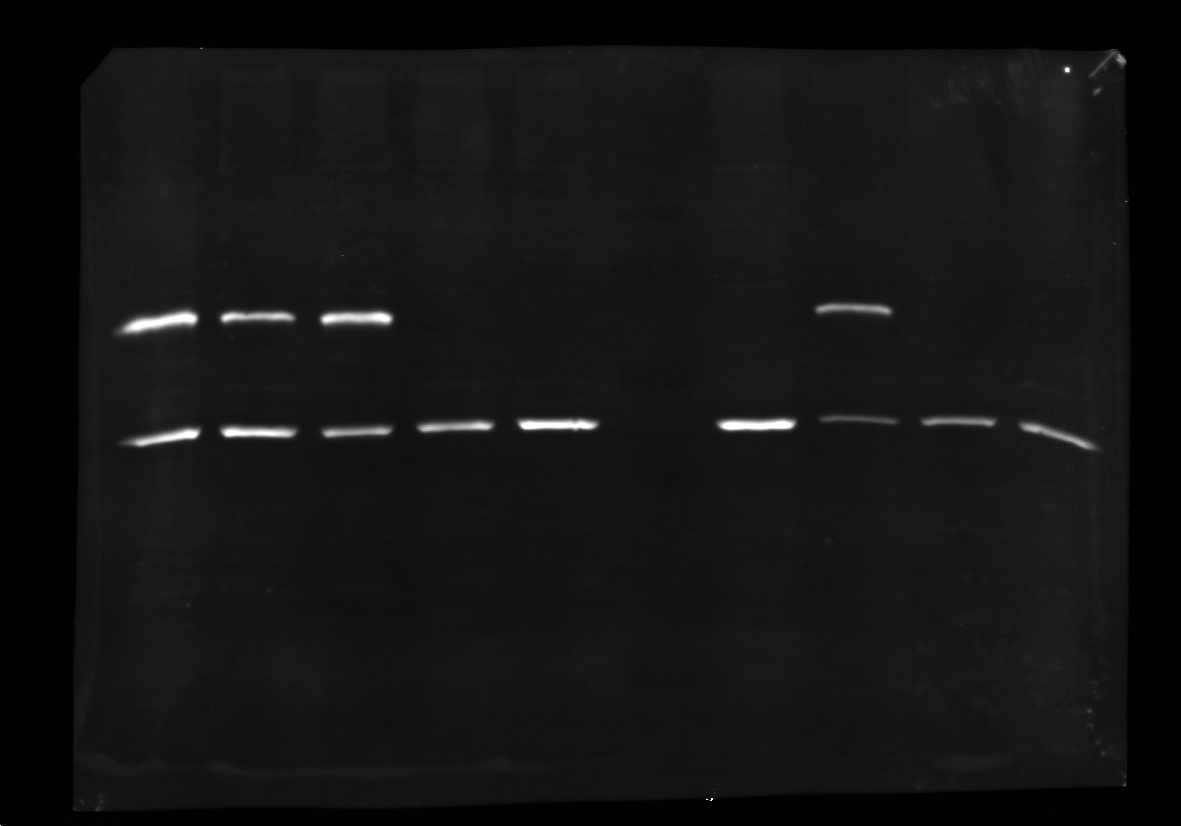

Supplement: Figure 2—source data 1. [file elife-96979-fig2-data1.zip › Figure 2_ source data/Raw unedited gels for (Figure 2)/Anti-Actin/2023-05-01-005331/800.TIF]

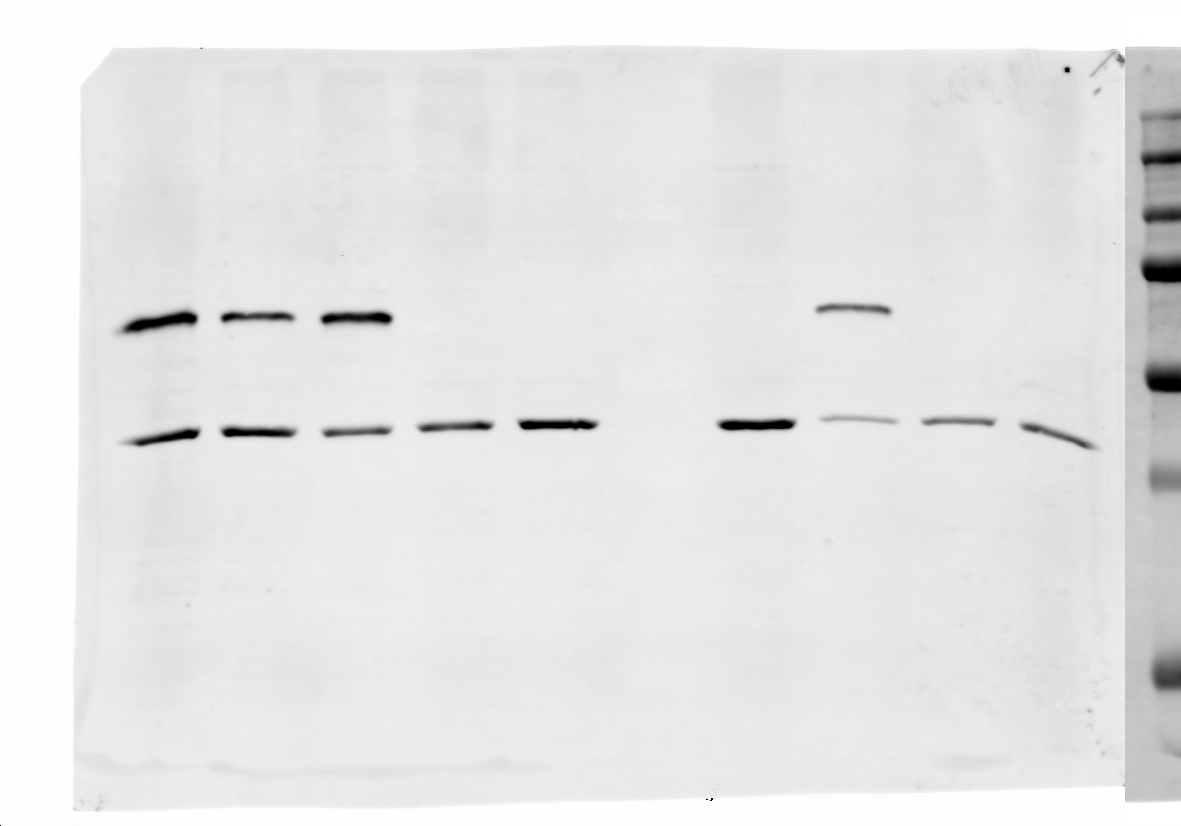

Supplement: Figure 2—source data 1. [file elife-96979-fig2-data1.zip › Figure 2_ source data/Raw unedited gels for (Figure 2)/Anti-Actin/2023-05-01-005331/800_modified.tif]

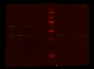

Supplement: Figure 2—source data 1. [file elife-96979-fig2-data1.zip › Figure 2_ source data/Raw unedited gels for (Figure 2)/Anti-Actin/2023-05-01-005331/2023-05-01-005331_1_TH.jpg]

Uncropped and labelled gels for (Figure 2\_figure supplemental 1)

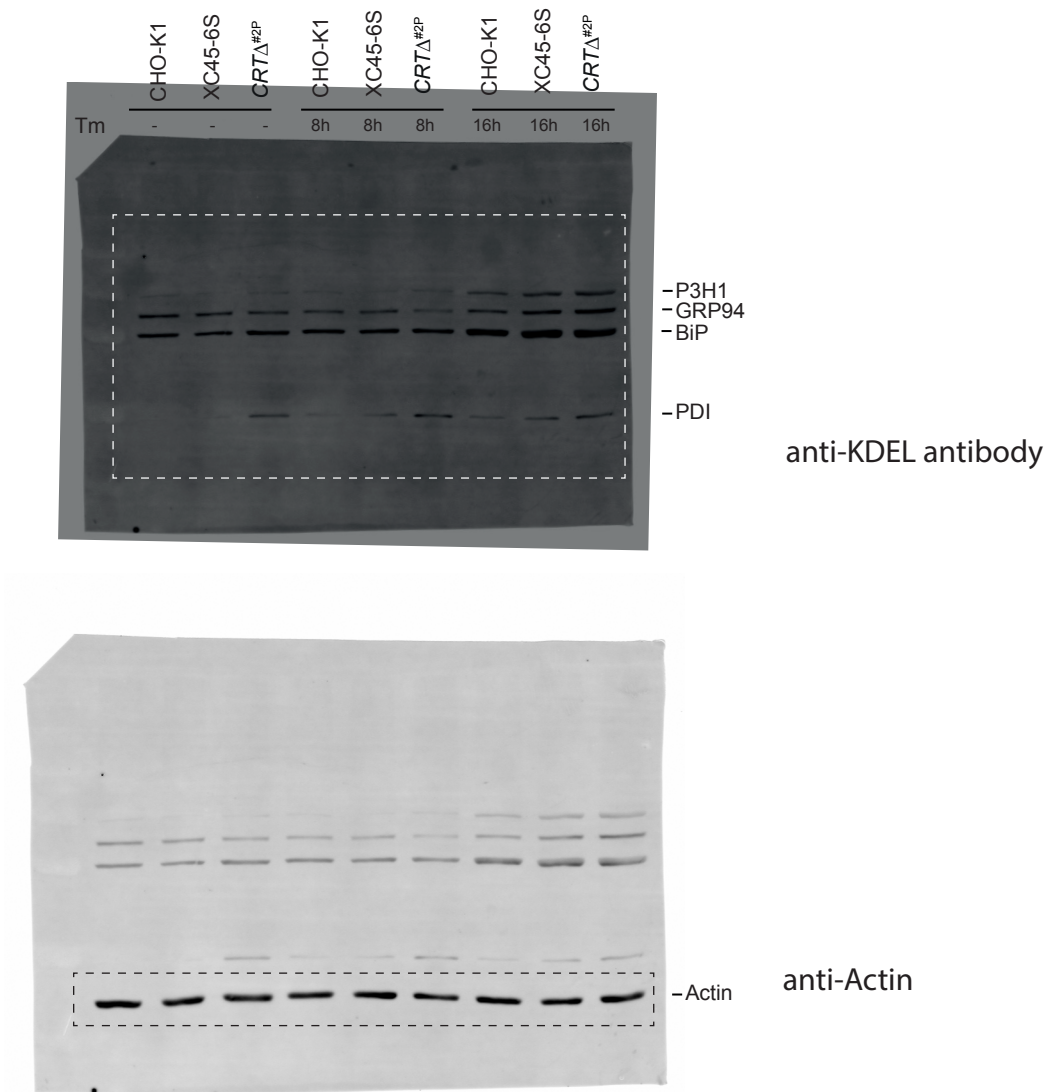

Supplement: Figure 2—figure supplement 1—source data 1. [file elife-96979-fig2-figsupp1-data1.zip › Figure 2_figure supplement 1_source data /Uncropped and labelled gels for (Figure 2_figure supplement 1).pdf]

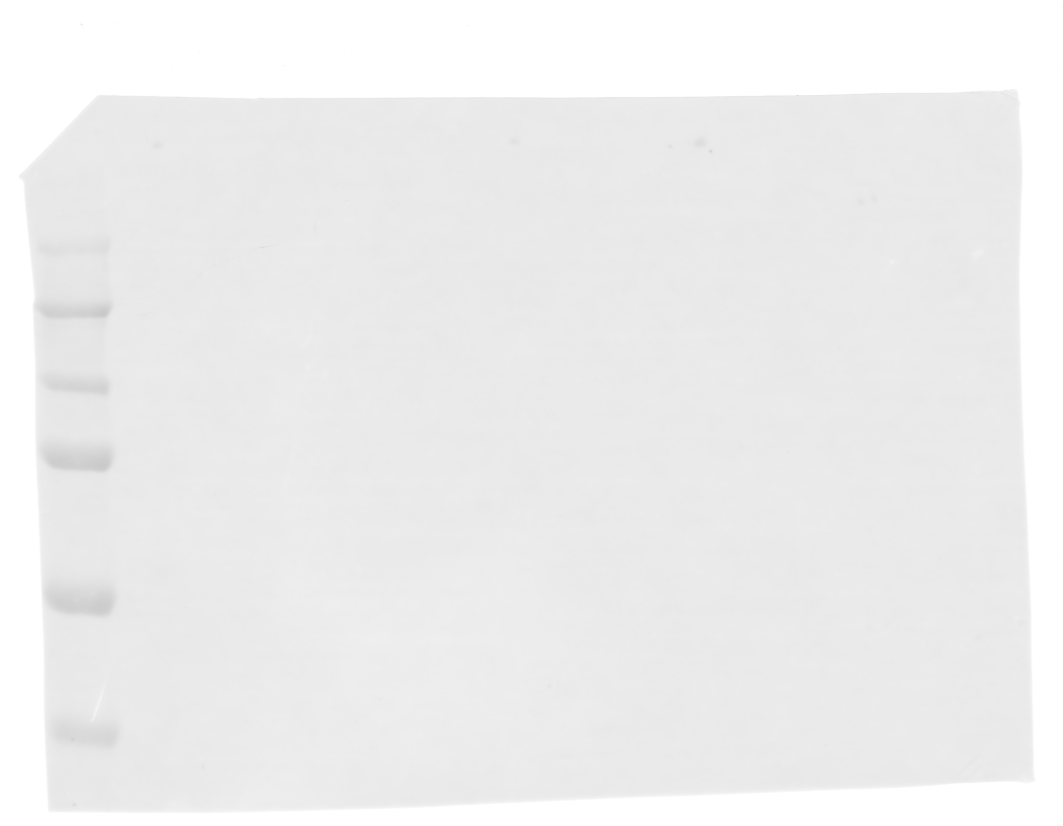

Supplement: Figure 2—figure supplement 1—source data 1. [file elife-96979-fig2-figsupp1-data1.zip › Figure 2_figure supplement 1_source data /Raw unedited gels for (Figure 2_figure 2_figure supplement 1)/Anti-KDEL/2024-05-03-135642/700MODIFIED.tif]

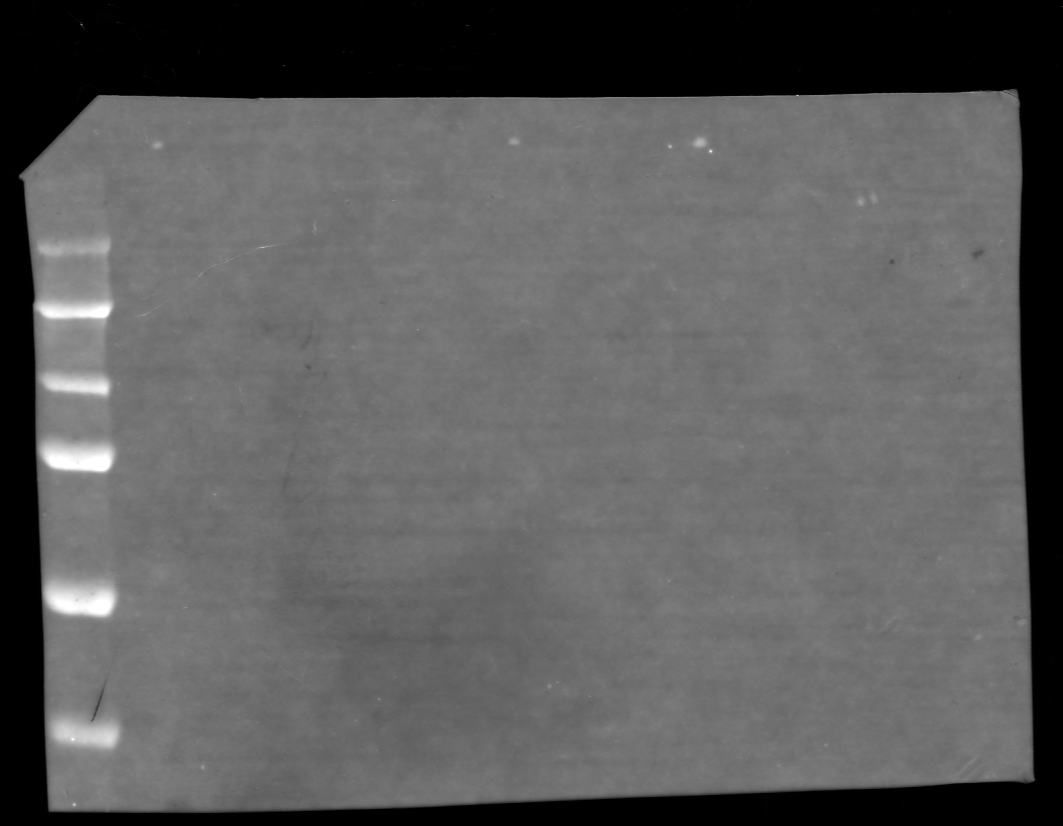

Supplement: Figure 2—figure supplement 1—source data 1. [file elife-96979-fig2-figsupp1-data1.zip › Figure 2_figure supplement 1_source data /Raw unedited gels for (Figure 2_figure 2_figure supplement 1)/Anti-KDEL/2024-05-03-135642/700.TIF]

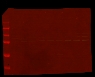

Supplement: Figure 2—figure supplement 1—source data 1. [file elife-96979-fig2-figsupp1-data1.zip › Figure 2_figure supplement 1_source data /Raw unedited gels for (Figure 2_figure 2_figure supplement 1)/Anti-KDEL/2024-05-03-135642/2024-05-03-135642_1_TH.jpg]

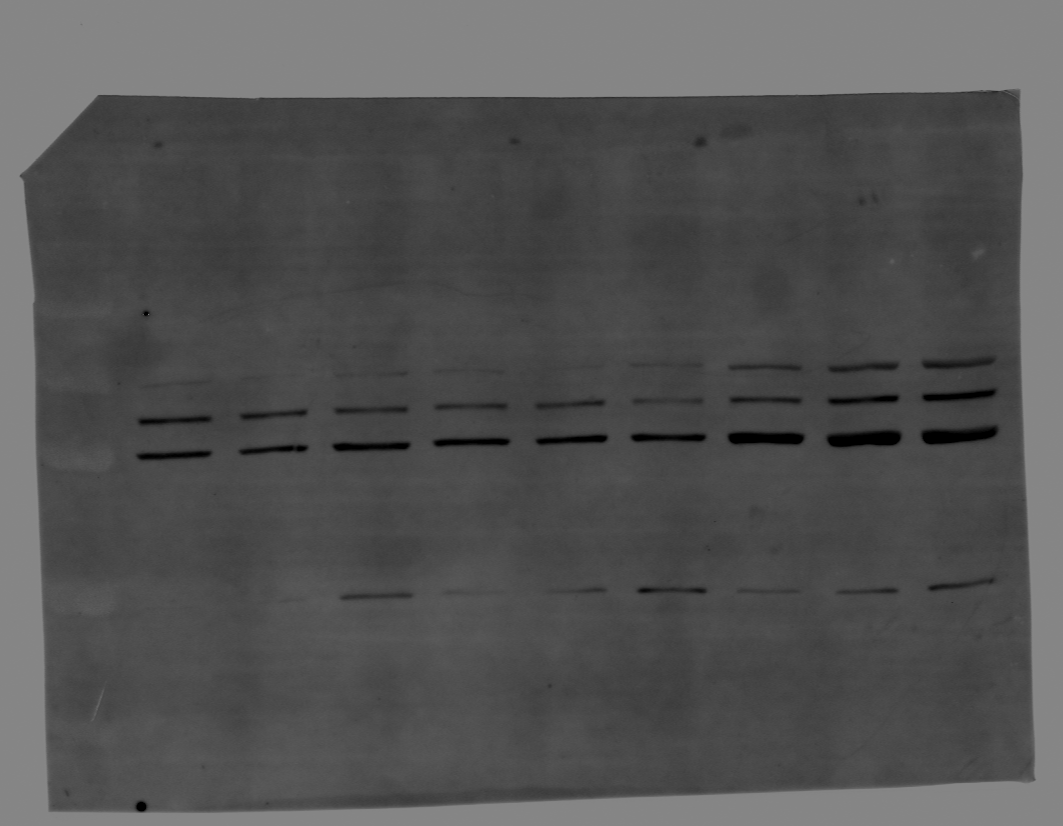

Supplement: Figure 2—figure supplement 1—source data 1. [file elife-96979-fig2-figsupp1-data1.zip › Figure 2_figure supplement 1_source data /Raw unedited gels for (Figure 2_figure 2_figure supplement 1)/Anti-KDEL/2024-05-03-135642/800MODIFIED.tif]

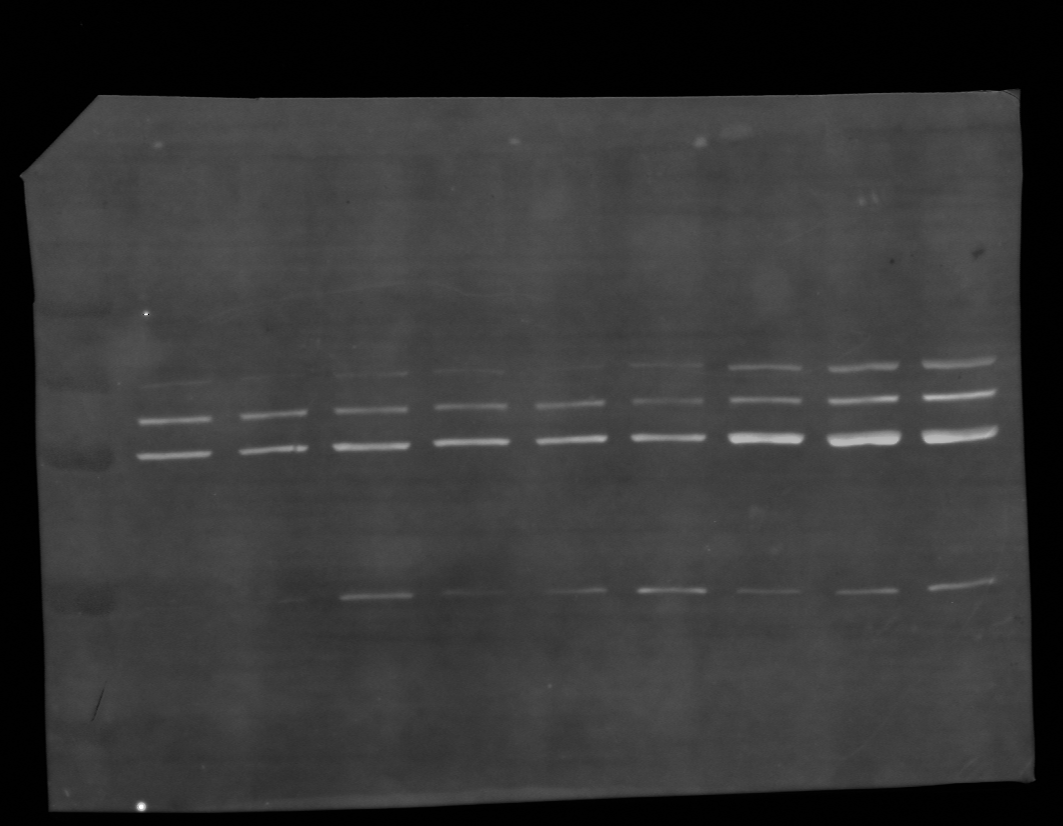

Supplement: Figure 2—figure supplement 1—source data 1. [file elife-96979-fig2-figsupp1-data1.zip › Figure 2_figure supplement 1_source data /Raw unedited gels for (Figure 2_figure 2_figure supplement 1)/Anti-KDEL/2024-05-03-135642/800.TIF]

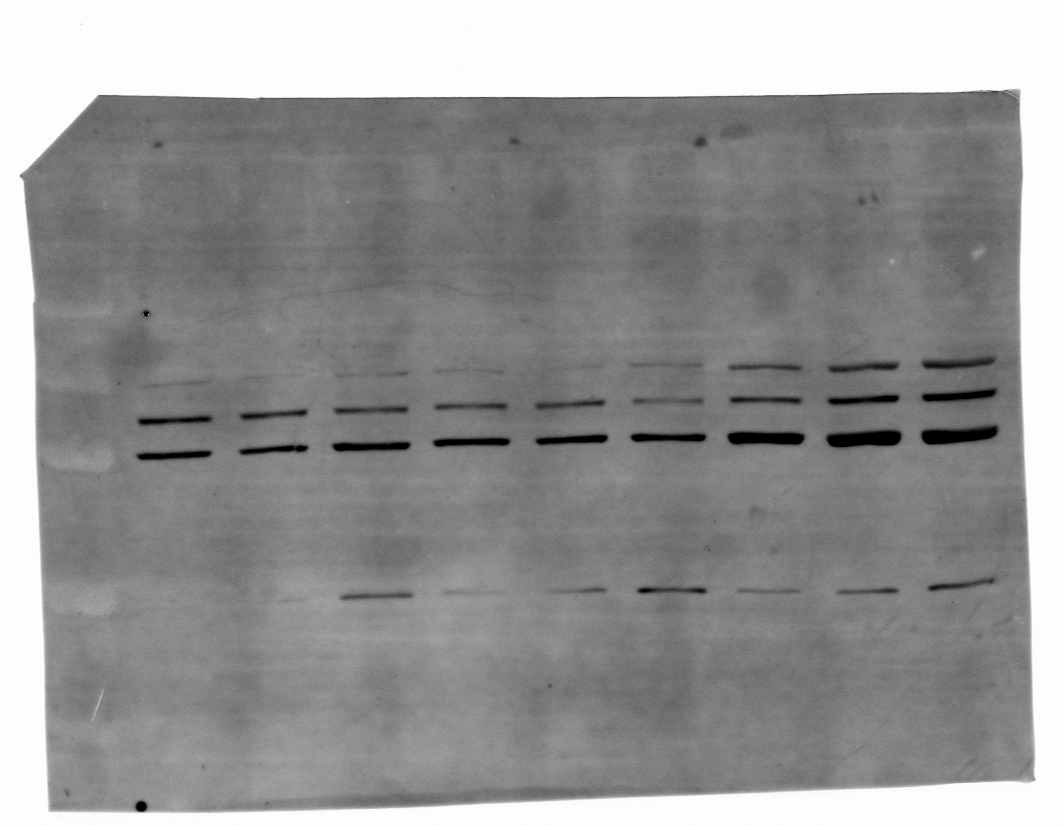

Supplement: Figure 2—figure supplement 1—source data 1. [file elife-96979-fig2-figsupp1-data1.zip › Figure 2_figure supplement 1_source data /Raw unedited gels for (Figure 2_figure 2_figure supplement 1)/Anti-KDEL/2024-05-03-135642/800_modified.tif]

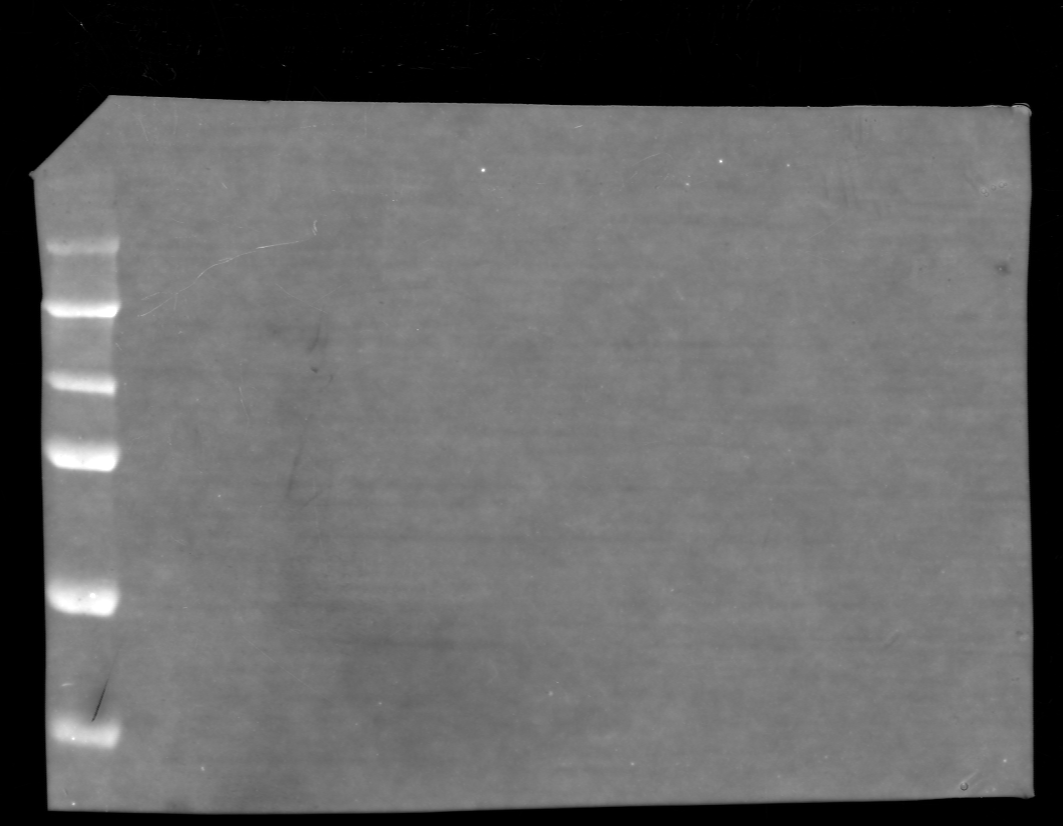

Supplement: Figure 2—figure supplement 1—source data 1. [file elife-96979-fig2-figsupp1-data1.zip › Figure 2_figure supplement 1_source data /Raw unedited gels for (Figure 2_figure 2_figure supplement 1)/Anti-actin-30/2024-05-07-151942/700.TIF]

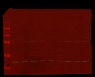

Supplement: Figure 2—figure supplement 1—source data 1. [file elife-96979-fig2-figsupp1-data1.zip › Figure 2_figure supplement 1_source data /Raw unedited gels for (Figure 2_figure 2_figure supplement 1)/Anti-actin-30/2024-05-07-151942/2024-05-07-151942_1_TH.jpg]

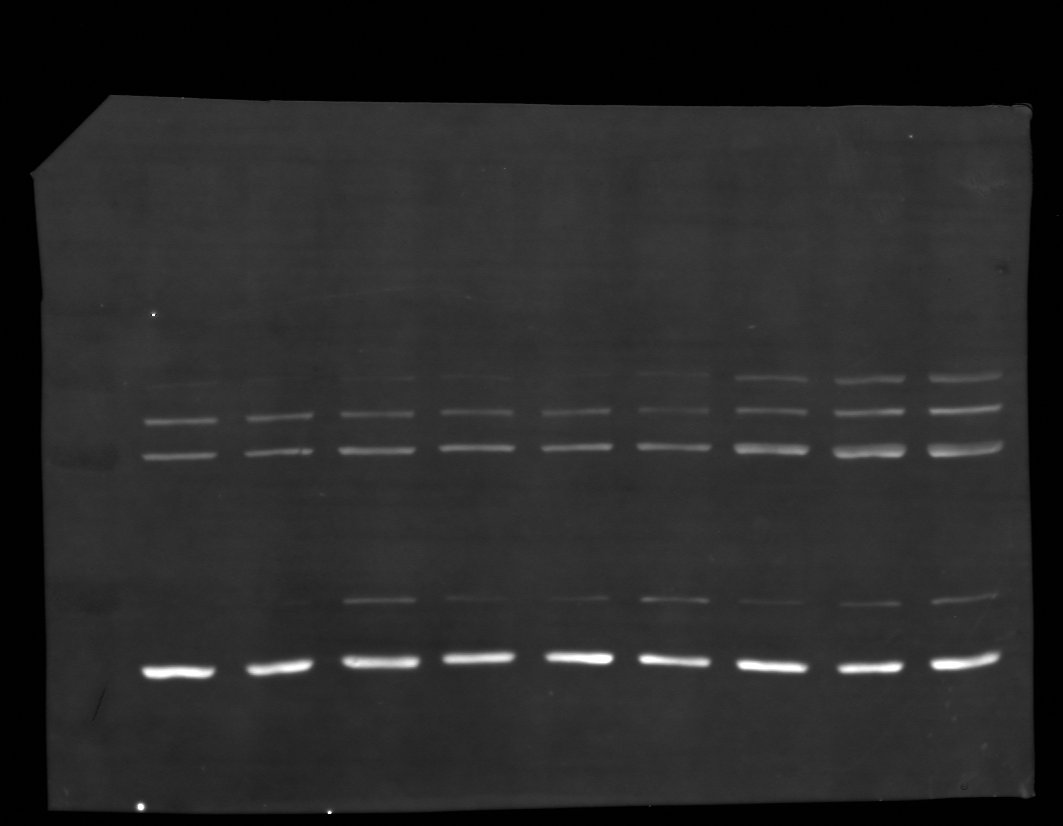

Supplement: Figure 2—figure supplement 1—source data 1. [file elife-96979-fig2-figsupp1-data1.zip › Figure 2_figure supplement 1_source data /Raw unedited gels for (Figure 2_figure 2_figure supplement 1)/Anti-actin-30/2024-05-07-151942/800.TIF]

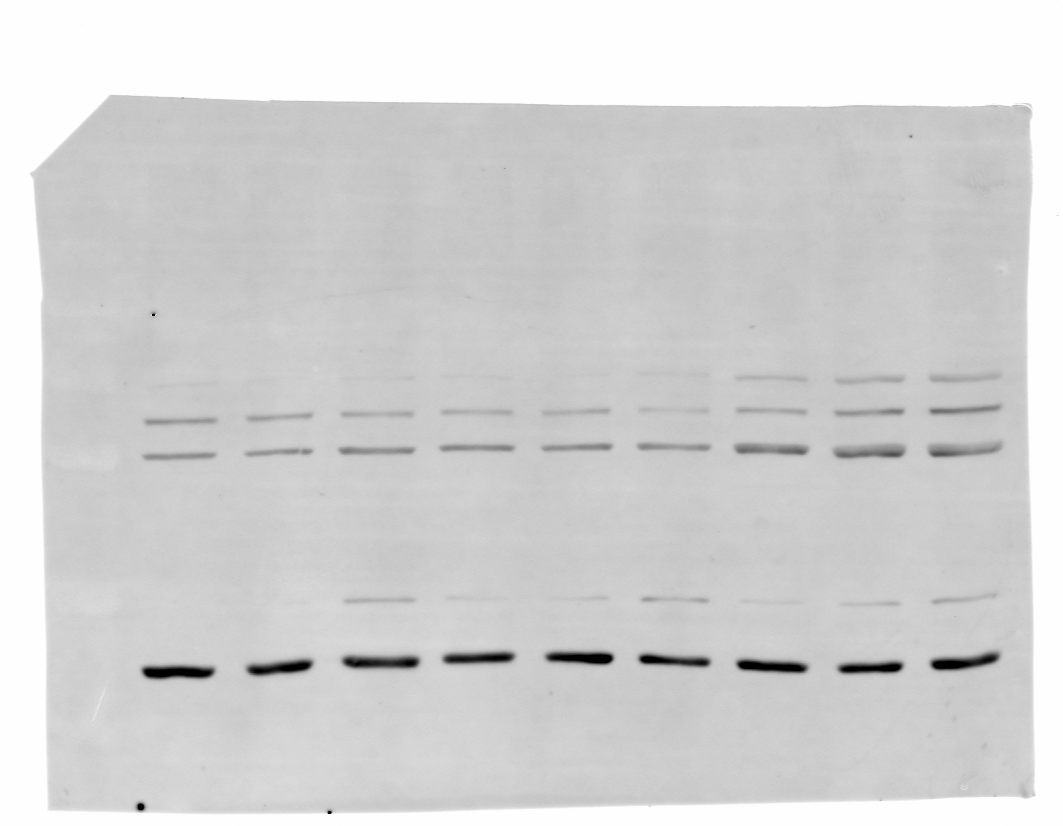

Supplement: Figure 2—figure supplement 1—source data 1. [file elife-96979-fig2-figsupp1-data1.zip › Figure 2_figure supplement 1_source data /Raw unedited gels for (Figure 2_figure 2_figure supplement 1)/Anti-actin-30/2024-05-07-151942/800_modified.tif]

Uncropped and labelled gels for (Figure 2-figure supplement 2)

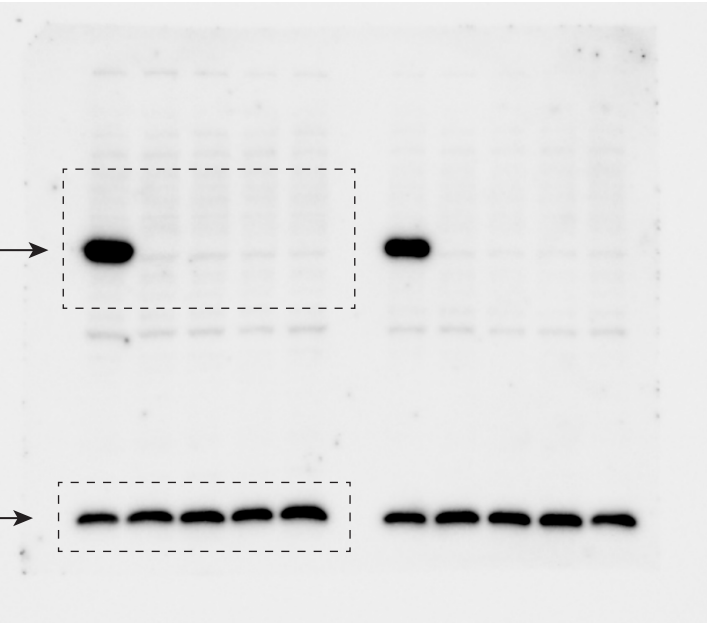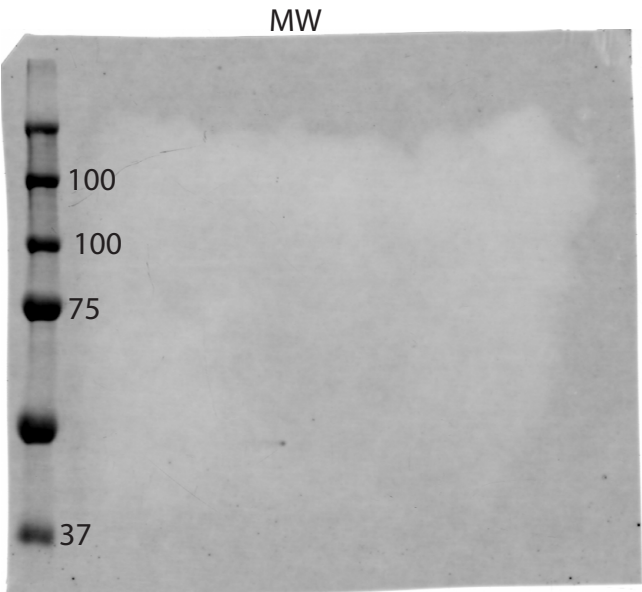

Supplement: Figure 2—figure supplement 2—source data 1. [file elife-96979-fig2-figsupp2-data1.zip › Figure 2_figure supplement 2_source data /Uncropped and labelled gels for (Figure 2_figure supplement 2).pdf]

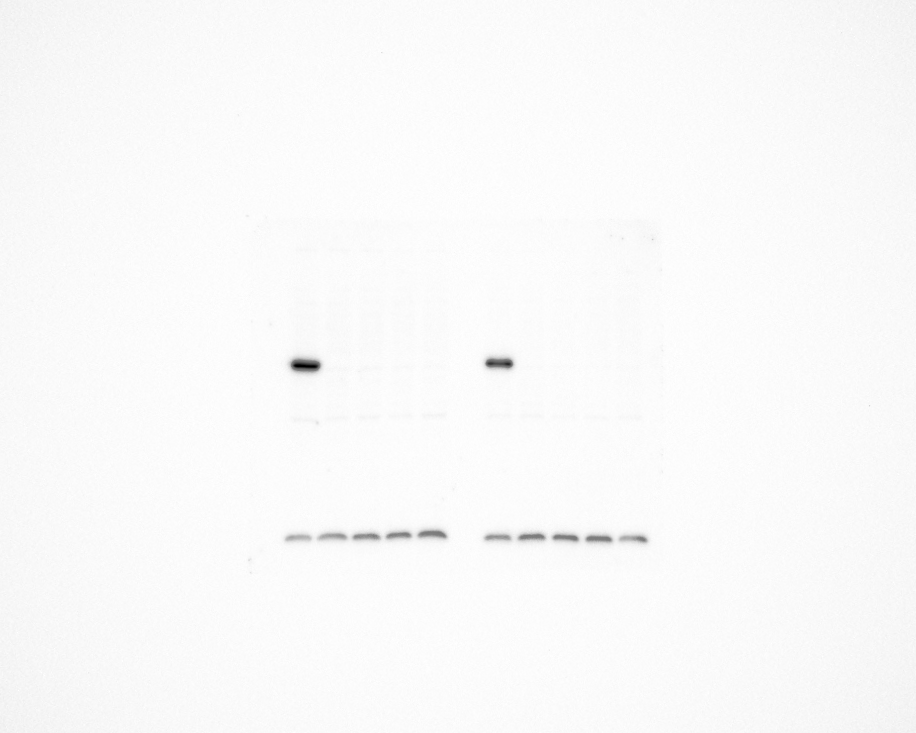

Supplement: Figure 2—figure supplement 2—source data 1. [file elife-96979-fig2-figsupp2-data1.zip › Figure 2_figure supplement 2_source data /Raw unedited gels for (Figure 2_figure supplement 2)/Anti-Calnexin/aog 2023-08-18 16h01m05s(Chemiluminescence).raw16.tif]

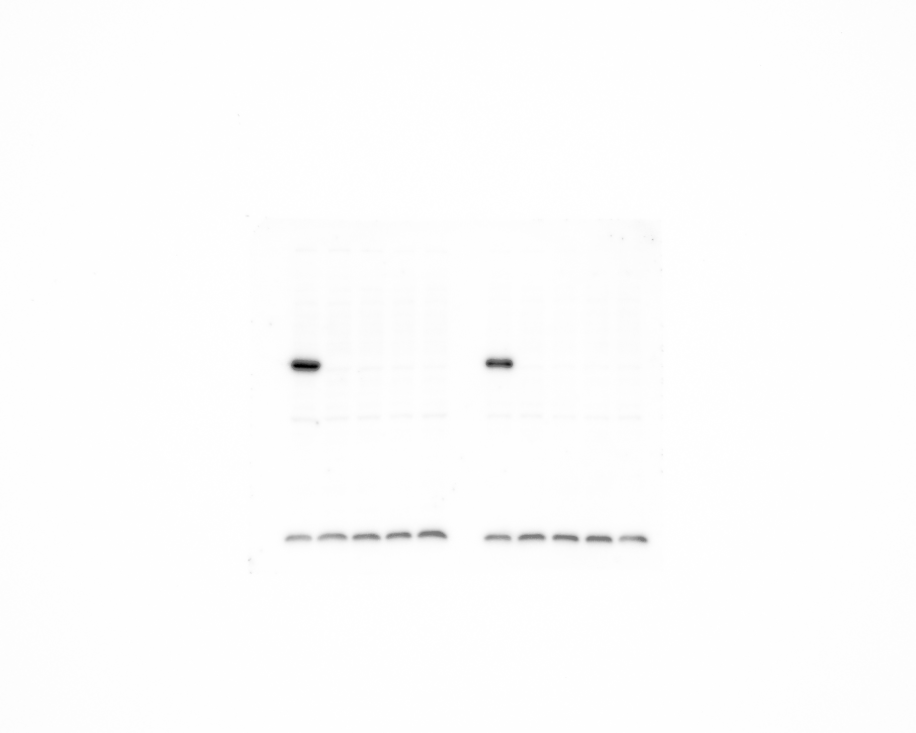

Supplement: Figure 2—figure supplement 2—source data 1. [file elife-96979-fig2-figsupp2-data1.zip › Figure 2_figure supplement 2_source data /Raw unedited gels for (Figure 2_figure supplement 2)/Anti-Calnexin/aog 2023-08-18 16h01m38s(Chemiluminescence).raw16.tif]

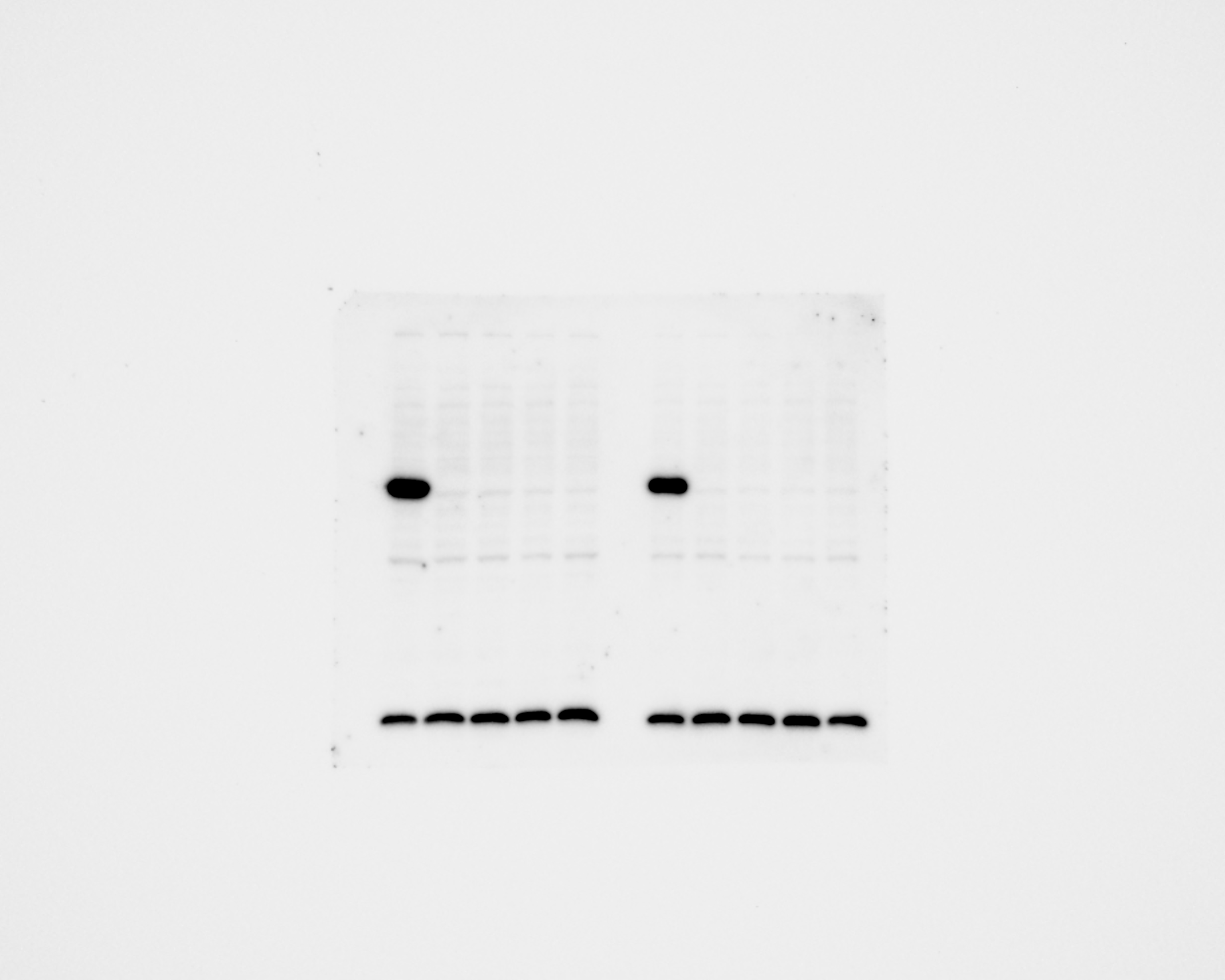

Supplement: Figure 2—figure supplement 2—source data 1. [file elife-96979-fig2-figsupp2-data1.zip › Figure 2_figure supplement 2_source data /Raw unedited gels for (Figure 2_figure supplement 2)/Anti-Calnexin/aog 2023-08-18 16h01m38s(Chemiluminescence).tif]

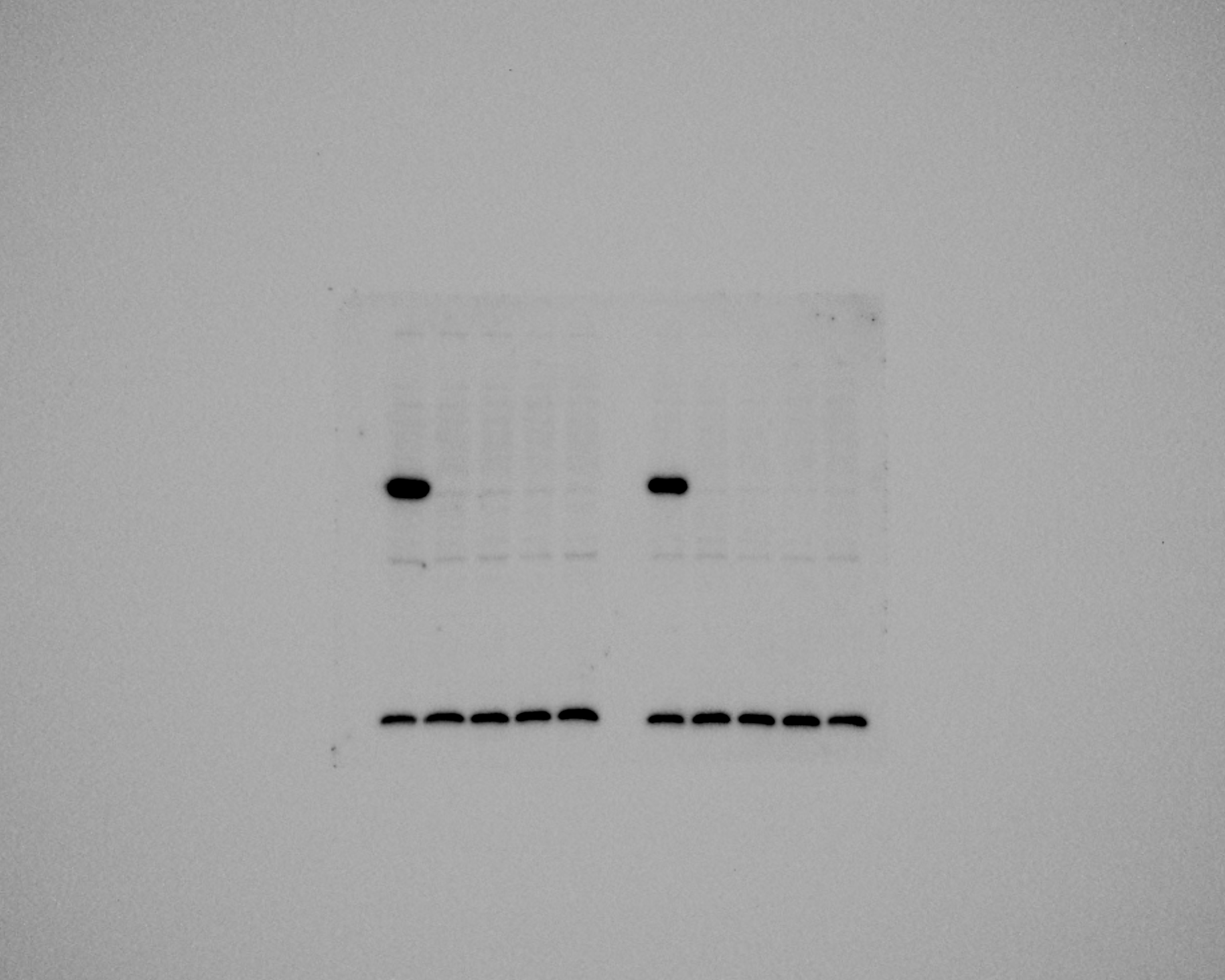

Supplement: Figure 2—figure supplement 2—source data 1. [file elife-96979-fig2-figsupp2-data1.zip › Figure 2_figure supplement 2_source data /Raw unedited gels for (Figure 2_figure supplement 2)/Anti-Calnexin/aog 2023-08-18 16h01m05s(Chemiluminescence).tif]

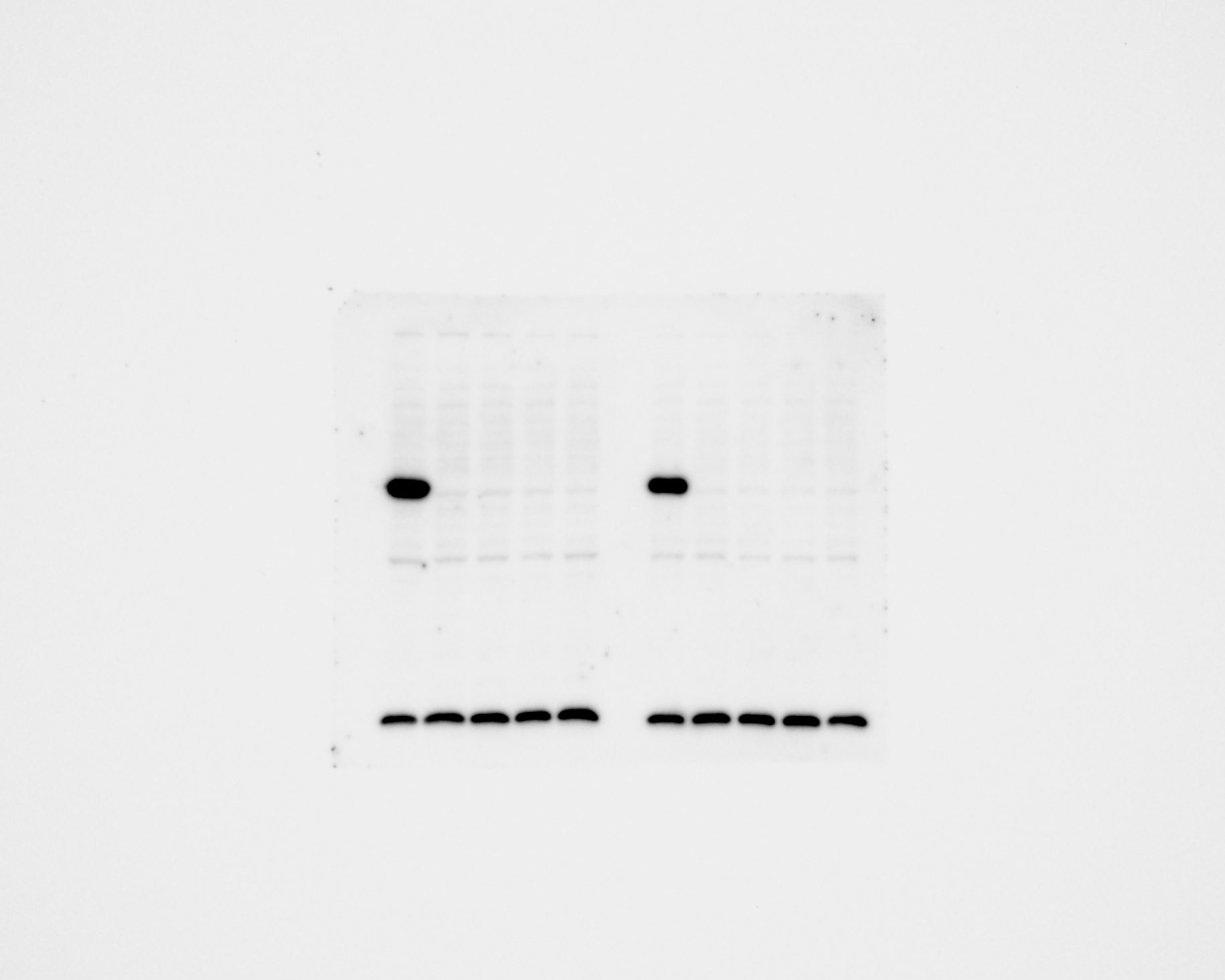

Supplement: Figure 2—figure supplement 2—source data 1. [file elife-96979-fig2-figsupp2-data1.zip › Figure 2_figure supplement 2_source data /Raw unedited gels for (Figure 2_figure supplement 2)/Anti-Calnexin/aog 2023-08-18 16h01m38s(Chemiluminescence).jpg]

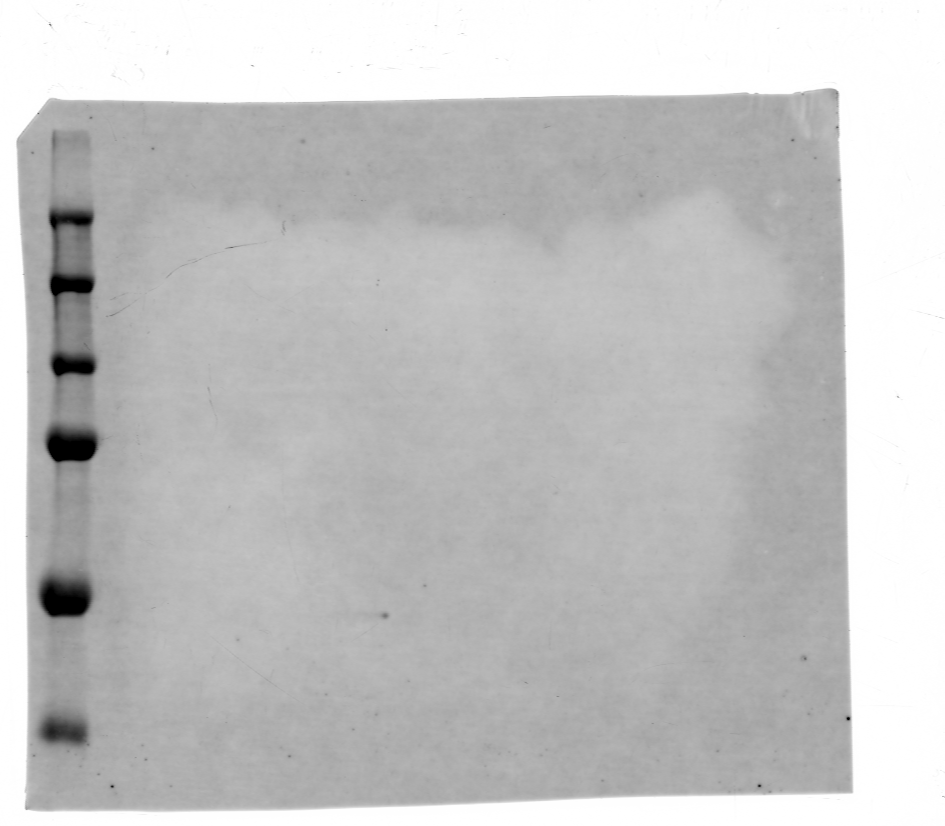

Supplement: Figure 2—figure supplement 2—source data 1. [file elife-96979-fig2-figsupp2-data1.zip › Figure 2_figure supplement 2_source data /Raw unedited gels for (Figure 2_figure supplement 2)/Anti-Actin /2023-08-21-154407/700MODIFIED.tif]

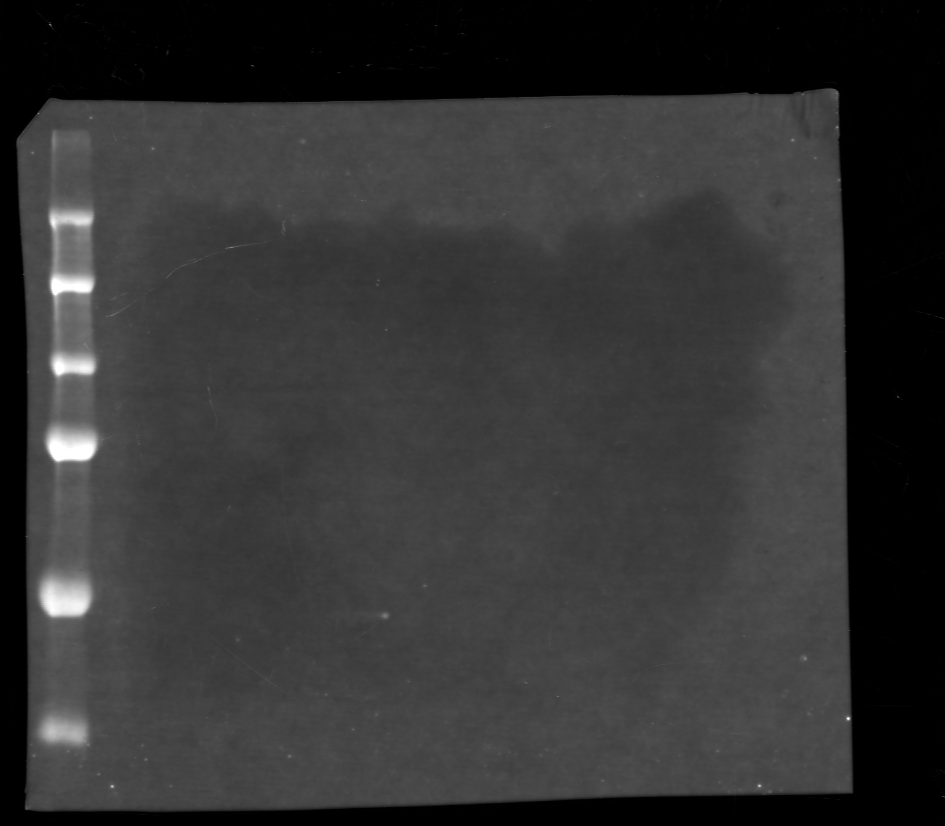

Supplement: Figure 2—figure supplement 2—source data 1. [file elife-96979-fig2-figsupp2-data1.zip › Figure 2_figure supplement 2_source data /Raw unedited gels for (Figure 2_figure supplement 2)/Anti-Actin /2023-08-21-154407/700.TIF]

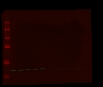

Supplement: Figure 2—figure supplement 2—source data 1. [file elife-96979-fig2-figsupp2-data1.zip › Figure 2_figure supplement 2_source data /Raw unedited gels for (Figure 2_figure supplement 2)/Anti-Actin /2023-08-21-154407/2023-08-21-154407_1_TH.jpg]

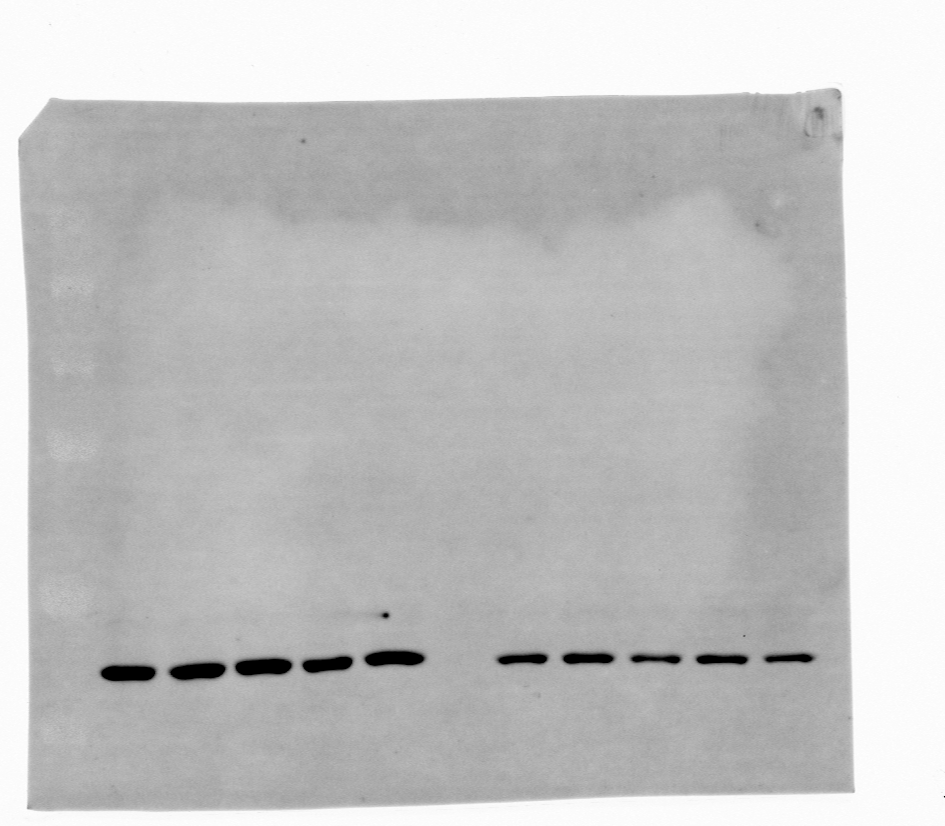

Supplement: Figure 2—figure supplement 2—source data 1. [file elife-96979-fig2-figsupp2-data1.zip › Figure 2_figure supplement 2_source data /Raw unedited gels for (Figure 2_figure supplement 2)/Anti-Actin /2023-08-21-154407/800MODIFED.tif]

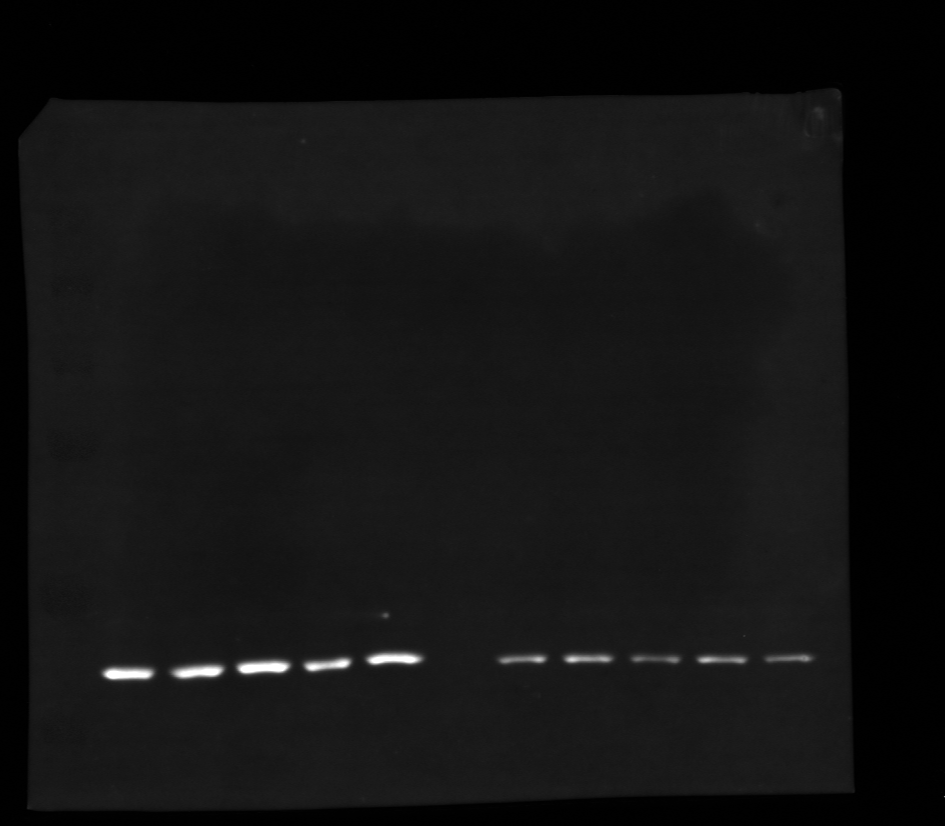

Supplement: Figure 2—figure supplement 2—source data 1. [file elife-96979-fig2-figsupp2-data1.zip › Figure 2_figure supplement 2_source data /Raw unedited gels for (Figure 2_figure supplement 2)/Anti-Actin /2023-08-21-154407/800.TIF]

Uncropped and labelled gels for (Figure 3B.1)

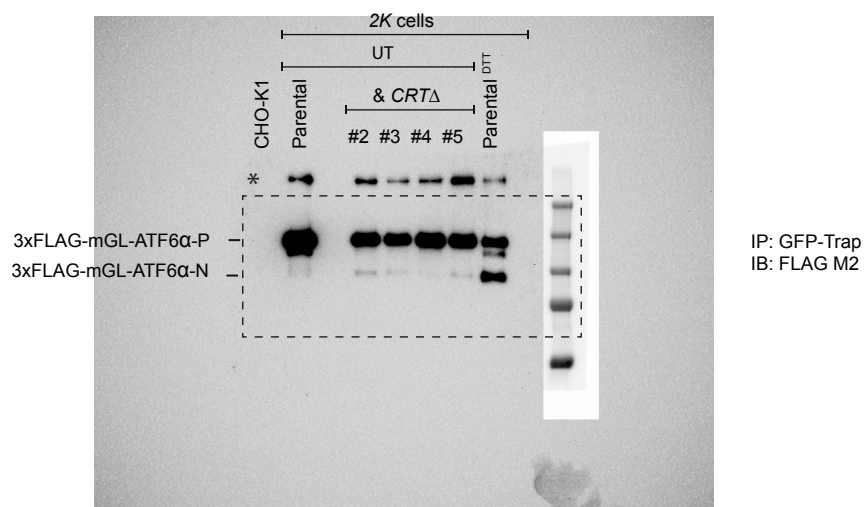

Uncropped and labelled gels for (Figure 3B.2)

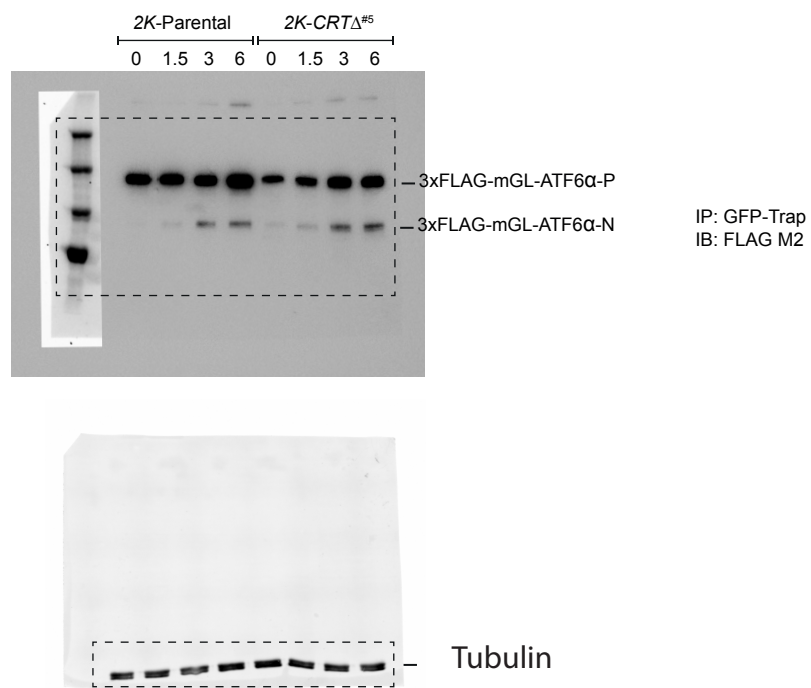

Supplement: Figure 3—source data 1. [file elife-96979-fig3-data1.zip › Figure 3_ source data/Uncropped and labelled gels for (Figure 3B.1 and 3B.2).pdf]

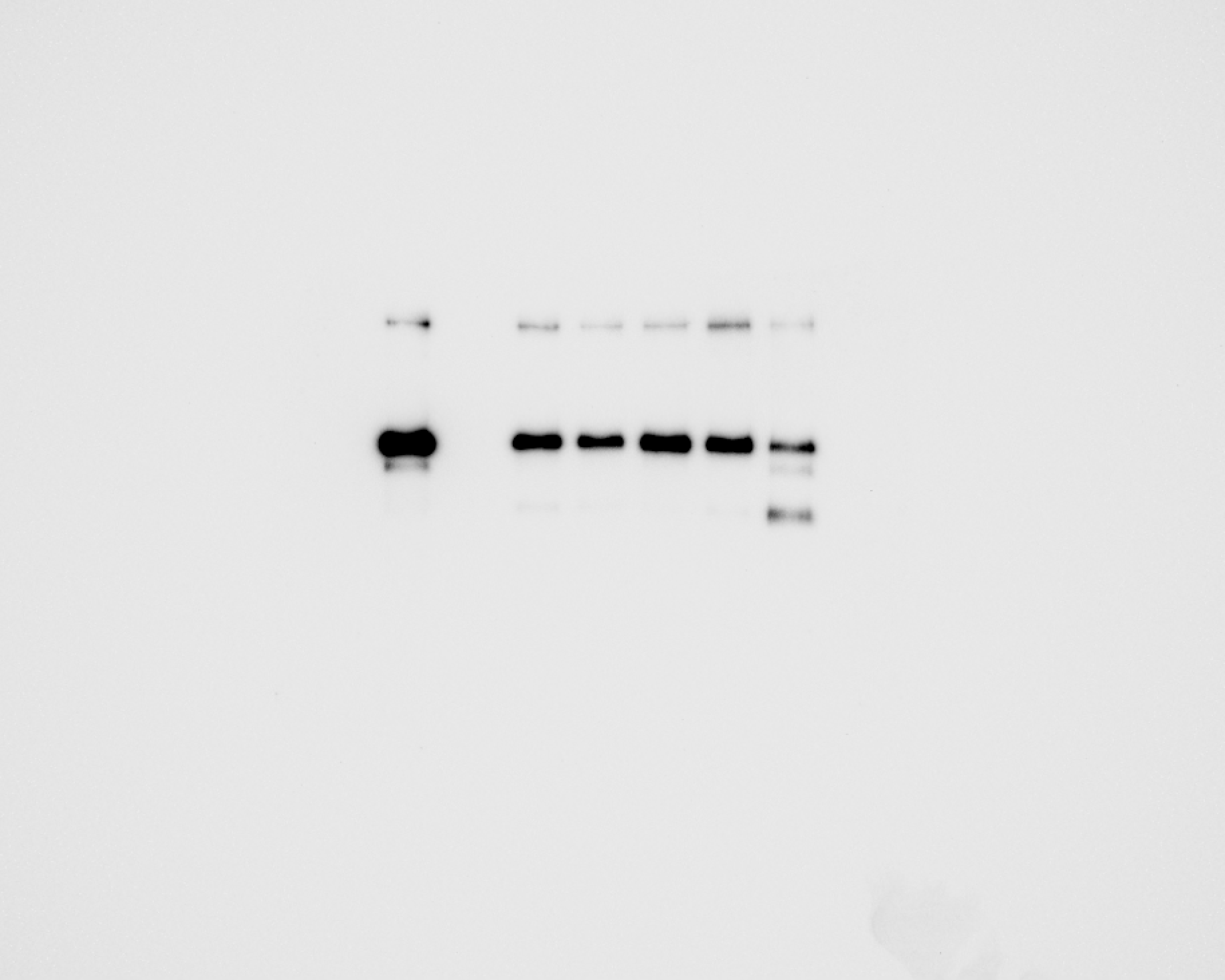

Supplement: Figure 3—source data 1. [file elife-96979-fig3-data1.zip › Figure 3_ source data/Raw unedited gels for (Figure 3)/Figure 3.B1/ChemiDoc_Anti-FLAG M2/aog 2023-11-01 15h26m34s(Chemiluminescence).tif]

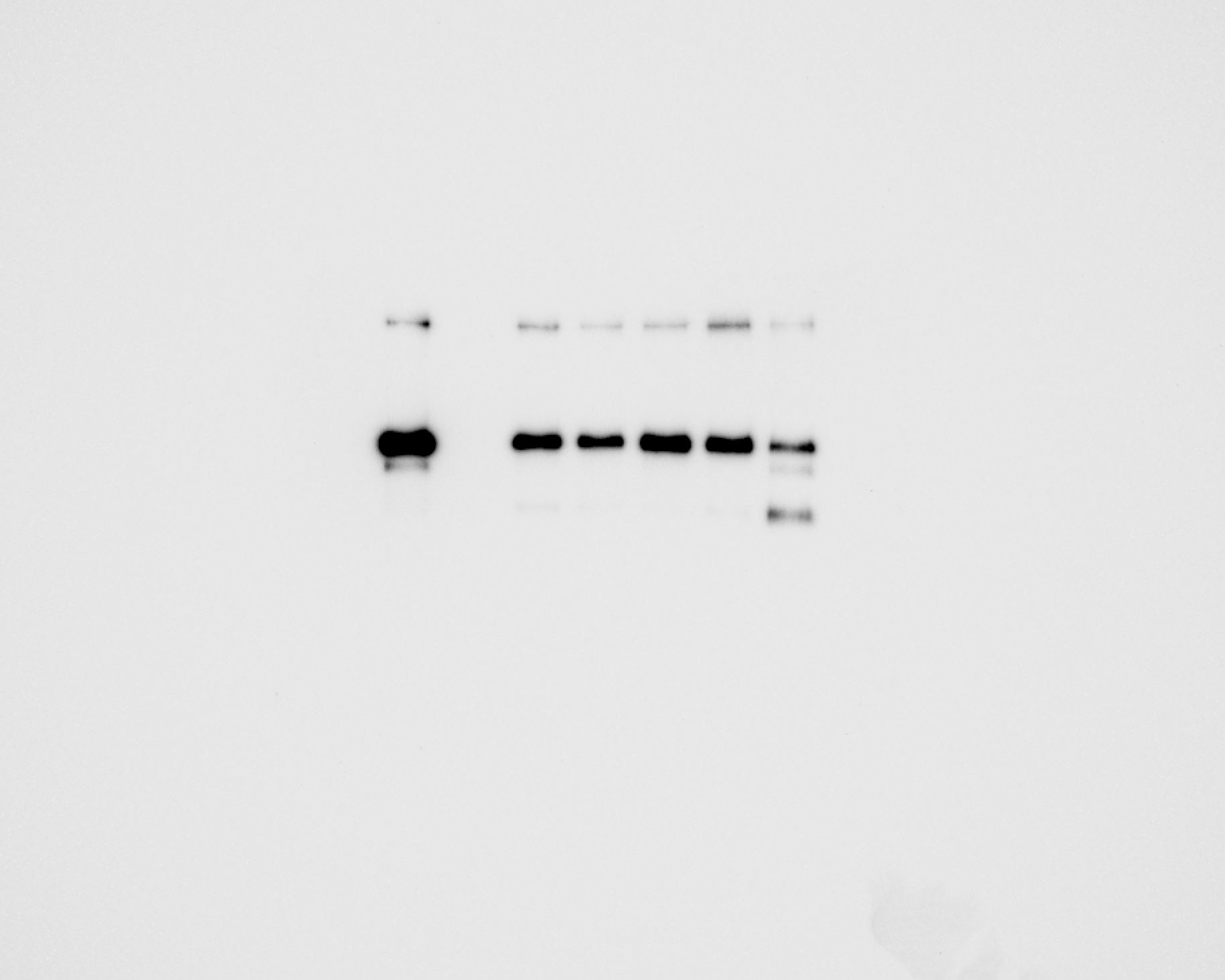

Supplement: Figure 3—source data 1. [file elife-96979-fig3-data1.zip › Figure 3_ source data/Raw unedited gels for (Figure 3)/Figure 3.B1/ChemiDoc_Anti-FLAG M2/aog 2023-11-01 15h26m34s(Chemiluminescence).jpg]

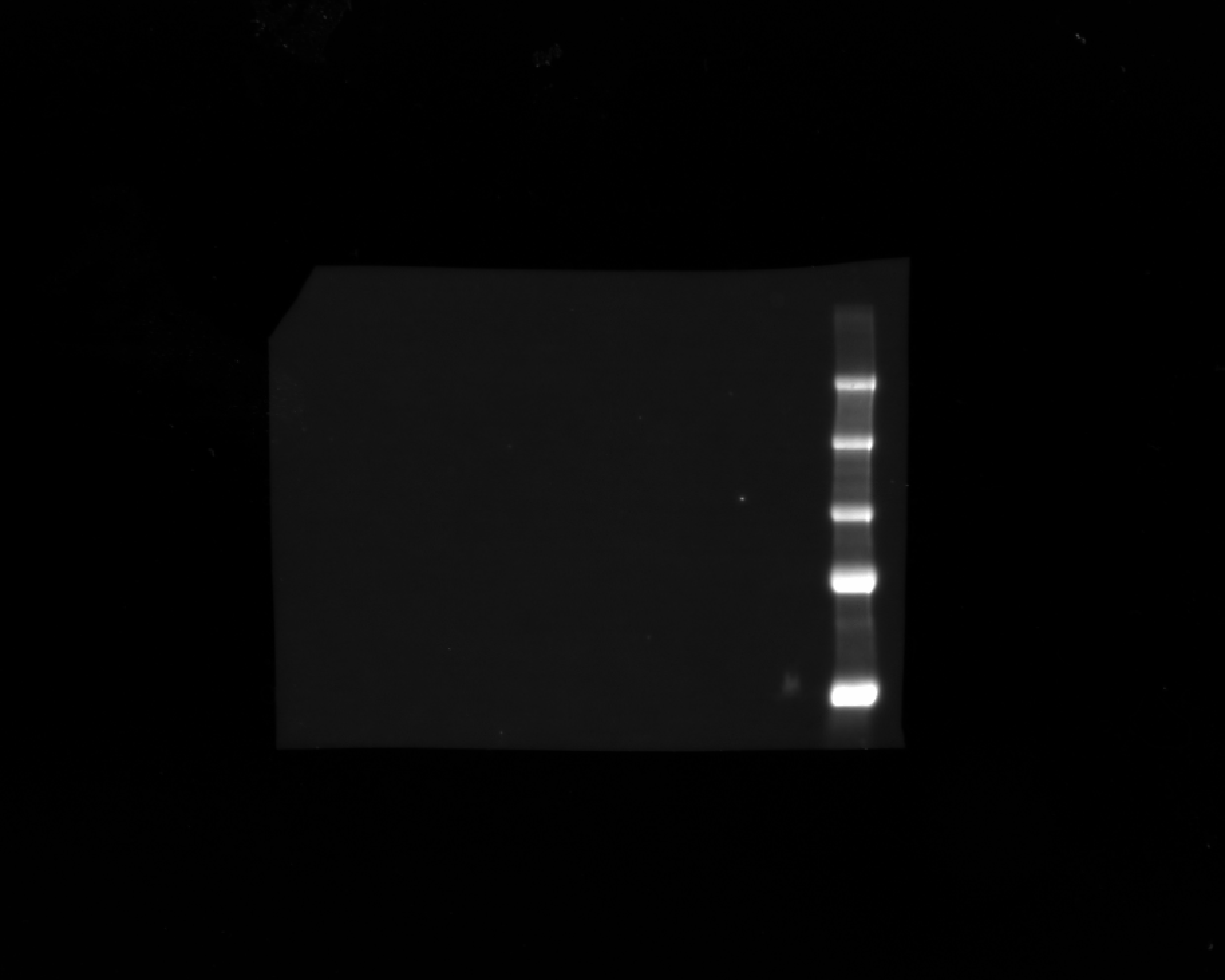

Supplement: Figure 3—source data 1. [file elife-96979-fig3-data1.zip › Figure 3_ source data/Raw unedited gels for (Figure 3)/Figure 3.B1/ChemiDoc_Anti-FLAG M2/aog 2023-11-01 15h27m15s(Coomassie Blue).tif]

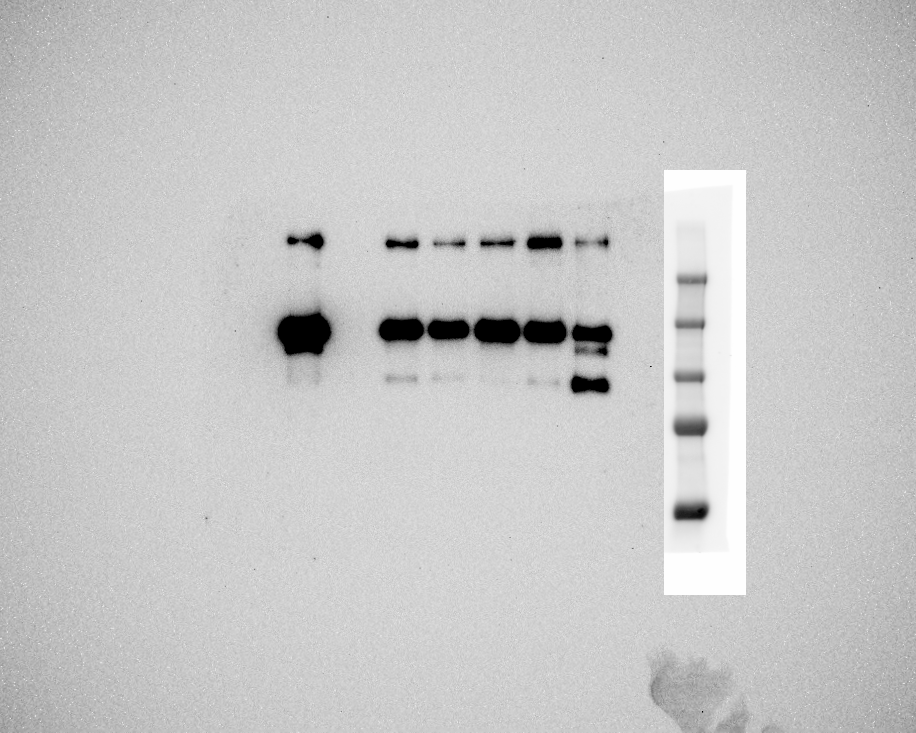

Supplement: Figure 3—source data 1. [file elife-96979-fig3-data1.zip › Figure 3_ source data/Raw unedited gels for (Figure 3)/Figure 3.B1/ChemiDoc_Anti-FLAG M2/aog 2023-11-01 15h26m34s(Chemiluminescence)_Modified.raw16.tif]

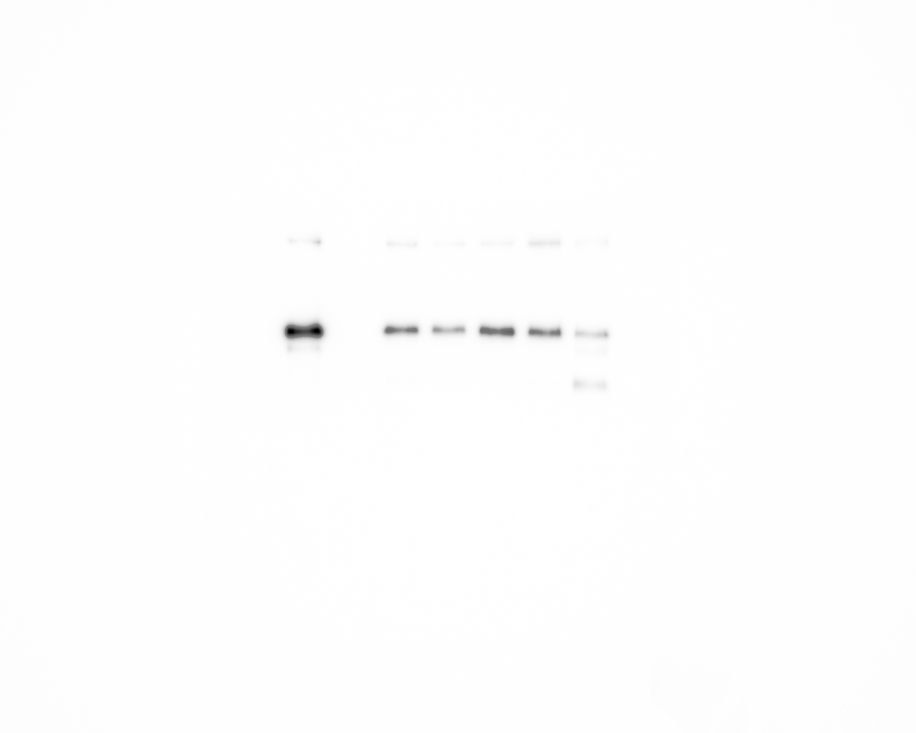

Supplement: Figure 3—source data 1. [file elife-96979-fig3-data1.zip › Figure 3_ source data/Raw unedited gels for (Figure 3)/Figure 3.B1/ChemiDoc_Anti-FLAG M2/aog 2023-11-01 15h26m34s(Chemiluminescence).raw16.tif]

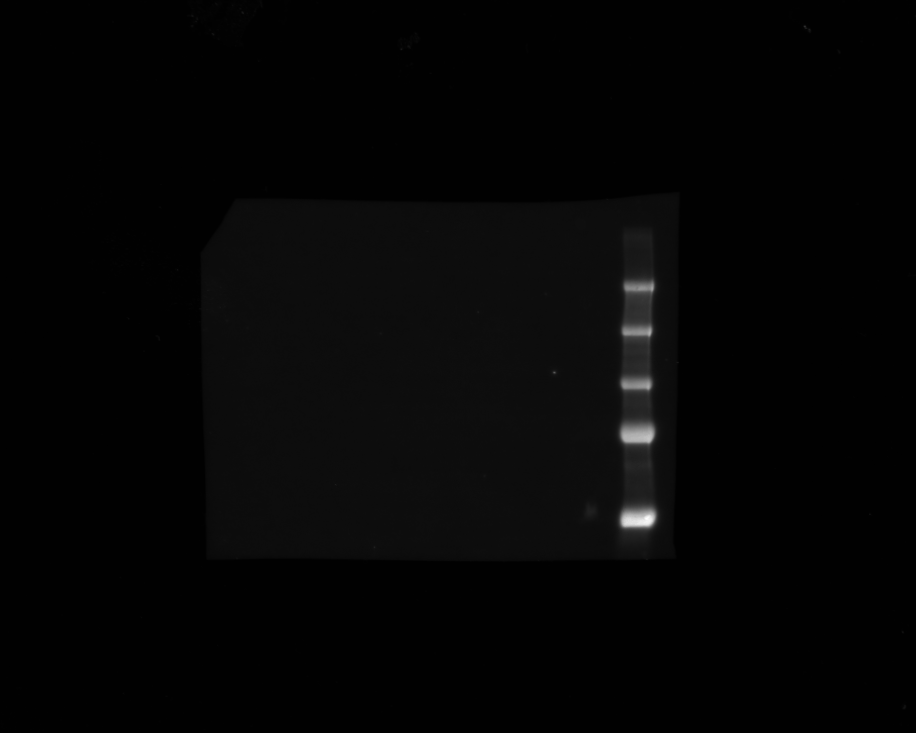

Supplement: Figure 3—source data 1. [file elife-96979-fig3-data1.zip › Figure 3_ source data/Raw unedited gels for (Figure 3)/Figure 3.B1/ChemiDoc_Anti-FLAG M2/aog 2023-11-01 15h27m15s(Coomassie Blue).raw16.tif]

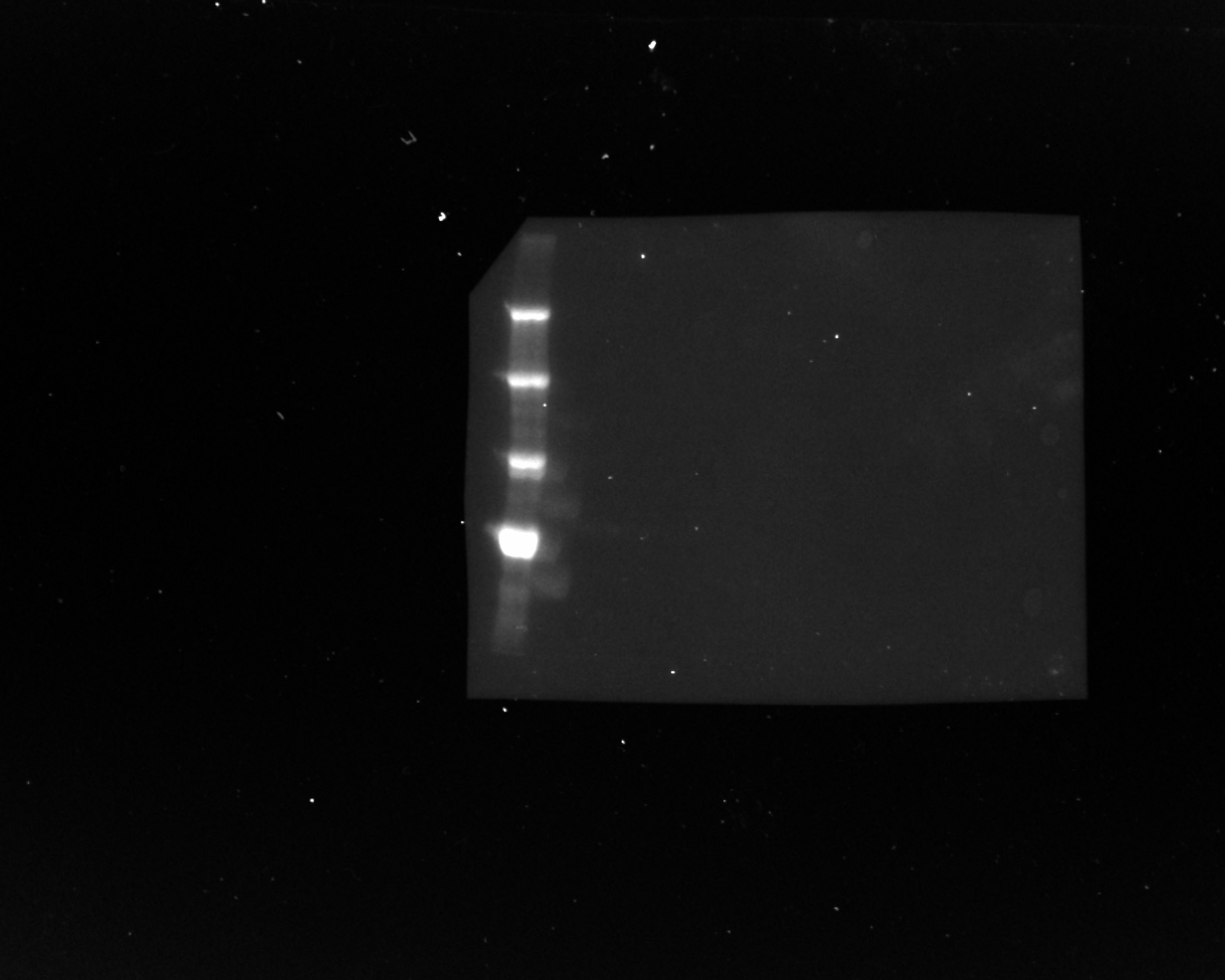

Supplement: Figure 3—source data 1. [file elife-96979-fig3-data1.zip › Figure 3_ source data/Raw unedited gels for (Figure 3)/Figure 3.B2/anti-FLAgM2 ChemiDoc/aog 2023-11-22 12h28m11s(Coomassie Blue).jpg]

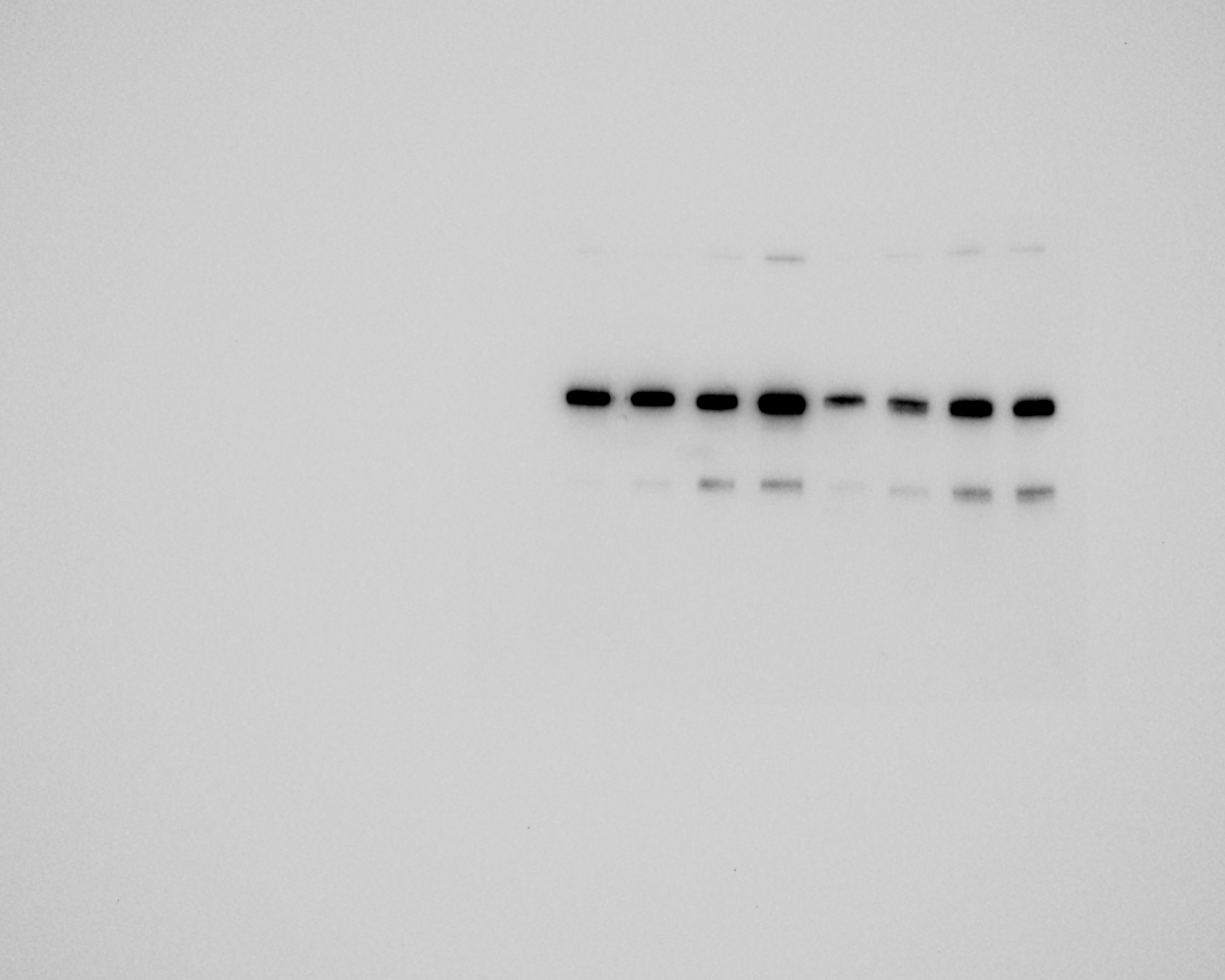

Supplement: Figure 3—source data 1. [file elife-96979-fig3-data1.zip › Figure 3_ source data/Raw unedited gels for (Figure 3)/Figure 3.B2/anti-FLAgM2 ChemiDoc/aog 2023-11-22 12h27m28s(Chemiluminescence).tif]

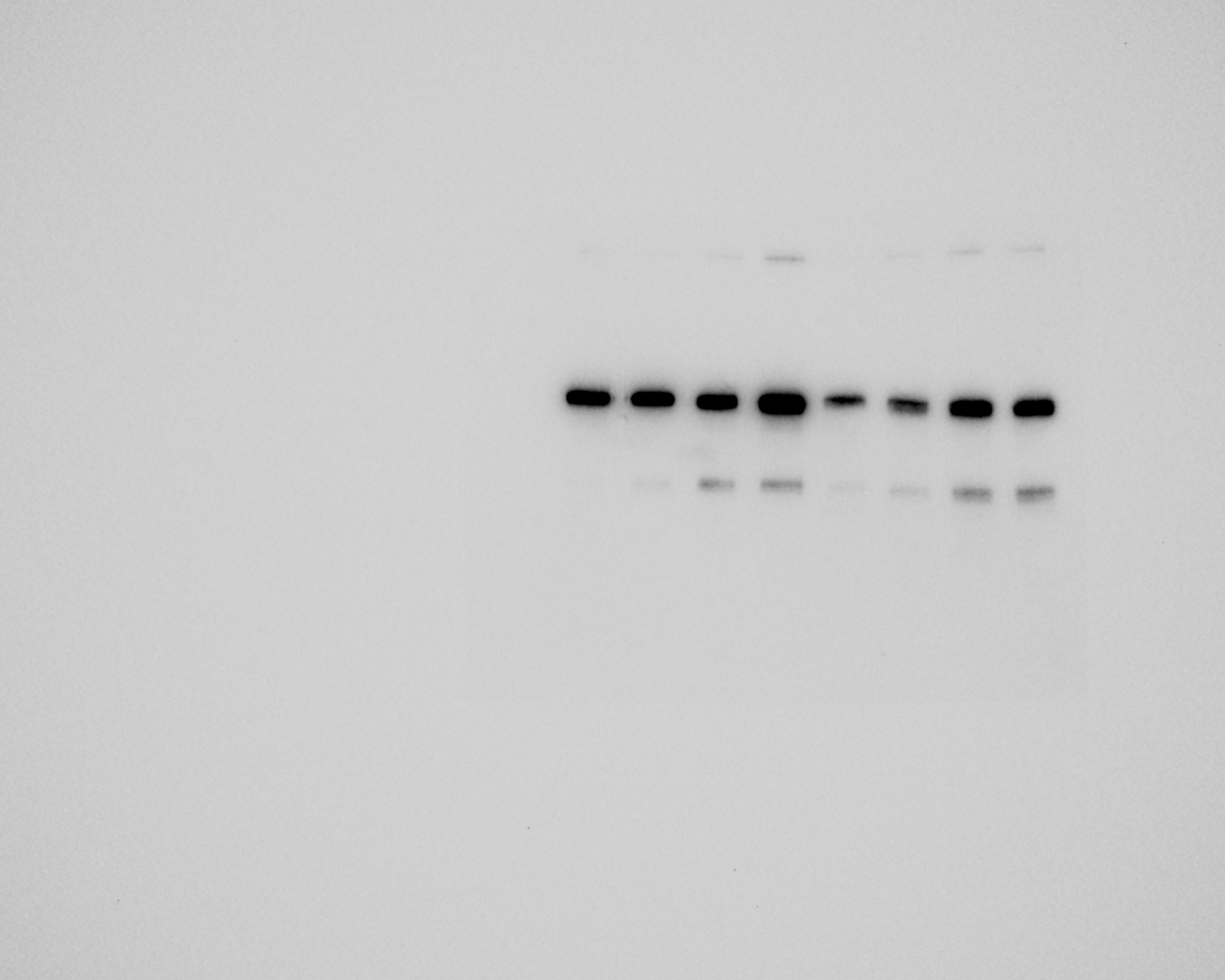

Supplement: Figure 3—source data 1. [file elife-96979-fig3-data1.zip › Figure 3_ source data/Raw unedited gels for (Figure 3)/Figure 3.B2/anti-FLAgM2 ChemiDoc/aog 2023-11-22 12h27m28s(Chemiluminescence).jpg]

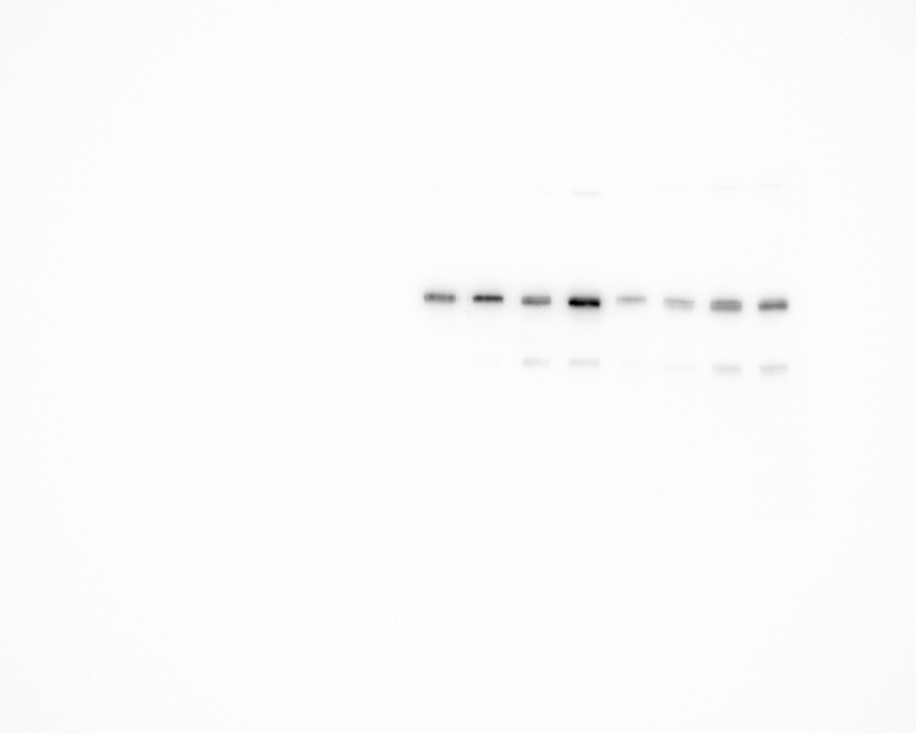

Supplement: Figure 3—source data 1. [file elife-96979-fig3-data1.zip › Figure 3_ source data/Raw unedited gels for (Figure 3)/Figure 3.B2/anti-FLAgM2 ChemiDoc/aog 2023-11-22 12h27m28s(Chemiluminescence).raw16.tif]

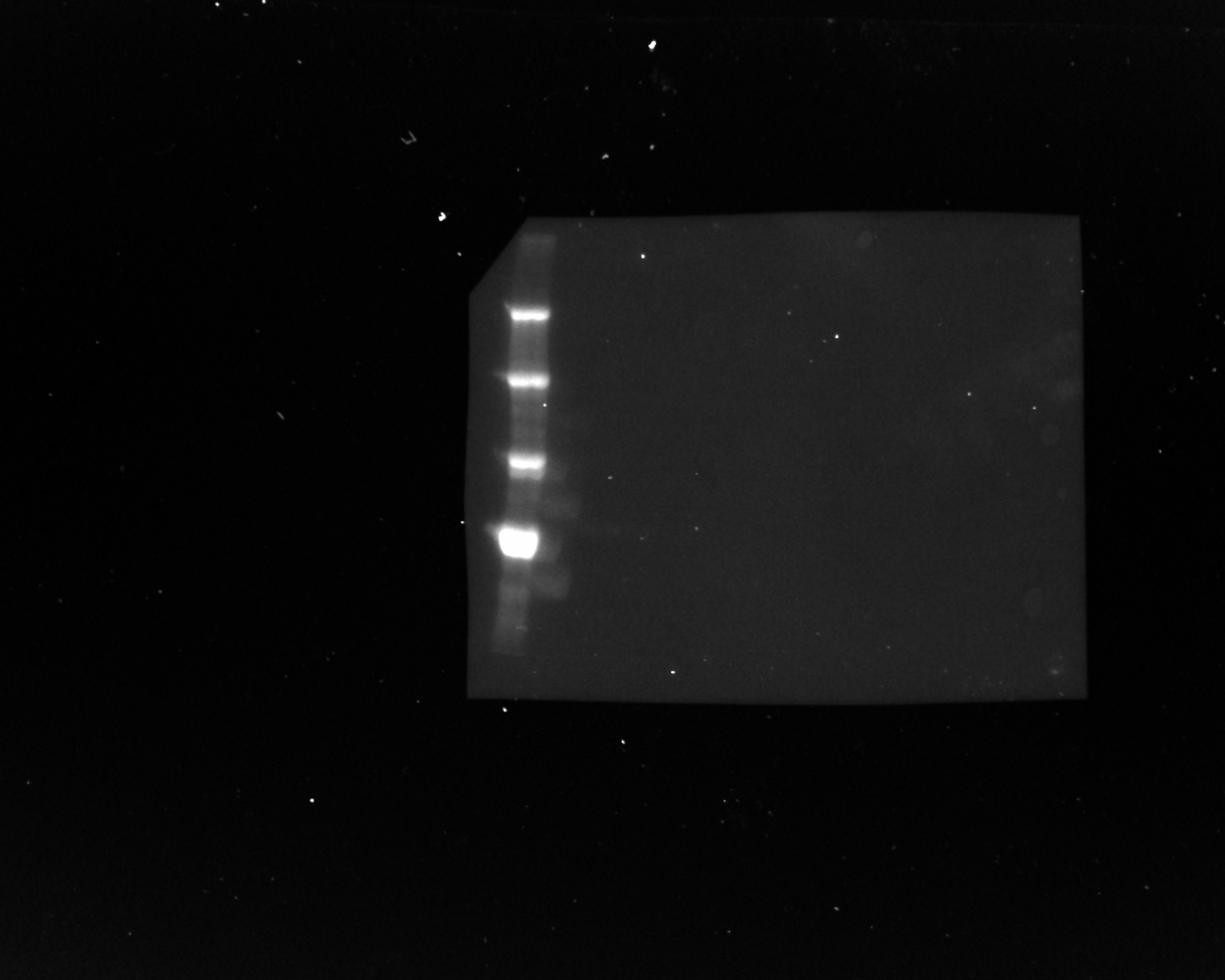

Supplement: Figure 3—source data 1. [file elife-96979-fig3-data1.zip › Figure 3_ source data/Raw unedited gels for (Figure 3)/Figure 3.B2/anti-FLAgM2 ChemiDoc/aog 2023-11-22 12h28m11s(Coomassie Blue).tif]

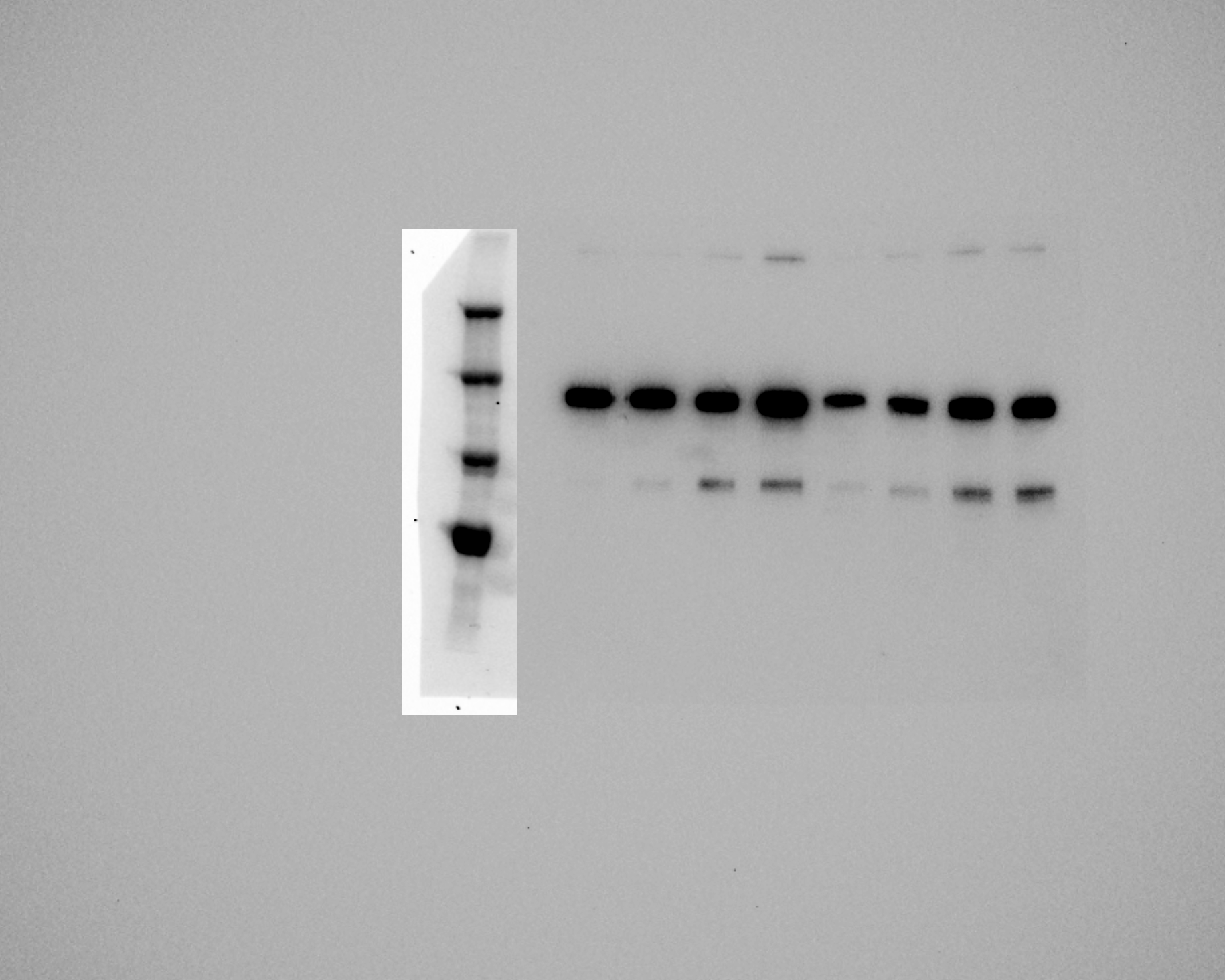

Supplement: Figure 3—source data 1. [file elife-96979-fig3-data1.zip › Figure 3_ source data/Raw unedited gels for (Figure 3)/Figure 3.B2/anti-FLAgM2 ChemiDoc/aog 2023-11-22 12h27m28s(Chemiluminescence)_modified.tif]

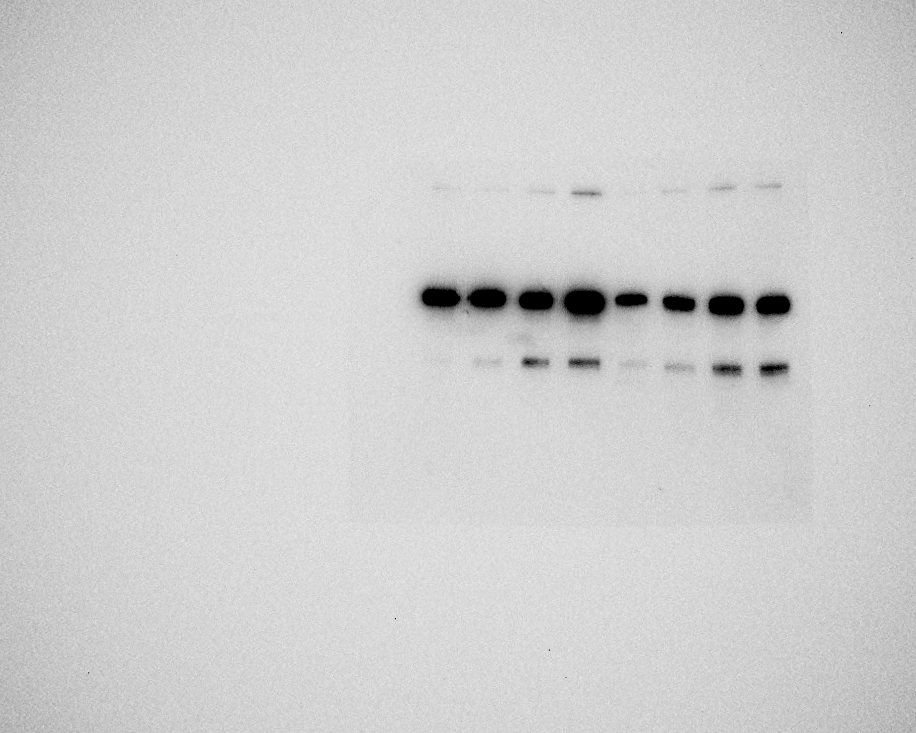

Supplement: Figure 3—source data 1. [file elife-96979-fig3-data1.zip › Figure 3_ source data/Raw unedited gels for (Figure 3)/Figure 3.B2/anti-FLAgM2 ChemiDoc/aog 2023-11-22 12h27m28s(Chemiluminescence)_Modified.raw16.tif]

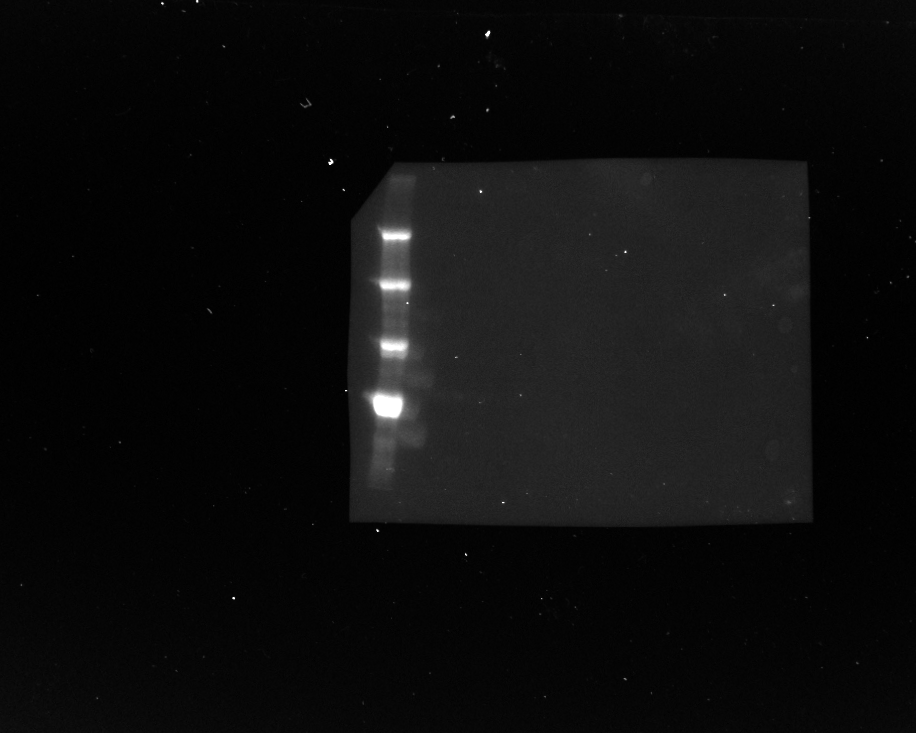

Supplement: Figure 3—source data 1. [file elife-96979-fig3-data1.zip › Figure 3_ source data/Raw unedited gels for (Figure 3)/Figure 3.B2/anti-FLAgM2 ChemiDoc/aog 2023-11-22 12h28m11s(Coomassie Blue).raw16.tif]

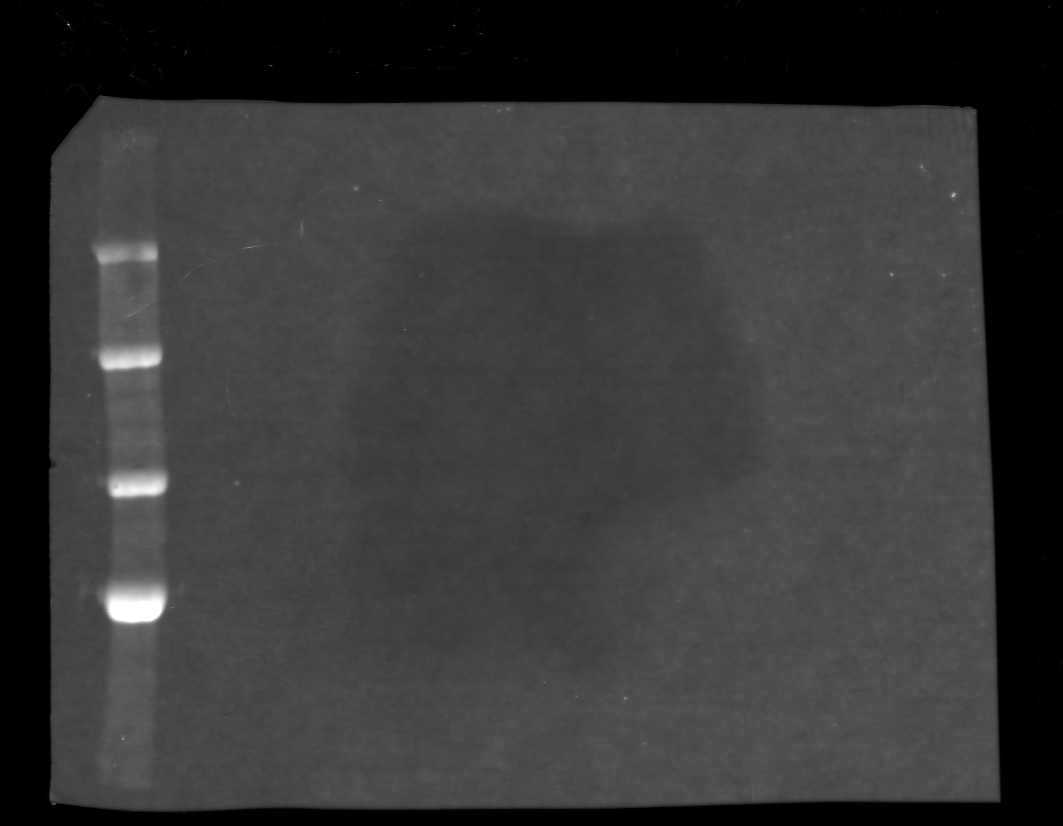

Supplement: Figure 3—source data 1. [file elife-96979-fig3-data1.zip › Figure 3_ source data/Raw unedited gels for (Figure 3)/Figure 3.B2/Anti-Tublin rabbit/2023-11-23-135305/700.TIF]

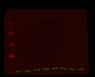

Supplement: Figure 3—source data 1. [file elife-96979-fig3-data1.zip › Figure 3_ source data/Raw unedited gels for (Figure 3)/Figure 3.B2/Anti-Tublin rabbit/2023-11-23-135305/2023-11-23-135305_1_TH.jpg]

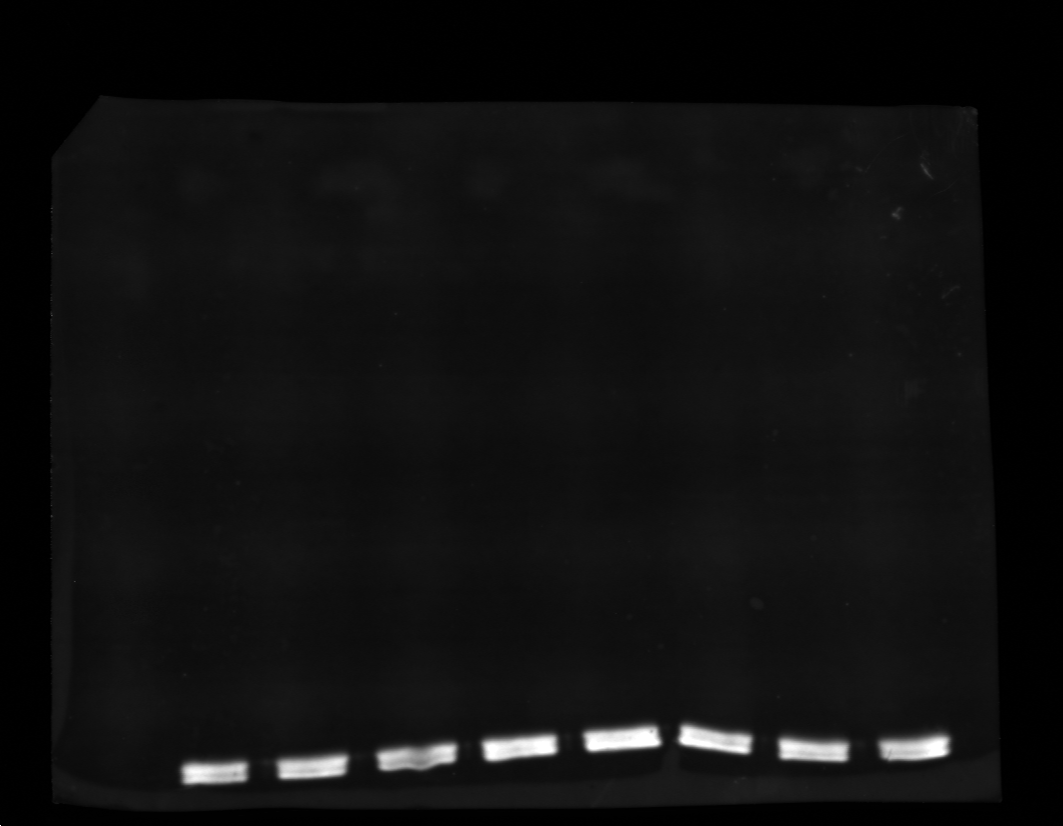

Supplement: Figure 3—source data 1. [file elife-96979-fig3-data1.zip › Figure 3_ source data/Raw unedited gels for (Figure 3)/Figure 3.B2/Anti-Tublin rabbit/2023-11-23-135305/800.TIF]

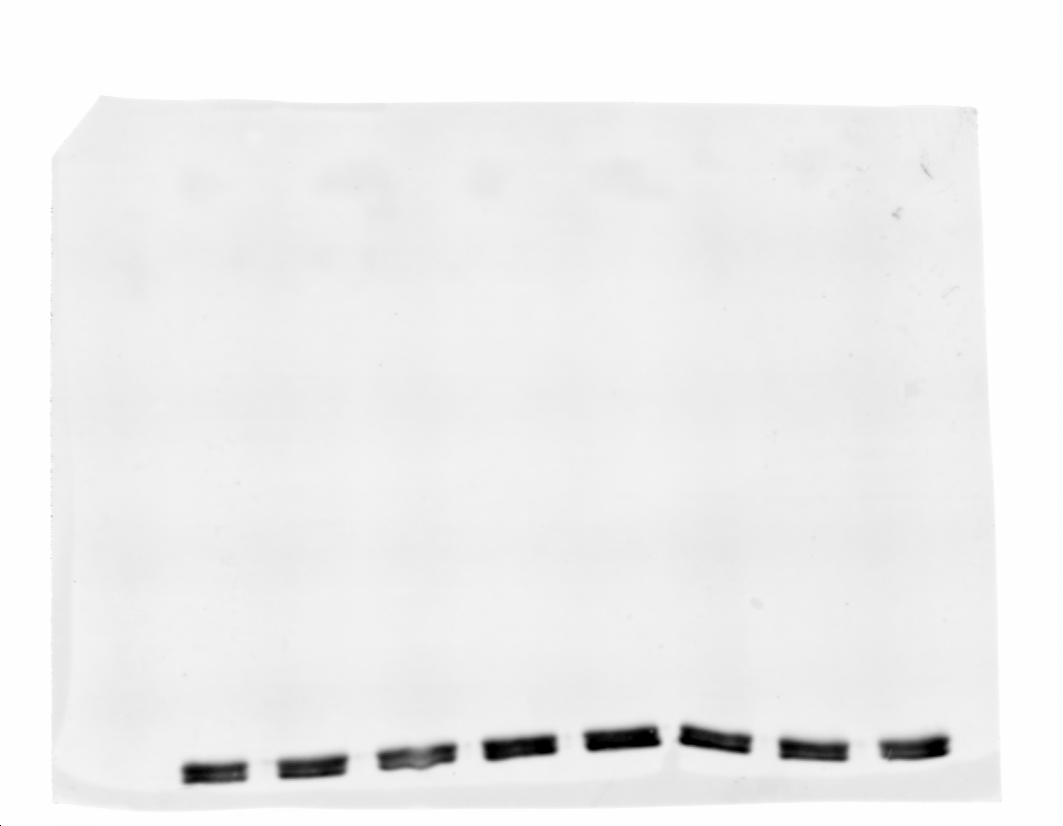

Supplement: Figure 3—source data 1. [file elife-96979-fig3-data1.zip › Figure 3_ source data/Raw unedited gels for (Figure 3)/Figure 3.B2/Anti-Tublin rabbit/2023-11-23-135305/800_modified.tif]

Uncropped and labelled gels for Figure 3\_figure supplement 1

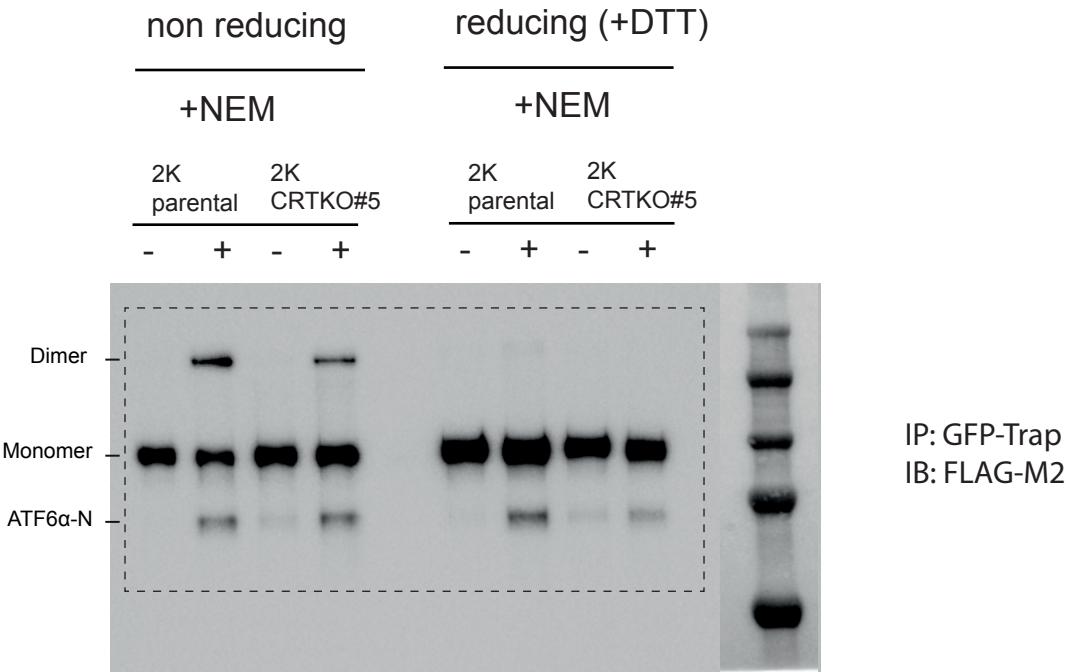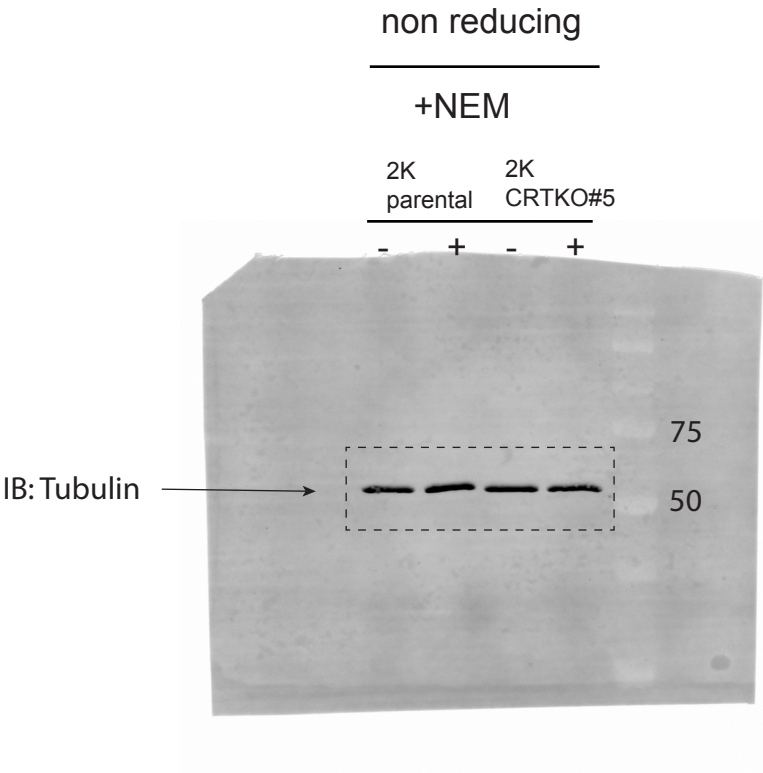

Supplement: Figure 3—figure supplement 1—source code 1. [file elife-96979-fig3-figsupp1-code1.zip › Figure 3_figure supplement 1_source data /Uncropped and labelled gels for (Figure 3_figure supplement 1).pdf]

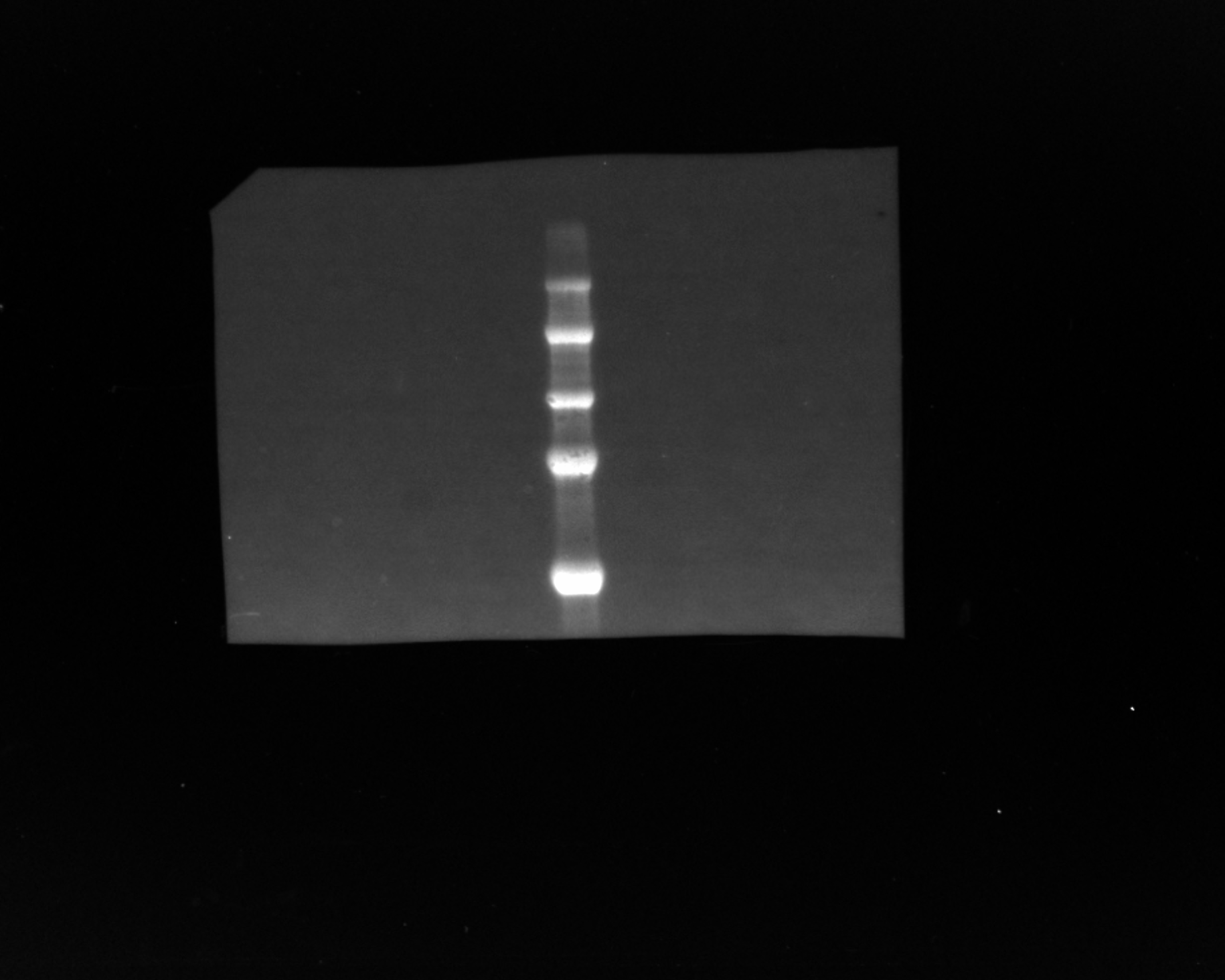

Supplement: Figure 3—figure supplement 1—source code 1. [file elife-96979-fig3-figsupp1-code1.zip › Figure 3_figure supplement 1_source data /Raw unedited gels for (Figure 3-figure supplement 1)/anti-FLAGM2 HRP/aog 2024-05-21 14h25m19s(Coomassie Blue).tif]

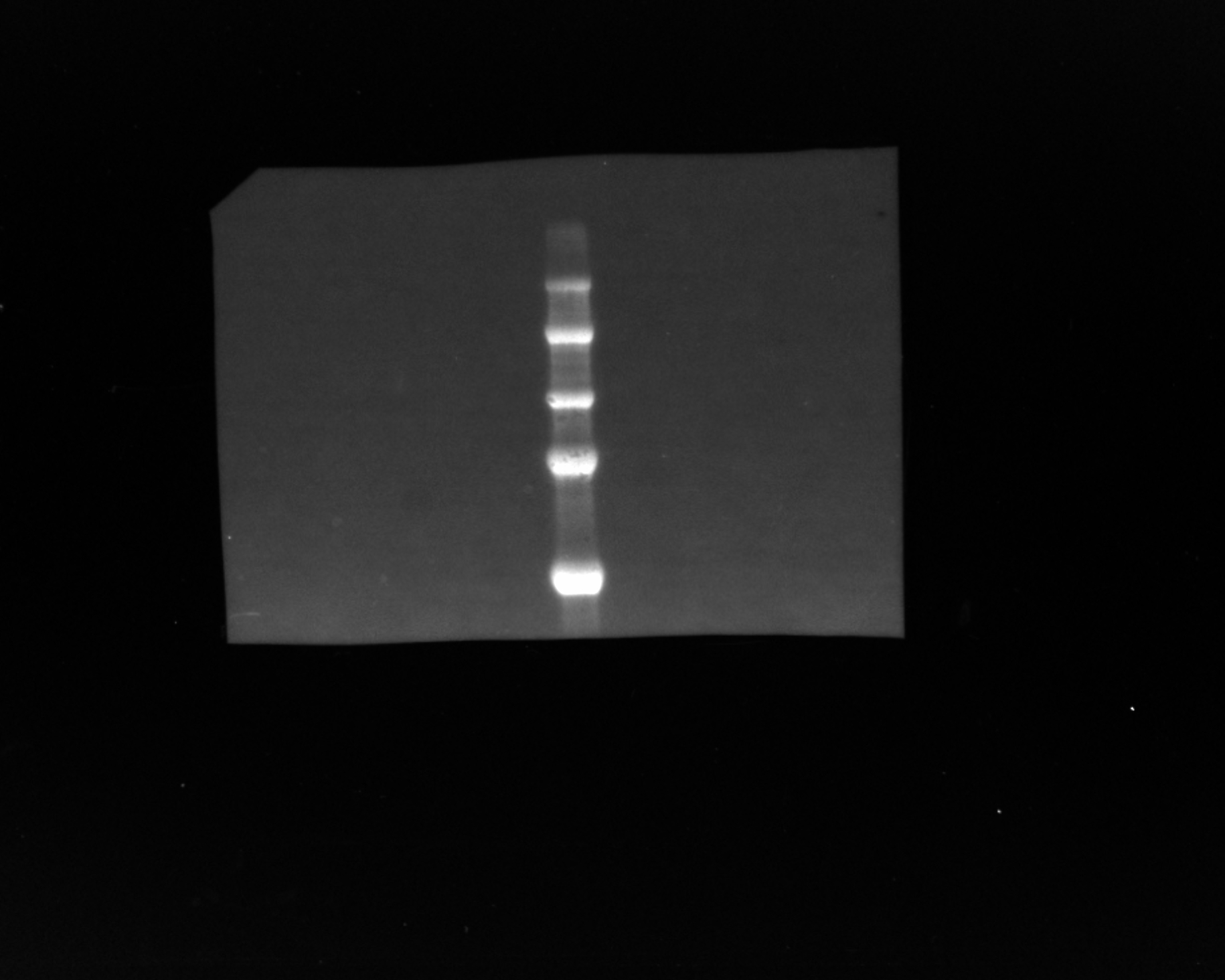

Supplement: Figure 3—figure supplement 1—source code 1. [file elife-96979-fig3-figsupp1-code1.zip › Figure 3_figure supplement 1_source data /Raw unedited gels for (Figure 3-figure supplement 1)/anti-FLAGM2 HRP/aog 2024-05-21 14h25m19s(Coomassie Blue).jpg]

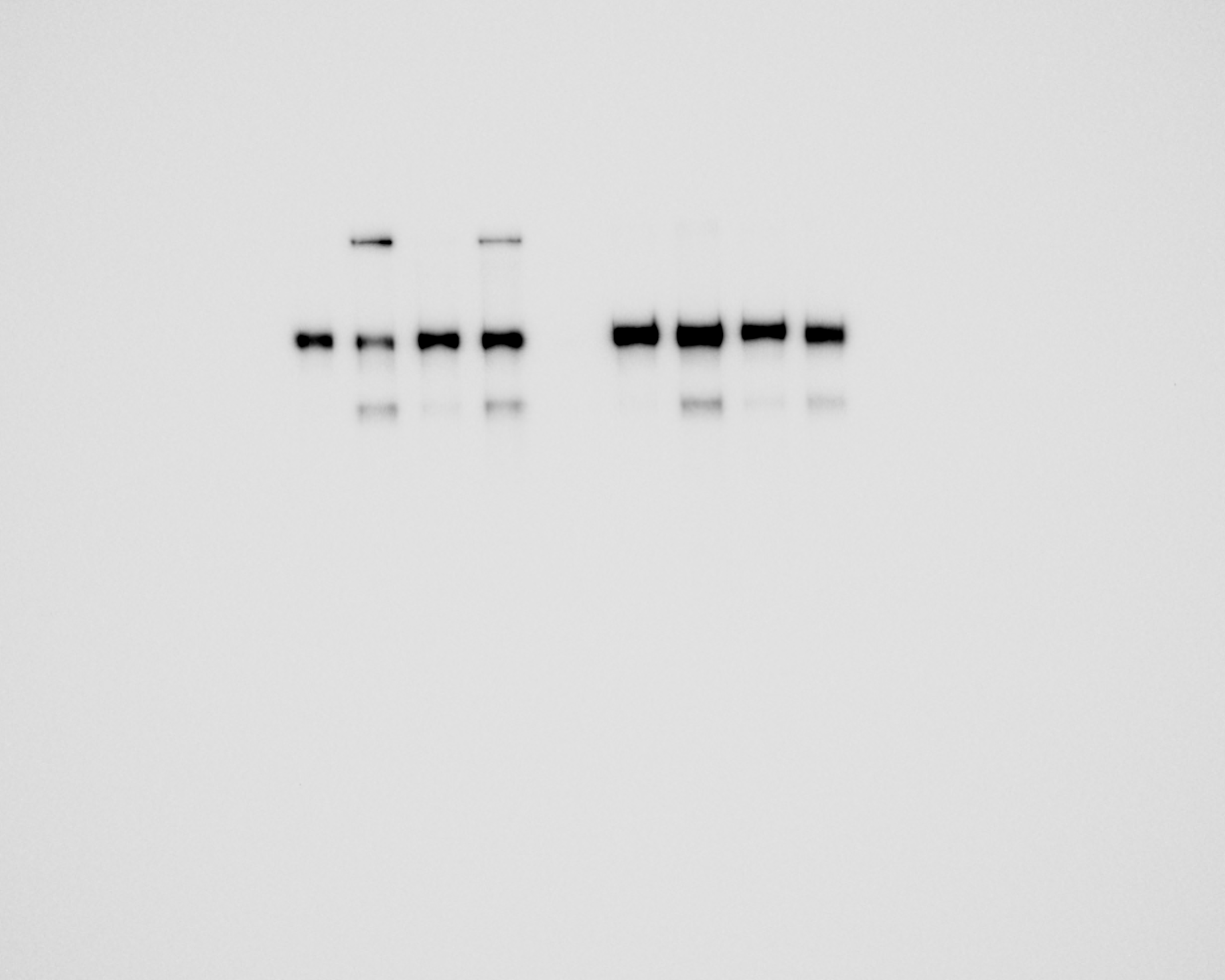

Supplement: Figure 3—figure supplement 1—source code 1. [file elife-96979-fig3-figsupp1-code1.zip › Figure 3_figure supplement 1_source data /Raw unedited gels for (Figure 3-figure supplement 1)/anti-FLAGM2 HRP/aog 2024-05-21 14h23m59s(Chemiluminescence).tif]

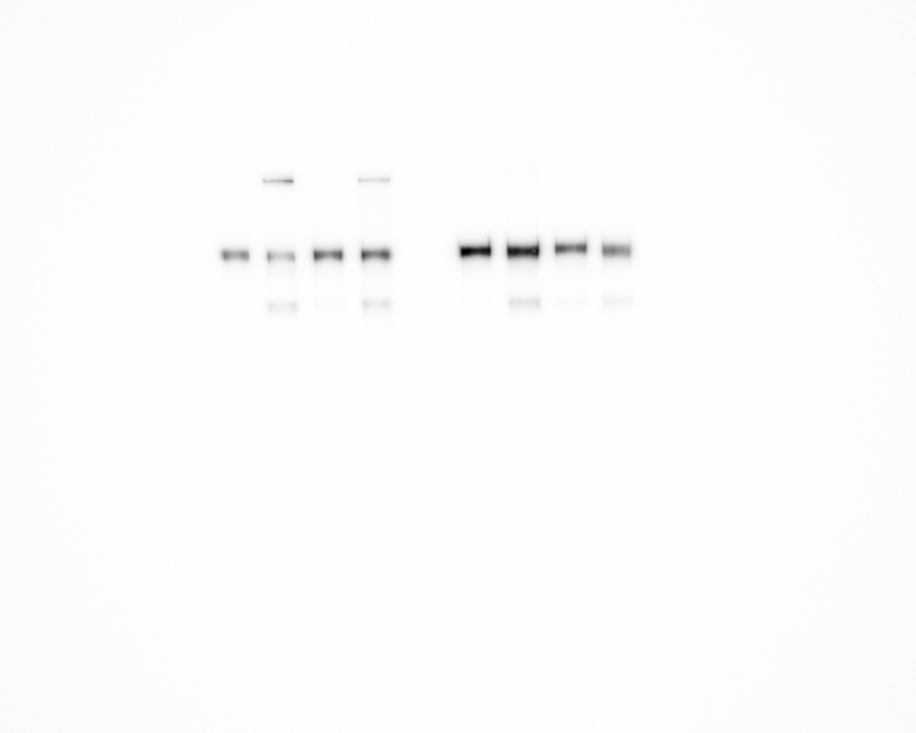

Supplement: Figure 3—figure supplement 1—source code 1. [file elife-96979-fig3-figsupp1-code1.zip › Figure 3_figure supplement 1_source data /Raw unedited gels for (Figure 3-figure supplement 1)/anti-FLAGM2 HRP/aog 2024-05-21 14h23m59s(Chemiluminescence).raw16.tif]

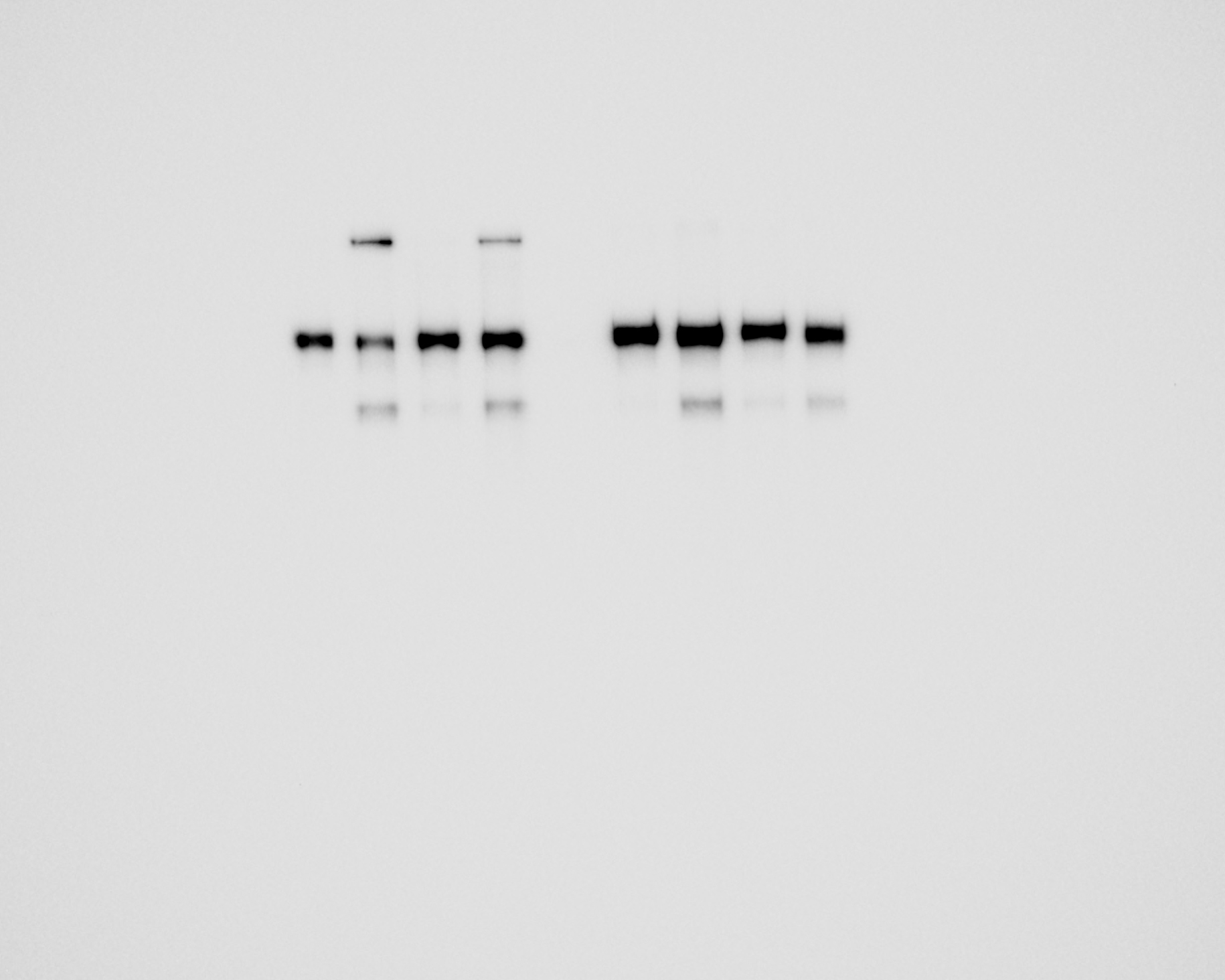

Supplement: Figure 3—figure supplement 1—source code 1. [file elife-96979-fig3-figsupp1-code1.zip › Figure 3_figure supplement 1_source data /Raw unedited gels for (Figure 3-figure supplement 1)/anti-FLAGM2 HRP/aog 2024-05-21 14h23m59s(Chemiluminescence).jpg]

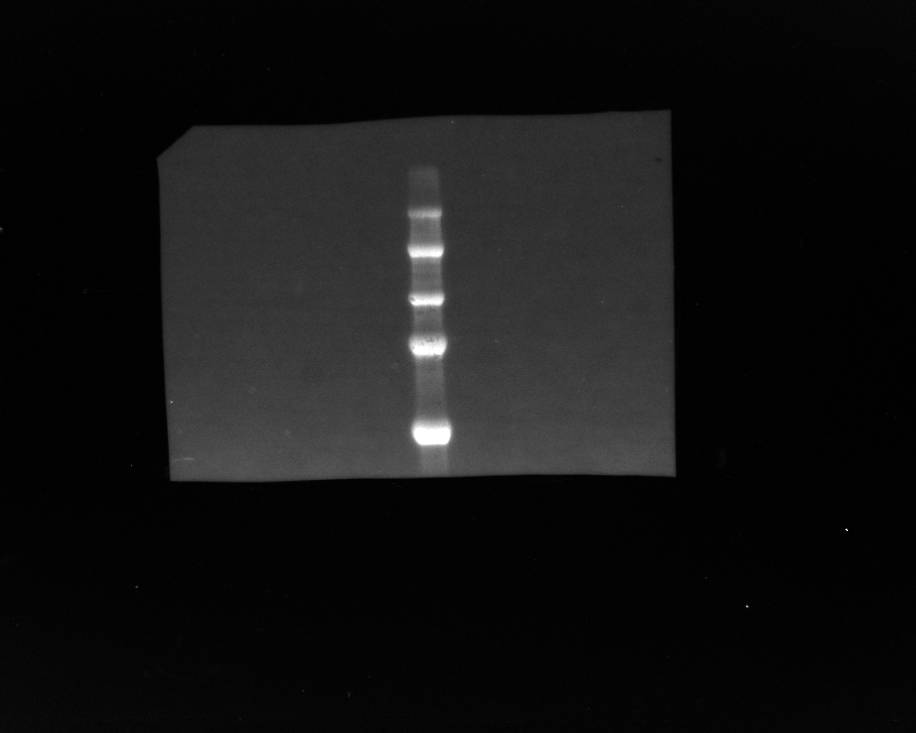

Supplement: Figure 3—figure supplement 1—source code 1. [file elife-96979-fig3-figsupp1-code1.zip › Figure 3_figure supplement 1_source data /Raw unedited gels for (Figure 3-figure supplement 1)/anti-FLAGM2 HRP/aog 2024-05-21 14h25m19s(Coomassie Blue).raw16.tif]

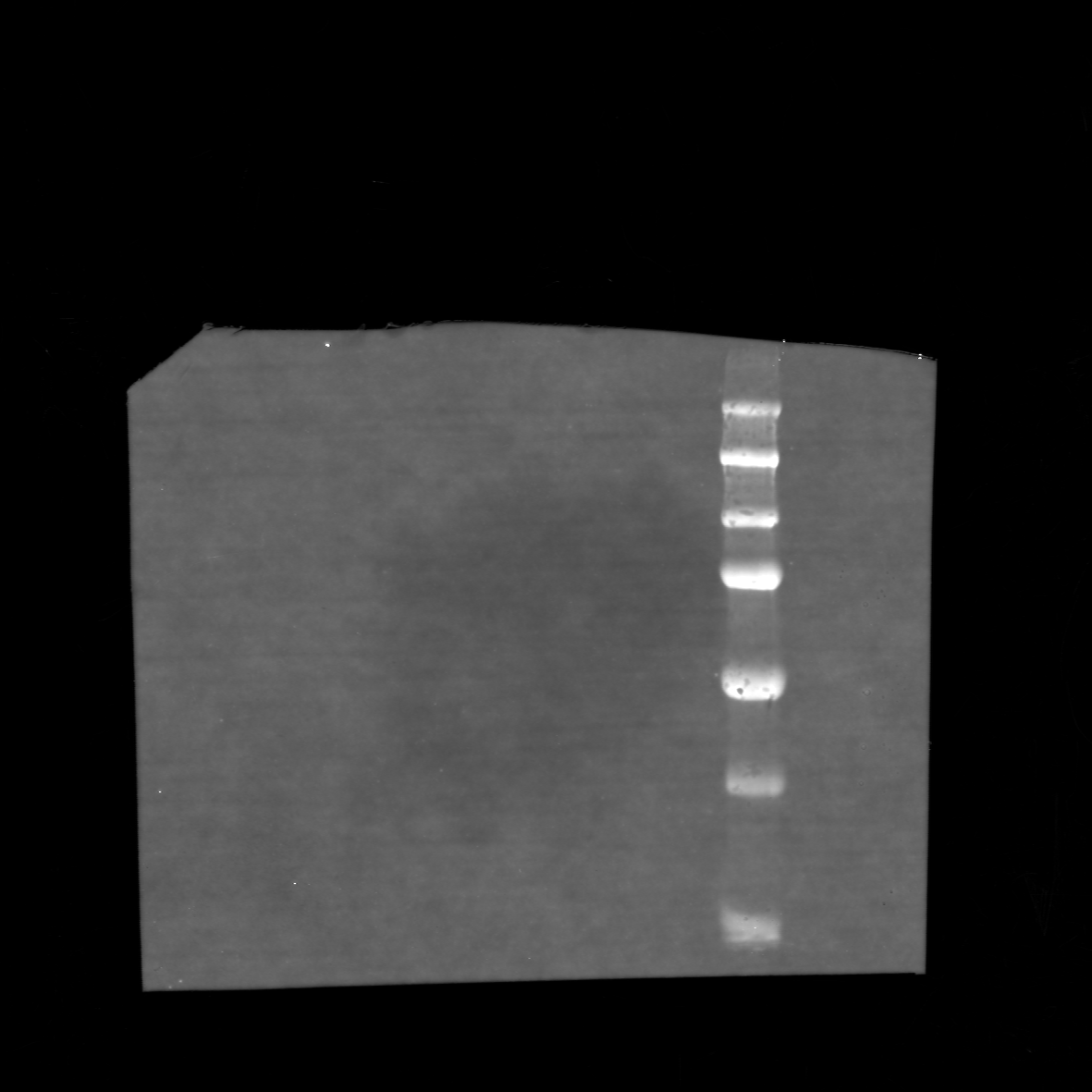

Supplement: Figure 3—figure supplement 1—source code 1. [file elife-96979-fig3-figsupp1-code1.zip › Figure 3_figure supplement 1_source data /Raw unedited gels for (Figure 3-figure supplement 1)/tubulin-licor/2024-05-28-142856/700.TIF]

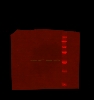

Supplement: Figure 3—figure supplement 1—source code 1. [file elife-96979-fig3-figsupp1-code1.zip › Figure 3_figure supplement 1_source data /Raw unedited gels for (Figure 3-figure supplement 1)/tubulin-licor/2024-05-28-142856/2024-05-28-142856_1_TH.jpg]

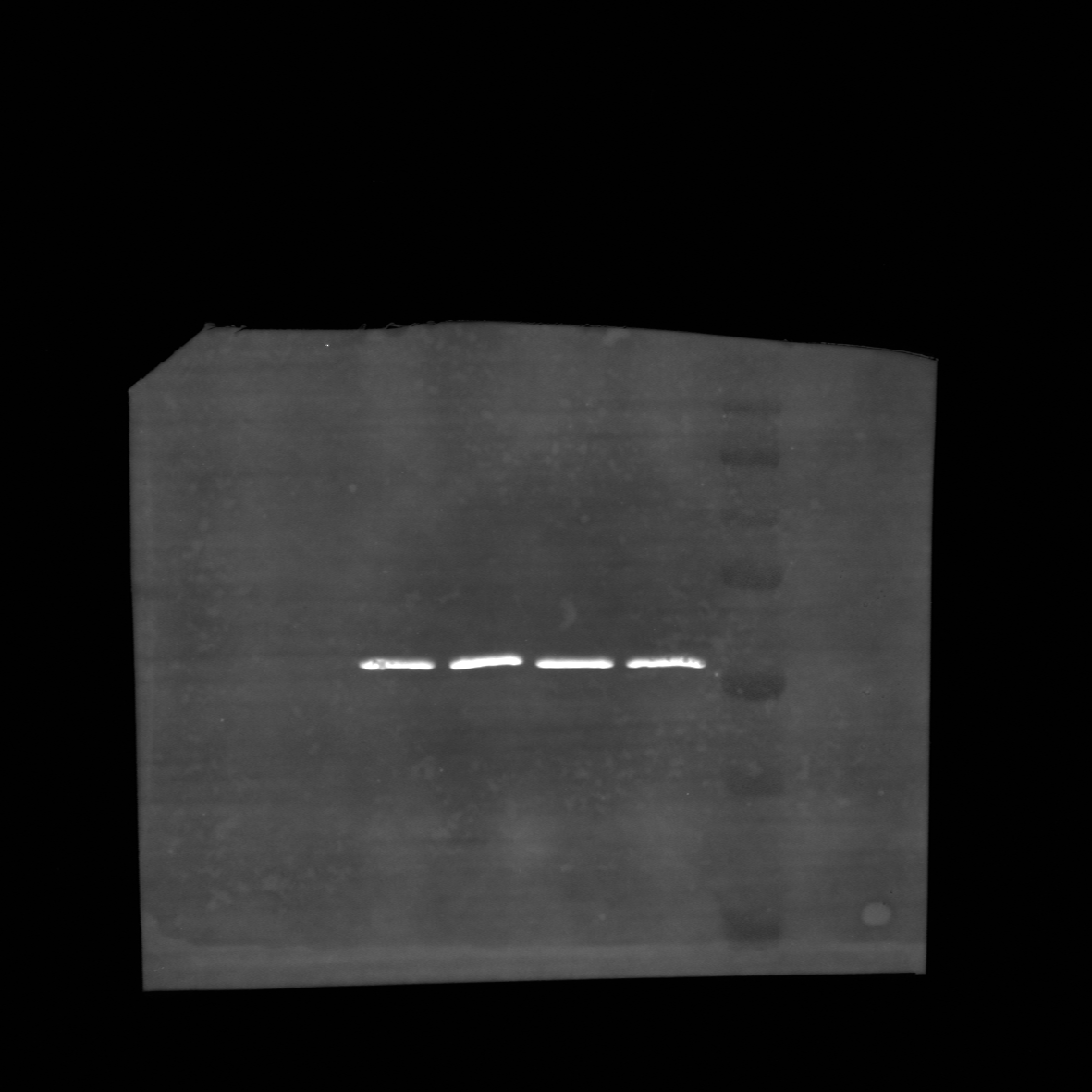

Supplement: Figure 3—figure supplement 1—source code 1. [file elife-96979-fig3-figsupp1-code1.zip › Figure 3_figure supplement 1_source data /Raw unedited gels for (Figure 3-figure supplement 1)/tubulin-licor/2024-05-28-142856/800.TIF]

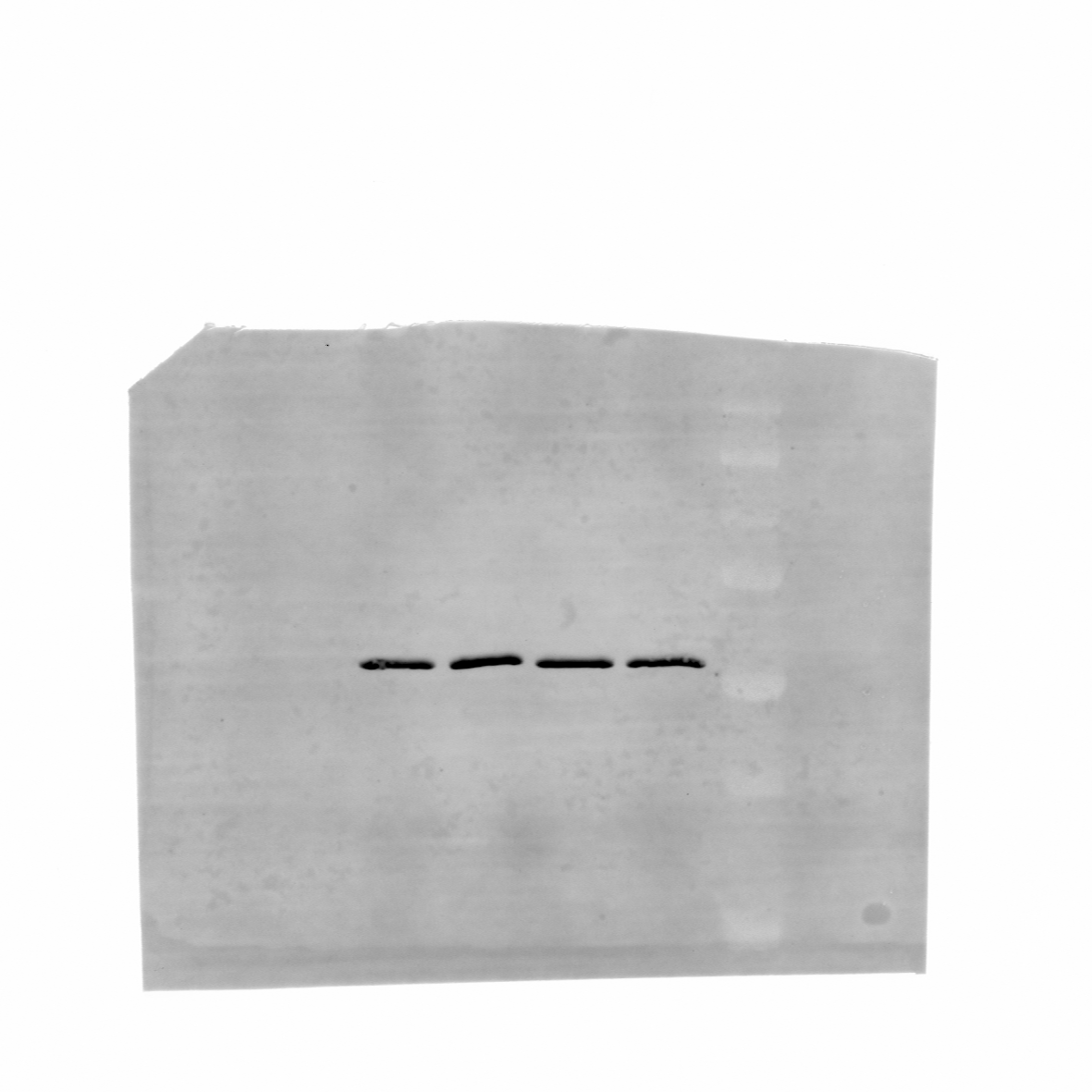

Supplement: Figure 3—figure supplement 1—source code 1. [file elife-96979-fig3-figsupp1-code1.zip › Figure 3_figure supplement 1_source data /Raw unedited gels for (Figure 3-figure supplement 1)/tubulin-licor/2024-05-28-142856/800_modified.tif]

Uncropped and labelled gels for (Figure 4)

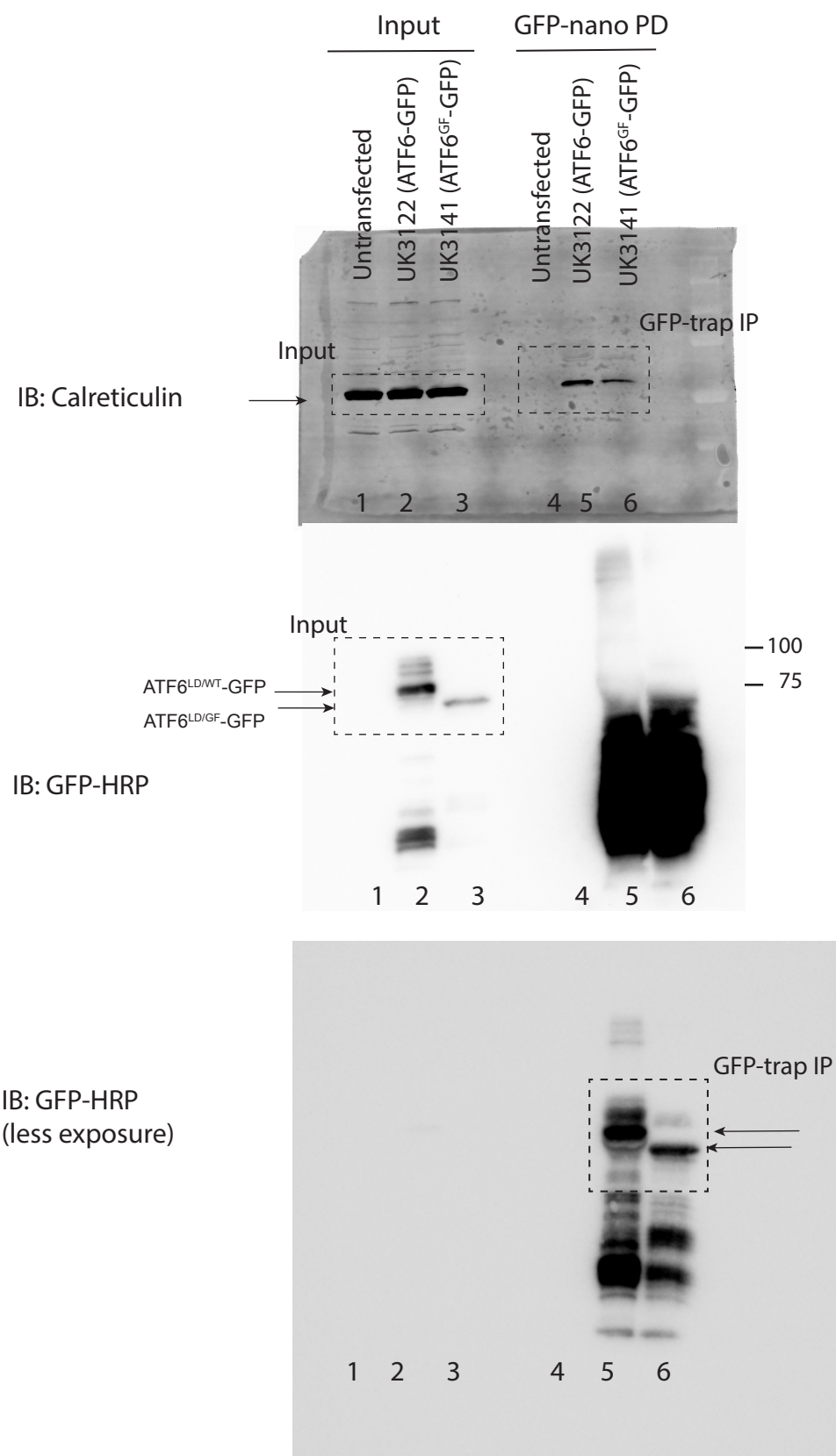

Supplement: Figure 4—source data 1. [file elife-96979-fig4-data1.zip › Figure 4_source data/Uncropped and labelled gels for (Figure 4).pdf]

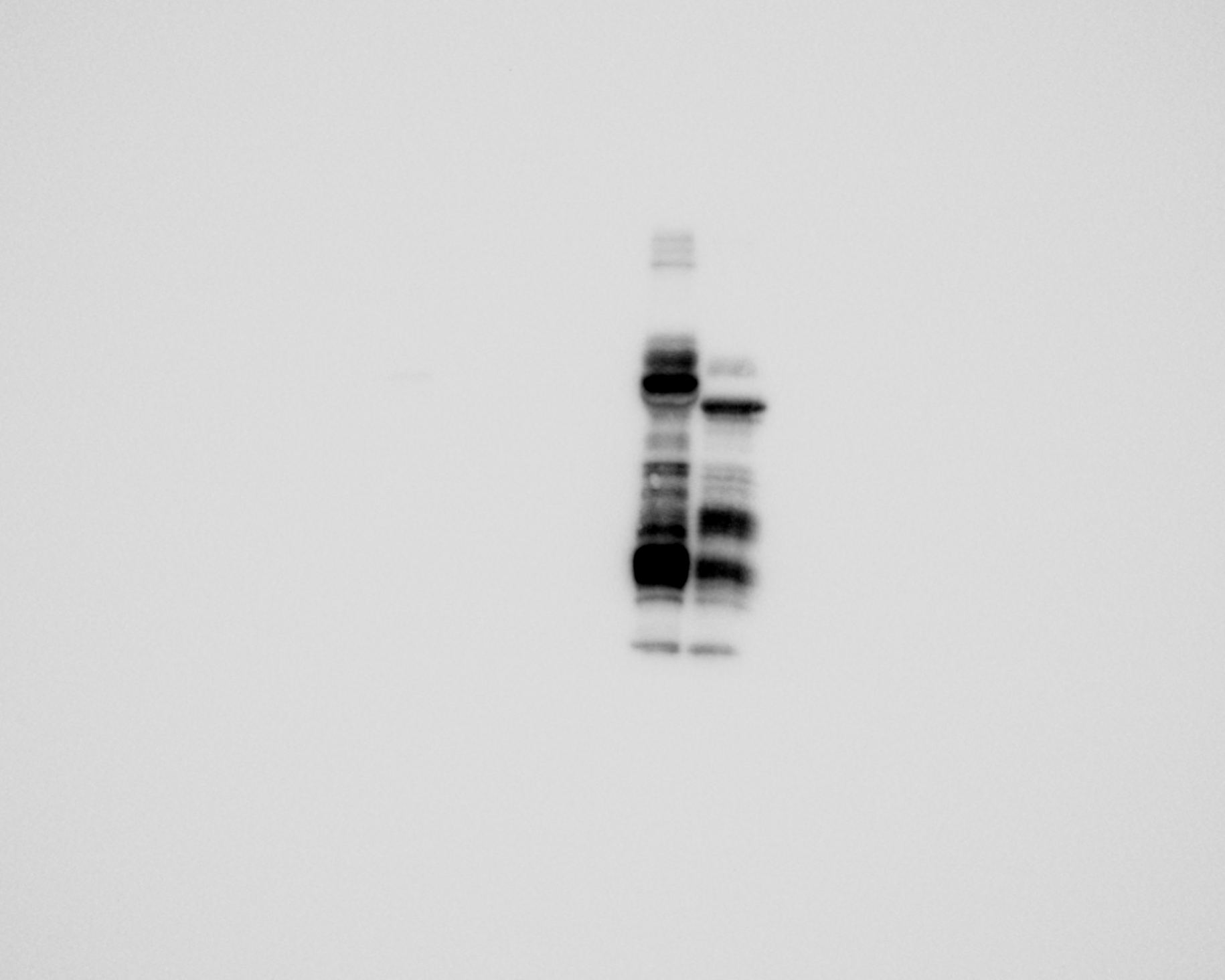

Supplement: Figure 4—source data 1. [file elife-96979-fig4-data1.zip › Figure 4_source data/Raw unedited gels for (Figure 4)/3. Anti-GFP HRP_less exposure for IP/aog 2023-07-18 10h46m35s(Chemiluminescence).jpg]

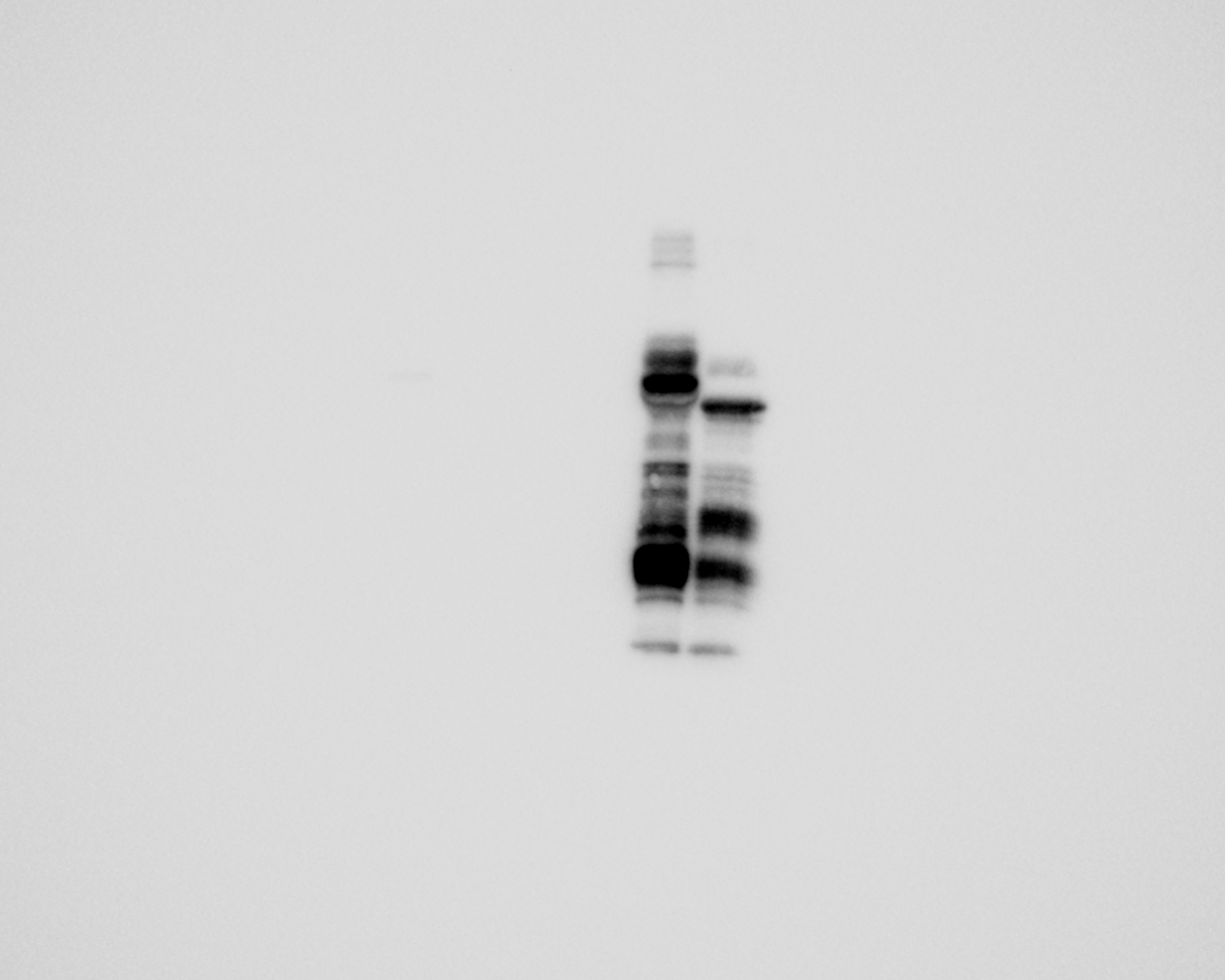

Supplement: Figure 4—source data 1. [file elife-96979-fig4-data1.zip › Figure 4_source data/Raw unedited gels for (Figure 4)/3. Anti-GFP HRP_less exposure for IP/aog 2023-07-18 10h46m35s(Chemiluminescence).tif]

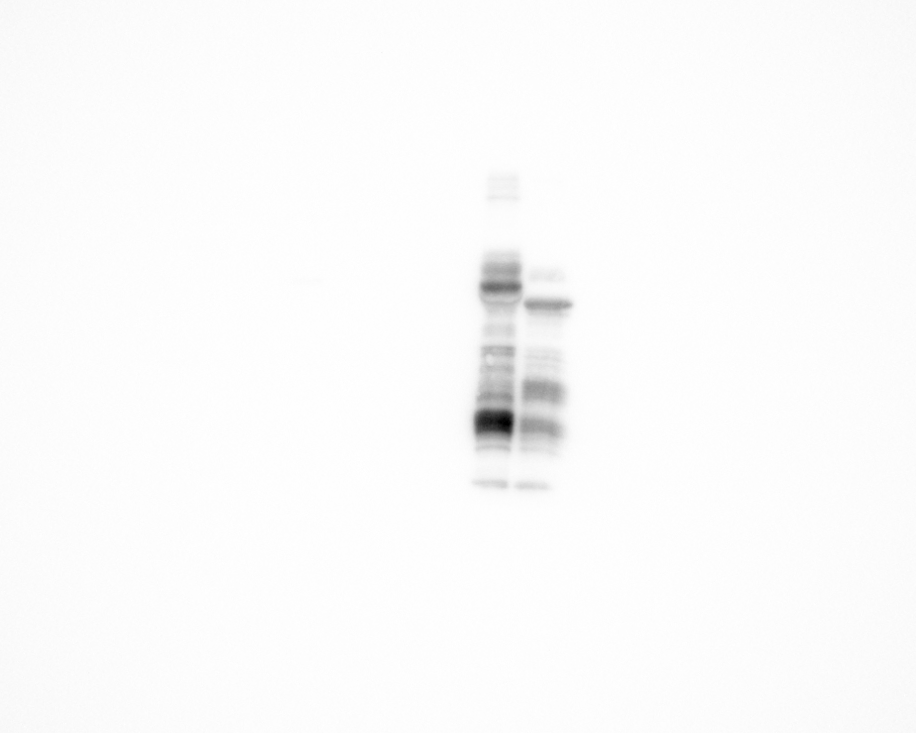

Supplement: Figure 4—source data 1. [file elife-96979-fig4-data1.zip › Figure 4_source data/Raw unedited gels for (Figure 4)/3. Anti-GFP HRP_less exposure for IP/aog 2023-07-18 10h46m35s(Chemiluminescence).raw16.tif]

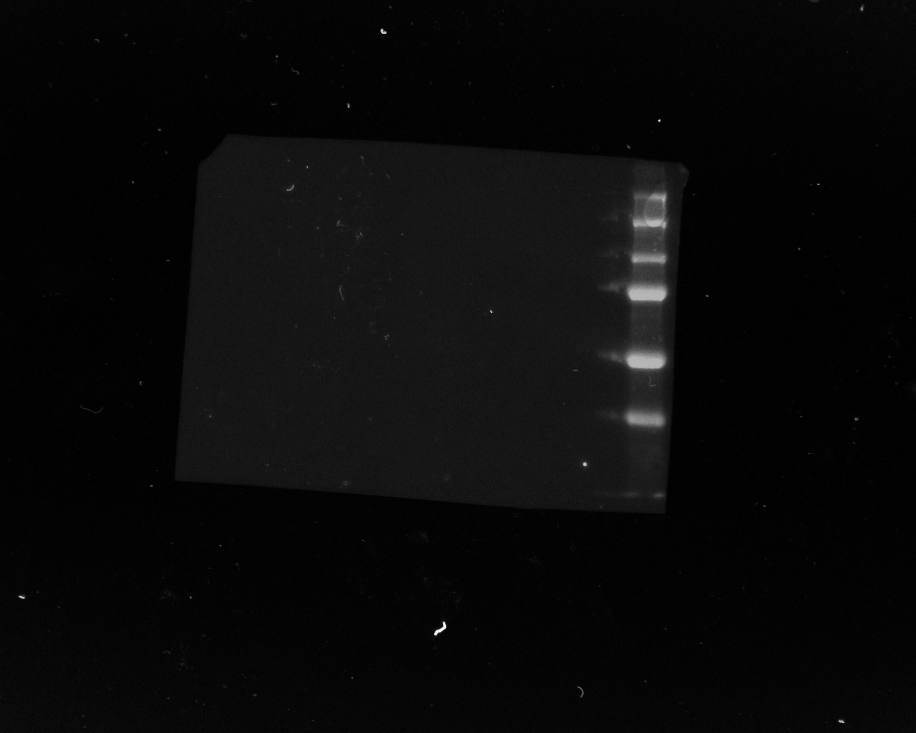

Supplement: Figure 4—source data 1. [file elife-96979-fig4-data1.zip › Figure 4_source data/Raw unedited gels for (Figure 4)/3. Anti-GFP HRP_less exposure for IP/aog 2023-07-18 10h47m11s(Coomassie Blue).raw16.tif]

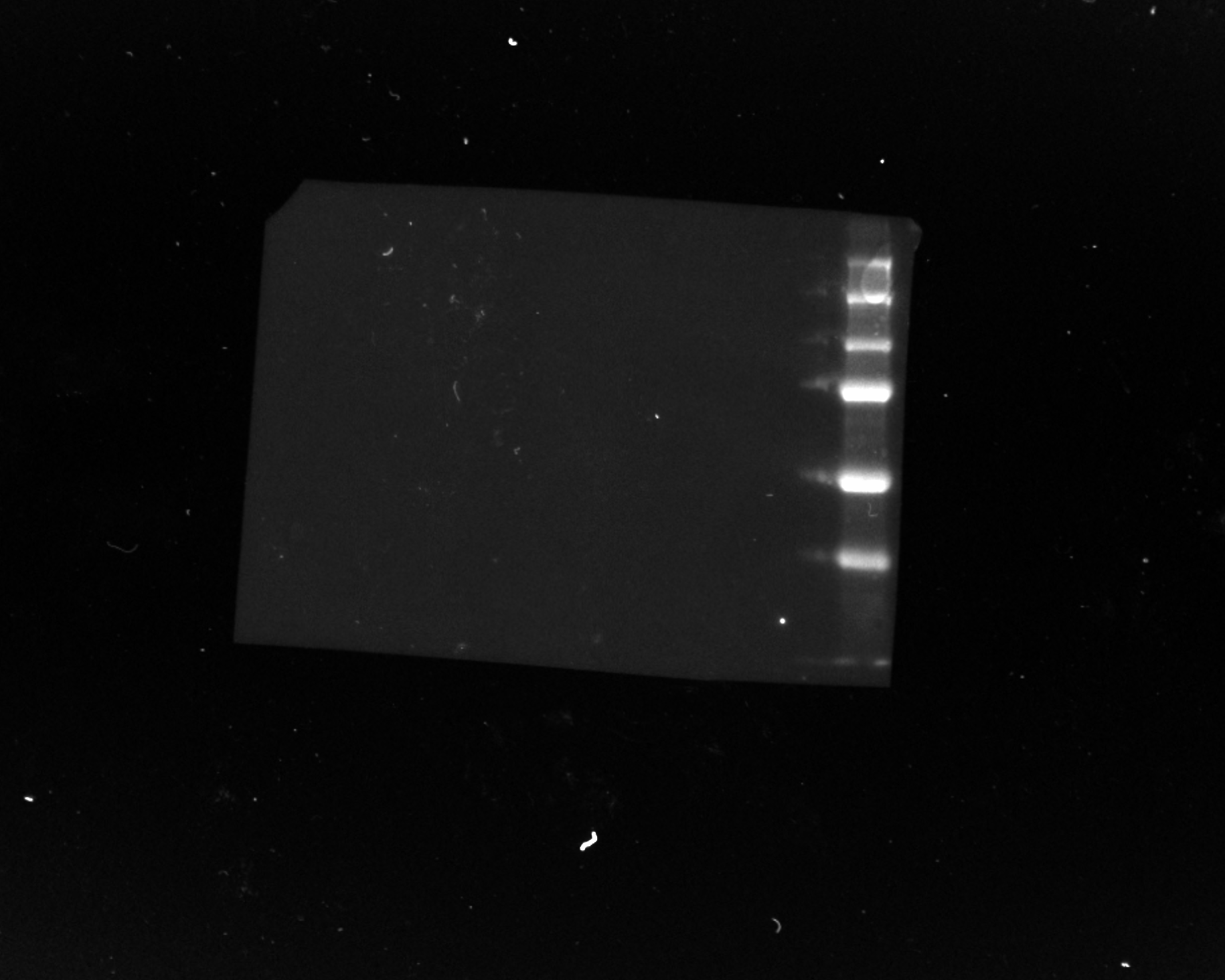

Supplement: Figure 4—source data 1. [file elife-96979-fig4-data1.zip › Figure 4_source data/Raw unedited gels for (Figure 4)/3. Anti-GFP HRP_less exposure for IP/aog 2023-07-18 10h47m11s(Coomassie Blue).tif]

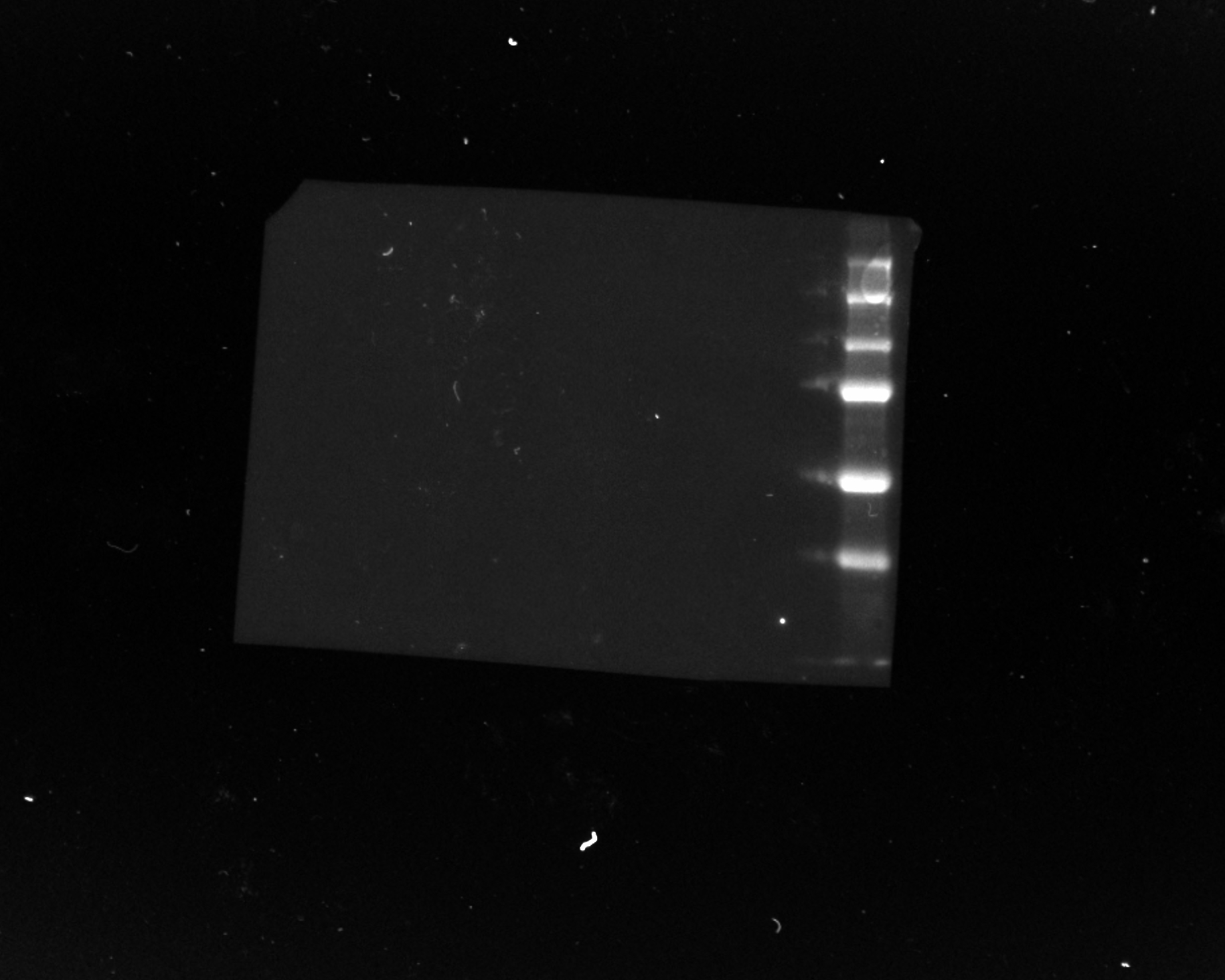

Supplement: Figure 4—source data 1. [file elife-96979-fig4-data1.zip › Figure 4_source data/Raw unedited gels for (Figure 4)/3. Anti-GFP HRP_less exposure for IP/aog 2023-07-18 10h47m11s(Coomassie Blue).jpg]
