# Supplementary figures and images for: A genome-wide CRISPR/Cas9 screen identifies calreticulin as a selective repressor of ATF6α (part 2 of 2)
Source: eLife. 2024 Jul 29;13:RP96979. doi: 10.7554/eLife.96979 (PMC11286266; doi:10.7554/eLife.96979)

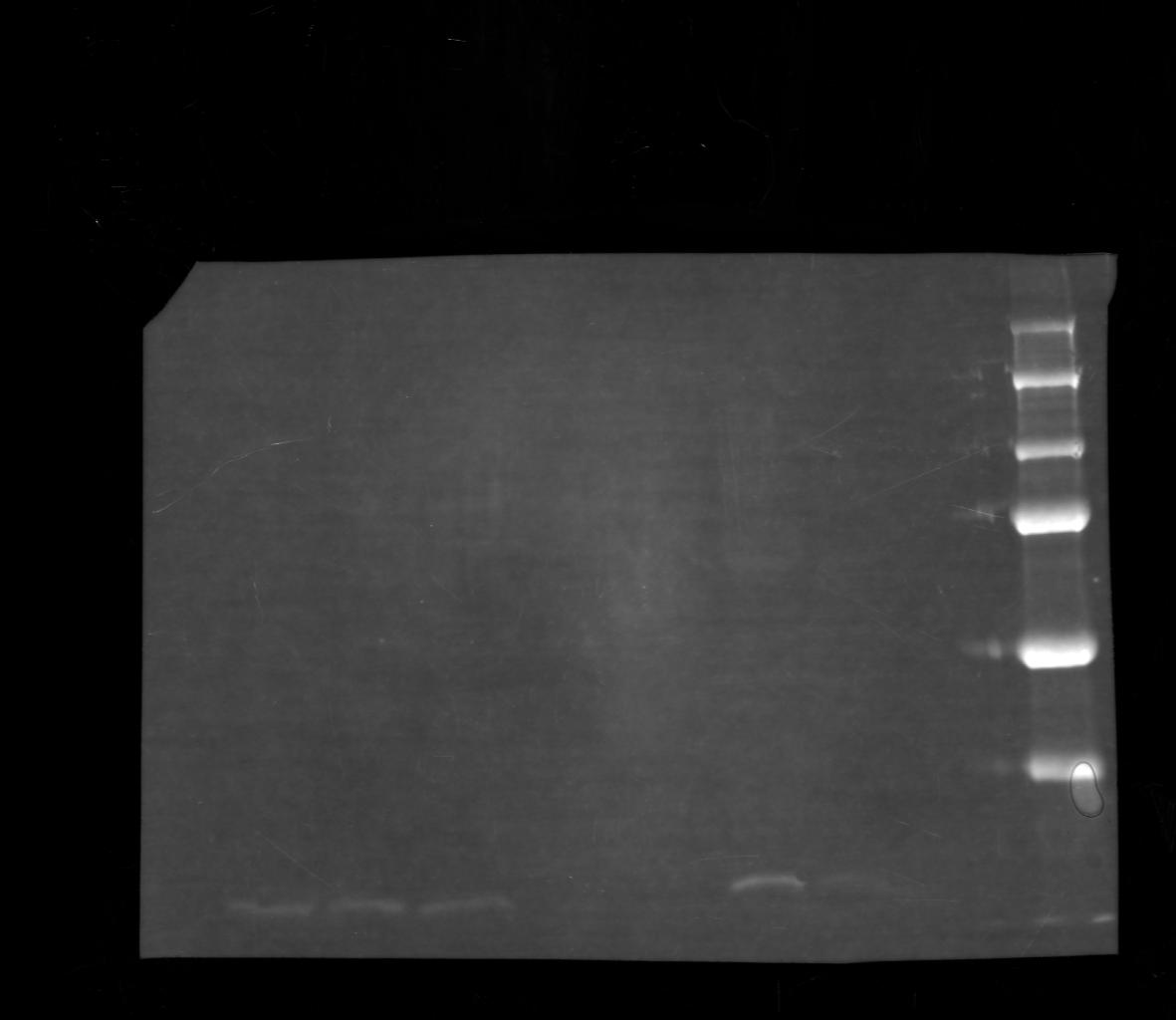

Supplement: Figure 4—source data 1. [file elife-96979-fig4-data1.zip › Figure 4_source data/Raw unedited gels for (Figure 4)/1. anti_CRT_Rb_Licor/700.TIF]

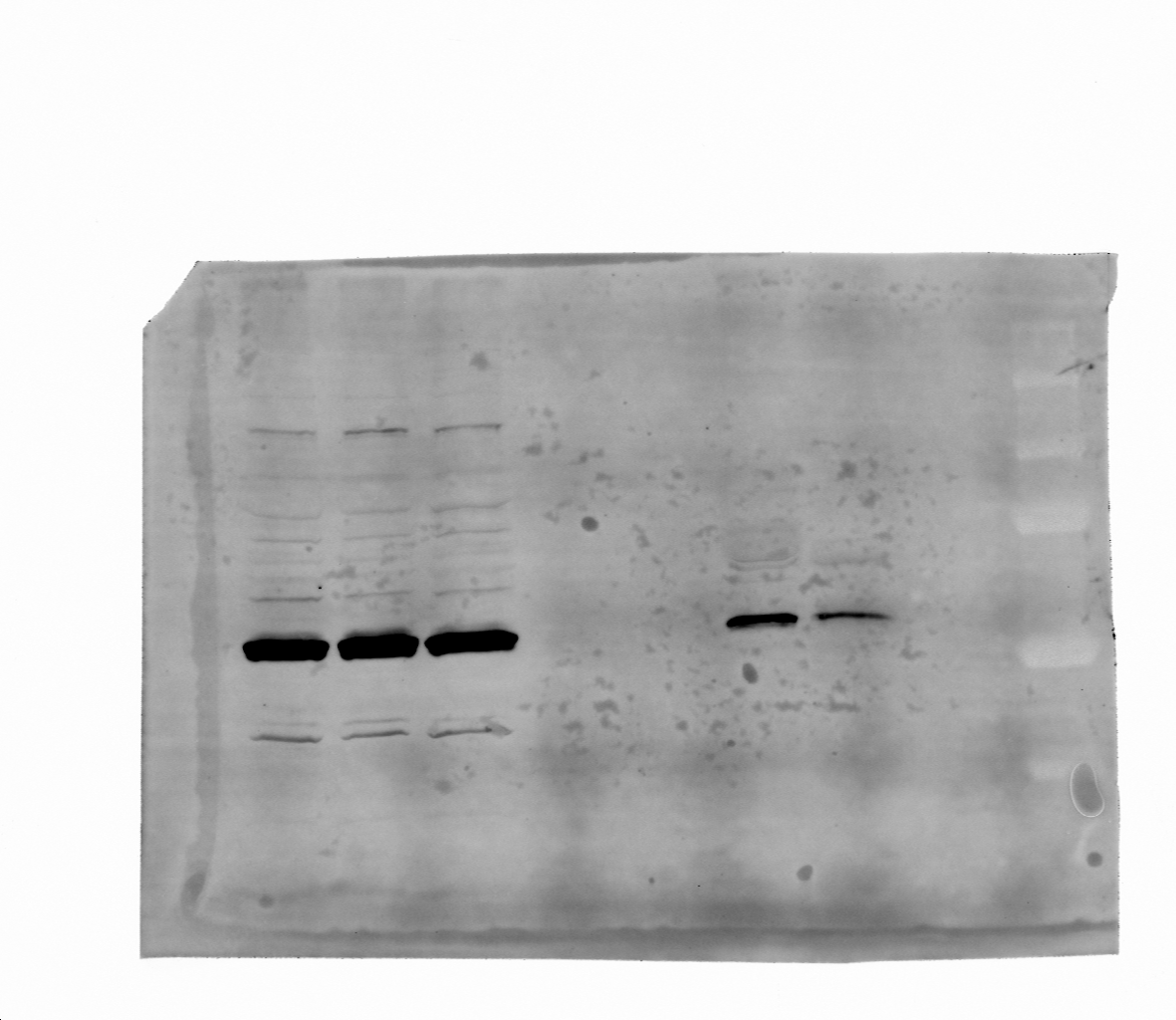

Supplement: Figure 4—source data 1. [file elife-96979-fig4-data1.zip › Figure 4_source data/Raw unedited gels for (Figure 4)/1. anti_CRT_Rb_Licor/800_Modified2.tif]

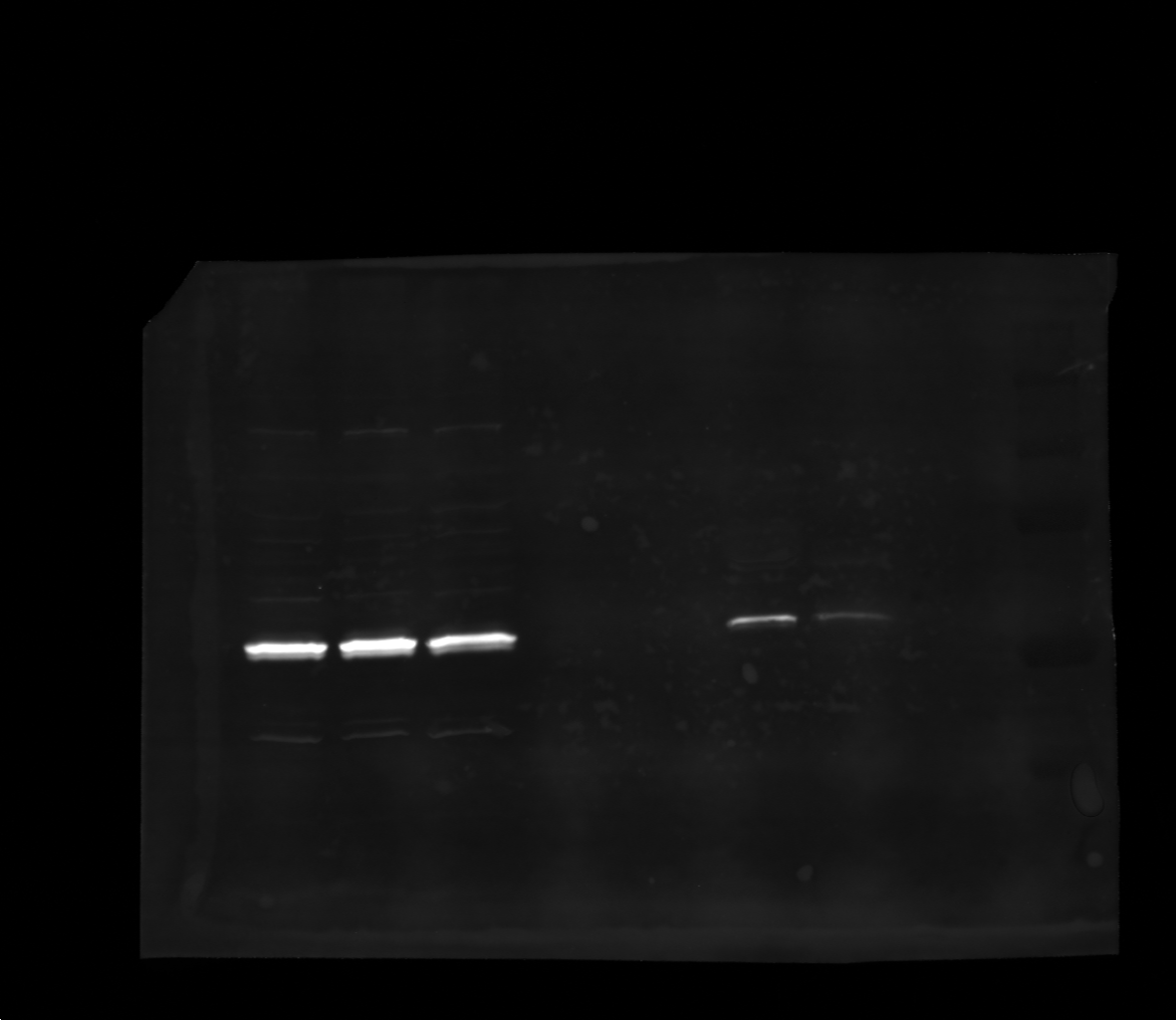

Supplement: Figure 4—source data 1. [file elife-96979-fig4-data1.zip › Figure 4_source data/Raw unedited gels for (Figure 4)/1. anti_CRT_Rb_Licor/800.TIF]

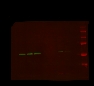

Supplement: Figure 4—source data 1. [file elife-96979-fig4-data1.zip › Figure 4_source data/Raw unedited gels for (Figure 4)/1. anti_CRT_Rb_Licor/2023-07-14-112039_230626_co-ip_2_trial_5_anti-G34_TH.jpg]

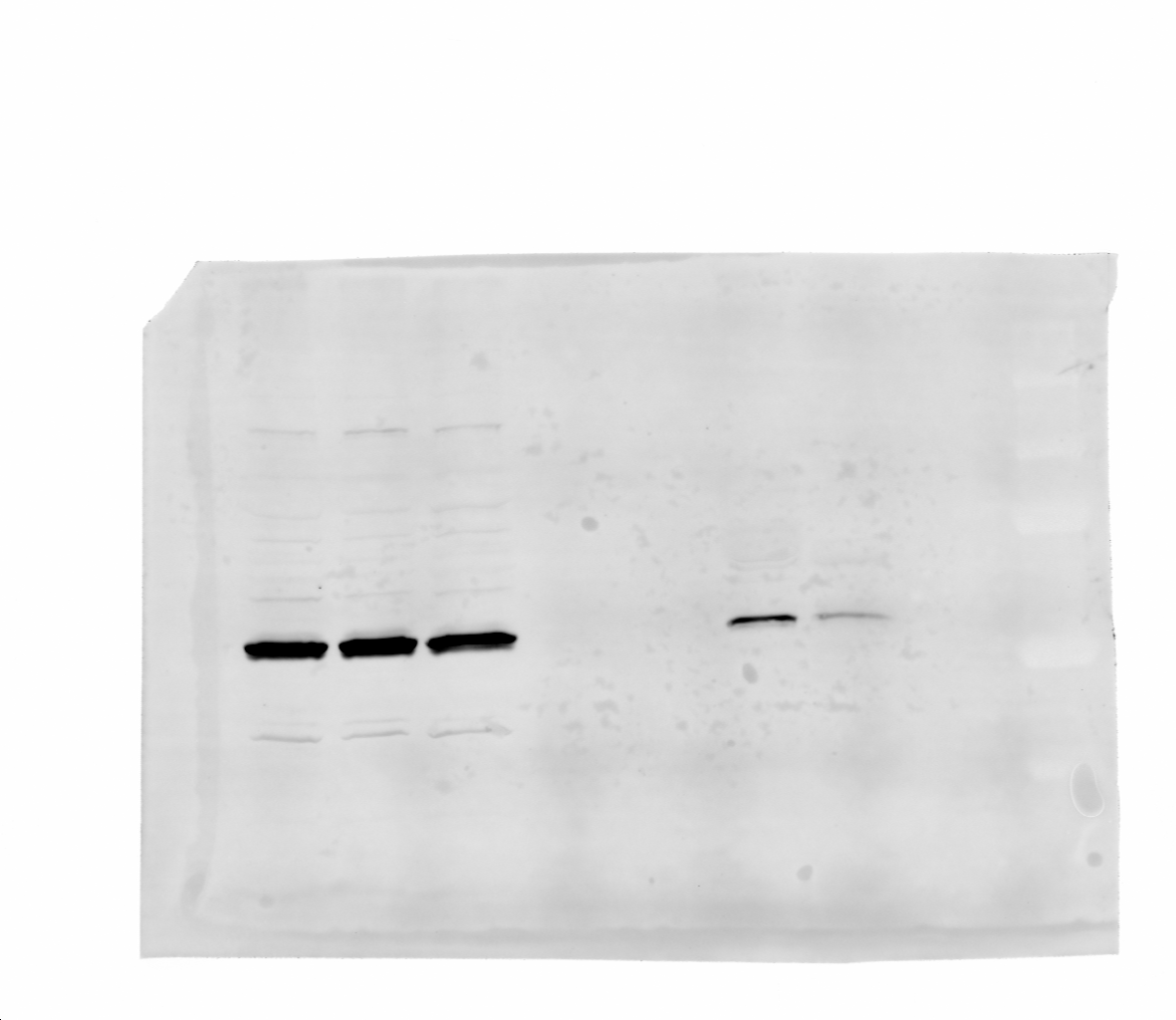

Supplement: Figure 4—source data 1. [file elife-96979-fig4-data1.zip › Figure 4_source data/Raw unedited gels for (Figure 4)/1. anti_CRT_Rb_Licor/800_Modified.tif]

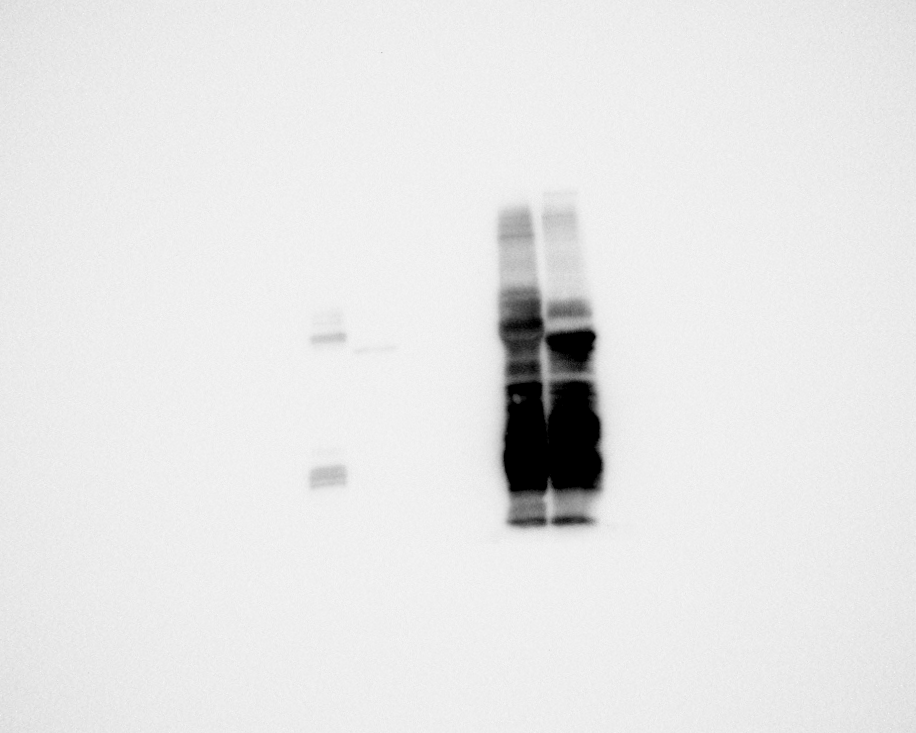

Supplement: Figure 4—source data 1. [file elife-96979-fig4-data1.zip › Figure 4_source data/Raw unedited gels for (Figure 4)/2. Anti-GFP HRP/aog 2023-07-14 13h53m16s(Chemiluminescence).raw16_MODIFIED.tif]

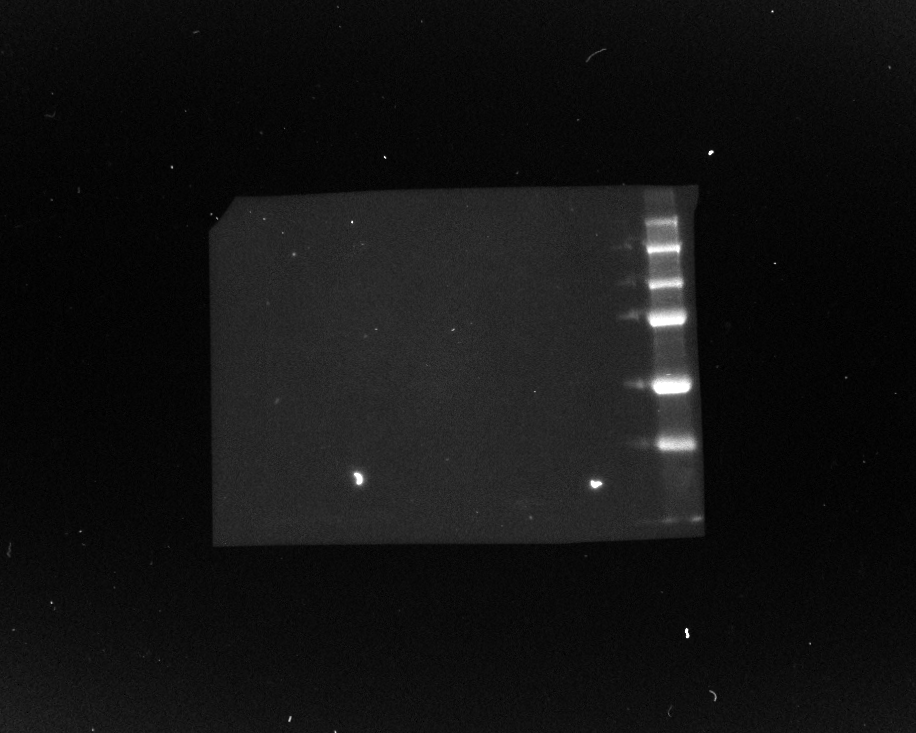

Supplement: Figure 4—source data 1. [file elife-96979-fig4-data1.zip › Figure 4_source data/Raw unedited gels for (Figure 4)/2. Anti-GFP HRP/aog 2023-07-14 13h53m50s(Coomassie Blue).raw16.tif]

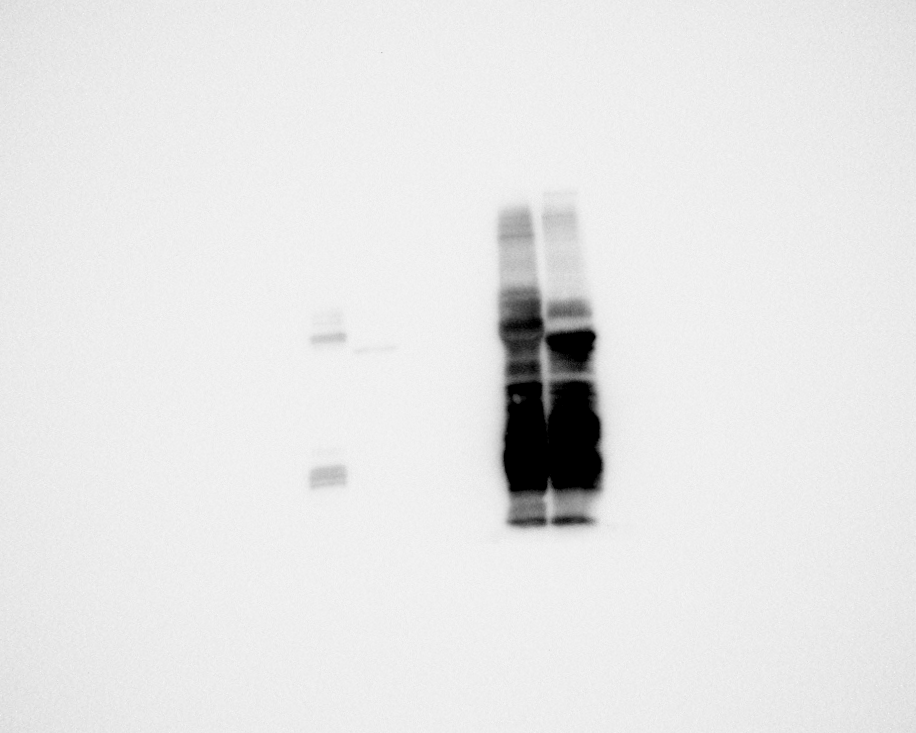

Supplement: Figure 4—source data 1. [file elife-96979-fig4-data1.zip › Figure 4_source data/Raw unedited gels for (Figure 4)/2. Anti-GFP HRP/aog 2023-07-14 13h53m16s(Chemiluminescence).raw16_Modified 2.tif]

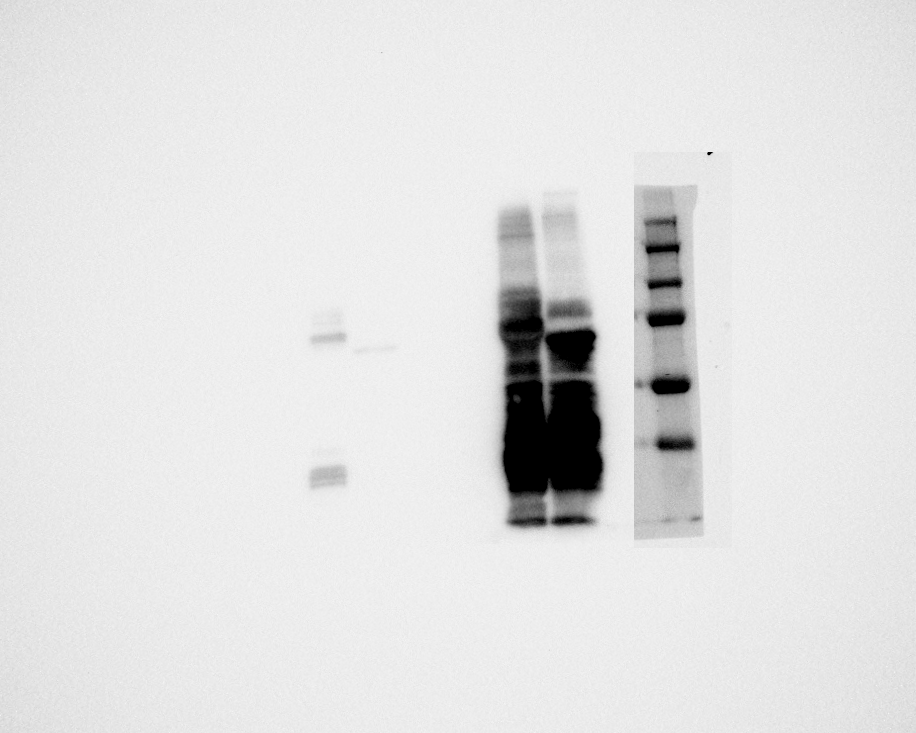

Supplement: Figure 4—source data 1. [file elife-96979-fig4-data1.zip › Figure 4_source data/Raw unedited gels for (Figure 4)/2. Anti-GFP HRP/aog 2023-07-14 13h53m16s(Chemiluminescence).raw16_Modified 3.tif]

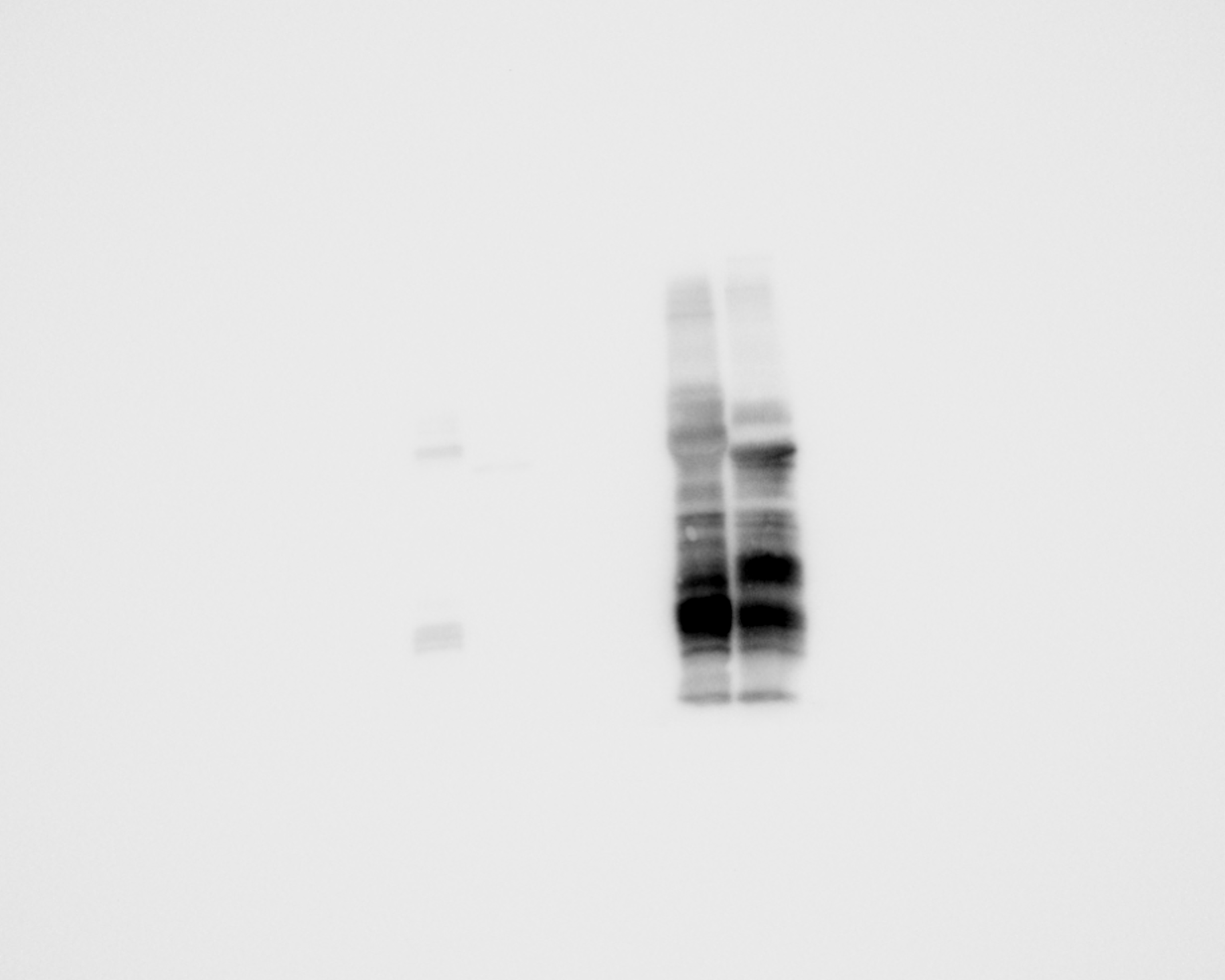

Supplement: Figure 4—source data 1. [file elife-96979-fig4-data1.zip › Figure 4_source data/Raw unedited gels for (Figure 4)/2. Anti-GFP HRP/aog 2023-07-14 13h53m16s(Chemiluminescence).tif]

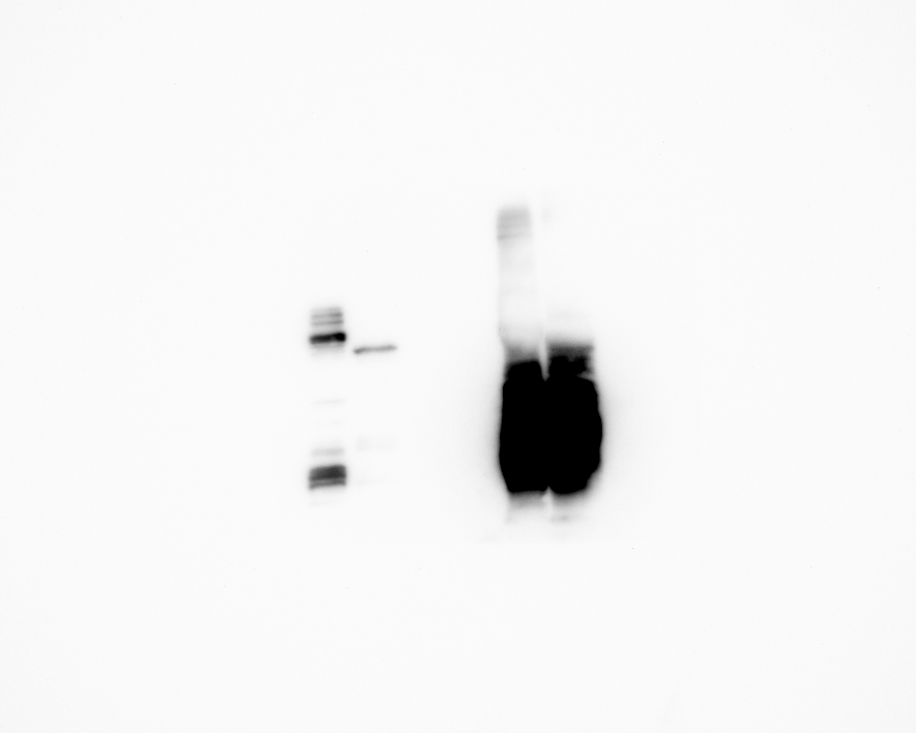

Supplement: Figure 4—source data 1. [file elife-96979-fig4-data1.zip › Figure 4_source data/Raw unedited gels for (Figure 4)/2. Anti-GFP HRP/aog 2023-07-14 13h56m02s(Chemiluminescence)MODIFIED.raw16.tif]

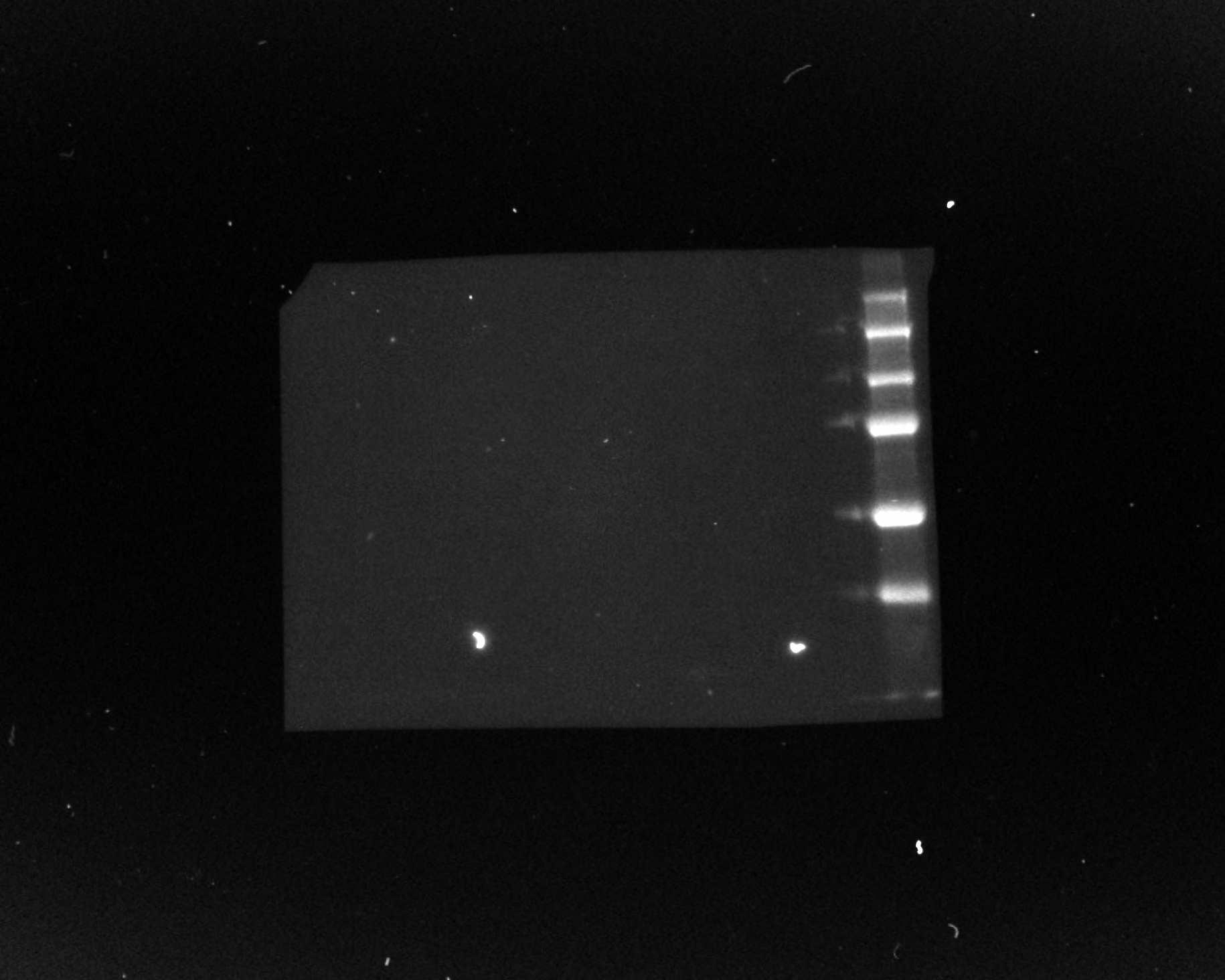

Supplement: Figure 4—source data 1. [file elife-96979-fig4-data1.zip › Figure 4_source data/Raw unedited gels for (Figure 4)/2. Anti-GFP HRP/aog 2023-07-14 13h53m50s(Coomassie Blue).tif]

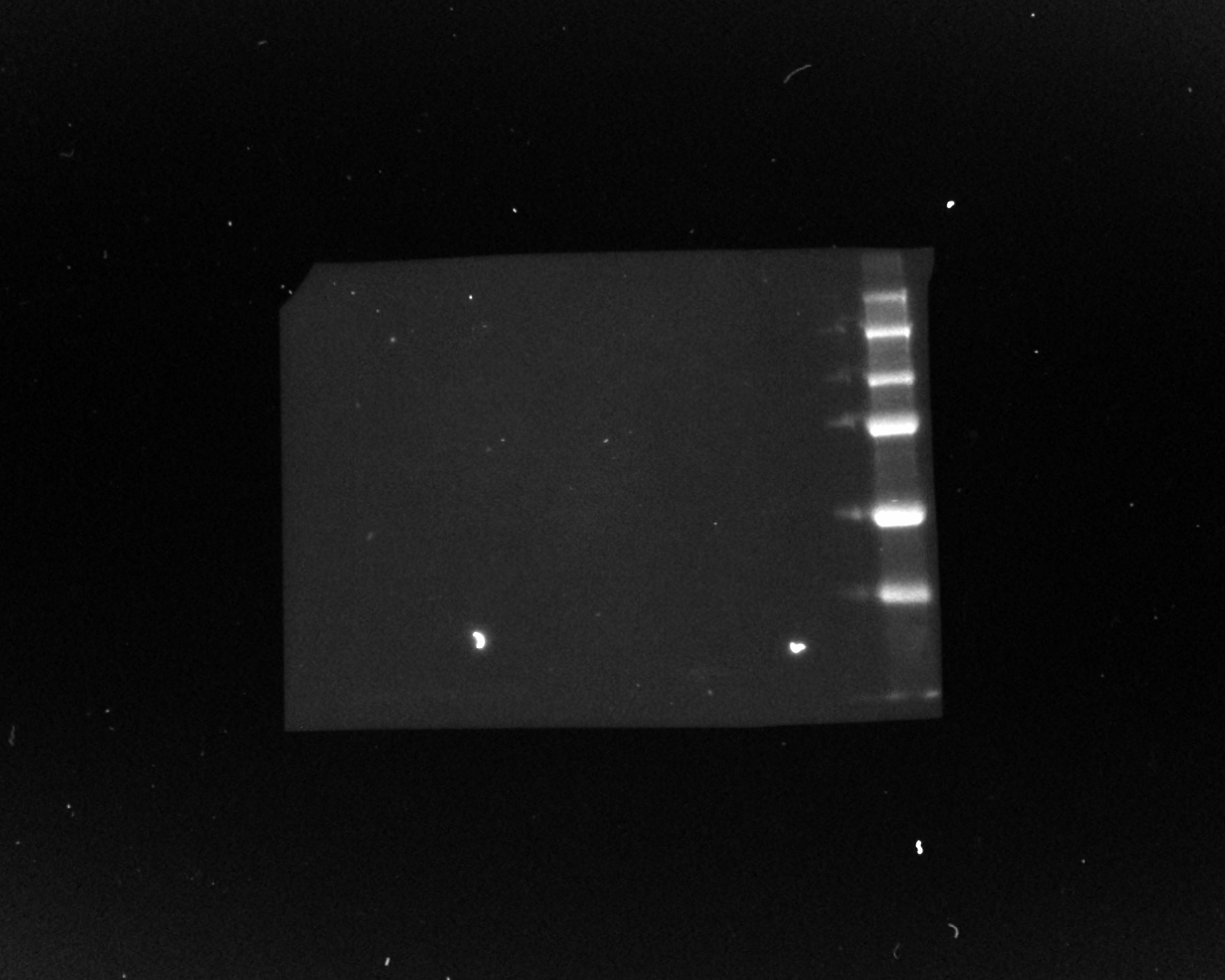

Supplement: Figure 4—source data 1. [file elife-96979-fig4-data1.zip › Figure 4_source data/Raw unedited gels for (Figure 4)/2. Anti-GFP HRP/aog 2023-07-14 13h53m50s(Coomassie Blue).jpg]

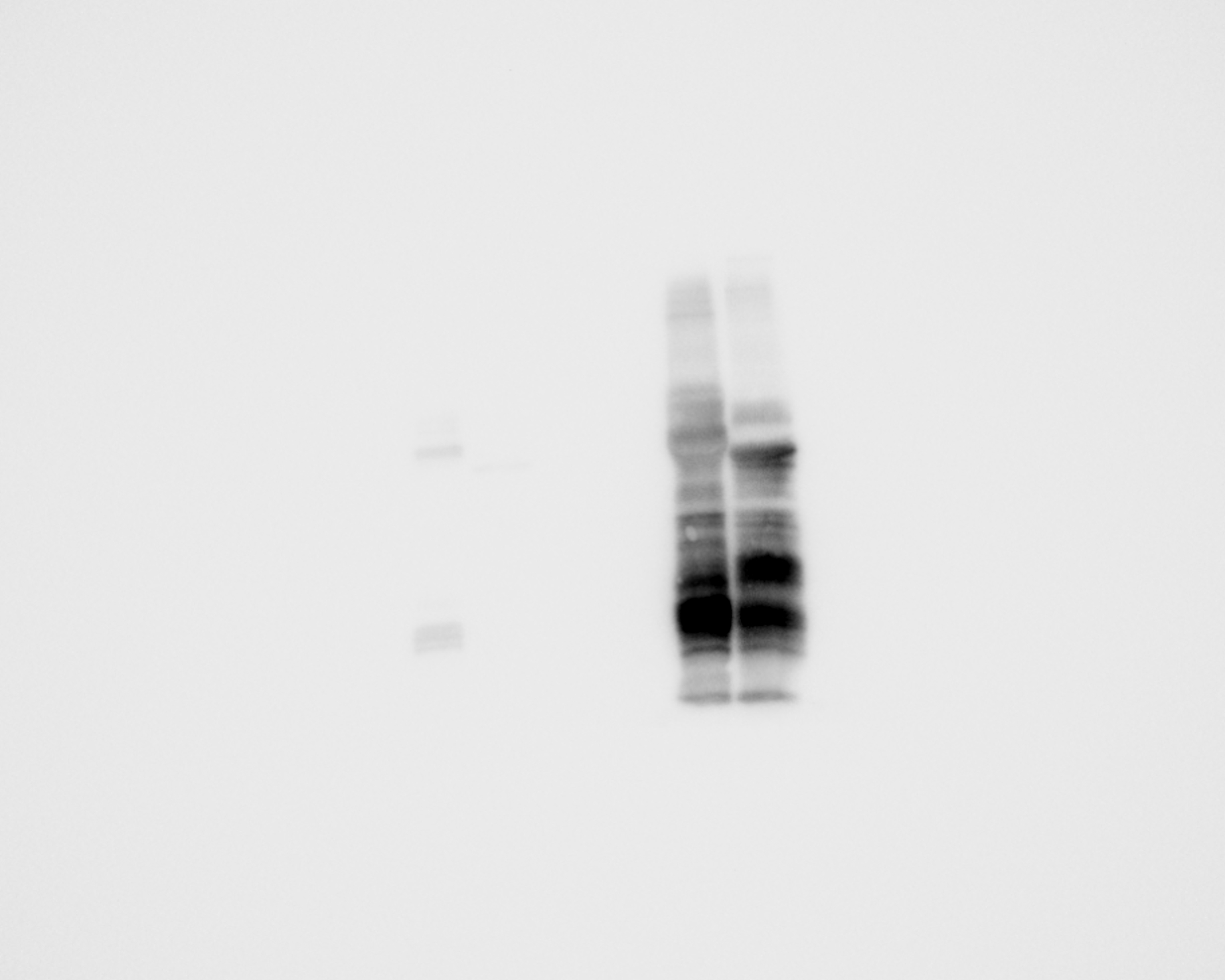

Supplement: Figure 4—source data 1. [file elife-96979-fig4-data1.zip › Figure 4_source data/Raw unedited gels for (Figure 4)/2. Anti-GFP HRP/aog 2023-07-14 13h53m16s(Chemiluminescence).jpg]

Uncropped and labelled gels for (Figure 4\_figure supplement 1)

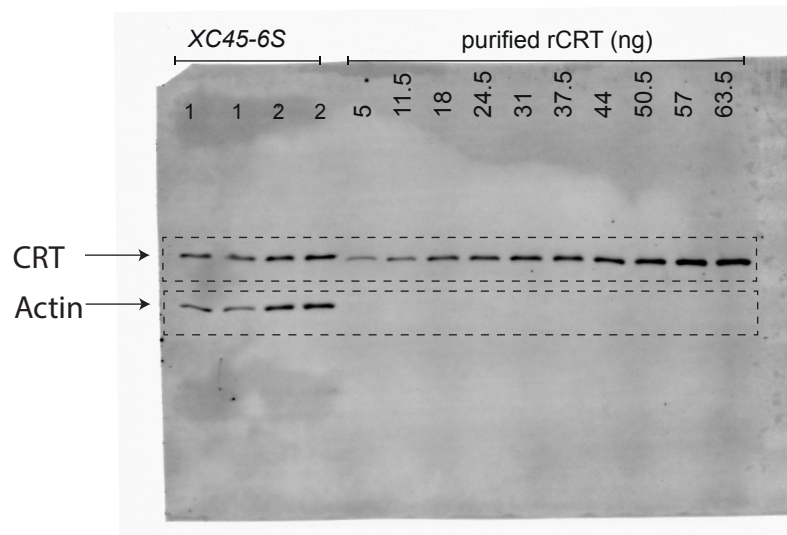

Supplement: Figure 4—figure supplement 1—source data 1. [file elife-96979-fig4-figsupp1-data1.zip › Figure 4_figure supplement 1_source data/Uncropped and labelled gels for (Figure 4-figure supplement 1).pdf]

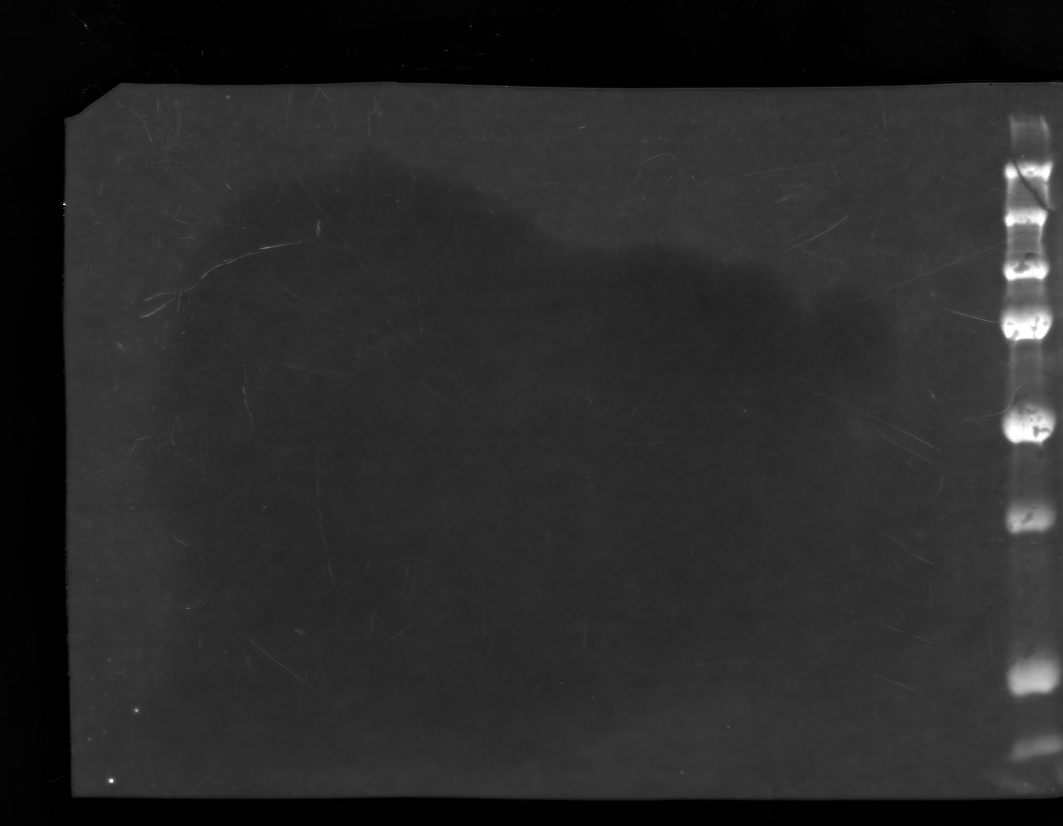

Supplement: Figure 4—figure supplement 1—source data 1. [file elife-96979-fig4-figsupp1-data1.zip › Figure 4_figure supplement 1_source data/Raw unedited gels for (Figure 4-figure supplement 1)/CRT and Actin/700.TIF]

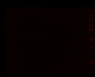

Supplement: Figure 4—figure supplement 1—source data 1. [file elife-96979-fig4-figsupp1-data1.zip › Figure 4_figure supplement 1_source data/Raw unedited gels for (Figure 4-figure supplement 1)/CRT and Actin/2023-03-03-145322_1_TH.jpg]

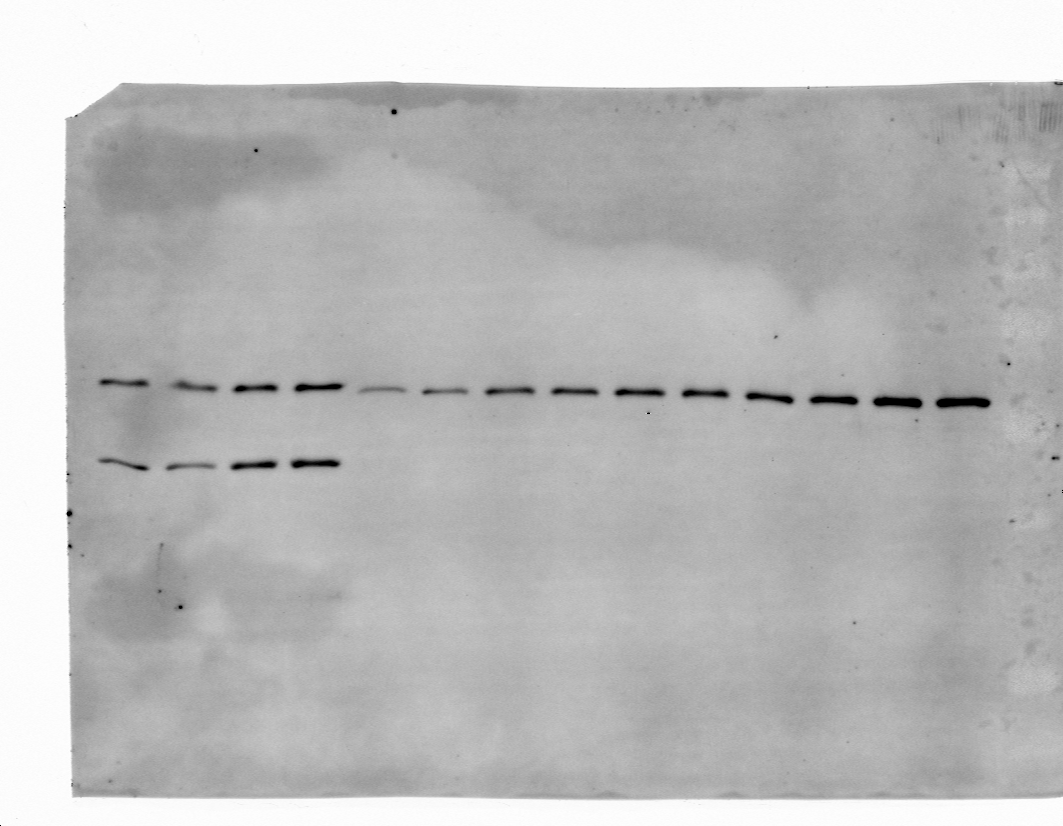

Supplement: Figure 4—figure supplement 1—source data 1. [file elife-96979-fig4-figsupp1-data1.zip › Figure 4_figure supplement 1_source data/Raw unedited gels for (Figure 4-figure supplement 1)/CRT and Actin/800 modified.tif]

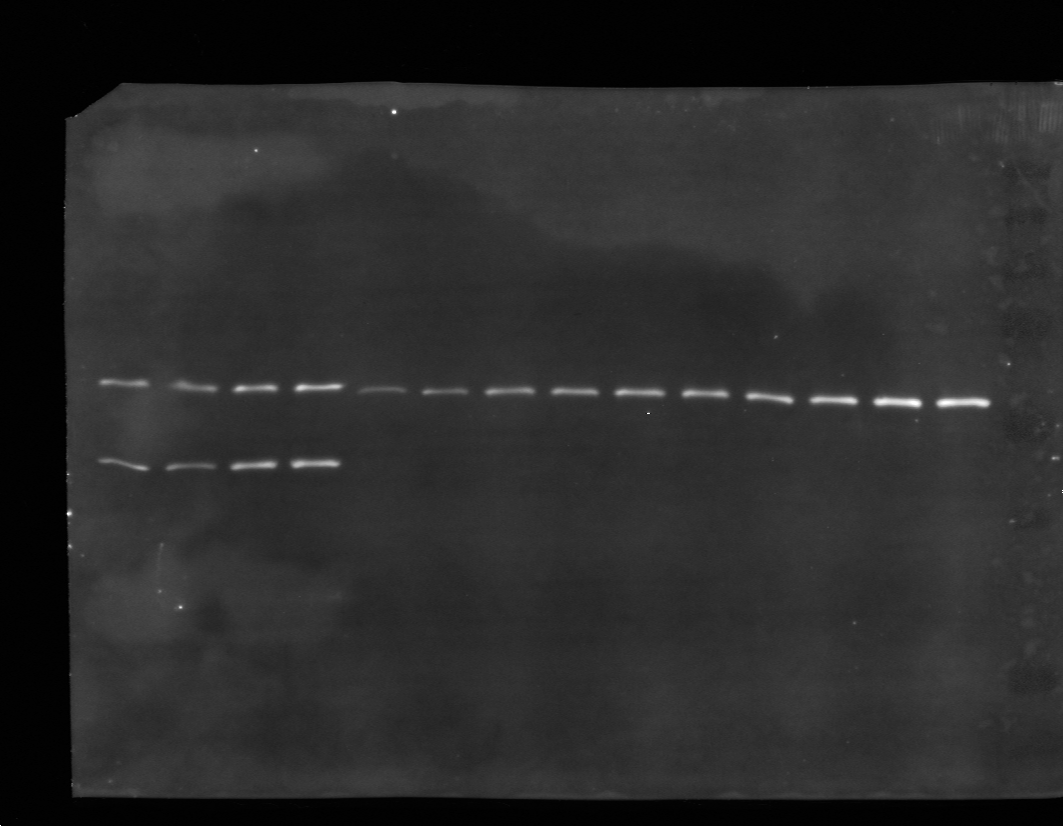

Supplement: Figure 4—figure supplement 1—source data 1. [file elife-96979-fig4-figsupp1-data1.zip › Figure 4_figure supplement 1_source data/Raw unedited gels for (Figure 4-figure supplement 1)/CRT and Actin/800.TIF]

Uncropped and labelled gels for (Figure 6)

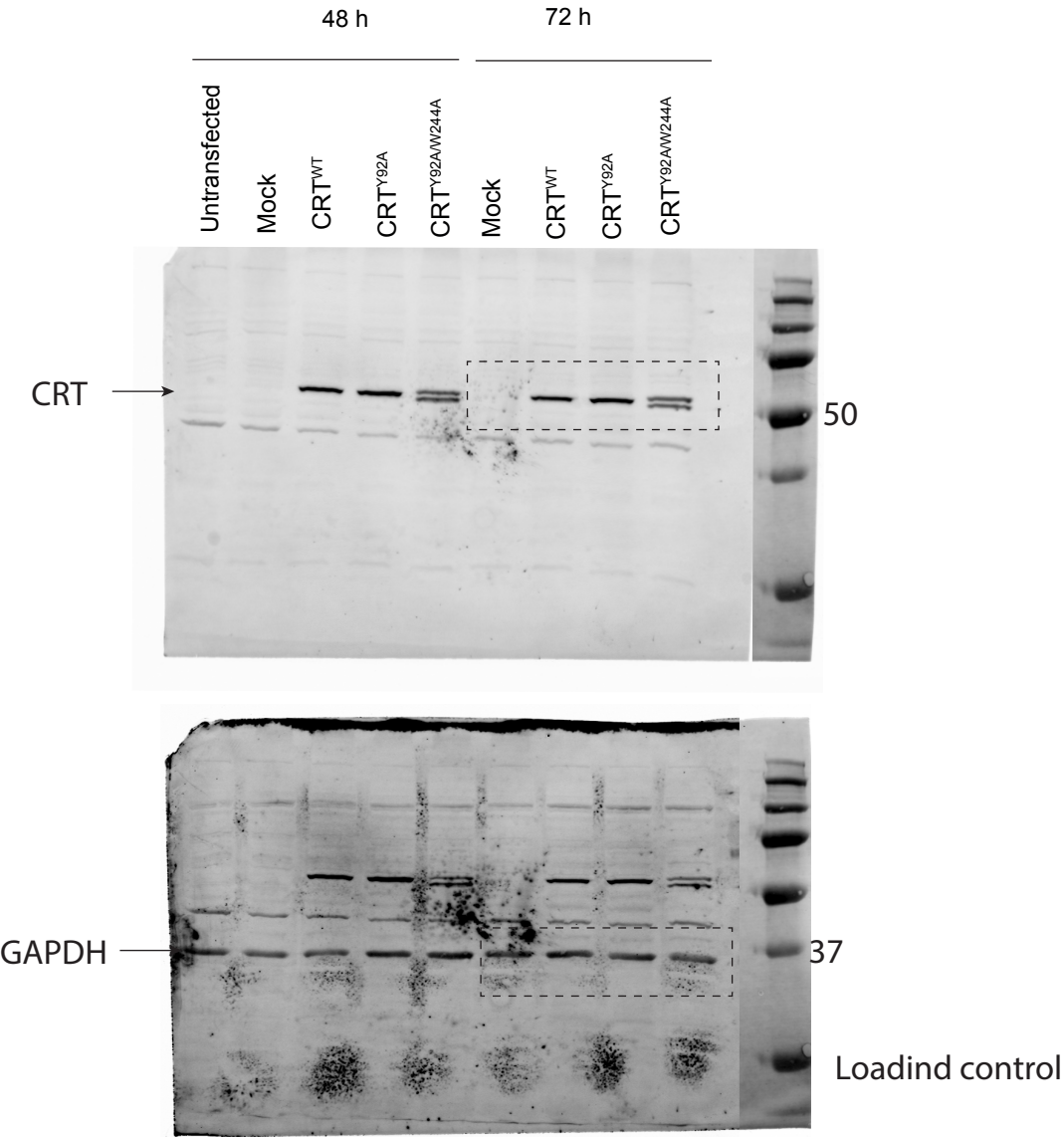

Supplement: Figure 6—source data 1. [file elife-96979-fig6-data1.zip › Figure 6_source data/Uncropped and labelled gels for (Figure 6).pdf]

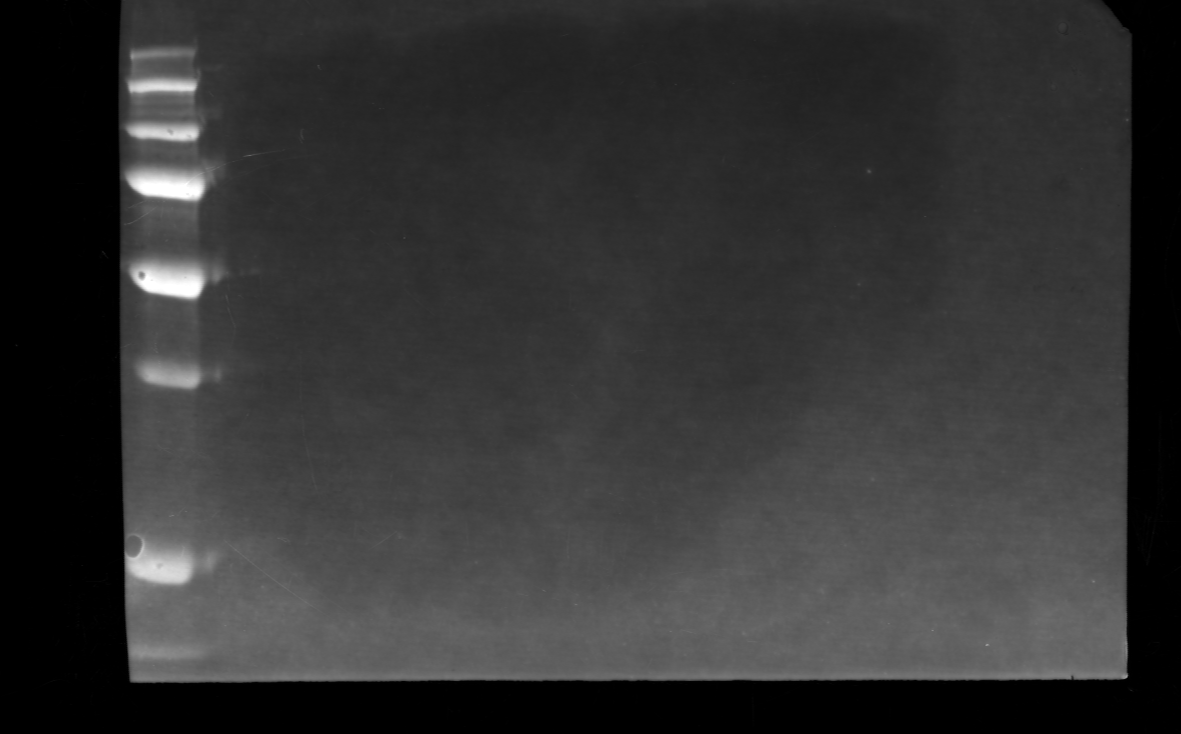

Supplement: Figure 6—source data 1. [file elife-96979-fig6-data1.zip › Figure 6_source data/Raw unedited gels for (Figure 6)/anti-CRT rb human/700.TIF]

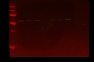

Supplement: Figure 6—source data 1. [file elife-96979-fig6-data1.zip › Figure 6_source data/Raw unedited gels for (Figure 6)/anti-CRT rb human/2023-11-28-143406_1_TH.jpg]

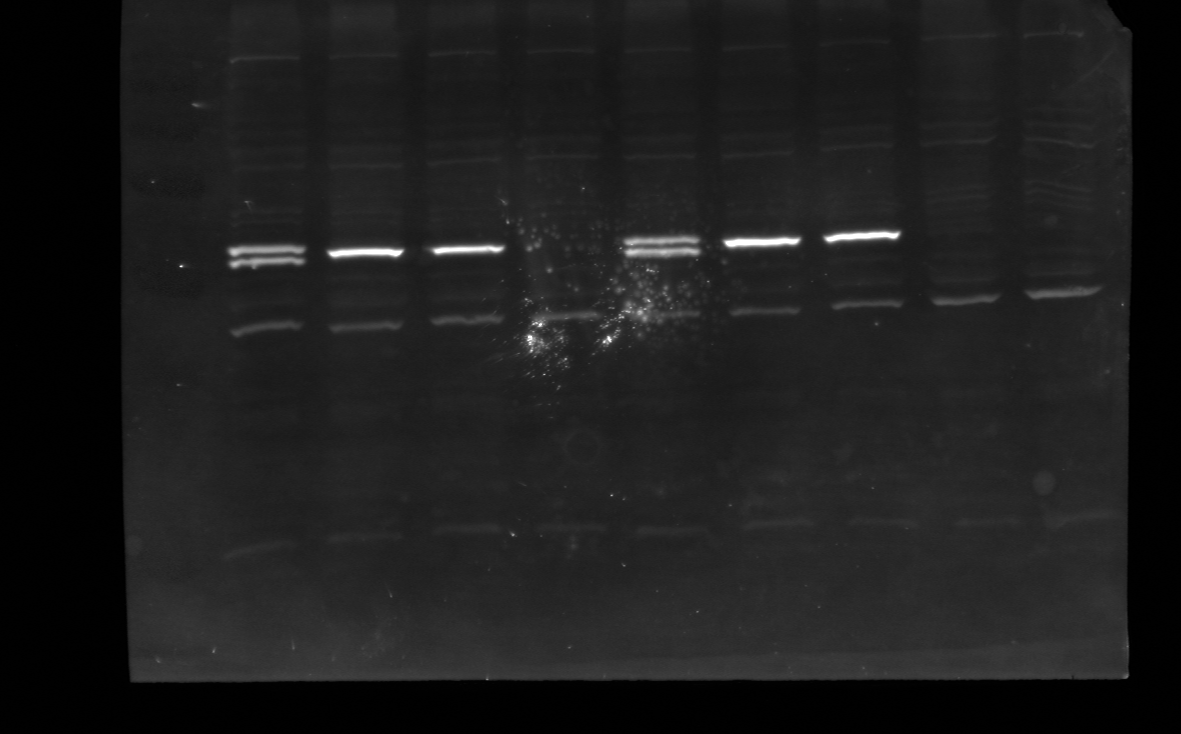

Supplement: Figure 6—source data 1. [file elife-96979-fig6-data1.zip › Figure 6_source data/Raw unedited gels for (Figure 6)/anti-CRT rb human/800.TIF]

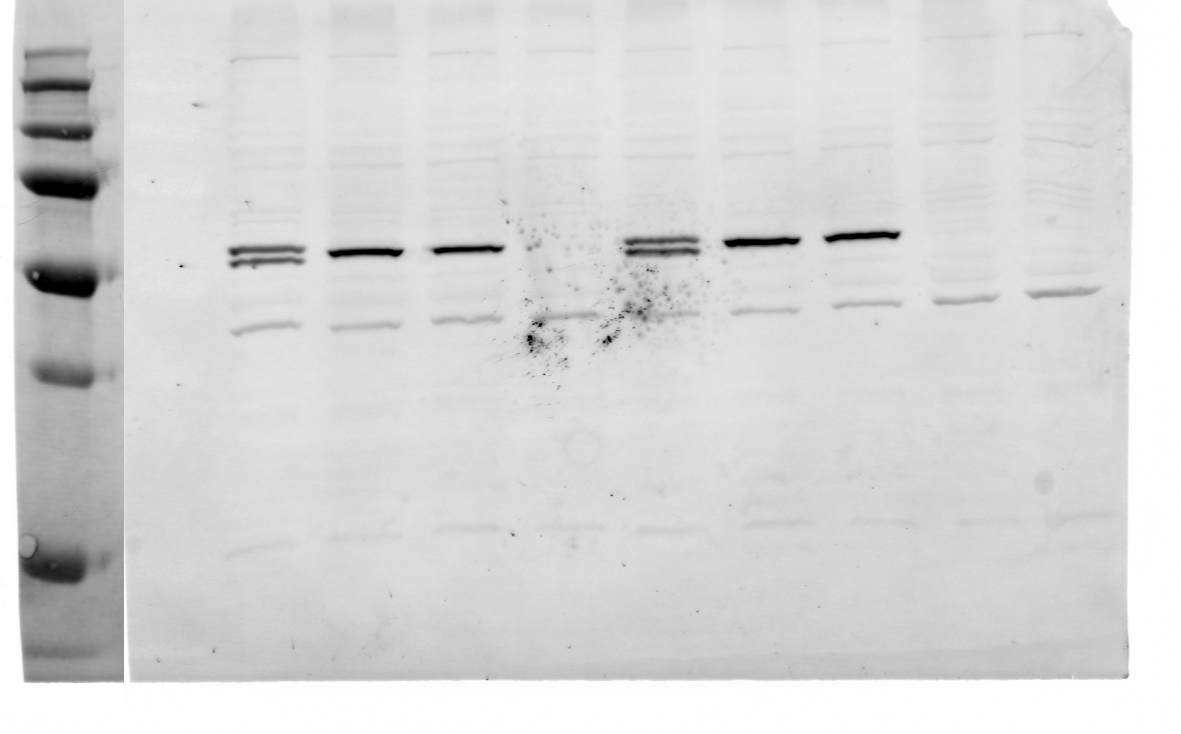

Supplement: Figure 6—source data 1. [file elife-96979-fig6-data1.zip › Figure 6_source data/Raw unedited gels for (Figure 6)/anti-CRT rb human/800_modified.tif]

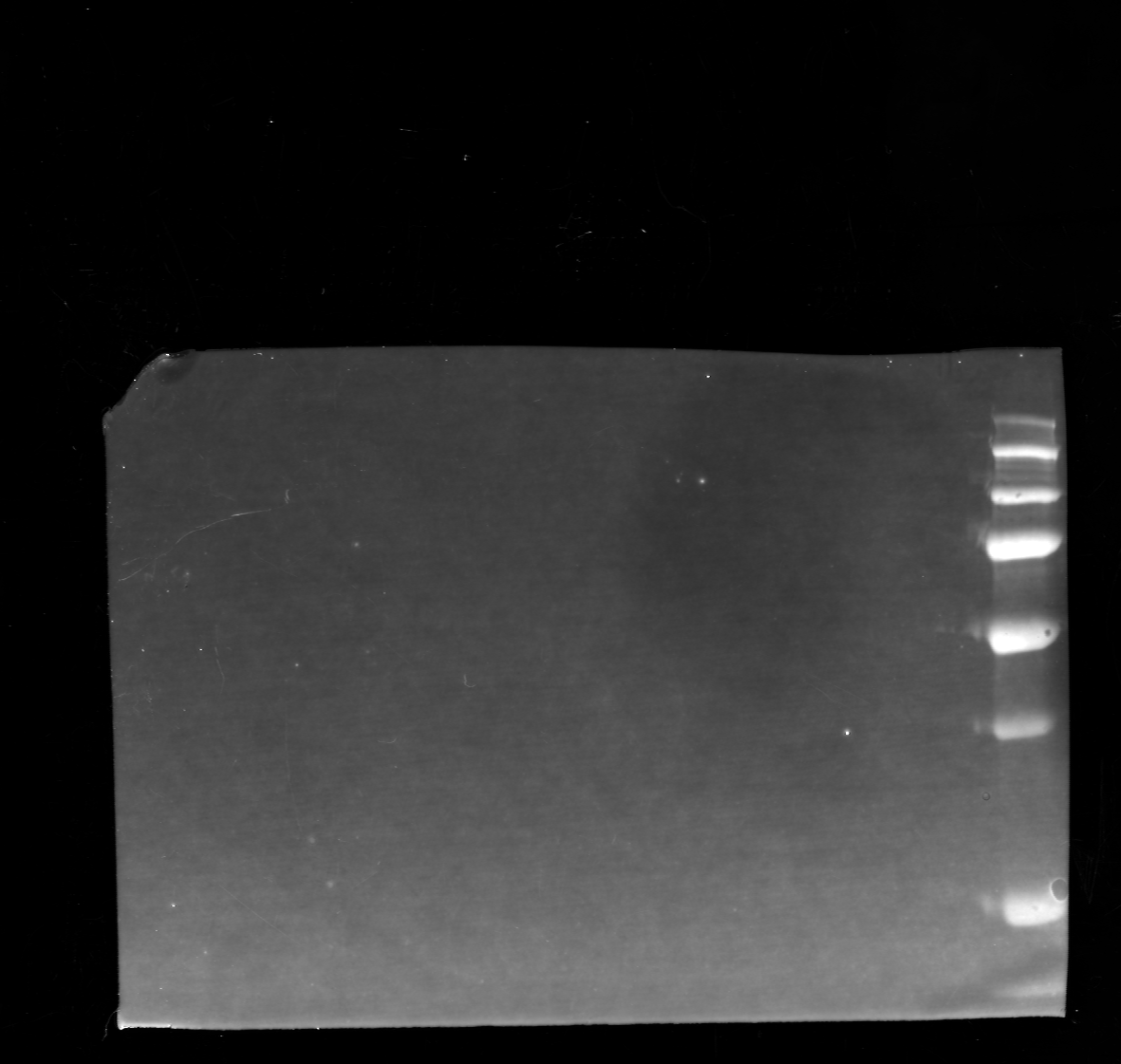

Supplement: Figure 6—source data 1. [file elife-96979-fig6-data1.zip › Figure 6_source data/Raw unedited gels for (Figure 6)/anti-GAPDH rb/700.TIF]

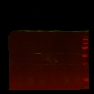

Supplement: Figure 6—source data 1. [file elife-96979-fig6-data1.zip › Figure 6_source data/Raw unedited gels for (Figure 6)/anti-GAPDH rb/2023-12-07-153835_1_TH.jpg]

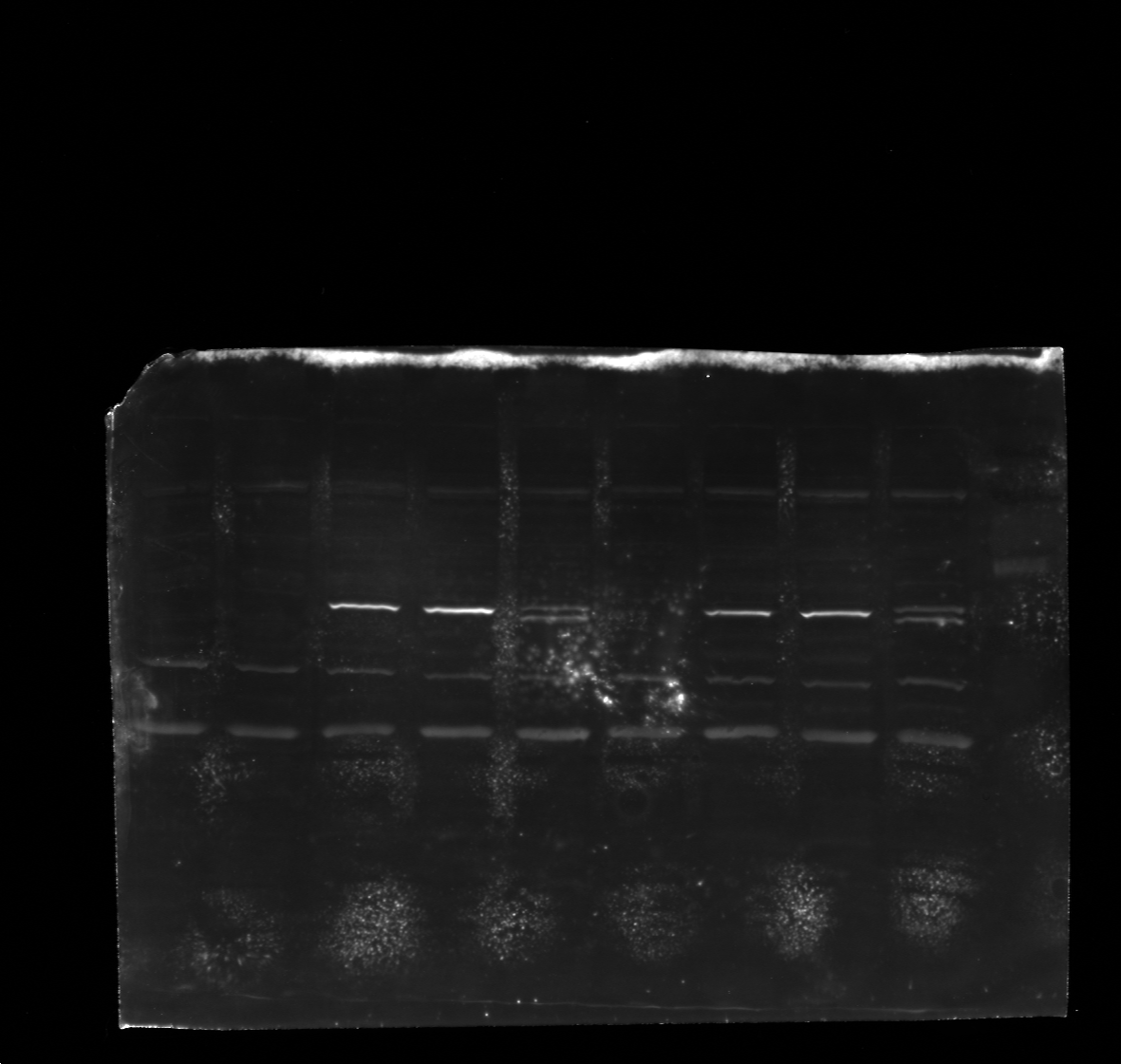

Supplement: Figure 6—source data 1. [file elife-96979-fig6-data1.zip › Figure 6_source data/Raw unedited gels for (Figure 6)/anti-GAPDH rb/800.TIF]

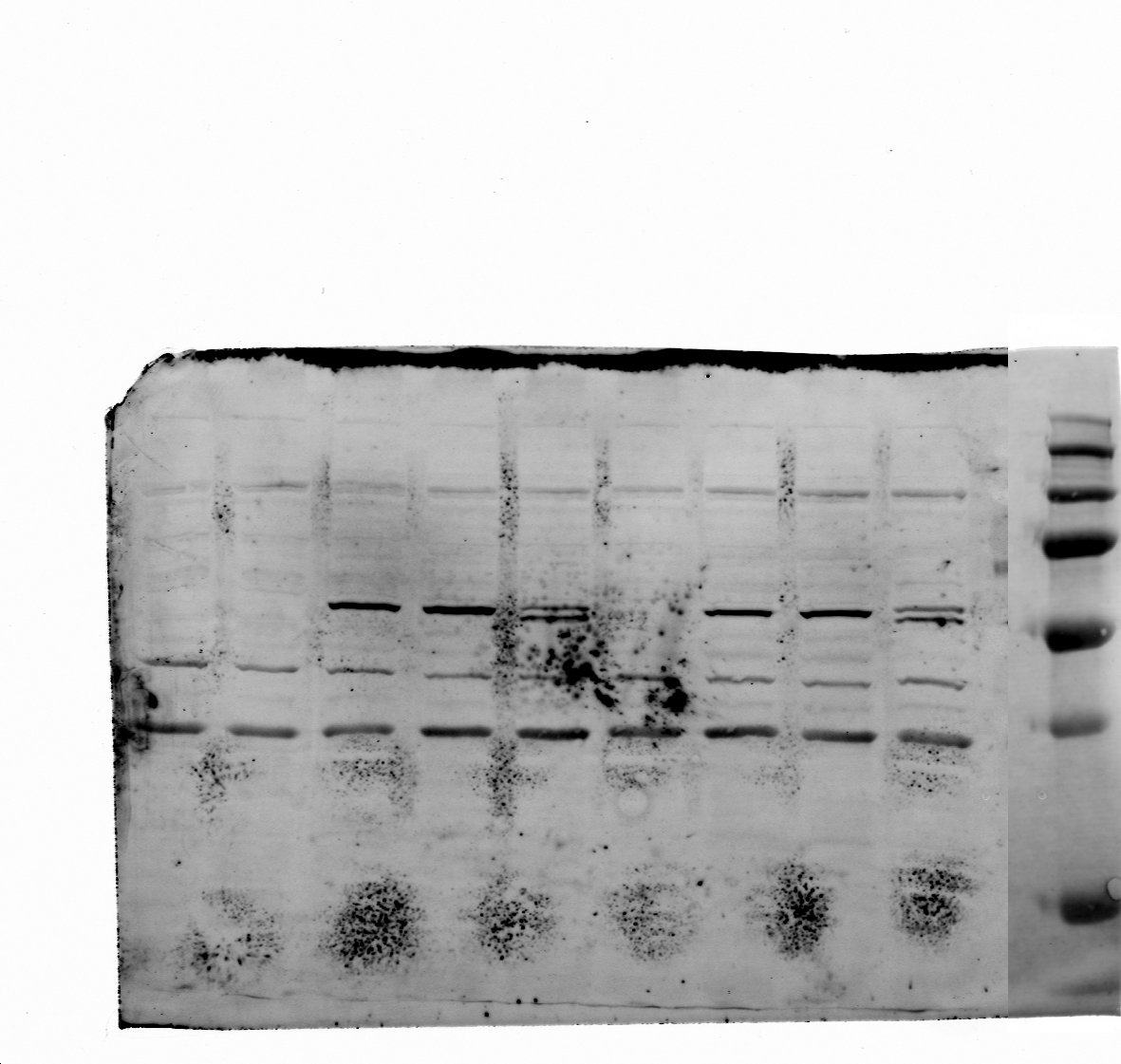

Supplement: Figure 6—source data 1. [file elife-96979-fig6-data1.zip › Figure 6_source data/Raw unedited gels for (Figure 6)/anti-GAPDH rb/800_modified.tif]

25. 07. 2023 Uncropped and labelled gel for Figure 7-B

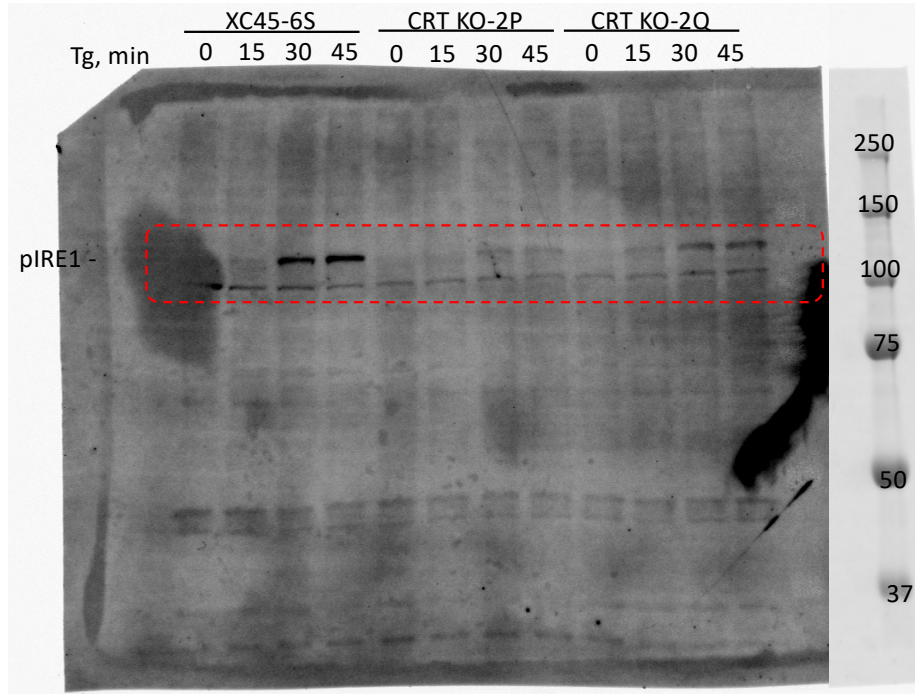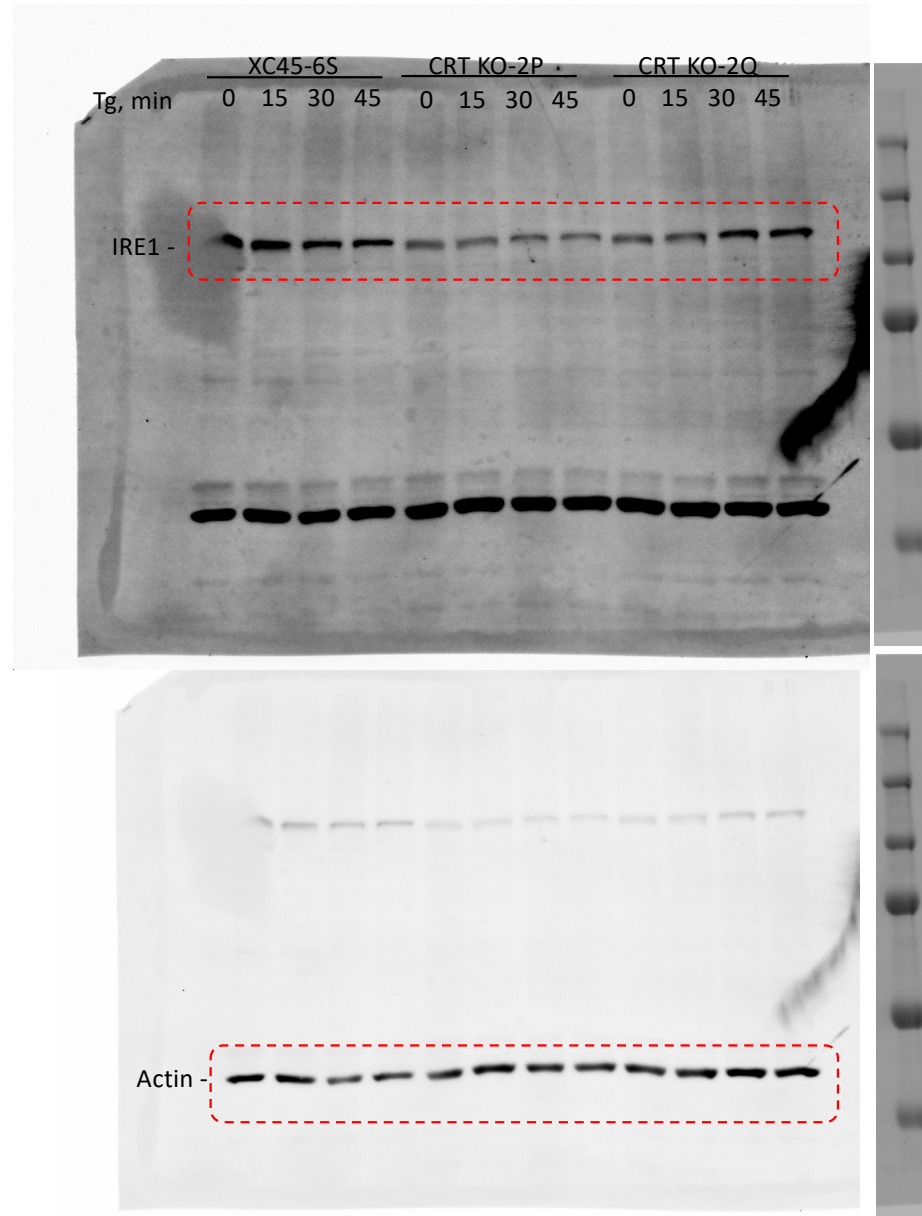

Supplement: Figure 7—source data 1. [file elife-96979-fig7-data1.zip › Figure 7_source data/Uncropped and labelled gel for Figure 7B.pdf]

25. 04. 2023 Uncropped and labelled gel for Figure 7-A

Chemidoc\_  
Sybr Safe

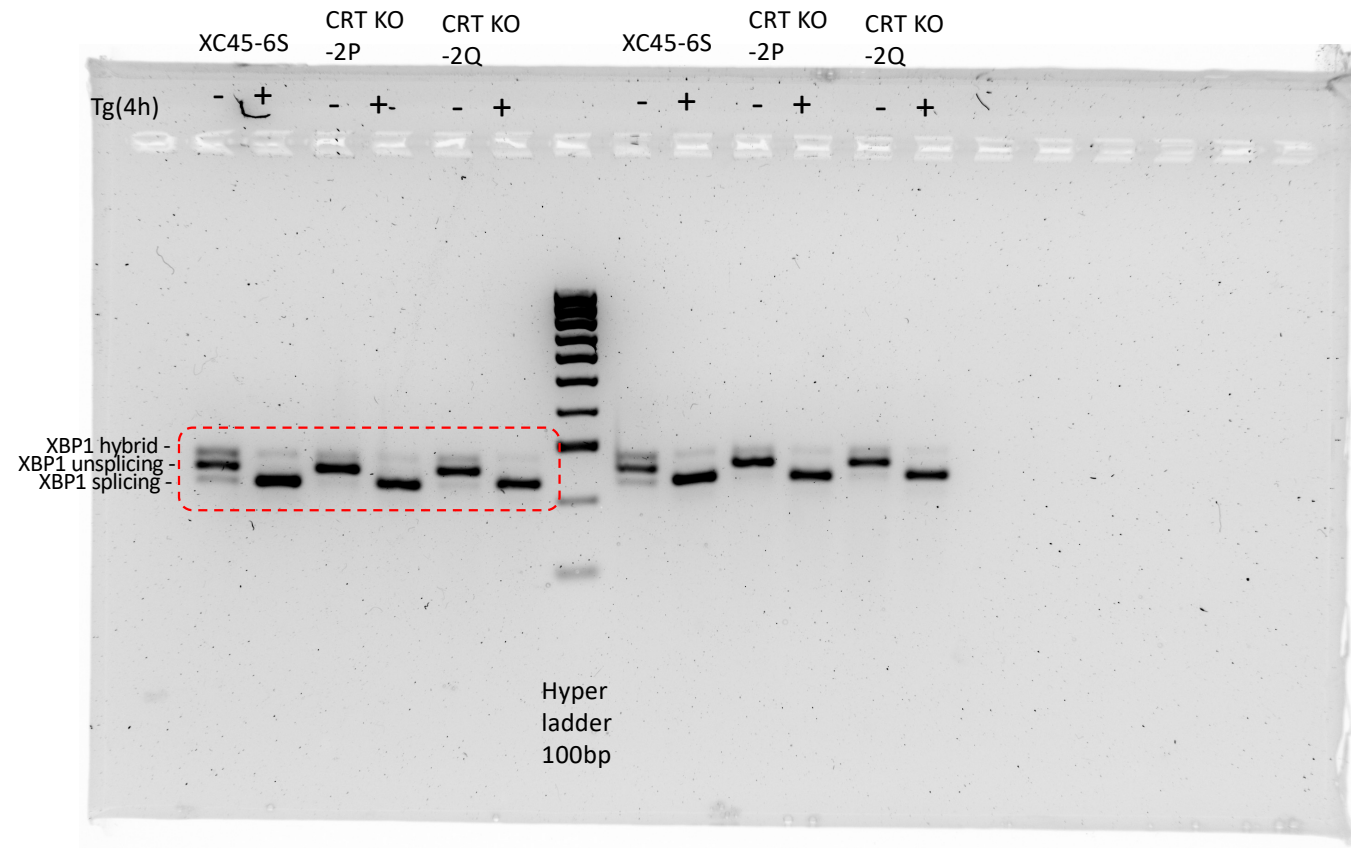

Supplement: Figure 7—source data 1. [file elife-96979-fig7-data1.zip › Figure 7_source data/Uncropped and labbelled gel for figure 7A.pdf]

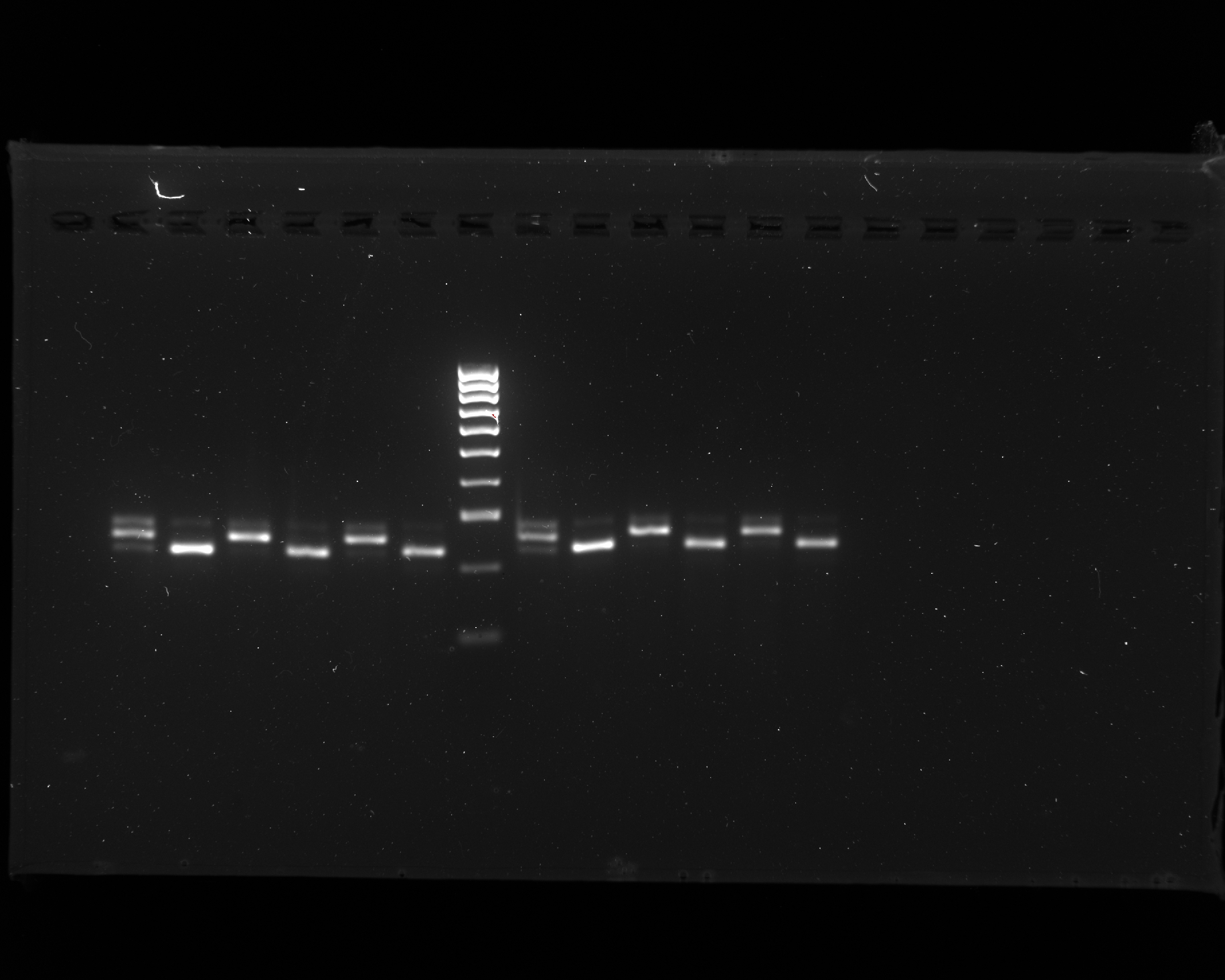

Supplement: Figure 7—source data 1. [file elife-96979-fig7-data1.zip › Figure 7_source data/Raw unedited gels for (Figure 7A)/aog 2023-04-25 13h57m06s(SYBR┬« Safe).jpg]

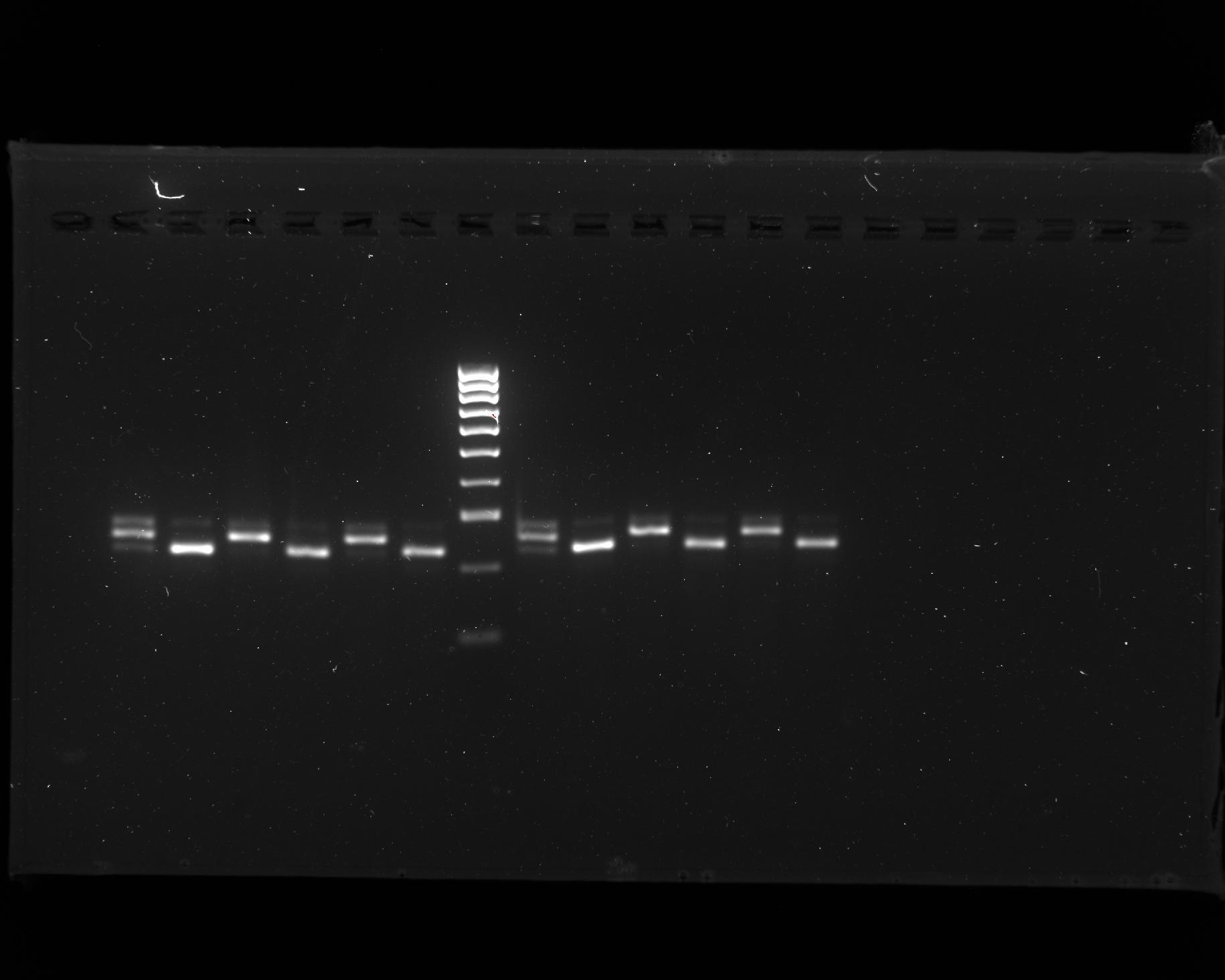

Supplement: Figure 7—source data 1. [file elife-96979-fig7-data1.zip › Figure 7_source data/Raw unedited gels for (Figure 7A)/aog 2023-04-25 13h57m06s(SYBR┬« Safe).tif]

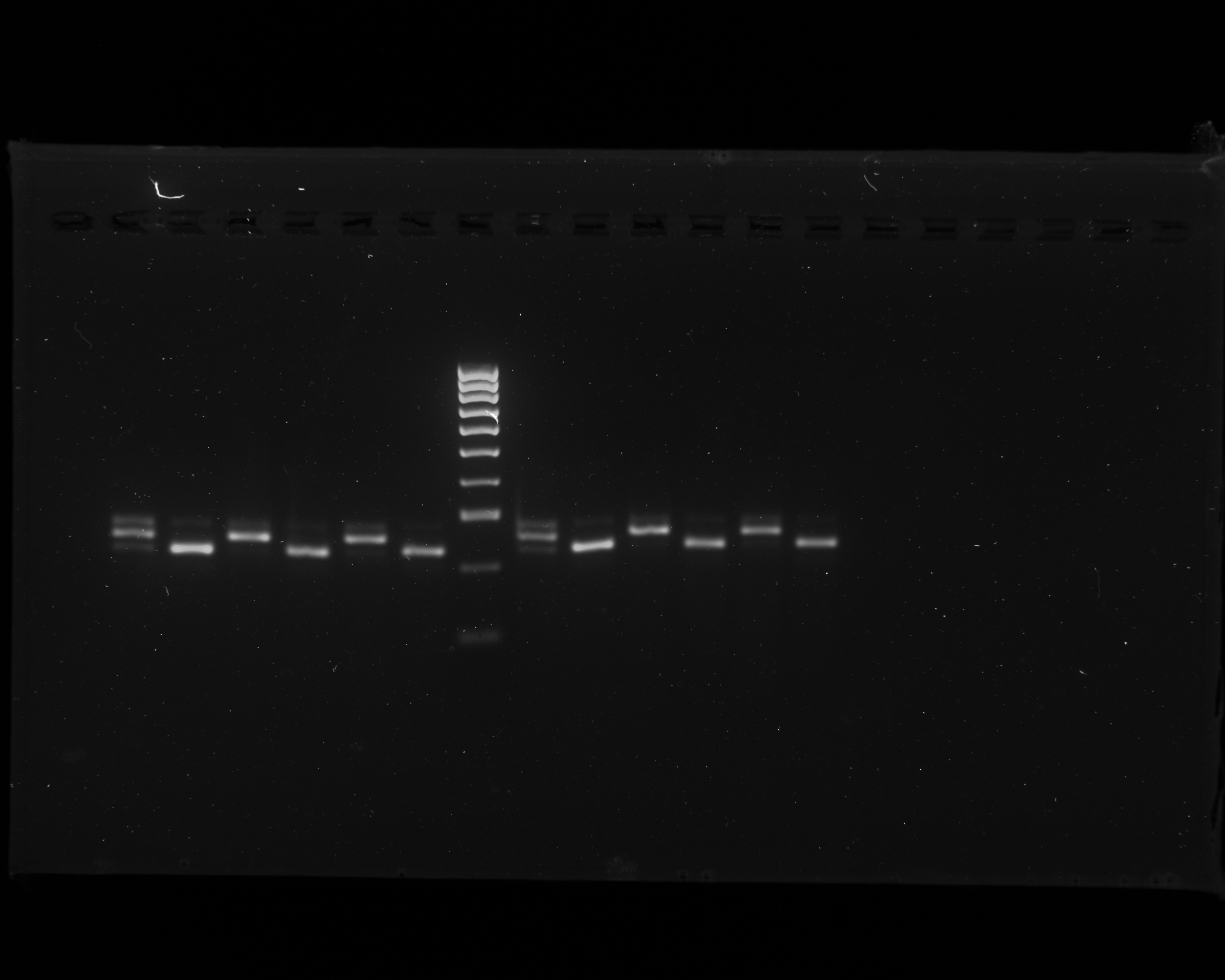

Supplement: Figure 7—source data 1. [file elife-96979-fig7-data1.zip › Figure 7_source data/Raw unedited gels for (Figure 7A)/aog 2023-04-25 13h57m06s(SYBR┬« Safe).raw16.tif]

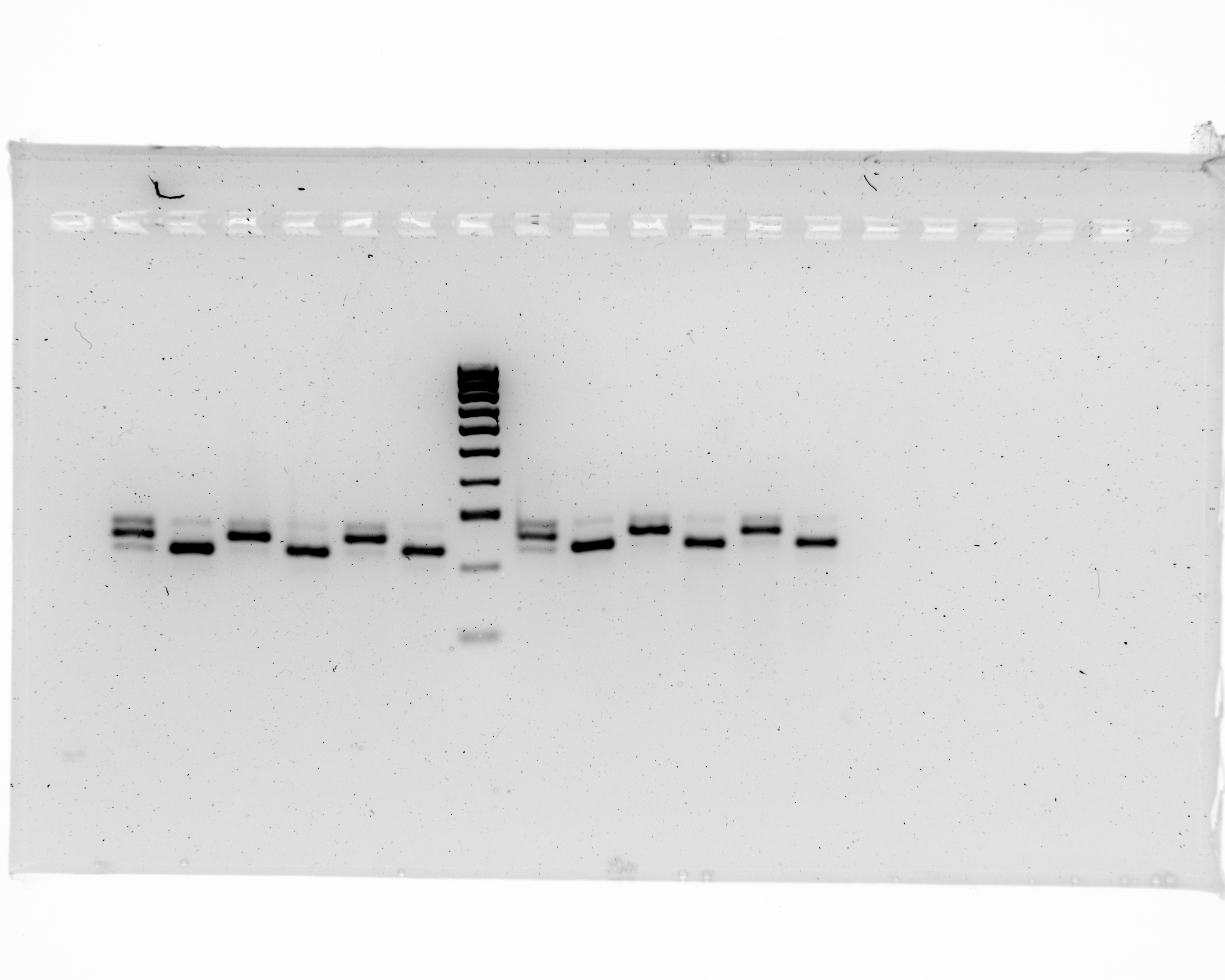

Supplement: Figure 7—source data 1. [file elife-96979-fig7-data1.zip › Figure 7_source data/Raw unedited gels for (Figure 7A)/aog 2023-04-25 13h57m06s(SYBR┬« Safe).raw16_Modified 2.tif]

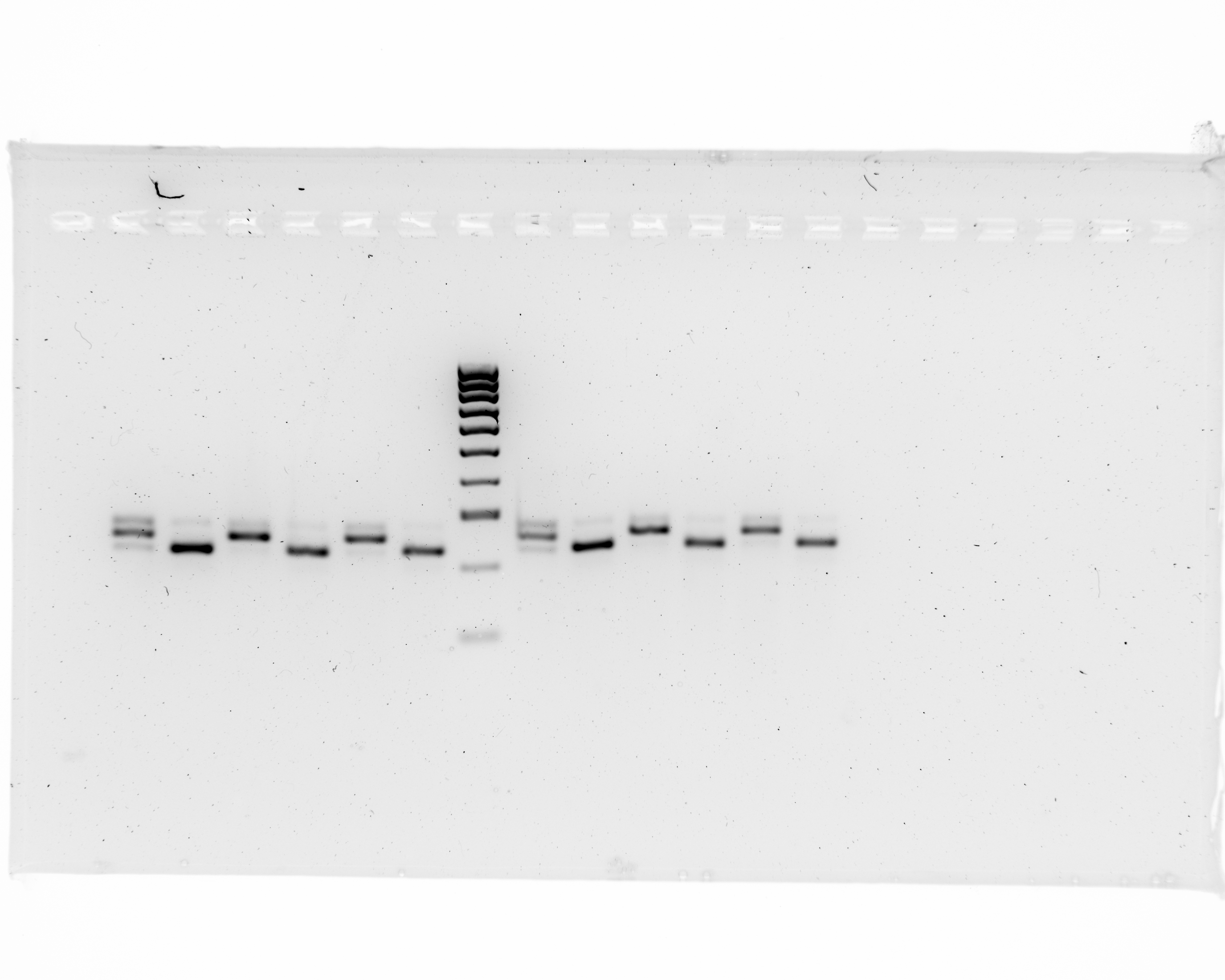

Supplement: Figure 7—source data 1. [file elife-96979-fig7-data1.zip › Figure 7_source data/Raw unedited gels for (Figure 7A)/aog 2023-04-25 13h57m06s(SYBR┬« Safe).raw16_MODIFIED.tif]

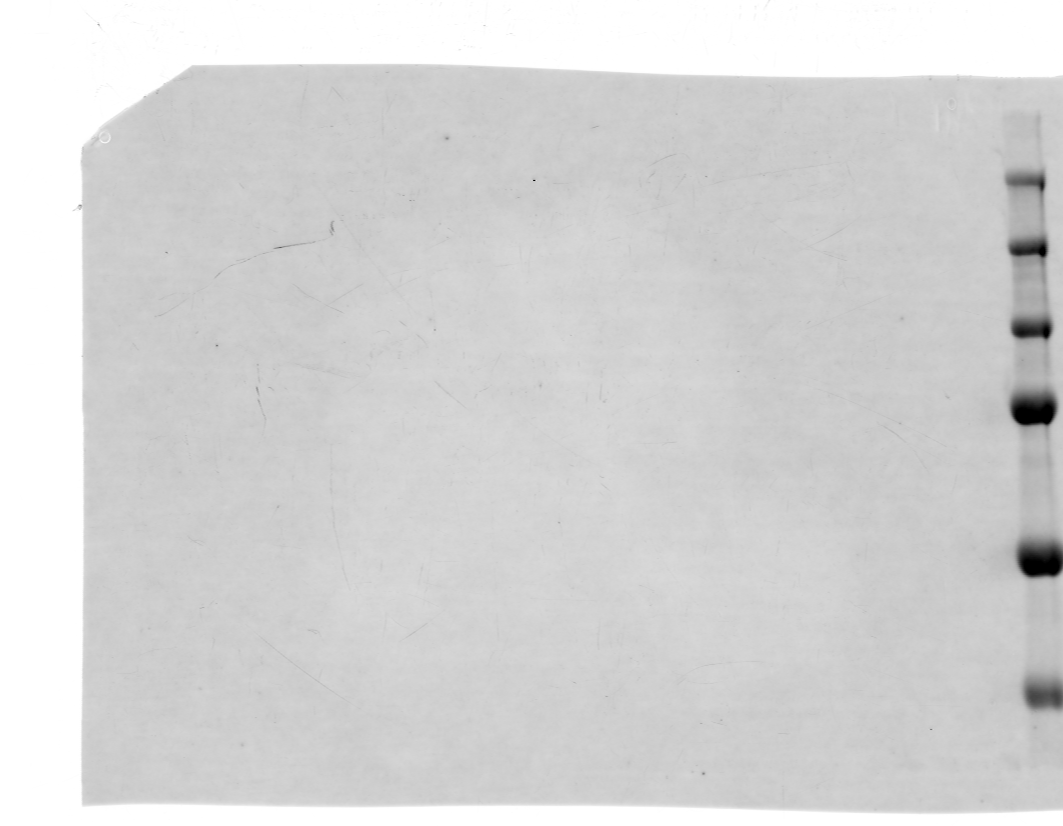

Supplement: Figure 7—source data 1. [file elife-96979-fig7-data1.zip › Figure 7_source data/Raw unedited gels for (Figure 7B)/Anti-IRE1/2023-07-26-160236/700modified.tif]

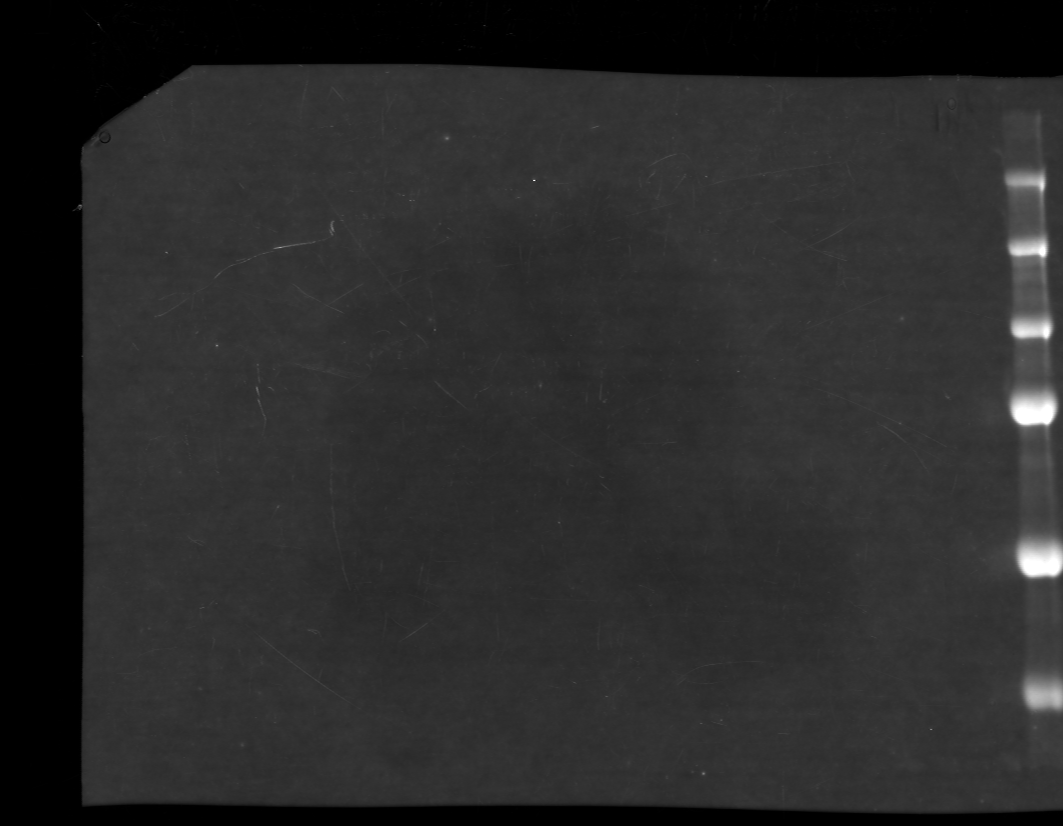

Supplement: Figure 7—source data 1. [file elife-96979-fig7-data1.zip › Figure 7_source data/Raw unedited gels for (Figure 7B)/Anti-IRE1/2023-07-26-160236/700.TIF]

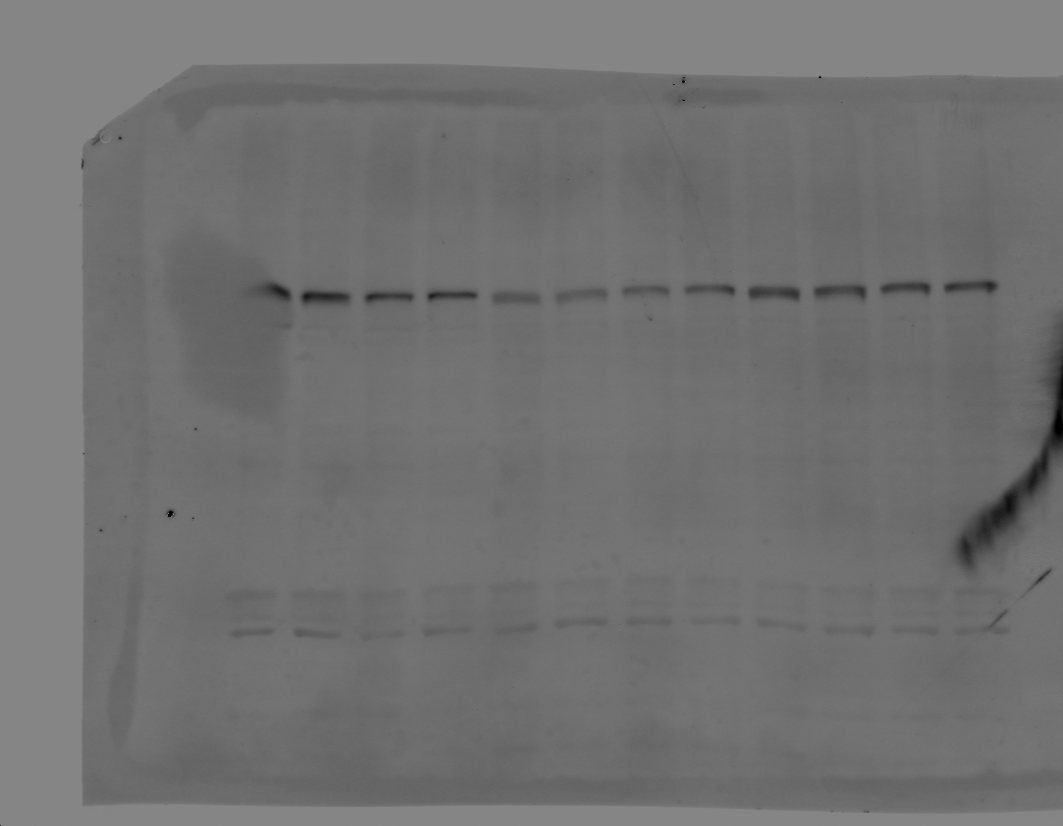

Supplement: Figure 7—source data 1. [file elife-96979-fig7-data1.zip › Figure 7_source data/Raw unedited gels for (Figure 7B)/Anti-IRE1/2023-07-26-160236/800modified.tif]

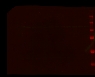

Supplement: Figure 7—source data 1. [file elife-96979-fig7-data1.zip › Figure 7_source data/Raw unedited gels for (Figure 7B)/Anti-IRE1/2023-07-26-160236/2023-07-26-160236_1_TH.jpg]

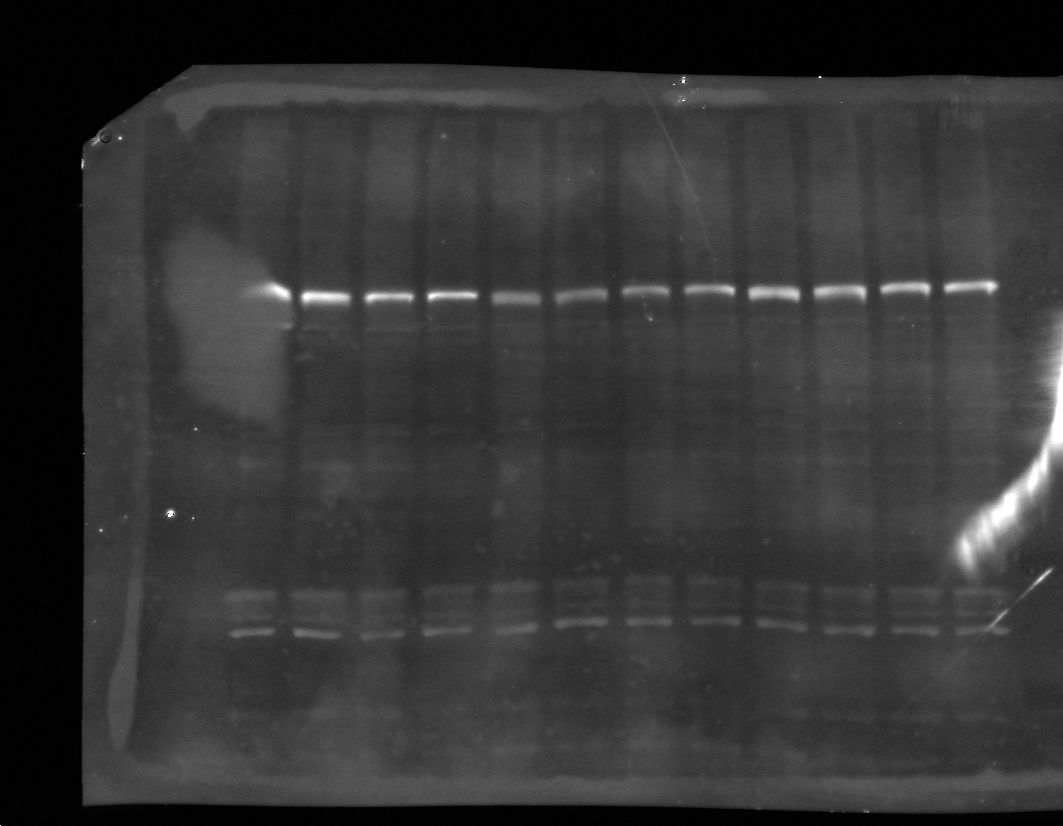

Supplement: Figure 7—source data 1. [file elife-96979-fig7-data1.zip › Figure 7_source data/Raw unedited gels for (Figure 7B)/Anti-IRE1/2023-07-26-160236/800.TIF]

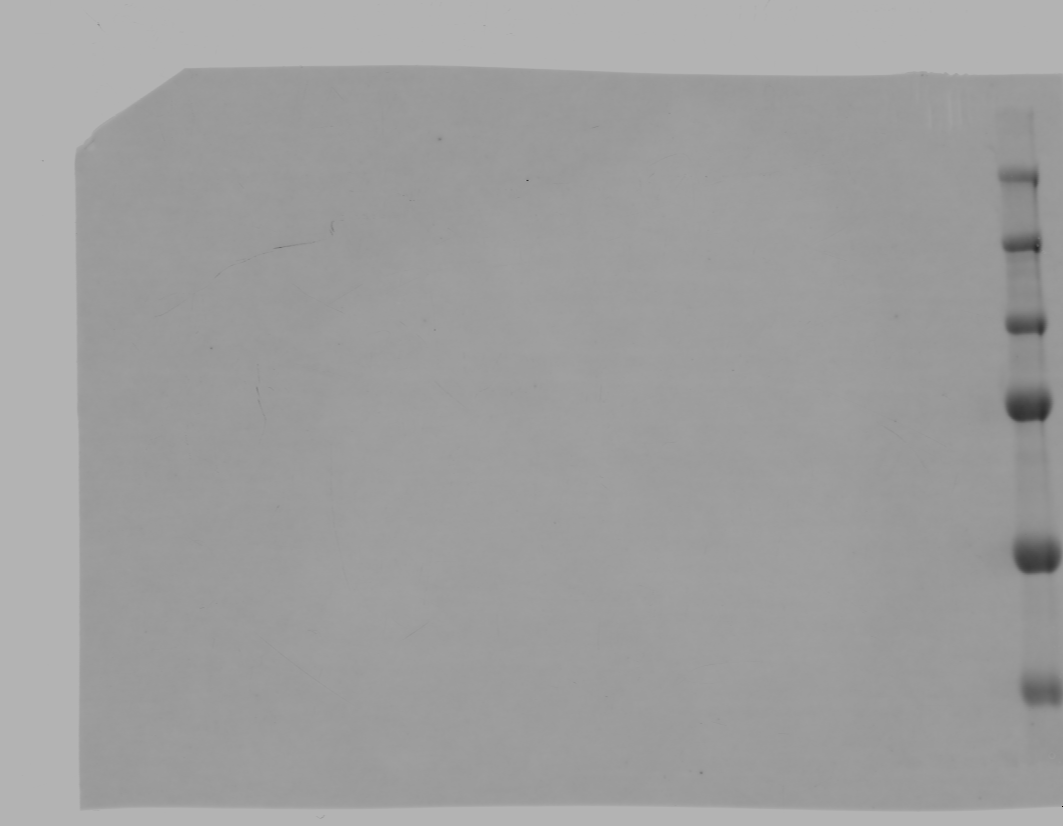

Supplement: Figure 7—source data 1. [file elife-96979-fig7-data1.zip › Figure 7_source data/Raw unedited gels for (Figure 7B)/Anti-Actin/2023-07-27-164026/700modified.tif]

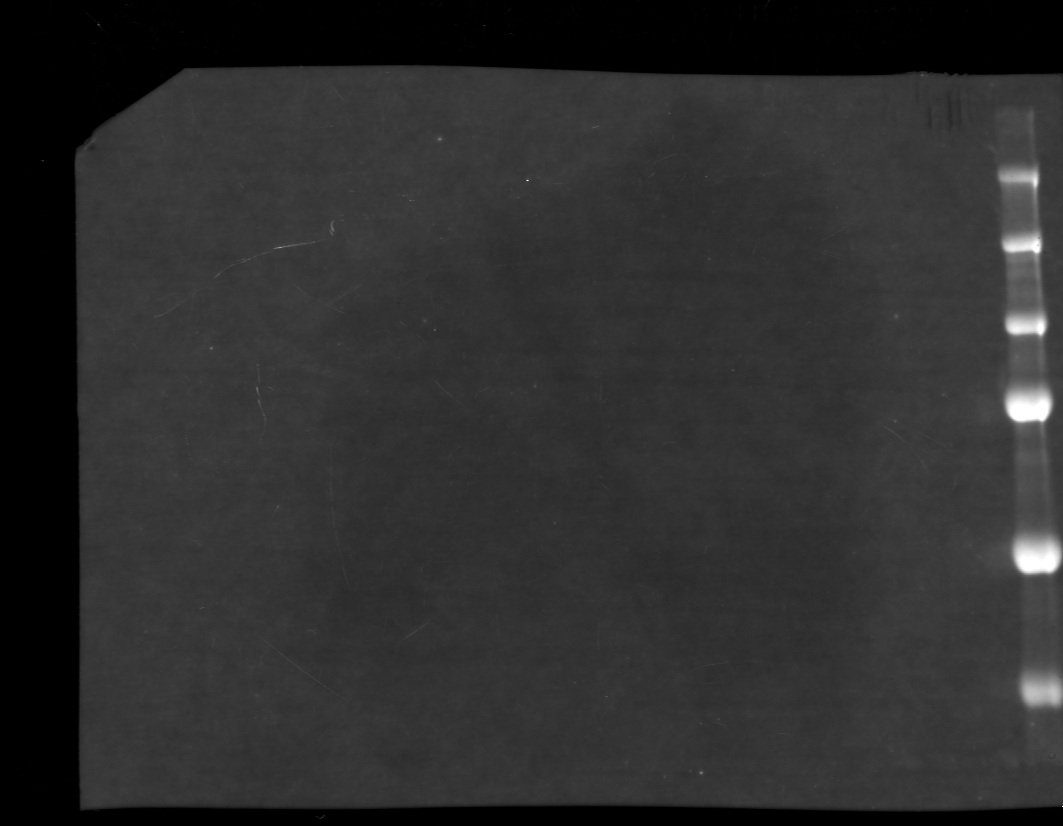

Supplement: Figure 7—source data 1. [file elife-96979-fig7-data1.zip › Figure 7_source data/Raw unedited gels for (Figure 7B)/Anti-Actin/2023-07-27-164026/700.TIF]

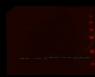

Supplement: Figure 7—source data 1. [file elife-96979-fig7-data1.zip › Figure 7_source data/Raw unedited gels for (Figure 7B)/Anti-Actin/2023-07-27-164026/2023-07-27-164026_1_TH.jpg]

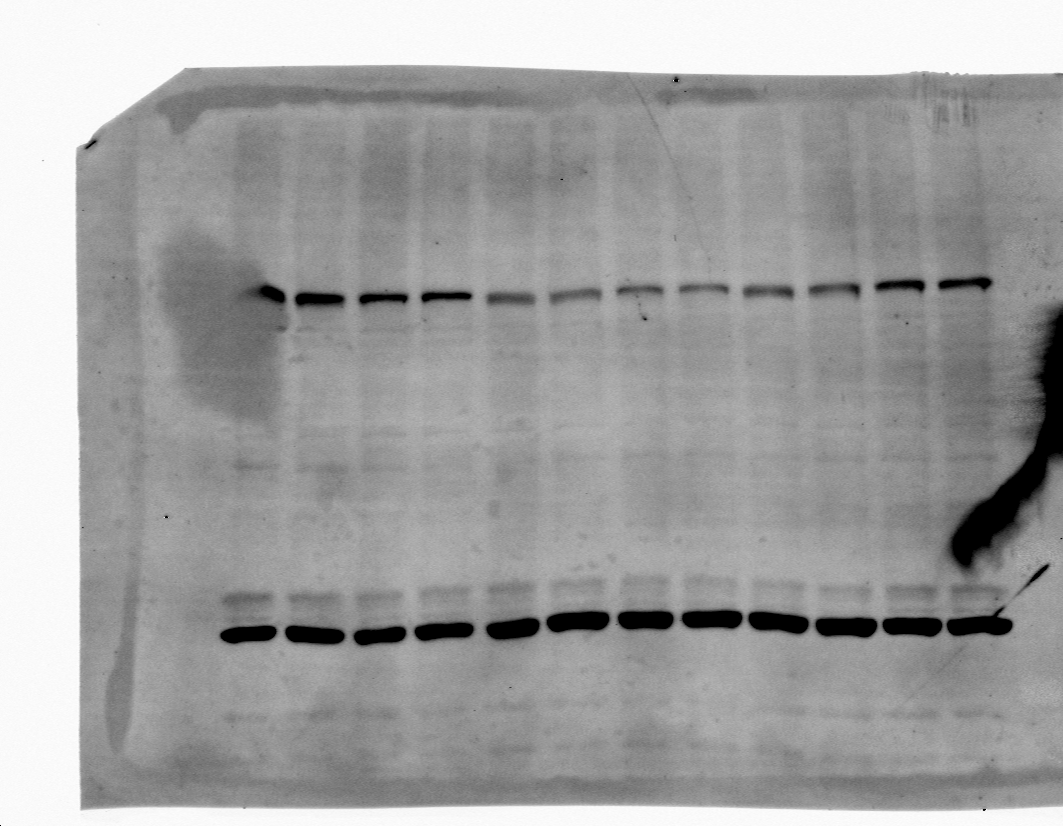

Supplement: Figure 7—source data 1. [file elife-96979-fig7-data1.zip › Figure 7_source data/Raw unedited gels for (Figure 7B)/Anti-Actin/2023-07-27-164026/800_Modified AO_for Ire1.tif]

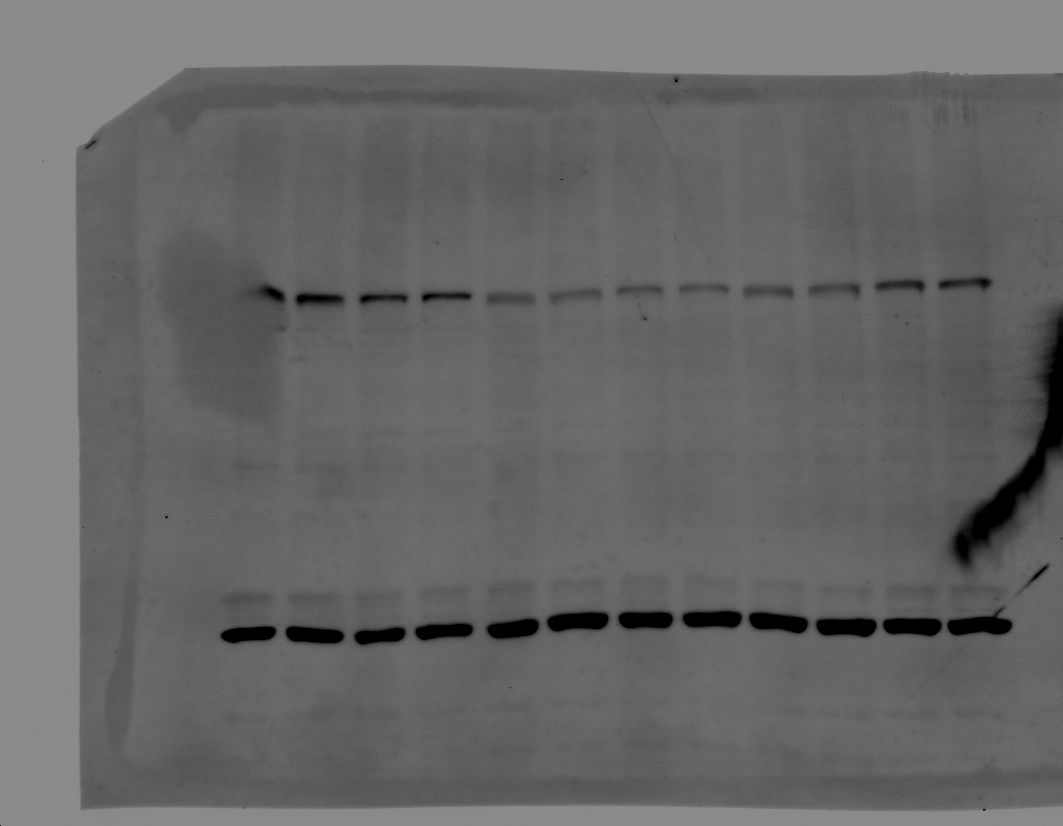

Supplement: Figure 7—source data 1. [file elife-96979-fig7-data1.zip › Figure 7_source data/Raw unedited gels for (Figure 7B)/Anti-Actin/2023-07-27-164026/800modified.tif]

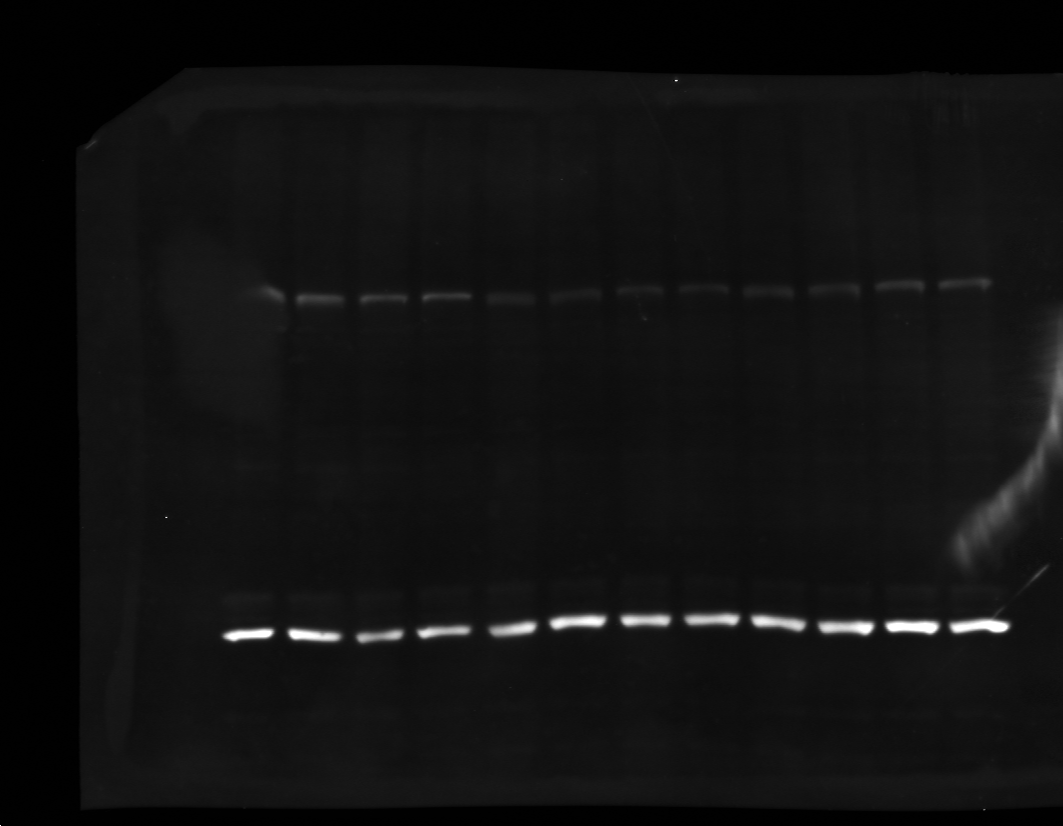

Supplement: Figure 7—source data 1. [file elife-96979-fig7-data1.zip › Figure 7_source data/Raw unedited gels for (Figure 7B)/Anti-Actin/2023-07-27-164026/800.TIF]

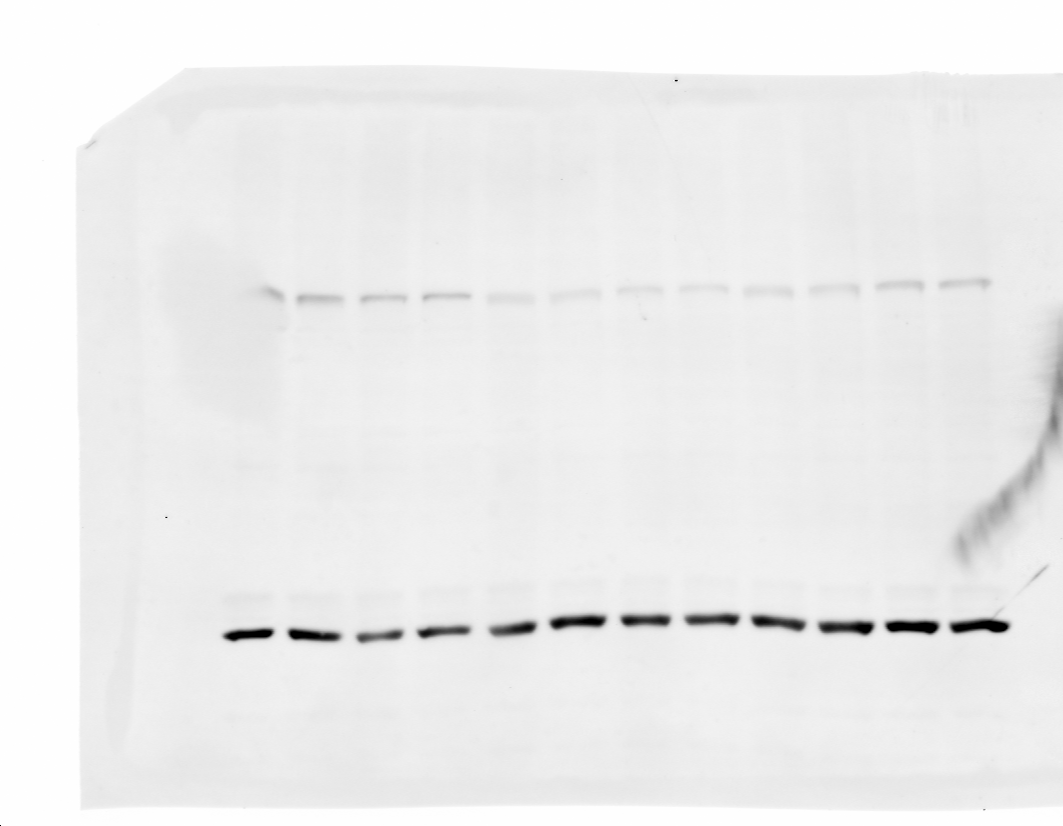

Supplement: Figure 7—source data 1. [file elife-96979-fig7-data1.zip › Figure 7_source data/Raw unedited gels for (Figure 7B)/Anti-Actin/2023-07-27-164026/800_Modified AO.tif]

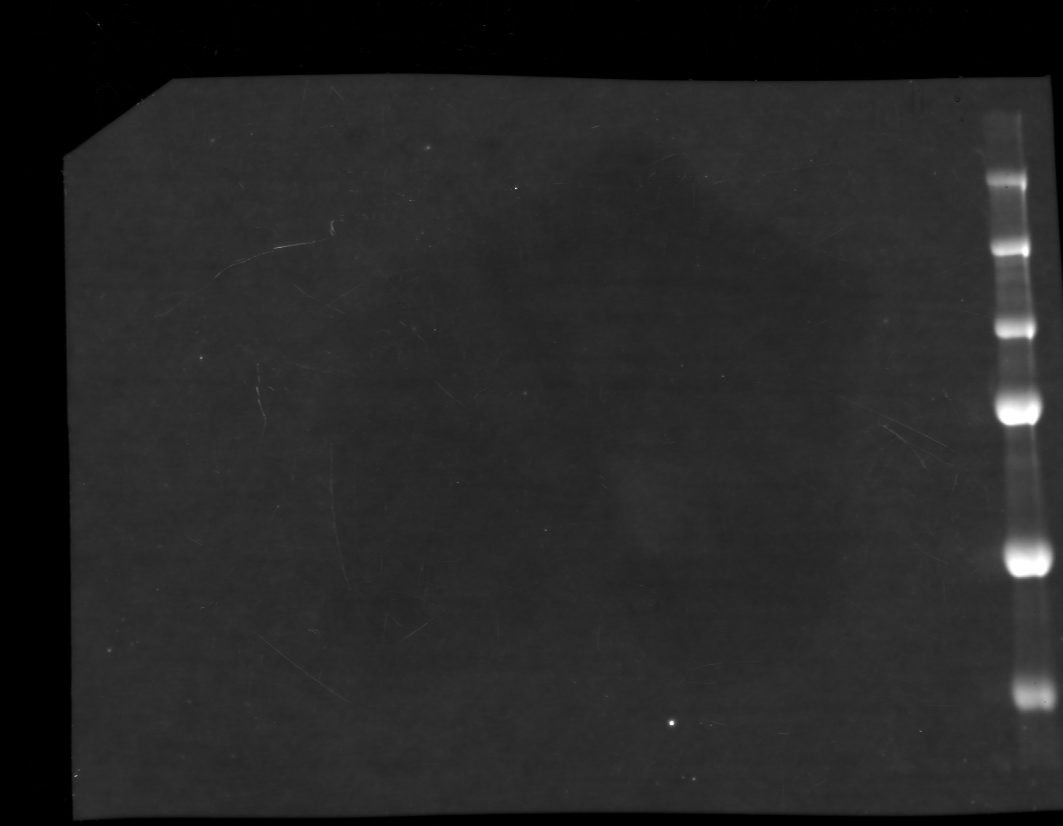

Supplement: Figure 7—source data 1. [file elife-96979-fig7-data1.zip › Figure 7_source data/Raw unedited gels for (Figure 7B)/Anti-pIRE1/2023-07-25-153135/700.TIF]

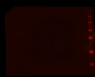

Supplement: Figure 7—source data 1. [file elife-96979-fig7-data1.zip › Figure 7_source data/Raw unedited gels for (Figure 7B)/Anti-pIRE1/2023-07-25-153135/2023-07-25-153135_230626_co-ip_2_trial_5_anti-G34_TH.jpg]

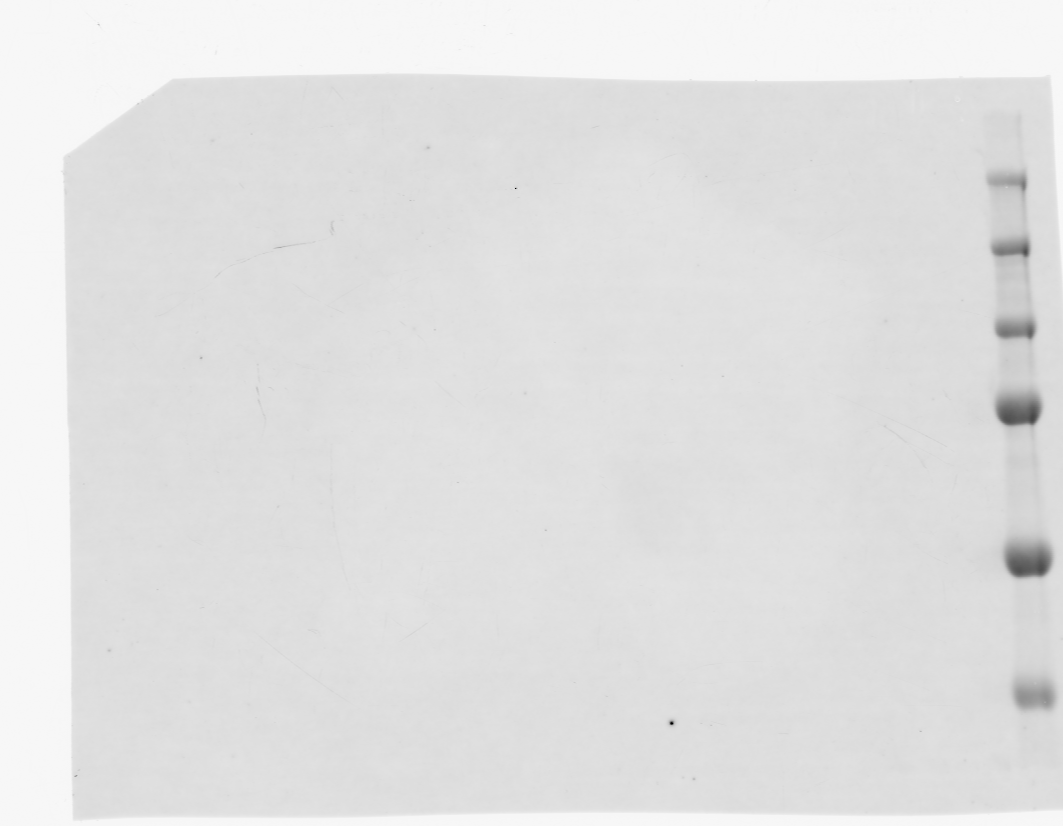

Supplement: Figure 7—source data 1. [file elife-96979-fig7-data1.zip › Figure 7_source data/Raw unedited gels for (Figure 7B)/Anti-pIRE1/2023-07-25-153135/700 MODIFIED.tif]

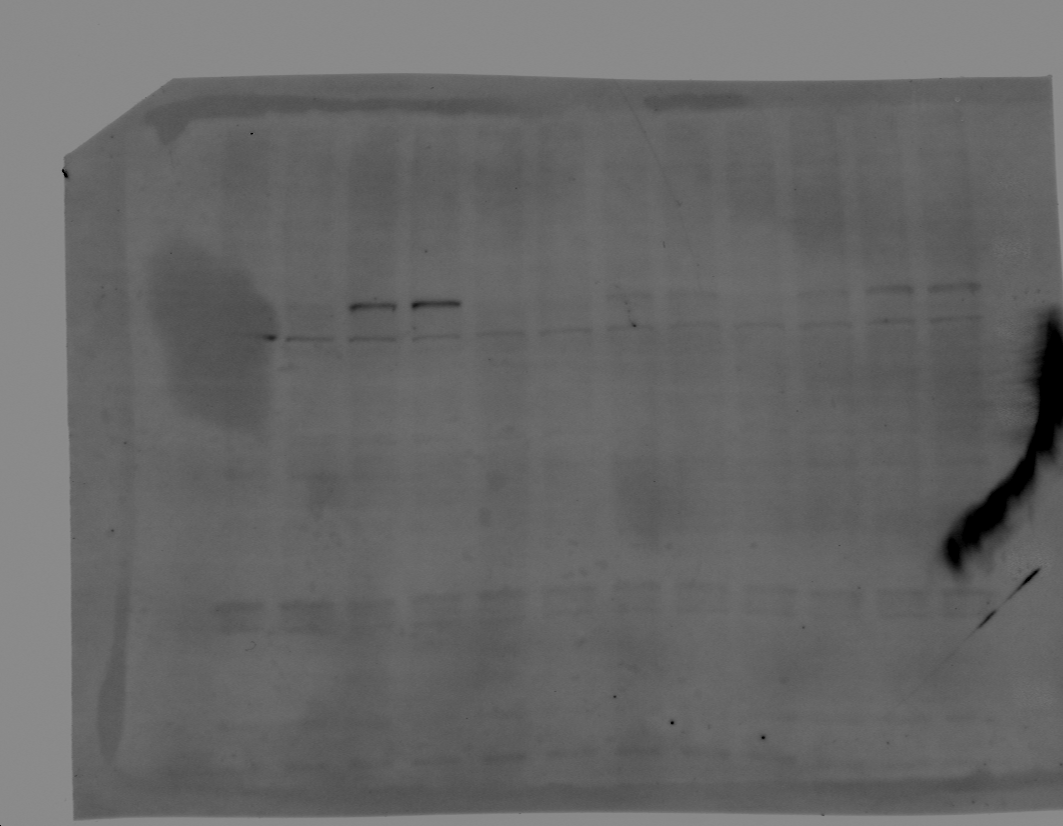

Supplement: Figure 7—source data 1. [file elife-96979-fig7-data1.zip › Figure 7_source data/Raw unedited gels for (Figure 7B)/Anti-pIRE1/2023-07-25-153135/800 MODIFIED.tif]

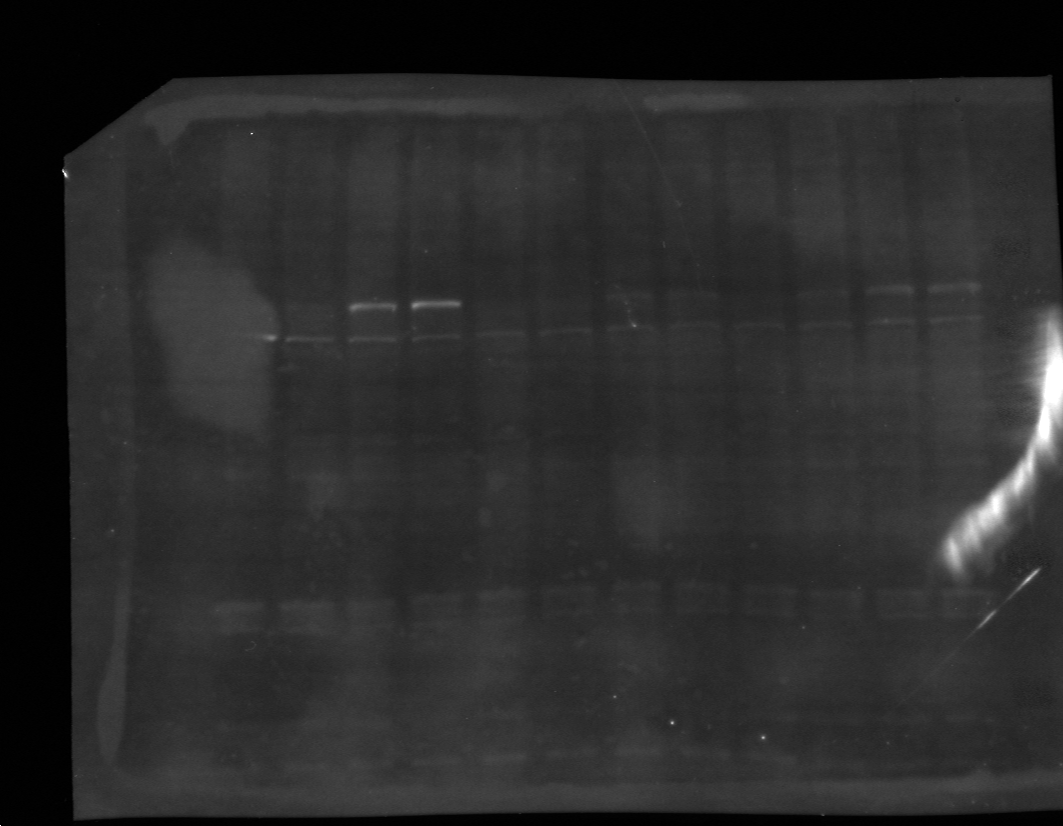

Supplement: Figure 7—source data 1. [file elife-96979-fig7-data1.zip › Figure 7_source data/Raw unedited gels for (Figure 7B)/Anti-pIRE1/2023-07-25-153135/800.TIF]

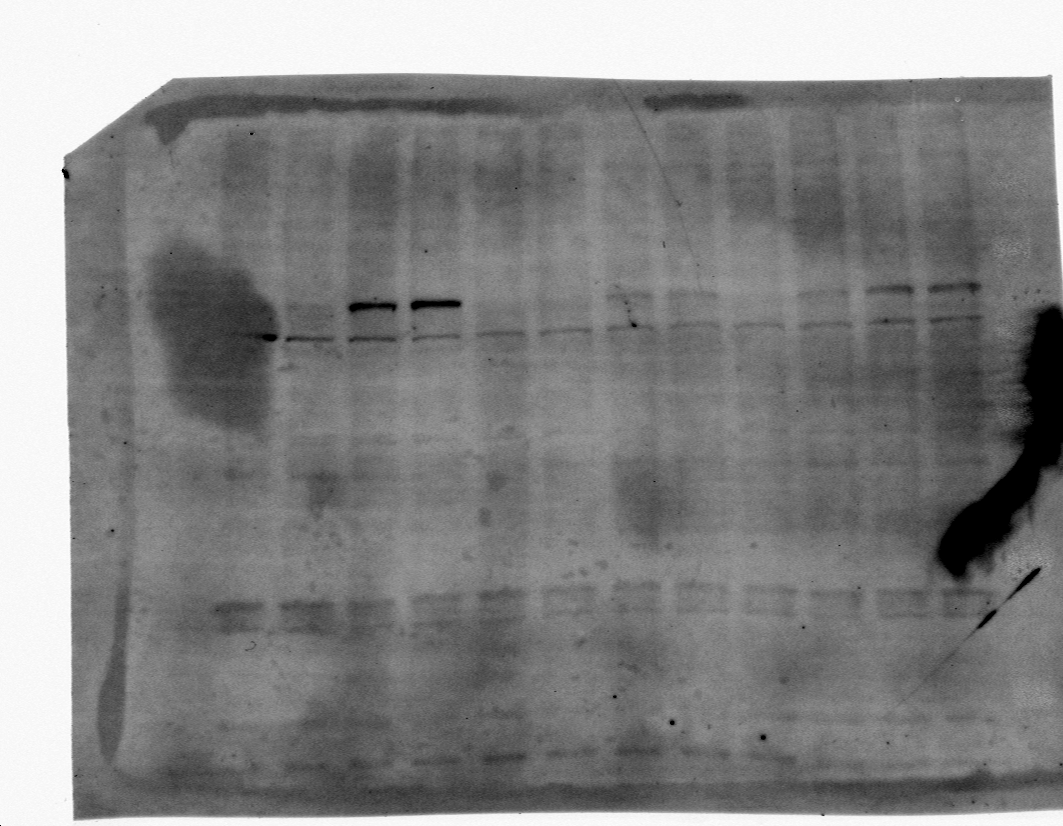

Supplement: Figure 7—source data 1. [file elife-96979-fig7-data1.zip › Figure 7_source data/Raw unedited gels for (Figure 7B)/Anti-pIRE1/2023-07-25-153135/800_modified AO.tif]
